# Aliphatic Amines Unlocked for Selective Transformations through Diazotization

**DOI:** 10.1002/anie.202419450

**Published:** 2024-12-12

**Authors:** Jakub Durka, Barbara Zielińska, Dorota Gryko

**Affiliations:** ^1^ Institute of Organic Chemistry Polish Academy of Sciences Kasprzaka 44/52 01-224 Warsaw Poland; ^2^ Department of Chemistry Warsaw University of Technology Noakowskiego 3 00-664 Warsaw Poland

**Keywords:** Aliphatic amines, Alkylation, Diazotization, Synthetic methods

## Abstract

While aromatic diazonium salts are important reagents in organic synthesis, *‘Diazonium ions generated from ordinary aliphatic primary amines are usually useless for preparative purposes, since they lead to a mixture of products giving not only substitution by any nucleophile present, but also elimination and rearrangements if the substrate permits*.’^1^ In this work, we report that this statement is no longer valid, and it is now possible to control diazotization of aliphatic amines by utilizing isopentyl nitrite in HFIP. This transformation enabled electrophilic aromatic substitution with these highly abundant and commercially available alkyl reagents, as well as transforming them into building blocks typically employed in organic synthesis. The methodology opens an avenue for reactions involving aliphatic amines, even such demanding substrates as amino acids, as a source of carbocations thus expanding the degree of chemical space.

## Graphical abstract

Despite the established state of knowledge, synthetically useful diazotation of aliphatic amines is possible. The key of this process is the use of 1,1,1,3,3,3‐hexafluoroisopropanol as a solvent. It improves the selectivity of the process and enables the transformation of the intermediates into the desired Friedel–Crafts products. In this way, after more than 150 years, also the diazotation of aliphatic amines is gaining its place in synthesis.

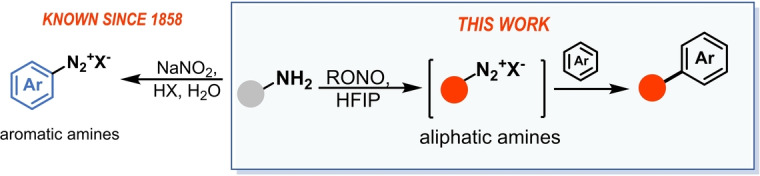

## Introduction

**Scheme 1:**
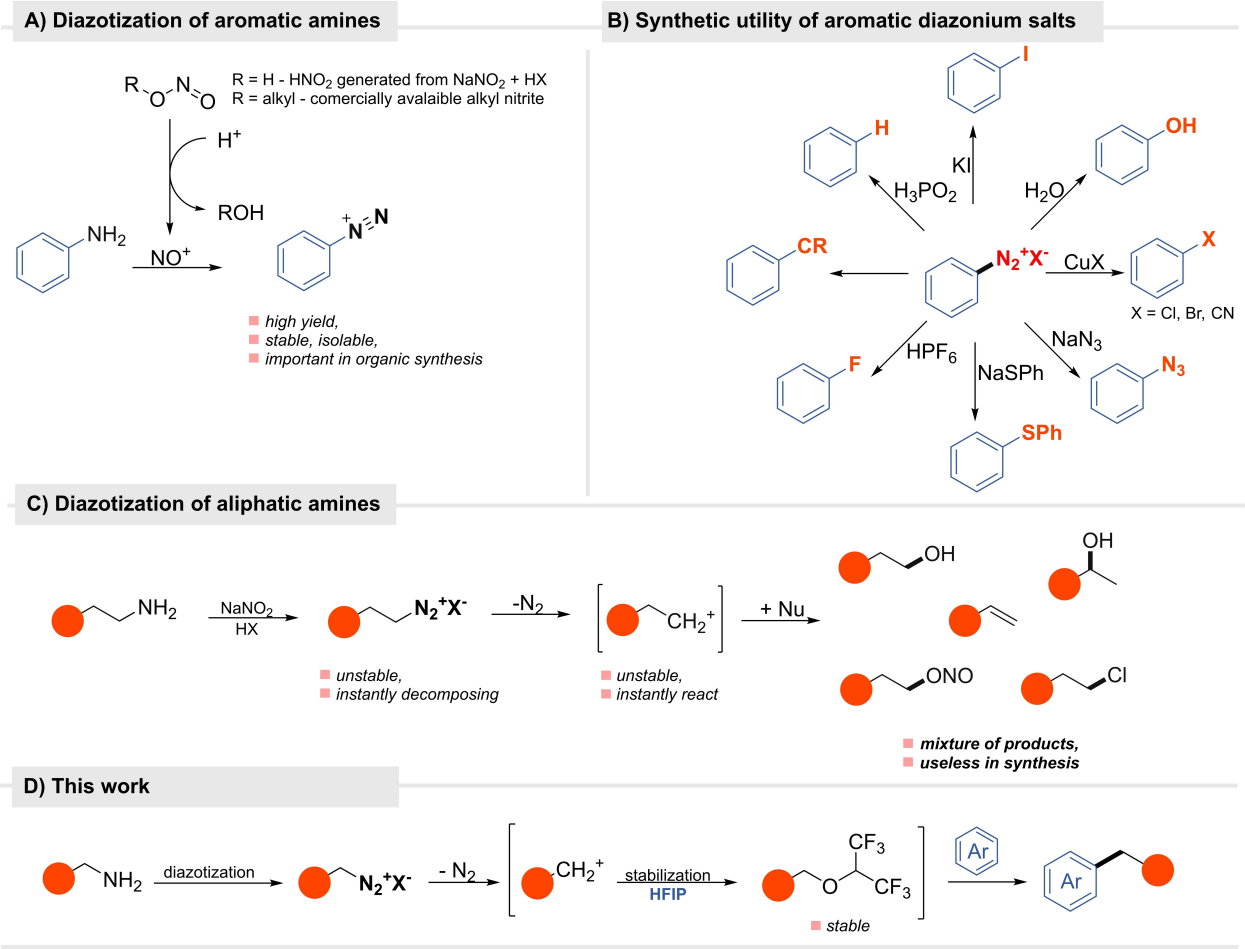
Diazotization of amines.

Amines play a vital role in nature and have found numerous applications in the chemical industry.[^1^, ^2^] Although they are produced on a large scale, they are mainly utilized in C−N bond forming reactions and rarely serve for the primary purpose of organic synthesis, which is to create new C−C bonds.^[3]^ By expanding the scope of these transformations, these highly abundant and bench‐stable reagents would immensely enhance the pool of available building blocks. Along these lines, several methods have been developed, but the most studied and widely applied is diazotization.[^4^, ^5^] Developed in the 19th century, this reaction transforms aromatic amines into stable diazonium salts that are widely used as synthetic intermediates since N_2_ is such a great leaving group (Scheme  1A).^[6]^ A diazotization/dediazotization strategy allows for the replacement of the ‐NH_2_ functionality with other substituents like −H, −F, −Cl, −Br, −I, −CN, −OH, −SPh, alkyl, aryl, etc (Scheme  1B).[^5^, ^6^] Such reactions are, however, limited to aromatic amines. For aliphatic analogs, these reactions are commonly considered nonselective.[^3^, ^7^] Aliphatic diazonium salts lack stabilization and therefore immediately release a nitrogen molecule generating a carbocation which captures any nucleophile present in the reaction medium (water, nitrite, the anion of the acid used, etc., Scheme  1C) to give a mixture of products, loses proton in elimination processes, or rearranges.[^8^, ^9^] Consequently, this reaction is regarded as non‐practical in organic synthesis; instead, other sources of carbocations that include alkenes, alcohols, or alkyl halides in the presence of acidic catalysts are employed.^[10]^

Diazotization reactions of aliphatic amines typically result in complex mixtures of products but over the last 140 years, isolated examples of selective reactions have been reported, usually involving intramolecular transformations. For example, aminomethyl‐cycloalkanes undergo the Demjanov rearrangement leading to alcohols. Enlarged rings, ranging from cyclobutane to cyclononane, have been successfully obtained using this method. The Tiffeneau‐Demjanov rearrangement proceeds similarly, diazotization of 1‐(aminomethyl)‐cycloalkanols gives access to ring‐expanded ketones.^[11]^ Furthermore, diazonium salts derived from α‐amino acids do not decompose into a carbocation, but instead intramolecular nucleophilic attack by the neighboring carboxylate furnishes α‐lactone. The subsequent S_N_2 reaction with the concomitant strain‐release gives access to optically pure 2‐halo‐ and 2‐hydroxy acids. Due to the non‐cationic mechanism, the reaction is stereoselective and occurs with retention of configuration.[^12^, ^13^] For diazonium salts with other electron‐withdrawing groups, the intramolecular nucleophilic substitution cannot occur, instead deprotonation (if a hydrogen atom is present at the α‐position), leading to the formation of stabilized diazo compounds, is taking place.[^14^, ^15^] These compounds have gained huge importance in organic synthesis, commonly used in reactions catalyzed by transition metals, or induced by light.^[16]^ While, in general, diazotization reactions in the presence of thiols resulted in the formation of disulfides, there are exceptions, as in the case of 2‐mercaptobenzothiazole that selectively gives sulfides.^[17]^

*Although isolated examples for selective diazotization of aliphatic amines do exist,*[^10^, ^11^, ^12^, ^13^, ^17^] *a general method for simple aliphatic amines remains to be found*. With this goal in mind, we challenged ourselves to develop a system enabling selective deaminative transformations of aliphatic amines via a diazotization/dediazotization strategy. We hypothesized that this transformation could be realized if the carbocation generated from a diazonium salt is transiently transformed into an intermediate that subsequently can participate in the electrophilic aromatic substitution in the reaction system/ environment. We have found that the use of HFIP for the diazotization step assures the selectivity of the Friedel–Crafts alkylation, halogenation, carboxylation reactions (Scheme  1D). This method extends the diazonization strategy to aliphatic amines.

## Results and Discussion

### Reaction Design and Optimization of the Reaction Conditions

Aliphatic amines easily form diazonium salts. These are extremely reactive species and thus are challenging to handle and utilize in controlled synthetic procedures. The diazotization/dediazotization selectivity could potentially be enhanced by a) stabilization of the carbocation, b) limiting the number of available nucleophiles,and/ or c) reducing the nucleophilicity of the system. It is well‐documented that fluorinated alcohols can dramatically influence the outcomes of chemical reactions by, among many effects, acting as mild acid catalysts or stabilizing cationic intermediates.^[18]^ This proved to be particularly crucial in reactions with alcohols.[^19^, ^20^] Recently, Lebauf, Moran, and co‐workers have shown that in the Friedel–Crafts arylation with primary aliphatic alcohols and epoxides, a protonated cluster of HFIP molecules acts as a Brønsted acid and lowers the kinetic barriers associated with dearomatization and assures selectivity.^[21]^ We thus anticipated that by stabilizing carbocations, hexafluoroisopropanol (HFIP, **1**)^[22]^ might also have a beneficial effect on reactions of aliphatic amines involving diazonium salts.

HFIP should impart the selective diazotization/dediazotization of aliphatic amines because:

- due to it is high polarity and mild acidity (p*K*_a_=9.3), it serves as an excellent hydrogen bond donor providing extensive stabilization of carbocations.^[18]^ It has been frequently employed in reactions involving these reactive intermediates, for example in Friedel–Crafts type transformations with alcohols,[^23^, ^24^, ^25^, ^26^, ^27^]
- due to its acidity, the generation of highly stabilized carbocations obviates the need for the addition of classically used Brønsted or Lewis acids, hence, ensuring milder reaction conditions.[^19^, ^28^] For example, polymerization of styrenes in this solvent occurs without any stimuli.^[29]^ In our case, it should facilitate the generation of the nitrosonium cation from alkyl nitrites. Consequently, the alkyl nitrite would be the sole reagent in the reaction, in addition to amine, thereby limiting the number of nucleophiles present in the solution,
- due to its exceptionally low nucleophilicity; the inductive effect of the two −CF_3_ groups renders HFIP much less nucleophilic than alcohol or water,^[18]^ typically used in diazotization reactions, thus side reactions (related to solvent) should be limited,
- according to the data available in the Mayr's Database of Reactivity Parameters, not only does HFIP itself exhibit low nucleophilicity, but also it is capable of reducing the nucleophilicity of other molecules present in the solution with which it forms hydrogen bonds.^[30]^ This is particularly significant as the complete elimination of nucleophiles from the diazotization reaction is impossible; by definition, 1) the respective alcohol is a byproduct formed from the nitrite, 2) during the reaction an equivalent of water is released,
- once the carbocation is generated from a diazonium salt, it may react with nucleophilic species (HFIP, alcohol, water) affording by‐products (hexafluoroisopropyl ether, alkyl ether, and alcohol). However, HFIP is known to facilitate acid‐catalyzed C−O bond cleavage regenerating carbocationic intermediates from such species.[^21^, ^24^, ^31^] Therefore, we anticipated that the addition of an acid would assure their transformation to the desired product.

**Scheme 2:**
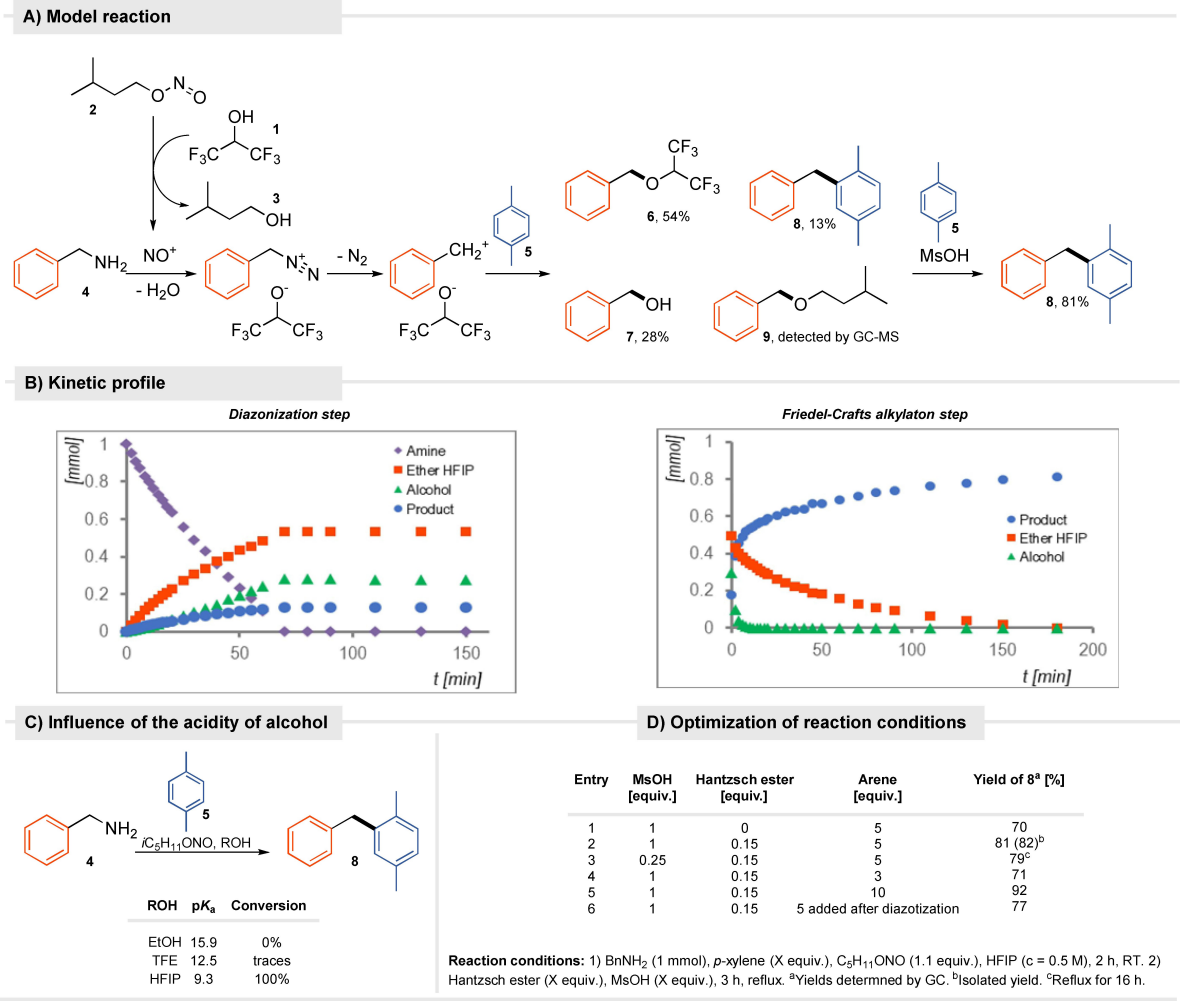
Reaction design and optimization studies.

To verify our hypothesis, we performed the model reaction of benzylamine (**4**) with isopentyl nitrite (**2**) and *p*‐ xylene (**5**) in HFIP which gave, the desired Friedel–Crafts derivative but as a minor product **8**, (13%), instead, benzyl HFIP ether **6** (54%), and benzyl alcohol (**7**, 28%) were observed (Scheme  2A). The GC‐MS analysis additionally revealed the formation of benzyl isopentyl ether **9** though in traces. Of course, this ratio of primary products depends on the amine used and the arene activity – for example, for 4‐methoxybenzylamine, the Friedel–Crafts alkylation was already the main process. These experimental data indicate that the acidity of HFIP itself is sufficient for complete conversion of the amine without the need for the addition of an acid. As a consequence, diazotization can be conducted under mild conditions, limiting the formation of by‐products, such as nitroso arenes.

Furthermore, the impact of the HFIP acidity is corroborated by the results obtained for the model reaction in other alcohols (Scheme  2C). Practically no conversion was observed for less acidic trifluoroethanol (p*K*_a_ = 12.5), while diazotization does not occur at all in ethanol.

The kinetic profile of the diazotization shows that hexafluoroisopropyl ether and the alkylated xylene are produced proportionally over time, while the rate of the benzyl alcohol formation slightly accelerates as the concentration of water increases during the reaction course (Scheme  2B).

The resulting products are stable in the reaction medium. Along this line, Lebauf, Moran, and co‐workers reported that hexafluoro isopropyl ethers form rapidly during hydroarylation of enamides and serve as a slow‐release reservoir for cations.^[31]^ Waldvogel regarded benzyl hexafluoro isopropyl ether as a molecular mask for the benzylic cation.^[32]^ Acid catalysis in HFIP has been documented to trigger transformations with alcohols and ethers. Thus, to push forward the Friedel‐Crafts alkylation with transient ethers, the addition of an acid seemed required. In fact, the addition of MsOH (1 equiv.) increased the yield up to 70%. Kinetic studies revealed that electrophilic aromatic substitution is much faster for alcohol than for the electron deficient hexafluoro isopropyl ether (Scheme 2B). Indeed, the results corroborate our hypothesis that under the appropriate conditions aliphatic amines can serve as a useful source of carbocations, thus giving a solution to the problem that has drawn the interest of many groups over the years. The reaction developed proceeds in a two‐step one‐pot sequence with one purification step.

Next, efforts were made to increase the reaction yield and optimize its conditions (Scheme 2D, for details see Supplementary Section 3.9, page 8). Among the nitrosating agents (*t*BuONO, NBu_4_NO_2_, NMe_4_NO_2_, NaNO_2_), *iso*pentyl nitrite proved to be the most versatile when used in a slight excess. The GC analysis of the crude reaction mixture revealed the formation of benzaldehyde. Presumably, after the addition of the acid, the remaining RONO oxidizes the alcohol formed. As a remedy to this issue, the addition of a reducing agent was foreseen. The Hantzsch ester (0.15 equiv., entry 2) acted most effectively. From now, the only side products observed in the model reaction resulted from subsequent benzylation of **8**, a known side reaction in electrophilic aromatic substitution. Typically, for Friedel–Crafts alkylation, it is beneficial to use an excess of the aromatic substrate. The yield gradually increased from 71% to 92% as the amount of *p*‐xylene (**5**) increased (from 3 to 10 equivalents, entries 4–5). Importantly, the reaction yield remained almost the same regardless of whether the aromatic substrate was added before or after the diazotization reaction (entries 2, 6). This feature enables the use of highly active aromatic substrates, which are susceptible to nitrosation or oxidation under such mild conditions. It was observed that for generating less stabilized carbocations it is beneficial to use TfOH over MsOH.

*Optimized Conditions*: Reaction conditions: 1) BnNH_2_ (**4**, 1 mmol), *p*‐xylene (**5**, 5 equiv.), C_5_H_11_ONO (**2**, 1.1 equiv.), HFIP (**1**, *c* = 0.5 M), 2 h, RT. 2) Hantzsch ester (0.15 equiv.), MsOH (1 equiv.), 3 h, reflux.

### Scope and Limitations

**Scheme 3:**
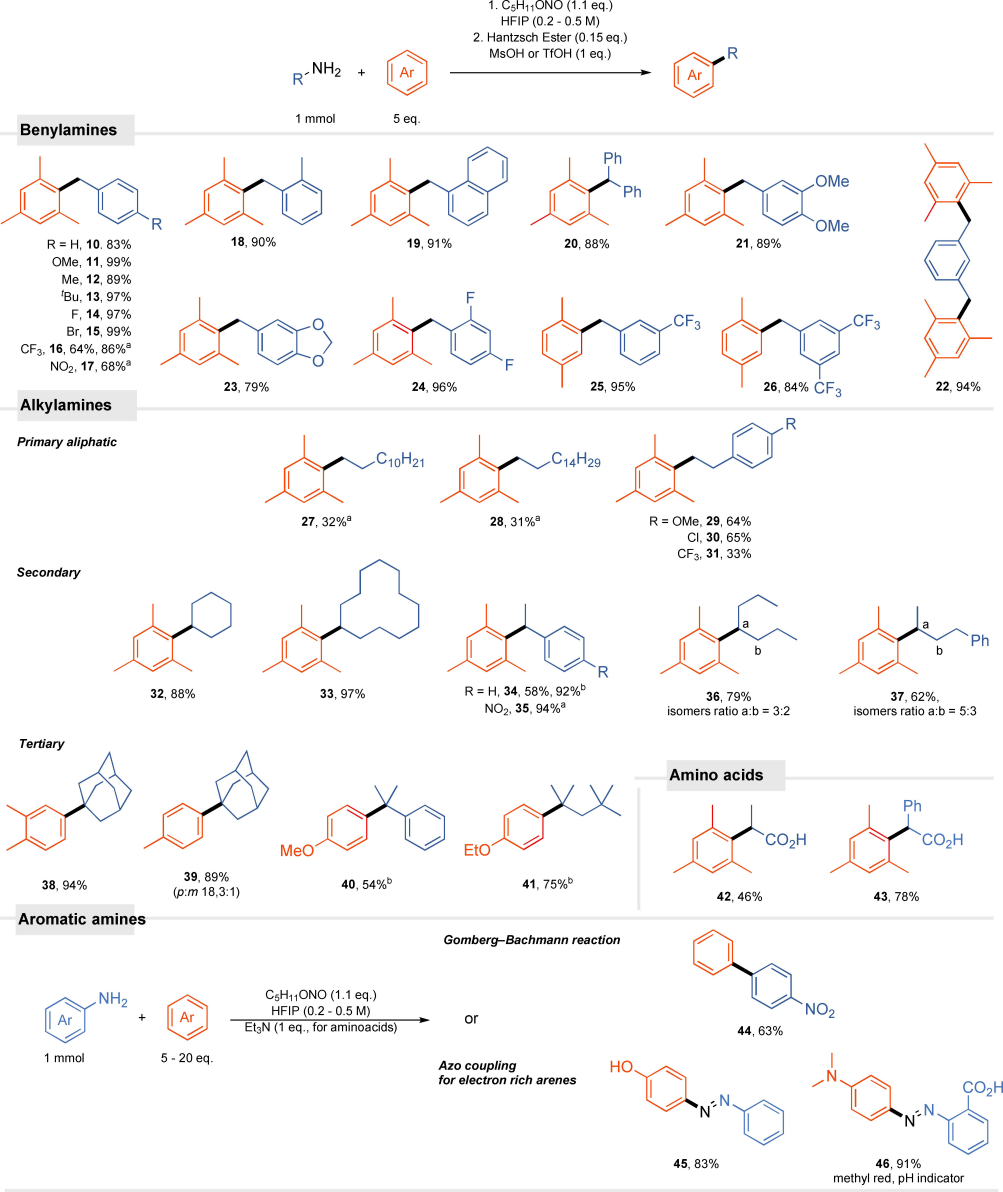
Friedel–Crafts alkylation with various aliphatic amines–scope and limitations. ^a^Reaction conditions: 1) amine (1 mmol), AcOH (1.5 equiv.), C_5_H_11_ONO (1.1 equiv.), DCE (c = 0.5 M). 2) Hantzsch ester (0.15 equiv.), HFIP (4 mL), arene (5 equiv.), MsOH or TfOH (1 equiv.). ^b^NBu_4_Br (1 equiv.) also added to the second step. Amino acids reaction conditions: amino acid (1 mmol), arene (20 equiv.), C_5_H_11_ONO (1.1 equiv.), Et_3_N (1 equiv.), HFIP (c = 0.17 M).

With the optimized conditions in hand, the scope of the Friedel–Crafts alkylation was evaluated. Electron‐rich and electron‐deficient benzylamines give desired products in good to excellent yields regardless of the substitution pattern. Even 3,5‐bisCF_3_‐ or 4‐CF_3_‐benzylamines react efficiently with alkyl benzenes (Scheme  3). Product **16** degrades with time under the reaction conditions but the replacement of HFIP with AcOH in the diazotization step mitigates this issue. The acetate formed in this process generates a cation more easily compared to HFIP ether. Furthermore, steric hindrance imposed by the methyl group at the *ortho* position of benzylamine does not interfere with the process. Despite acidic conditions and alcoholic solvent, the acetal group is not cleaved (**23**).

Importantly, unstabilized linear alkylamines are suitable starting materials in the developed method. Under the optimized diazotization conditions, hexadecylamine gives a complex mixture of products; the modified protocol (AcOH in the place of HFIP for diazotization), however, ensures the formation of the desired product **28** in serviceable yield (31%). Here, significantly higher nucleophilicity of the acetate anion compared to HFIP ensures the formation of a transient intermediate ester before side rearrangements take place. Phenylethylamines performed much better, forming the corresponding products in good yields, presumably because of the mechanism involving the stabilized phenonium cation postulated by Lebauf and Moran as an intermediate in the dehydroxylative arylation of phenylethanols.^[21]^ The use of the diazotization process enabled the synthesis of products with an electron withdrawing group at the phenyl ring **31**, which is not accessible via methods using alcohols as substrates.^[21]^ In this case, a by‐product resulting from the rearrangement to the stabilized benzyl cation forms. α‐Secondary amines are also effective as alkylating reagents in the developed Friedel–Crafts alkylation.

Cycloalkyl reagents furnish products **32**, **33** in high yields whilst for linear secondary amines, a mixture of compounds forms due to a classical carbocation rearrangement. For 1‐phenylethylamine, formation of styrene, a great electrophile acceptor leading to oligomerization, competes with the desired reaction pathway. In this case, the yield of 58% improves significantly upon the addition of NBu_4_Br (1 equiv.). Assumingly, it prevents styrene oligomerization by transforming it into benzylic bromide that subsequently reacts via the S_N_1 mechanism to give the final product. Furthermore, the Friedel–Crafts functionalization of arenes with tertiary alkyl carbocations is also possible as represented by reactions with adamantylamine. Non‐cyclic substrates also required the addition of bromide, as previously described, to achieve alkylated arenes in good yields (**40**, **41**).

As mentioned earlier, amino acids are unique substrates for which diazotization reactions can be carried out with synthetically useful yields. The reaction is, however, usually performed in water, severely limiting its scope to simple, usually ionic nucleophiles. Although amino acids are insoluble in HFIP, the addition of base (NEt_3_) to the developed protocol proved to be beneficial, thus allowing uncharged, non‐polar, carbon nucleophiles to be employed. Along this line, we have used alanine for the synthesis of an analogue of a nonsteroidal anti‐inflammatory drugs (**42**).

This protocol gives access to 2‐arylcarboxylic acids in synthethically useful yields in one‐step, while the reported ibuprofen synthesis requires 3 steps and use of highly toxic hydrogen fluoride and carbon monoxide.^[33]^

An interesting trend can be observed for optically active substrates. Product **34** derived from (*R*)‐α‐methylbenzylamine was obtained as a racemic mixture, evidencing that the reaction proceeds via the assumed carbocation intermediate. When the methyl substituent was replaced with the carboxyl group, e.g. (*S*)‐phenylglycine was used as a starting material, acid **43** formed with 10% ee. For (*S*)‐alanine the ee increased to 81%. These results suggests the change in the reaction mechanism for amino acids, where the formation of chiral α‐lactone governs the enantioselectivity of the transformation, hence the retention of configuration can be observed.^[34]^ The low enantioselectivity for the reaction with (*S*)‐phenylglycine indicates that the competitive mechanism through the phenyl‐stabilized carbocation still dominates in contrast to (*S*)‐alanine.

Not limited to the examples mentioned above, the diazotization protocol also enables the synthesis of aromatic diazonium salts. They can be obtained in situ from anilines and reacted with the aromatic compound present in the system. For weakly nucleophilic arenes, the Gomberg‐Bachmann reaction takes place (**44**), while for phenol or aniline, diazo coupling occurs, leading to the corresponding dyes. In this way, methyl red **46**, a well‐known pH indicator, was synthesized in an excellent yield of 91%. It is worth noting that this reaction occurs in one step, immediately after the addition of the alkyl nitrite, and thus, utilizing two‐step procedures involving neutralization of nitrous acids in between, as in an aqueous environment, is not required.

**Scheme 4:**
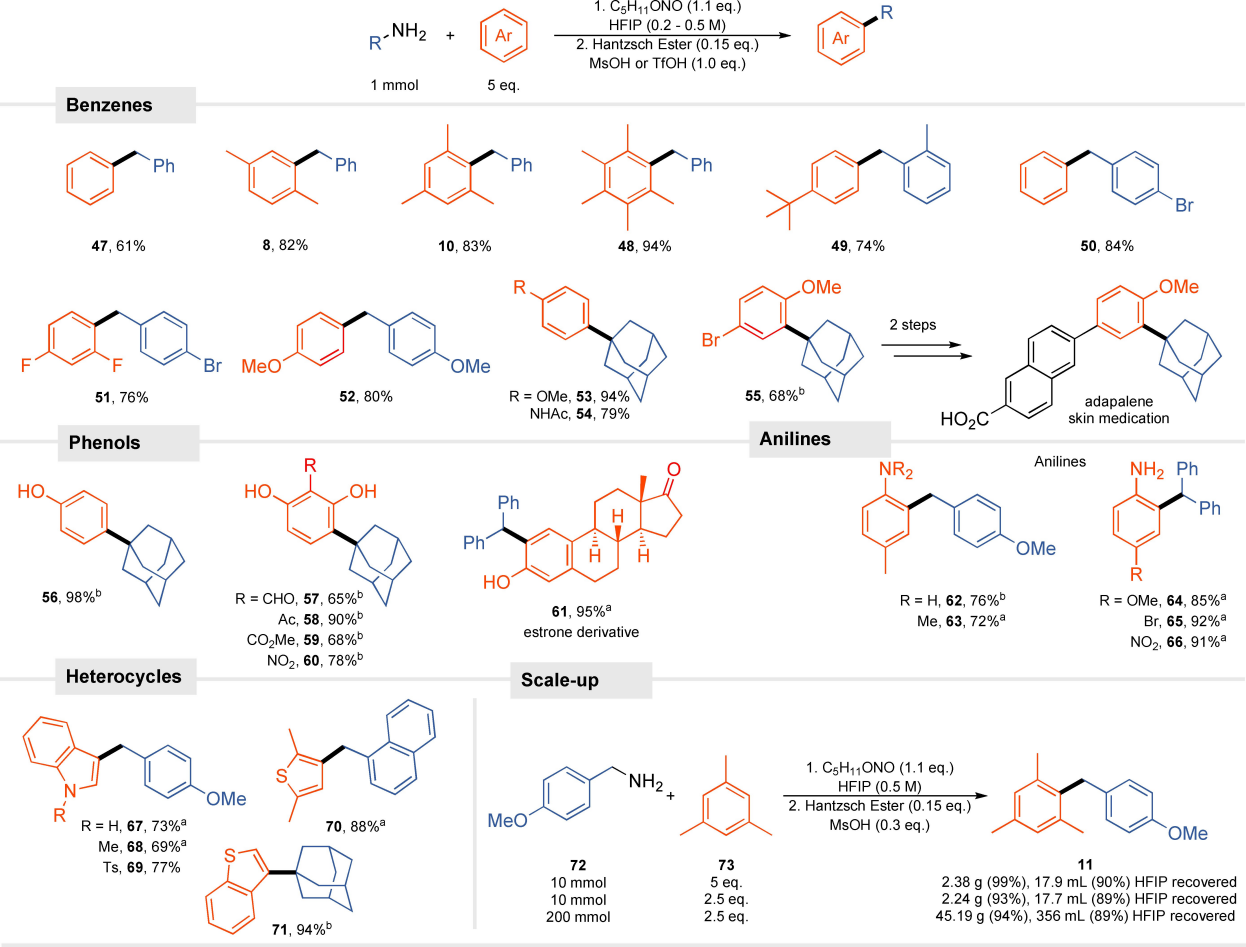
Scope and limitations functionalizations of arenes. ^a^Reaction conditions: 1) amine (1 mmol), AcOH (1.5 equiv.), C_5_H_11_ONO (1.1 equiv.), DCE (c = 0.5 M). 2) Hantzsch ester (0.15 equiv.), HFIP (4 mL), arene (5 equiv.), MsOH or TfOH (1 equiv.). ^b^Reaction conditions: 1) amine (1 mmol), C_5_H_11_ONO (1.1 equiv.), HFIP (c = 0.2 ‐ 0.5 M). 2) Hantzsch ester (0.15 equiv.), arene (5 equiv.), MsOH or TfOH (1 equiv.). ^c^Contains 8% *ortho* isomer.

Subsequently, the set of aromatic substrates was examined. Typically for Fiedel‐Crafts type reactions, the yield increased with the nucleophilicity of the arene (**47**, **8**, **10**, **48**), but even 1,3‐difluorobenzene was used efficiently benzylated (**51**) (Scheme  4). Alkylation of anisole, phenol, or acetanilide works well as represented by the synthesis of a substrate for the production of adapalene **55**. In electrophilic aromatic substitution, as the nucleophilicity of the arene decreases, the efficacy of the alkylation decreases. In our case, however, benzene or even deactivated 1,3‐difluorobenzene is sufficiently reactive to afford product **51**. The presence of two hydroxyl groups effectively neutralizes the deactivating effect of electron‐withdrawing groups, thus enabling exploration of a broader group of arenes. Along this line, the reaction tolerates all major EWGs, even the highly reactive aldehyde group (**57** ‐ **60**). Furthermore, the use of HFIP allows for efficient alkylation of anilines despite their basic nature and susceptibility to diazotization. Here, we took advantage of the fact that the arene can be added after the diazonization step. Moreover, even the presence of a strongly deactivating nitro group does not interrupt the reaction, providing excellent yields (**66**). In addition to benzene derivatives, heteroaromatic compounds are also successfully employed. Both *N*‐substituted and *N*‐H indole give products **67** ‐ **69** in good yields. Thiophene derivatives react excellently. Note that for most anilines and heteroaromatic compounds, diazotization employing acetic acid is more efficient.

To demonstrate the synthetic impact of our method, we chose the synthesis of compound **11** as a test reaction that was scaled by a factor of 10 and then 200. The experiment on a 10 mmol scale showed that reducing the amount of arene to 2.5 equiv. did not affect the efficacy of the reaction (99% versus 93%). From the 200 mmol scale, the desired product was isolated by crystallization (45.2 g, 94% yield,95% purity) with no erosion of selectivity or yield. HFIP was recovered by distillation (89%) and can thus be reused, which makes the protocol possible for technical applications. Despite the evolution of nitrogen, the reaction on such a scale was weakly exothermic.

**Scheme 5:**
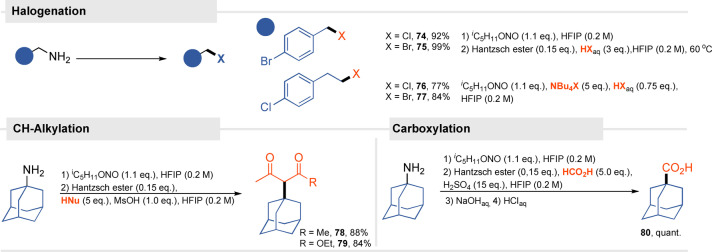
The developed methodology enable the transformation of amines into valuable building blocks–organohalides and carboxylic acids.

The developed methodology is not limited to alkylation, other nucleophiles engage in this transformation as well (Scheme 5). C‐nucleophiles can be used providing they possess a significant partial negative charge on the carbon atom. Derivatives of acetylacetone and ethyl acetoacetate **78**, **79** were obtained in high yields. Deaminative halogenations transform amines into classical aliphatic building blocks using the cheapest possible halogenating agents–aqueous hydrohalic acids without the need for modifying the conditions. Phenylethylamines give organic halides step in high yields already at diazotization, potential side reactions of oxidation of halide ions are negligible. Carboxylation under Koch reaction conditions was also successfully employed, obtaining adamantane carboxylic acid (**80**) in quantitative yield after hydrolysis of the hexafluoroisopropyl ester. The method therefore allows for the transformation of an amine functionality into a carboxylic acid in a simple way using very cheap substrates.

## Conclusions

We have demonstrated that diazotization of aliphatic amines, contrary to what is believed, can serve preparative purposes. The key to success in utilizing this transformation lies in the use of HFIP as a reaction medium that enables application of these reagents as precursors of carbocations. Here, these reactive intermediates are trapped by HFIP in a less reactive state thus enabling selective reactions to take place.

The diazotization process followed by subsequent acid‐catalyzed alkylation of aromatic compounds enables Friedel–Crafts‐type reactions in yields reaching quantitative. The described method stands out among deamination reactions because of its operational simplicity. It also allows for the generation of alkylated products involving highly destabilized carbocations, which are inaccessible from traditionally used alcohols or alkyl halides.

In addition to solving a long‐standing issue, the two‐step sequence procedure brings several additional benefits. Given the ready availability of the amine, from natural or commercial sources in wide structural varieties, and no need for prefunctionalization it also exhibits a very good atom economy compared to other deamination approaches. The only byproducts of the developed transformation are oxidized Hantzsch ester and innocuoussmall molecules of water and nitrogen (the alcohol, formed from alkyl nitrite, is used as a substrate for the synthesis of this reagent). No harmful or corrosive gases are produced, the purification process is straightforward and reaction is easily scalable.

We believe that our methodology lays ground for further exploration of other reactions involving aliphatic diazonium salts.

## Conflict of Interests

The authors declare no conflict of interest.

1

## Supporting information

As a service to our authors and readers, this journal provides supporting information supplied by the authors. Such materials are peer reviewed and may be re‐organized for online delivery, but are not copy‐edited or typeset. Technical support issues arising from supporting information (other than missing files) should be addressed to the authors.

Supporting Information

## Data Availability

The data that support the findings of this study are available in the supplementary material of this article.
