# Supplementary material for: Aliphatic Amines Unlocked for Selective Transformations through Diazotization
Source: Angew Chem Int Ed Engl. 2024 Dec 12;64(7):e202419450. doi: 10.1002/anie.202419450 (PMC11811684; doi:10.1002/anie.202419450)
Supplement: Supplementary file 1 — Supporting Information [file ANIE-64-e202419450-s001.pdf]

Angewandte Chemie
Eine Zeitschrift der Gesellschaft Deutscher Chemiker

Supporting Information

Aliphatic Amines Unlocked for Selective Transformations through Diazotization

J. Durka, B. Zielińska, D. Gryko**

Supporting Information

ALIPHATIC AMINES UNLOCKED FOR SELECTIVE TRANSFORMATION VIA DIAZOTIZATION

Jakub Durka,* Barbara Zielińska, and Dorota Gryko*

Institute of Organic Chemistry Polish Academy of Sciences, Kasprzaka 44/52, 01-224 Warsaw, Poland

Table of Contents

1. General information **3**

2. General procedures **4**

2.1 General procedure A for the alkylation of arenes with amines 4

2.2 General procedure B for the alkylation of arenes with amines 4

2.3 General procedure C for the alkylation of arenes with amines 5

2.4 General procedure D for the alkylation, arylation, or azo coupling of arenes with amines 5

3. Optimisation of reaction parameters **6**

3.1 Background experiments 6

3.2 Different reducing agents 6

3.3 Optimisation of the amount of nitrite 7

3.4 Optimisation of the amount of arene 7

3.5 Optimisation of the amount of Hantzsch ester 7

3.6 Optimisation of the amount of acid 8

3.7 Optimisation of the amount of HFIP 8

3.8 The influence of reaction time and temperature 8

3.9 Optimisation of the nitrite reagent 8

4. Scope and characterisation of products **10**

5. Mechanistic studies **46**

5.1 Kinetic studies 46

5.2 Influence of the acidity of alcoholic solvent 47

6. NMR spectra **48**

7. References **120**

1. General information

All solvents and commercially available reagents were purchased as reagent grade and used without further purification, unless otherwise stated. Reactions were performed without the exclusion of air or moisture. They were monitored by gas chromatography (GC, Shimadzu GCMS-QP2010 SE gas chromatograph with FID detector and Zebron ZB 5MSi column) and thin layer chromatography (TLC), using 0.20 mm Merck silica plates (60F-254) and visualized using UV-light, anisaldehyde or bromocresol green stain, with heat as a developing agent. Colum chromatography was performed on Merck silica gel 60 (230-400 mesh). GC yields were calibrated using dodecane as an internal standard.

NMR spectra were recorded on Bruker 400 MHz and calibrated using residual undeuterated solvent ($CHCl_3$ – 7.26 ppm ^{1}H NMR, 77.16 ppm ^{13}C NMR) or TMS as internal reference. Low-resolution mass spectra (LRMS) were recorded on an Applied Biosystems API 365 mass spectrometer using an electrospray ionisation (ESI) technique. High resolution mass spectra (HRMS) were recorded on a Waters AutoSpec Premier instrument using electron ionisation (EI) or a Waters SYNAPT G2-S HDMS instrument using electrospray ionization (ESI) with time of flight detector (TOF). Melting points were recorded on a Marienfeld MPM-H2 melting point apparatus and are uncorrected.

2. General procedures

2.1 General procedure A for the alkylation of arenes with amines

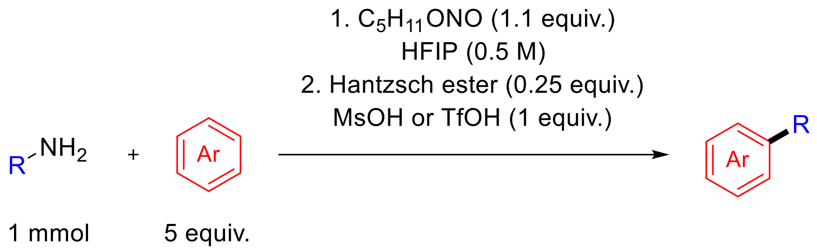

A 25 mL round-bottom flask equipped with a magnetic bar was charged with an amine (1.00 mmol, 1.00 equiv.), arene (5.00 mmol, 5.00 equiv.), HFIP (2.0 mL), and *iso*-pentyl nitrite (148 µL, 1.10 mmol, 1.10 equiv.). The reaction mixture was stirred at room temperature for 2 h. Then, Hantzsch ester (38 mg, 0.15 mmol, 0.15 equiv.), and MsOH or TfOH (65 µL or 88 µL, 1.0 mmol, 1.0 equiv.) were added. The reaction mixture was stirred at the indicated temperature for the indicated time. If the required temperature exceeded the boiling point of the solvent, the reaction mixture was transferred to a screw-cap vial. Upon completion, the reaction mixture was quenched with triethylammonium acetate (161 mg, 1.00 mmol) and concentrated *in vacuo*. A crude product was purified using column chromatography.

2.2 General procedure B for the alkylation of arenes with amines

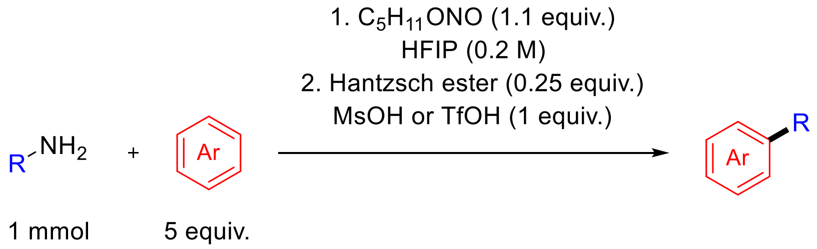

A 25 mL round-bottom flask equipped with a magnetic bar was charged with an amine (1.00 mmol, 1.00 equiv.), HFIP (5.0 mL), and *iso*-pentyl nitrite (148 µL, 1.10 mmol, 1.10 equiv.). The reaction mixture was stirred at room temperature for 2 h. Then, Hantzsch ester (38 mg, 0.15 mmol, 0.15 equiv.), arene (5.00 mmol, 5.00 equiv.) and MsOH or TfOH (65 µL or 88 µL, 1.0 mmol, 1.0 equiv.) were added. The reaction mixture was stirred at the indicated temperature for the indicated time. If the required temperature exceeded the boiling point of the solvent, the reaction mixture was transferred to a screw-cap vial. Upon completion, the reaction mixture

was quenched with triethylammonium acetate (161 mg, 1.00 mmol) and concentrated *in vacuo*. A crude product was purified using column chromatography.

2.3 General procedure C for the alkylation of arenes with amines

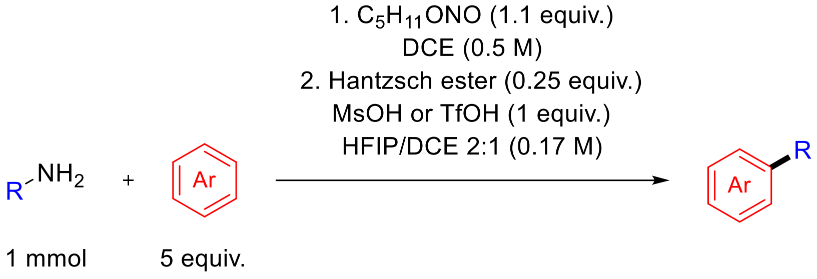

A 25 mL round-bottom flask equipped with a magnetic bar was charged with an amine (1.00 mmol, 1.00 equiv.), acetic acid (86 µL, 1.5 mmol, 1.5 equiv.), DCE (2.0 mL), and *iso*-pentyl nitrite (148 µL, 1.10 mmol, 1.10 equiv.). The reaction mixture was brought to reflux and stirred for 30 minutes. Then, after cooling, Hantzsch ester (38 mg, 0.15 mmol, 0.15 equiv.), HFIP (4.0 mL), arene (5.00 mmol, 5.00 equiv.) and MsOH or TfOH (65 µL or 88 µL, 1.0 mmol, 1.0 equiv.) were added. The reaction mixture was stirred at the indicated temperature for the indicated time. If the required temperature exceeded the boiling point of the solvent, the reaction mixture was transferred to a screw-cap vial. Upon completion, the reaction mixture was quenched with triethylammonium acetate (161 mg, 1.0 mmol) and concentrated *in vacuo*. A crude product was purified using column chromatography.

2.4 General procedure D for the alkylation, arylation, or azo coupling of arenes with amines

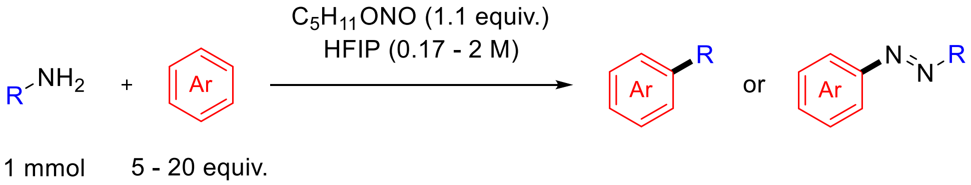

A 25 mL round-bottom flask equipped with a magnetic bar was charged with an amine (1.00 mmol, 1.00 equiv.), arene (5 – 20 equiv.), HFIP (0,5 – 6 mL), and *iso*-pentyl nitrite (148 µL, 1.10 mmol, 1.10 equiv.). The reaction mixture was stirred at room temperature for 16 h. Upon completion, the reaction mixture was quenched with triethylammonium acetate (161 mg, 1.0 mmol) and concentrated *in vacuo*. A crude product was purified using column chromatography.

3. Optimisation of reaction parameters

Model reaction

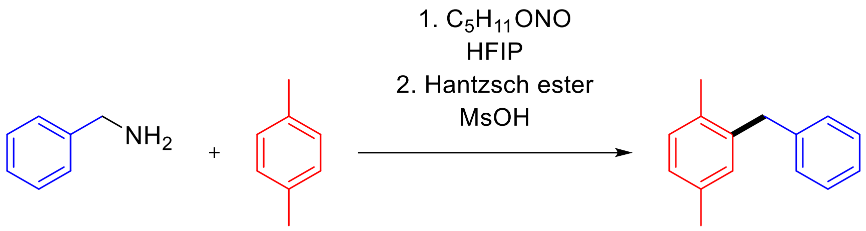

Reaction conditions: according to the general procedure A: 1) $BnNH_2$ (1.00 mmol), *p*-xylene (5.00 equiv.), $C_5H_{11}ONO$ (1.10 equiv.), HFIP (c = 0.5 M), 2 h, RT. 2) Hantzsch ester (0.15 equiv.), MsOH (1.0 equiv.), 3 h, reflux.

3.1 Background experiments

Entry	Deviation from the standerd conditions	Yield of X [%]
1	none	81
2	no MsOH	13
3	no Hantzsch ester	70
4	TFE instead of HFIP	0
5	*p*-xylene added to 2. step	77

Reaction conditions: 1) $BnNH_2$ (1.00 mmol), *p*-xylene (5.00 equiv.), $C_5H_{11}ONO$ (1.10 equiv.), HFIP (c = 0.5 M), 2 h, RT. 2) Hantzsch ester (0.15 equiv.), MsOH (1.0 equiv.), 3 h, reflux.

3.2 Different reducing agents

Entry	Reducing agent	Added to step	Yield of X [%]
1	none	-	70
2	urea	1	63
3	urea	2	69
4	hexanal	1	46
5	hexanal	2	75
6	$BuNH_2$	2	73
7	NH_4HCO_3	2	69
8	Hantzsch ester	1	58
9	Hantzsch ester	2	81

Reaction conditions: 1) $BnNH_2$ (1.00 mmol), *p*-xylene (5.00 equiv.), $C_5H_{11}ONO$ (1.10 equiv.), HFIP (c = 0.5 M), 2 h, RT. 2) reducing agent (0.15 equiv.), MsOH (1.0 equiv.), 3 h, reflux.

3.3 Optimisation of the amount of nitrite

Entry	$C_5H_{11}ONO$ (equiv.)	Yield of X [%]
1	1.00	74
2	1.05	78
3	1.10	81
4	1.15	81

Reaction conditions: 1) $BnNH_2$ (1.00 mmol), *p*-xylene (5.00 equiv.), $C_5H_{11}ONO$ (X equiv.), HFIP (c = 0.5 M), 2 h, RT. 2) Hantzsch ester (0.25 equiv.), MsOH (1.0 equiv.), 3 h, reflux.

3.4 Optimisation of the amount of arene

Entry	*p*-xylene (equiv.)	Yield of X [%]
1	3.00	71
2	5.00	81
3	10.00	92

Reaction conditions: 1) $BnNH_2$ (1.00 mmol), *p*-xylene (X equiv.), $C_5H_{11}ONO$ (1.10 equiv.), HFIP (c = 0.5 M), 2 h, RT. 2) Hantzsch ester (0.15 equiv.), MsOH (1.0 equiv.), 3 h, reflux. The use of 5 mmol was chosen as a compromise between the excess of arene and the efficiency.

3.5 Optimisation of the amount of Hantzsch ester

Entry	Hantzsch ester (equiv.)	Yield of X [%]
1	0.05	71
2	0.10	74
3	0.15	81
4	0.25	81

Reaction conditions: 1) $BnNH_2$ (1.00 mmol), *p*-xylene (5.00 equiv.), $C_5H_{11}ONO$ (1.10 equiv.), HFIP (c = 0.5 M), 2 h, RT. 2) Hantzsch ester (X equiv.), MsOH (1.0 equiv.), 3 h, reflux.

3.6 Optimisation of the amount of acid

Entry	MsOH (equiv.)	Yield of X [%]
1	0.25[a]	78
2	0.50	79
3	1.00	81

Reaction conditions: 1) $BnNH_2$ (1.00 mmol), *p*-xylene (5.00 equiv.), $C_5H_{11}ONO$ (1.10 equiv.), HFIP (c = 0.5 M), 2 h, RT. 2) Hantzsch ester (0.15 equiv.), MsOH (X equiv.), 3 h, reflux. [a]Reflux for 16h. Although the amount of acid used had no effect on the yield of the model reaction, 1 equiv. was used for scope evaluation due to the occasional acid-binding side reactions.

3.7 Optimisation of the amount of HFIP

Entry	c of $BnNH_2$ (M)	Yield of X [%]
1	2.0	70
2	1.0	81
3	0.5	81
4	0.33	81

Reaction conditions: 1) $BnNH_2$ (1.00 mmol), *p*-xylene (5.00 equiv.), $C_5H_{11}ONO$ (1.10 equiv.), HFIP (c = X M), 2 h, RT. 2) Hantzsch ester (0.15 equiv.), MsOH (1.0 equiv.), 3 h, reflux.

3.8 The influence of reaction time and temperature

Entry	Conditions of the 2. step	Yield of X [%]
1	24 h, room temperature	76
2	3 h, reflux	81
3	1.5 h, 140 °C	81

Reaction conditions: 1) $BnNH_2$ (1.00 mmol), *p*-xylene (5.00 equiv.), $C_5H_{11}ONO$ (1.10 equiv.), HFIP (c = 0.5 M), 2 h, RT. 2) Hantzsch ester (0.15 equiv.), MsOH (1.0 equiv.), X h, X °C.

3.9 Optimisation of the nitrite reagent

The choice of nitrite reagent was based on the experience gained while developing the project, not only by optimising the model reaction. We tested 5 different sources of nitrite:

Sodium nitrite $NaNO_2$ – while being the cheapest and easiest accesible, it is not soluble in HFIP and therefore was found innefective, even after the addition of phase transfer catalyst.

Tetrabutylammonium nitrite NBu_4NO_2 – although the reagent is commercially available it is expensive, hygroscopic, and very non-atom economical.

Tetramethylammonium nitrite NMe_4NO_2 – this reagent was desired to adress both solubility and atom economy issues. It was synthesised in a simple salt metathesis reaction between $NaNO_2$ and NMe_4Cl in 95% ethanol, after evaporation of the solvent. It is less hygroscopic than the tetrabutylammonium salt, has lower solubility in HFIP and, nevertheless, enables the reaction to proceed. The problem associated with the use of ionic nitrites under these conditions is the side reaction of nitration of aromatic rings, which is not detected in the case of nitrous acid esters.

***tert*-Butyl nitrite C_4H_9ONO** – is cheap, commercially available, and enables the diazotization reaction to proceed smoothly. However, in the second reaction step, stable tertiary carbocations are generated from the leftover *t*-butanol, leading to products of the undesired Friedel-Crafts reaction.

***iso*-Pentyl nitrite $C_5H_{11}ONO$** – is similarly cheap, available, and useful in the diazotization process as *tert*-butyl ester. It has a significant advantage over it in the second stage of the reaction because the products of undesirable alkylation are observed only at very high reaction temperatures. Moreover, due to the lack of steric hindrance, it more easily attacks substituted rings of substrates, e.g. mesitylene, and not the reaction product.

4. Scope and characterization of products

2-Benzylmesitylene (10)[1]

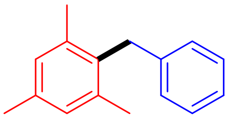

Following General Procedure A, compound **10** was obtained from benzylamine (107 mg, 1.00 mmol) and mesitylene (601 mg, 5.00 mmol). The reaction mixture was refluxed with MsOH for 3 h. Purified by column chromatography (petroleum ether) to afford 174 mg of compound **10** as colorless oil. **(Yield = 83%).**

^{1}H NMR (400 MHz, $CDCl_3$) δ 7.36 – 7.26 (m, 2H), 7.25 – 7.16 (m, 1H), 7.13 – 7.04 (m, 2H), 6.96 (s, 2H), 4.09 (s, 2H), 2.37 (s, 3H), 2.28 (s, 6H).

^{13}C NMR (100 MHz, $CDCl_3$) δ 140.2, 137.1, 135.7, 133.9, 129.0, 128.4, 127.9, 125.7, 34.8, 21.0, 20.2.

2-(4-Methoxybenzyl)mesitylene (11)[2]

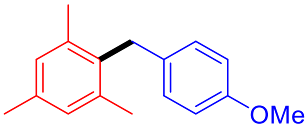

Following General Procedure A, compound **11** was obtained from 4-methoxybenzylamine (137 mg, 1.00 mmol) and mesitylene (601 mg, 5.00 mmol). The reaction mixture was stirred with MsOH at room temperature for 16 h. Purified by column chromatography (AcOEt/hexanes gradually from 0:100 to 3:97) to afford 239 mg of compound **11** as a white solid. **(Yield = 99%).**

^{1}H NMR (400 MHz, $CDCl_3$) δ 6.93 (d, J = 8.7 Hz, 2H), 6.89 (s, 2H), 6.80 – 6.76 (m, 2H), 3.95 (s, 2H), 3.77 (s, 3H), 2.28 (s, 3H), 2.21 (s, 6H).

^{13}C NMR (100 MHz, $CDCl_3$) δ 157.7, 136.9, 135.5, 134.2, 132.1, 128.9, 128.7, 113.8, 55.2, 33.8, 20.9, 20.1.

2-(4-Methylbenzyl)mesitylene (12)[1]

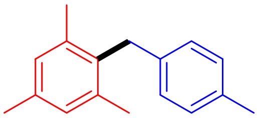

Following General Procedure A, compound **12** was obtained from 4-methylbenzylamine (121 mg, 1.00 mmol) and mesitylene (601 mg, 5.00 mmol). The reaction mixture was refluxed with MsOH for 3 h. Purified by column chromatography (petroleum ether) to afford 200 mg of compound **12** as colorless oil. **(Yield = 89%).**

^{1}H NMR (400 MHz, $CDCl_3$) δ 7.10 (d, *J* = 7.8 Hz, 2H), 7.02 – 6.89 (m, 4H), 4.04 (s, 2H), 2.36 (s, 6H), 2.27 (s, 6H).

^{13}C NMR (100 MHz, $CDCl_3$) δ 137.0, 137.0, 135.6, 135.1, 134.0, 129.1, 128.9, 127.8, 34.3, 21.0, 20.9, 20.2.

2-(4-*tert*-Butylbenzyl)mesitylene (13)[1]

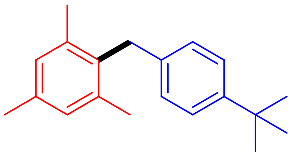

Following General Procedure A, compound **13** was obtained from 4-*tert*-butylbenzylamine (163 mg, 1.00 mmol) and mesitylene (601 mg, 5.00 mmol). The reaction mixture was refluxed with MsOH for 3 h. Purified by column chromatography (petroleum ether) to afford 258 mg of compound **13** as a white solid. **(Yield = 97%).**

^{1}H NMR (400 MHz, $CDCl_3$) δ 7.25 – 7.22 (m, 2H), 6.95 (d, *J* = 8.6 Hz, 2H), 6.88 (s, 2H), 3.98 (s, 2H), 2.29 (s, 3H), 2.22 (s, 6H), 1.28 (s, 9H).

^{13}C NMR (100 MHz, $CDCl_3$) δ 148.4, 137.0, 137.0, 135.5, 134.2, 128.9, 127.6, 125.2, 34.3, 34.3, 31.5, 20.9, 20.2.

2-(4-Fluorobenzyl)mesitylene (14)[1]

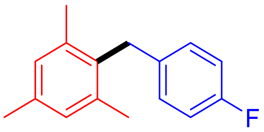

Following General Procedure A, compound **14** was obtained from 4-fluorobenzylamine (125 mg, 1.00 mmol) and mesitylene (601 mg, 5.00 mmol). The reaction mixture was refluxed with

MsOH for 4 h. Purified by column chromatography (petroleum ether) to afford 222 mg of compound **14** as colorless oil. **(Yield = 97%).**

^{1}H NMR (400 MHz, CDCl$_3$) δ 7.01 – 6.89 (m, 6H), 3.99 (s, 2H), 2.31 (s, 3H), 2.21 (s, 6H).

^{13}C NMR (100 MHz, CDCl$_3$) δ 161.2 (d, J = 243.3 Hz), 136.9, 135.8, 135.7 (d, J = 3.2 Hz), 133.6, 129.1 (d, J = 7.7 Hz), 129.0, 115.1 (d, J = 21.1 Hz), 33.9, 20.9, 20.0.

2-(4-Bromobenzyl)mesitylene (15)[1]

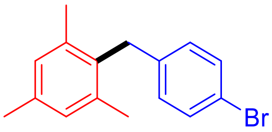

Following General Procedure A, compound **15** was obtained from 4-bromobenzylamine (223 mg, 1.00 mmol) and mesitylene (601 mg, 5.00 mmol). The reaction mixture was refluxed with MsOH for 4 h. Purified by column chromatography (petroleum ether) to afford 287 mg of compound **15** as a white solid. **(Yield = 99%).**

^{1}H NMR (400 MHz, CDCl$_3$) δ 7.43 – 7.33 (m, 2H), 6.97 – 6.89 (m, 4H), 4.00 (s, 2H), 2.33 (s, 3H), 2.23 (s, 6H).

^{13}C NMR (100 MHz, CDCl$_3$) δ 139.2, 136.9, 136.0, 133.2, 131.4, 129.6, 129.1, 119.5, 34.2, 20.9, 20.1.

2-(4-Trifluoromethylbenzyl)mesitylene (16)[1]

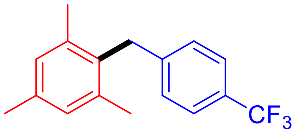

Following General Procedure A, compound **16** was obtained from 4-trifluoromethylbenzylamine (175 mg, 1.00 mmol) and mesitylene (601 mg, 5.00 mmol). The reaction mixture was refluxed with TfOH for 48 h. Purified by column chromatography (petroleum ether) to afford 177 mg of compound **16** as colorless oil. **(Yield = 64%)**

Following General Procedure C, compound **16** was obtained from 4-trifluoromethylbenzylamine (175 mg, 1.00 mmol) and mesitylene (601 mg, 5.00 mmol). The reaction mixture was stirred with TfOH at 100 °C for 16 h. Purified by column chromatography (petroleum ether) to afford 240 mg of compound **16** as colorless oil. **(Yield = 86%).**

¹H NMR (400 MHz, CDCl₃) δ 7.48 (d, *J* = 8.0 Hz, 2H), 7.12 (d, *J* = 8.6 Hz, 2H), 6.90 (s, 2H), 4.06 (s, 2H), 2.30 (s, 3H), 2.18 (s, 6H).

¹³C NMR (100 MHz, CDCl₃) δ 144.5, 136.9, 136.6, 132.8, 129.1, 128.3, 128.1, 125.3 (q, *J*= 3.7 Hz), 123.0, 34.6, 20.9, 20.1.

2-(4-Nitrobenzyl)mesitylene (17)[3]

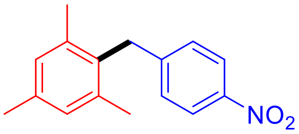

Following General Procedure C, compound **17** was obtained from 4-nitrobenzylamine (152 mg, 1.00 mmol) and mesitylene (601 mg, 5.00 mmol). The reaction mixture was stirred with TfOH at 100 °C for 16 h. Purified by column chromatography (0:100 to 3:97 AcOEt/hexanes) to afford 174 mg of compound **17** as a yellowish solid. **(Yield = 68%).**

¹H NMR (400 MHz, CDCl₃) δ 8.15 – 8.05 (m, 2H), 7.17 (d, *J* = 8.8 Hz, 2H), 6.91 (s, 2H), 4.11 (s, 2H), 2.30 (s, 3H), 2.19 (s, 6H).

¹³C NMR (100 MHz, CDCl₃) δ 148.3, 146.4, 136.8, 136.5, 132.1, 129.2, 128.6, 123.7, 34.8, 20.9, 20.1.

2-(2-Methylbenzyl)mesitylene (18)[1]

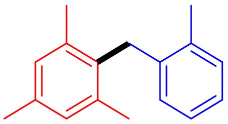

Following General Procedure A, compound **18** was obtained from 2-methylbenzylamine (121 mg, 1.00 mmol) and mesitylene (601 mg, 5.00 mmol). The reaction mixture was refluxed with MsOH for 3 h. Purified by column chromatography (petroleum ether) to afford 202 mg of compound **18** as a white solid. **(Yield = 90%).**

¹H NMR (400 MHz, CDCl₃) δ 7.28 – 7.22 (m, 1H), 7.15 (t, *J* = 7.3 Hz, 1H), 7.05 (t, *J* = 7.5 Hz, 1H), 6.98 (s, 2H), 6.59 (d, *J* = 7.6 Hz, 1H), 3.95 (s, 2H), 2.49 (s, 3H), 2.38 (s, 3H), 2.22 (s, 6H).

¹³C NMR (100 MHz, CDCl₃) δ 138.0, 137.2, 136.2, 135.6, 133.6, 129.7, 128.9, 126.3, 126.1, 125.8, 32.1, 21.0, 19.9, 19.8.

2-(Naphthalen-1-ylmethyl)mesitylene (19)[2]

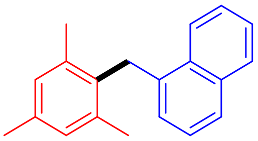

Following General Procedure A, compound **19** was obtained from 1-naphthylmethylamine (157 mg, 1.00 mmol) and mesitylene (601 mg, 5.00 mmol). The reaction mixture was stirred with MsOH at room temperature for 16 h. Purified by column chromatography (petroleum ether) to afford 237 mg of compound **19** as a white solid. **(Yield = 91%).**

^{1}H NMR (400 MHz, $CDCl_3$) δ 8.33 (d, *J* = 8.5 Hz, 1H), 8.00 – 7.93 (m, 1H), 7.77 (d, *J* = 8.2 Hz, 1H), 7.71 – 7.64 (m, 1H), 7.64 – 7.57 (m, 1H), 7.36 – 7.29 (m, 1H), 7.04 (s, 2H), 6.77 (dd, *J* = 7.1, 1.1 Hz, 1H), 4.49 (s, 2H), 2.43 (s, 3H), 2.24 (s, 6H)

^{13}C NMR (100 MHz, $CDCl_3$) δ 137.5, 135.9, 135.4, 133.8, 133.2, 132.4, 129.0, 128.9, 126.6, 126.0, 125.8, 125.6, 123.6, 123.2, 31.5, 21.1, 19.9.

2-Benzhydrylmesitylene (20)[2]

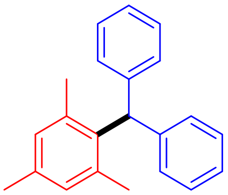

Following General Procedure A, compound **20** was obtained from benzhydrylamine (183 mg, 1.00 mmol) and mesitylene (601 mg, 5.00 mmol). The reaction mixture was stirred with MsOH at room temperature for 16 h. Purified by column chromatography (petroleum ether) to afford 252 mg of compound **20** as a white solid. **(Yield = 88%).**

^{1}H NMR (400 MHz, $CDCl_3$) δ 7.34 – 7.29 (m, 4H), 7.27 – 7.22 (m, 2H), 7.19 – 7.14 (m, 4H), 6.92 (s, 2H), 6.07 (s, 1H), 2.33 (s, 3H), 2.06 (s, 6H).

^{13}C NMR (100 MHz, $CDCl_3$) δ 142.6, 137.6, 137.2, 136.0, 130.2, 129.4, 128.2, 126.0, 51.1, 22.0, 20.9.

2-(3,4-Dimethoxybenzyl)mesitylene (21)[2]

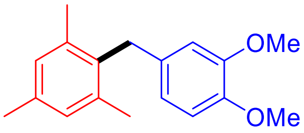

Following General Procedure A, compound **21** was obtained from 3,4-dimethoxybenzylamine (167 mg, 1.00 mmol) and mesitylene (601 mg, 5.00 mmol). The reaction mixture was stirred with MsOH at room temperature for 16 h. Purified by column chromatography (AcOEt/hexanes gradually from 0:100 to 3:97) to afford 241 mg of compound **21** as a yellowish solid. **(Yield = 89%).**

^{1}H NMR (400 MHz, $CDCl_3$) δ 6.90 (s, 2H), 6.73 (d, *J* = 8.2 Hz, 1H), 6.64 (d, *J* = 1.3 Hz, 1H), 6.52 – 6.45 (m, 1H), 3.98 (s, 2H), 3.83 (s, 3H), 3.82 (s, 3H), 2.31 (s, 3H), 2.24 (s, 6H).

^{13}C NMR (100 MHz, $CDCl_3$) δ 149.0, 147.2, 137.0, 135.6, 133.9, 132.7, 128.9, 119.5, 111.6, 111.3, 55.9, 55.8, 34.2, 20.9, 20.1.

1,3-Bis(2,4,6-trimethylbenzyl)benzene (22)[4]

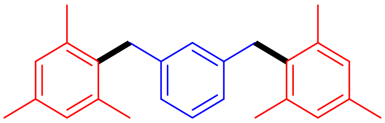

Following General Procedure A, compound **22** was obtained from 1,3-bis(aminomethyl)-benzene (136 mg, 1.00 mmol) and mesitylene (1202 mg, 10.0 mmol). The reaction mixture was refluxed with MsOH for 3 h. Purified by column chromatography (petroleum ether) to afford 323 mg of compound **22** as a white solid. **(Yield = 94%).**

^{1}H NMR (400 MHz, $CDCl_3$) 7.11 – 7.03 (m, 1H), 6.88 (s, 4H), 6.78 – 6.73 (m, 3H), 3.95 (s, 4H), 2.31 (s, 6H), 2.18 (s, 12H).

^{13}C NMR (100 MHz, $CDCl_3$) δ 140.1, 136.9, 135.5, 133.9, 128.8, 128.3, 127.9, 125.0, 34.7, 20.9, 20.1.

2-Piperonylmesitylene (23)

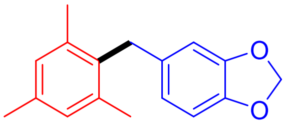

Following General Procedure A, compound **23** was obtained from piperonylamine (151 mg, 1.00 mmol) and mesitylene (601 mg, 5.00 mmol). The reaction mixture was stirred with MsOH

at room temperature for 16 h. Purified by column chromatography (AcOEt/hexanes gradually from 0:100 to 3:97) to afford 200 mg of compound **23** as yellowish oil. **(Yield = 78%)**

^{1}H NMR (400 MHz, $CDCl_3$) δ 6.92 (s, 2H), 6.72 (d, *J* = 7.9 Hz, 1H), 6.54 (s, 1H), 6.51 (d, *J* = 8.0 Hz, 1H), 5.91 (s, 2H), 3.97 (s, 2H), 2.33 (s, 3H), 2.25 (s, 6H).

^{13}C NMR (100 MHz, $CDCl_3$) δ 147.8, 145.6, 137.0, 135.7, 134.0, 134.0, 129.0, 120.6, 108.4, 108.1, 100.8, 34.3, 20.9, 20.1.

HRMS (m/z) (EI) [M] calculated for $C_{17}H_{18}O_2$: 254.1307, found: 254.1305.

1,4-Dimethyl-2-(2,4-difluorobenzyl)benzene (24)[5]

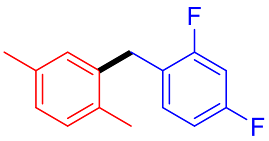

Following General Procedure A, compound **24** was obtained from 2,4-difluorobenzylamine (143 mg, 1.00 mmol) and *p*-xylene (531 mg, 5.00 mmol).The reaction mixture was refluxed with TfOH for 16 h. Purified by column chromatography (petroleum ether) to afford 224 mg of compound **24** as colorless oil. **(Yield = 96%).**

^{1}H NMR (400 MHz, $CDCl_3$) δ 7.10 (d, *J* = 7.6 Hz, 1H), 7.01 (d, *J* = 7.6 Hz, 1H), 6.97 – 6.88 (m, 2H), 6.87 – 6.81 (m, 1H), 6.81 – 6.74 (m, 1H), 3.93 (s, 2H), 2.32 (s, 3H), 2.24 (s, 3H).

^{13}C NMR (100 MHz, $CDCl_3$) δ 162.4 (dd, *J* = 74.3, 12.1 Hz), 159.9 (dd, *J* = 75.2, 12.1 Hz), 137.0, 135.6, 133.4, 131.0 (dd, *J* = 9.4, 6.2 Hz), 130.5, 130.3, 127.4, 123.3 (dd, *J* = 16.1, 3.8 Hz), 111.0 (dd, *J* = 20.8, 3.8 Hz), 103.5 (t, *J* = 25.3 Hz), 31.4, 31.4, 20.9, 18.9.

1,4-Dimethyl-2-(3-trifluoromethylbenzyl)benzene (25)[5]

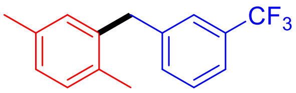

Following General Procedure A, compound **25** was obtained from 3-trifluoromethylbenzylamine (175 mg, 1.00 mmol) and *p*-xylene (531 mg, 5.00 mmol). The reaction mixture was refluxed with TfOH for 16 h. Purified by column chromatography (petroleum ether) to afford 252 mg of compound **25** as colorless oil. **(Yield = 95%).**

^{1}H NMR (400 MHz, $CDCl_3$) δ 7.48 (d, *J* = 7.8 Hz, 1H), 7.44 (s, 1H), 7.39 (t, *J* = 7.7 Hz, 1H), 7.30 (d, *J* = 7.7 Hz, 1H), 7.10 (d, *J* = 7.6 Hz, 1H), 7.02 (d, *J* = 7.7 Hz, 1H), 6.95 (s, 1H), 4.03 (s, 2H), 2.33 (s, 3H), 2.21 (s, 3H).

^{13}C NMR (100 MHz, $CDCl_3$) δ 141.6, 137.6, 135.7, 133.4, 132.0, 130.9, 130.8, 130.6, 130.4, 128.8, 127.6, 124.1 (dq, *J* = 256.0, 3.8 Hz), 39.3, 21.0, 19.2.

1,4-Dimethyl-2-(3,5-bis(trifluoromethyl)benzyl)benzene (26)[6]

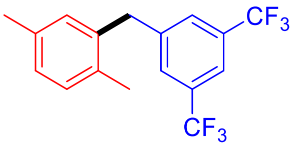

Following General Procedure A, compound **26** was obtained from 3,5-bis(trifluoromethyl)benzylamine (243 mg, 1.00 mmol) and *p*-xylene (531 mg, 5.00 mmol). The reaction mixture was stirred with TfOH at 140 °C for 40 h. Purified by column chromatography (petroleum ether) to afford 277 mg of compound **26** as colorless oil. **(Yield = 83%).**

^{1}H NMR (400 MHz, $CDCl_3$) δ 7.71 (s, 1H), 7.56 (s, 2H), 7.09 (d, *J* = 7.8 Hz, 1H), 7.02 (d, *J* = 7.8 Hz, 1H), 6.90 (s, 1H), 4.06 (s, 2H), 2.31 (s, 3H), 2.17 (s, 3H).

^{13}C NMR (100 MHz, $CDCl_3$) δ 143.2, 136.4, 136.0, 133.3, 131.7 (q, *J* = 33.0 Hz), 130.7, 130.7, 128.8 – 128.6 (m), 128.0, 123.4 (q, *J* = 272.6 Hz), 120.1 (sep, *J* = 3.9 Hz), 39.1, 20.9, 19.1.

2-Dodecylmesitylene (27)[7]

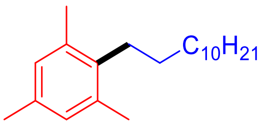

Following a slightly modified General Procedure C, compound **27** was obtained from dodecylamine (185 mg, 1.00 mmol) and mesitylene (601 mg, 5.00 mmol). DCE was carefully evaporated after diazotization so that the second step could proceed in pure HFIP. The reaction mixture was stirred with TfOH at 70 °C for 30 min and then at 140 °C for 16 h. Purified by column chromatography (petroleum ether) to afford 92 mg of compound **27** as colorless oil. **(Yield = 32%).**

^{1}H NMR (400 MHz, $CDCl_3$) δ 6.83 (s, 2H), 2.61 – 2.52 (m, 2H), 2.29 (s, 6H), 2.25 (s, 3H), 1.48 – 1.39 (m, 4H), 1.32 – 1.25 (m, 16H), 0.93 – 0.87 (m, 4H).

^{13}C NMR (100 MHz, $CDCl_3$) δ 136.7, 135.8, 134.7, 128.8, 31.9, 30.3, 29.7, 29.7, 29.7, 29.5, 29.5, 29.4, 22.7, 20.8, 19.7, 14.1.

2-Hexadecylmesitylene (28)[7]

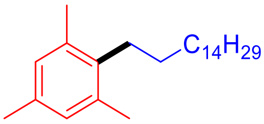

Following a slightly modified General Procedure C, compound **28** was obtained from hexadecylamine (241 mg, 1.00 mmol) and mesitylene (601 mg, 5.00 mmol). DCE was carefully evaporated after diazotization so that the second step could proceed in pure HFIP. The reaction mixture was stirred with TfOH at 70 °C for 30 min and then at 140 °C for 16 h. Purified by column chromatography (petroleum ether) to afford 107 mg of compound **28** as colorless oil. **(Yield = 31%).**

^{1}H NMR (400 MHz, $CDCl_3$) δ 6.82 (s, 2H), 2.59 – 2.52 (m, 2H), 2.28 (s, 6H), 2.24 (s, 3H), 1.46 – 1.39 (m, 4H), 1.26 (s, 24H), 0.88 (t, *J* = 6.8 Hz, 3H).

^{13}C NMR (100 MHz, $CDCl_3$) δ 136.7, 135.8, 134.7, 128.8, 31.9, 30.3, 29.7, 29.7, 29.7, 29.5, 29.4, 29.3, 29.3, 22.7, 20.8, 19.7, 14.1.

2-(4-Methoxyphenethyl)mesitylene (29)[7]

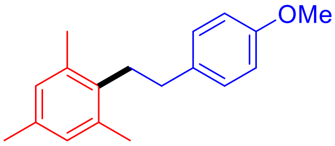

Following General Procedure A, compound **29** was obtained from 4-methoxyphenethylamine (151 mg, 1.00 mmol) and mesitylene (601 mg, 5.00 mmol). The reaction mixture was refluxed with TfOH for 16 h. Purified by column chromatography (AcOEt/hexanes gradually from 0:100 to 3:97) to afford 162 mg of compound **29** as yellowish oil. **(Yield = 64%).**

^{1}H NMR (400 MHz, $CDCl_3$) δ 7.21 (d, *J* = 8.5 Hz, 2H), 6.95 – 6.89 (m, 4H), 3.86 (s, 3H), 2.92 (dd, *J* = 10.5, 6.3 Hz, 2H), 2.75 (dd, *J* = 10.4, 6.3 Hz, 2H), 2.37 (s, 6H), 2.33 (s, 3H).

^{13}C NMR (100 MHz, $CDCl_3$) δ 158.0, 136.0, 135.6, 135.2, 134.5, 129.2, 129.0, 113.9, 55.3, 34.7, 32.0, 20.9, 19.7.

2-(4-Chlorophenethyl)mesitylene (30)[7]

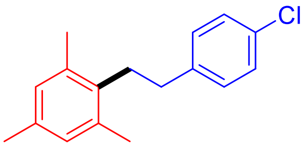

Following General Procedure A, compound **30** was obtained from 4-chlorophenethylamine (156 mg, 1.00 mmol) and mesitylene (601 mg, 5.00 mmol). The reaction mixture was refluxed with TfOH for 16 h. Purified by column chromatography (petroleum ether) to afford 169 mg of compound **30** as colorless oil. **(Yield = 65%).**

^{1}H NMR (400 MHz, $CDCl_3$) δ 7.34 – 7.28 (m, 2H), 7.21 – 7.15 (m, 2H), 6.90 (s, 2H), 2.90 (dd, J = 10.8, 5.8 Hz, 2H), 2.76 (dd, J = 10.7, 5.8 Hz, 2H), 2.33 (s, 6H), 2.32 (s, 3H).

^{13}C NMR (100 MHz, $CDCl_3$) δ 140.7, 136.0, 135.3, 135.0, 131.7, 129.7, 129.1, 128.5, 34.9, 31.6, 20.9, 19.7.

2-(4-Trifluoromethylphenethyl)mesitylene (31)[8]

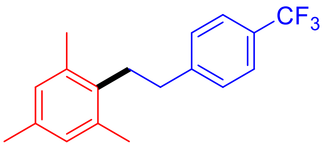

Following General Procedure A, compound **31** was obtained from 4-trifluoromethylphenethylamine (189 mg, 1.00 mmol) and mesitylene (601 mg, 5.00 mmol). The reaction mixture was refluxed with TfOH for 16 h. Purified by column chromatography (petroleum ether) to afford 97 mg of compound **31** as a white solid. **(Yield = 33%).**

^{1}H NMR (400 MHz, $CDCl_3$) δ 7.57 (d, J = 8.0 Hz, 2H), 7.33 (d, J = 8.0 Hz, 2H), 6.87 (s, 2H), 2.94 – 2.86 (m, 2H), 2.84 – 2.77 (m, 2H), 2.31 (s, 6H), 2.28 (s, 3H).

^{13}C NMR (100 MHz, $CDCl_3$) δ 146.3, 146.3, 136.0, 135.5, 134.8, 129.1, 128.6, 128.6, 128.3, 127.9, 125.8, 125.4, 125.4, 125.3, 125.3, 123.1, 35.4, 31.4, 20.8, 19.6.

2-Cyclohexylmesitylene (32)[9]

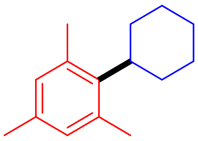

Following General Procedure A, compound **32** was obtained from cyclohexylamine (99 mg, 1.00 mmol) and mesitylene (601 mg, 5.00 mmol). The reaction mixture was refluxed with TfOH for 16 h. Purified by column chromatography (petroleum ether) to afford 178 mg of compound **32** as colorless oil. **(Yield = 88%).**

^{1}H NMR (400 MHz, $CDCl_3$) δ 7.01 (s, 1H), 6.94 (s, 1H), 6.87 – 6.81 (m, 2H), 3.05 – 2.94 (m, 1H), 2.73 – 2.63 (m, 1H), 2.51 – 2.32 (m, 6H), 2.30 – 2.28 (m, 3H), 2.26 (s, 6H), 2.23 – 2.21 (m, 3H), 2.00 – 1.75 (m, 10H), 1.50 – 1.24 (m, 10H).

^{13}C NMR (100 MHz, $CDCl_3$) δ 143.3, 140.2, 136.1, 134.7, 133.9, 133.4, 132.3, 131.7, 128.2, 126.8, 123.9, 41.4, 39.8, 33.9, 33.9, 30.6, 27.8, 27.3, 27.3, 26.4, 26.4, 20.6, 19.4, 19.1, 18.7.

2-Cyclododecylmesitylene (33)[7]

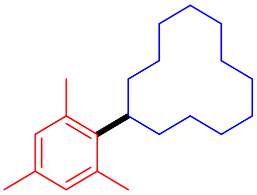

Following General Procedure A, compound **33** was obtained from cyclododecylamine (183 mg, 1.00 mmol) and mesitylene (601 mg, 5.00 mmol). The reaction mixture was refluxed with TfOH for 16 h. Purified by column chromatography (petroleum ether) to afford 277 mg of compound **33** as a yellowish solid. **(Yield = 97%).**

^{1}H NMR (400 MHz, $CDCl_3$) δ 6.84 (s, 2H), 3.23 (p, J = 6.7 Hz, 1H), 2.42 (s, 3H), 2.38 (s, 3H), 2.27 (s, 3H), 2.04 – 1.93 (m, 2H), 1.75 – 1.27 (m, 20H).

^{13}C NMR (100 MHz, $CDCl_3$) δ 140.6, 136.7, 136.4, 134.6, 131.3, 128.9, 34.1, 29.8, 24.8, 24.6, 24.5, 23.2, 22.3, 21.9, 21.7, 20.6.

2-(1-Phenylethyl)mesitylene (34)[2]

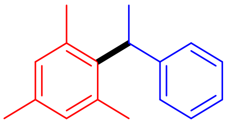

Following General Procedure A, compound **34** was obtained from (*R*)-(+)-α-methylbenzylamine (121 mg, 1.00 mmol) and mesitylene (601 mg, 5.00 mmol). The reaction mixture was refluxed with MsOH for 3 h. Purified by column chromatography (petroleum ether) to afford 129 mg of compound **34** as colorless oil. **(Yield = 58%)**

Following General Procedure A, compound **34** was obtained from (*R*)-(+)-α-methylbenzylamine (121 mg, 1.00 mmol) and mesitylene (601 mg, 5.00 mmol). The reaction mixture was refluxed with NBu_4Br (322 mg, 1.00 mmol, added before the acid) and MsOH for 3 h. Purified by column chromatography (petroleum ether) to afford 207 mg of compound **34** as colorless oil. **(Yield = 92%).**

$[\alpha]D_{20}$ = 0.00 (c = 0.67 in $CHCl_3$)

^{1}H NMR (400 MHz, $CDCl_3$) δ 7.34 – 7.28 (m, 2H), 7.25 – 7.18 (m, 3H), 6.88 (s, 2H), 4.70 (q, J = 7.3 Hz, 1H), 2.32 (s, 3H), 2.20 (s, 6H), 1.72 (d, J = 7.3 Hz, 3H).

^{13}C NMR (100 MHz, $CDCl_3$) δ 145.5, 140.1, 136.5, 135.4, 130.0, 128.2, 126.9, 125.3, 37.9, 21.1, 20.8, 16.9.

2-(1-(4-Nitrophenyl)ethyl)mesitylene (35)[10]

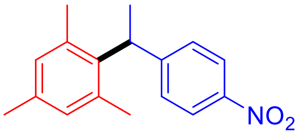

Following General Procedure C, compound **35** was obtained from 1-(4-nitrophenyl)ethylamine (166 mg, 1.00 mmol) and mesitylene (601 mg, 5.00 mmol). The reaction mixture was stirred with TfOH at 100 °C for 16 h. Purified by column chromatography (AcOEt/hexanes gradually from 0:100 to 3:97) to afford 254 mg of compound **35** as a yellowish solid. **(Yield = 94%).**

^{1}H NMR (400 MHz, $CDCl_3$) δ 8.21 – 8.05 (m, 2H), 7.41 – 7.28 (m, 2H), 6.86 (s, 2H), 4.67 (q, J = 7.2 Hz, 1H), 2.28 (s, 3H), 2.10 (s, 6H), 1.72 (d, J = 7.2 Hz, 3H).

^{13}C NMR (100 MHz, $CDCl_3$) δ 153.8, 145.9, 138.6, 136.2, 136.2, 130.3, 127.6, 123.4, 38.3, 21.0, 20.7, 16.9.

2-(4-Heptyl)mesitylene (36A), 2-(3-heptyl)mesitylene (36B)

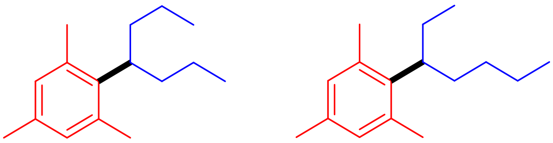

Following General Procedure A, compounds **36A** and **36B** was obtained from 4-heptylamine (115 mg, 1.00 mmol) and mesitylene (601 mg, 5.00 mmol). The reaction mixture was refluxed with TfOH for 16 h. Purified by column chromatography (petroleum ether) to afford 173 mg of compounds **36A** and **36B** 3:2 as colorless oil. **(Yield = 79%)**

HRMS (m/z) (EI) [M] calculated for $C_{16}H_{26}$: 218.2035, found: 218.2031.

GC Chromatogram: purity: 98%, peak ratio: 2:3

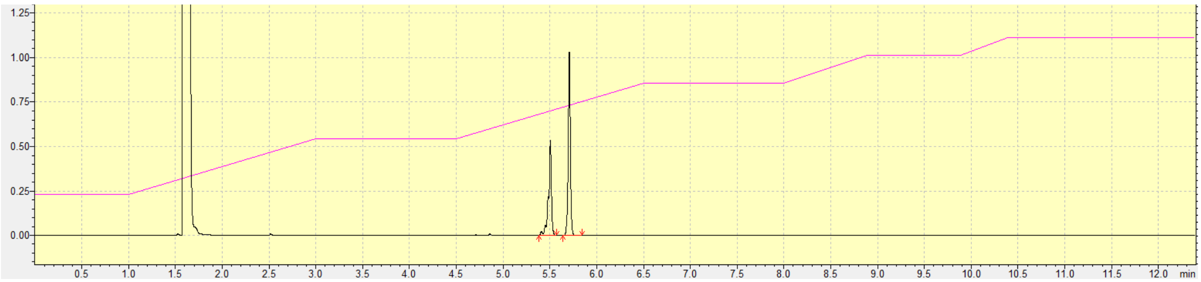

3-Mesityl-1-phenylbutane (37A)[7], 2-mesityl-1-phenylbutane (37B)

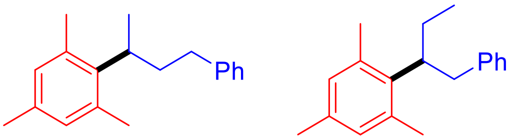

Following General Procedure A, compounds **37A** and **37B** was obtained from 4-phenyl-2-butylamine (149 mg, 1.00 mmol) and mesitylene (601 mg, 5.00 mmol). The reaction mixture was refluxed with TfOH for 16 h. Purified by column chromatography (petroleum ether) to afford 156 mg of compounds **37A** and **37B** 5:3 as colorless oil. **(Yield = 62%).**

^{1}H NMR (400 MHz, CDCl$_3$) δ 7.34 – 7.09 (m, 8H), 6.82 (m, 3H), 3.36 – 3.20 (m, 1.6H), 3.11 – 2.95 (m, 1.2H), 2.70 – 2.53 (m, 2.2H), 2.51 (s, 1.8H), 2.40 (s, 3H), 2.29 (s, 3H), 2.28 (s, 1.8H), 2.25 – 1.74 (m, 8.4H), 1.37 (d, J = 7.3 Hz, 3H), 0.81 (t, J = 7.4 Hz, 1.8H).

^{13}C NMR (100 MHz, CDCl$_3$) δ 142.6, 142.0, 139.7, 137.8, 137.3, 136.1, 134.9, 131.2, 129.1, 128.5, 128.3, 128.1, 125.7, 77.4, 77.1, 76.8, 45.0, 40.9, 37.3, 34.7, 34.2, 26.3, 21.9, 21.6, 21.5, 20.7, 20.7, 19.1, 13.0.

1-(3,4-Dimethylphenyl)adamantane (38)[11,12]

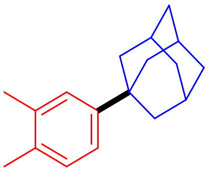

Following General Procedure A, compound **38** was obtained from 1-adamantylamine (151 mg, 1.00 mmol) and o-xylene (531 mg, 5.00 mmol). The reaction mixture was stirred with MsOH at room temperature for 16 h. Purified by column chromatography (petroleum ether) to afford 225 mg of compound **38** as a white solid. **(Yield = 94%).**

^{1}H NMR (400 MHz, $CDCl_3$) δ 7.18 (s, 1H), 7.17 – 7.10 (m, 2H), 2.31 (s, 3H), 2.28 (s, 3H), 2.16 – 2.10 (m, 3H), 1.95 (d, *J* = 2.7 Hz, 6H), 1.87 – 1.76 (m, 6H).

^{13}C NMR (100 MHz, $CDCl_3$) δ 149.0, 136.0, 133.6, 129.4, 126.2, 122.2, 43.3, 36.9, 35.8, 29.1, 20.1, 19.2.

1-(4-Methylphenyl)adamantane (39)[13]

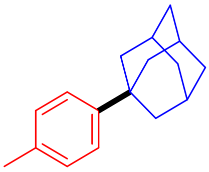

Following General Procedure A, compound **39** was obtained from 1-adamantylamine (151 mg, 1.00 mmol) and toluene (461 mg, 5.00 mmol). The reaction mixture was stirred with MsOH at room temperature for 16 h. Purified by column chromatography (petroleum ether) to afford 202 mg of compound **39** as a white solid. **(Yield = 89%).**

^{1}H NMR (400 MHz, $CDCl_3$) δ 7.33 – 7.27 (m, 2H), 7.20 – 7.14 (m, 2H), 2.36 (s, 3H), 2.17 – 2.09 (m, 3H), 1.95 (d, *J* = 2.8 Hz, 6H), 1.85 – 1.77 (m, 6H).

^{13}C NMR (100 MHz, $CDCl_3$) δ 148.5, 134.9, 128.8, 124.7, 43.3, 36.9, 35.9, 29.1, 20.9.

4-Cumylanisole (40)[14]

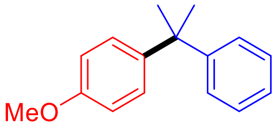

Following General Procedure A, compound **40** was obtained from cumylamine (135 mg, 1.00 mmol) and anisole (541 mg, 5.00 mmol). The reaction mixture was refluxed with NBu_4Br (322

mg, 1.00 mmol, added before the acid) and MsOH for 3 h. Purified by column chromatography (AcOEt/hexanes gradually from 0:100 to 3:97) to afford 123 mg of compound **40** as yellowish oil. **(Yield = 54%).**

^{1}H NMR (400 MHz, $CDCl_3$) δ 7.31 – 7.27 (m, 3H), 7.23 – 7.16 (m, 3H), 6.88 – 6.81 (m, 2H), 3.81 (s, 3H), 1.72 (s, 6H).

^{13}C NMR (100 MHz, $CDCl_3$) δ 157.5, 151.0, 142.9, 128.0, 127.8, 126.8, 125.6, 113.3, 55.2, 42.3, 30.9.

1-Ethoxy-4-(2,4,4-trimethylpentan-2-yl)benzene (41)[15]

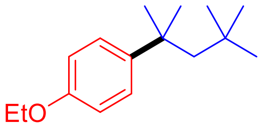

Following General Procedure A, compound **41** was obtained from *tert*-octylamine (129 mg, 1.00 mmol) and ethoxybenzene (611 mg, 5.00 mmol). The reaction mixture was refluxed with NBu_4Br (322 mg, 1.00 mmol, added before the acid) and MsOH at room temperature for 3 h. Purified by column chromatography (AcOEt/hexanes gradually from 0:100 to 3:97) to afford 176 mg of compound **41** as yellowish oil. **(Yield = 75%).**

^{1}H NMR (400 MHz, $CDCl_3$) δ 7.31 – 7.22 (m, 2H), 6.88 – 6.78 (m, 2H), 4.03 (q, J = 7.0 Hz, 2H), 1.72 (s, 2H), 1.41 (t, J = 7.0 Hz, 2H), 1.36 (s, 6H), 0.74 (s, 9H).

^{13}C NMR (100 MHz, $CDCl_3$) δ 156.6, 142.1, 127.0, 113.7, 63.3, 57.0, 37.9, 32.3, 31.8, 31.7, 14.9.

2-Mesitylpropanoic acid (42)[31]

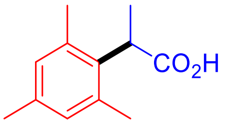

Following General Procedure D, compound **42** was obtained from alanine (89 mg, 1.00 mmol) and mesitylene (2404 mg, 20.00 mmol) with HFIP (6 mL) with Et_3N (140 µL, 1.00 mmol, 1.00 equiv.). Purified by column chromatography (DCM) to afford 89 mg of compound **42** as a yellowish solid. **(Yield = 46%).**

$[\alpha]D_{20}$ = −81.55 (c = 0.75 in $CHCl_3$). 81% ee.

^{1}H NMR (400 MHz, $CDCl_3$) δ 11.78 (s, 1H), 6.89 (s, 2H), 4.14 (q, J = 7.2 Hz, 1H), 2.32 (s, 6H), 2.29 (s, 3H), 1.47 (d, J = 7.2 Hz, 3H).

^{13}C NMR (100 MHz, $CDCl_3$) δ 181.6, 136.4, 136.0, 134.7, 129.8, 40.1, 20.8, 20.3, 15.3.

To determine the ratio of enantiomers the compound was transformed into a mixture of diasteromeric amides in the reaction with (*R*)-(+)-α-methylbenzylamine.

_N_-((_R_)-1-phenylethyl)-2-mesitylpropanamide (42a)

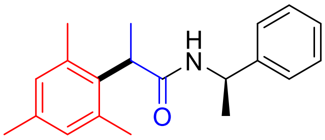

A 25 mL round-bottom flask equipped with a magnetic bar was charged with compound **42** (58 mg, 0.3 mmol, 1.0 equiv.), (R)-(+)-α-methylbenzylamine (121 mg, 1.00 mmol, 3.33 equiv.) and dry THF (1.0 mL). Then 4-dimethylaminopyridine (6.1 mg, 0.05 mmol, 0.17 equiv.) and *N*-(3-dimethylaminopropyl)-*N′*-ethylcarbodiimide hydrochloride (192 mg, 1.00 mmol, 3.33 equiv.) were added and flask was flushed with argon. The reaction mixture was stirred at room temperature for 4 h. Upon completion, the reaction mixture was transferred to separatory funnel with 40 mL Et_2O. Ethereal solution was washed with 3 x 25 mL 0.5 M HCl, 2 x 25 mL saturated $NaHCO_3$. The organic phase was dried with Na_2SO_4, filtered, and evaporated. A crude product **42a** was purified via column chromatography (AcOEt/hexanes, gradually from 10:90 to 30:70) to afford 60 mg of mixture of diastereoisomers (9.7:1) of compound **42a** as a yellowish oil. **(Yield = 68%).**

^{1}H NMR (600 MHz, $CDCl_3$) δ 7.30 – 7.23 (m, $2H_{major}$ + $2H_{minor}$), 7.22 – 7.18 (m, $1H_{major}$ + $1H_{minor}$), 7.16 – 7.10 (m, $2H_{major}$ + $2H_{minor}$), 6.86 (s, $1H_{minor}$), 6.81 (s, $1H_{major}$), 5.52 – 5.42 (m, $1H_{major}$ + $1H_{minor}$), 5.19 – 5.09 (m, $1H_{major}$ + $1H_{minor}$), 3.98 – 3.91 (m, $1H_{major}$ + $1H_{minor}$), 2.30 – 2.05 (m, $6H_{major}$ + $6H_{minor}$), 1.43 (d, J = 7.2 Hz, $3H_{minor}$), 1.40 (d, J = 7.2 Hz, $3H_{major}$), 1.38 – 1.34 (m, $3H_{major}$ + $3H_{minor}$).

^{13}C NMR (151 MHz, $CDCl_3$, major diastereoisomer) δ 173.8, 143.2, 136.7, 136.5, 135.0, 130.0, 128.4, 127.1, 126.2, 48.7, 40.8, 21.8, 20.8, 20.3, 14.7.

HRMS (m/z) (APCI) $[M+H]^+$ calculated for $C_{20}H_{26}NO$: 296.2014, found: 296.2016.

GC Chromatogram: diastereoisomer peak ratio: 9.7:1.

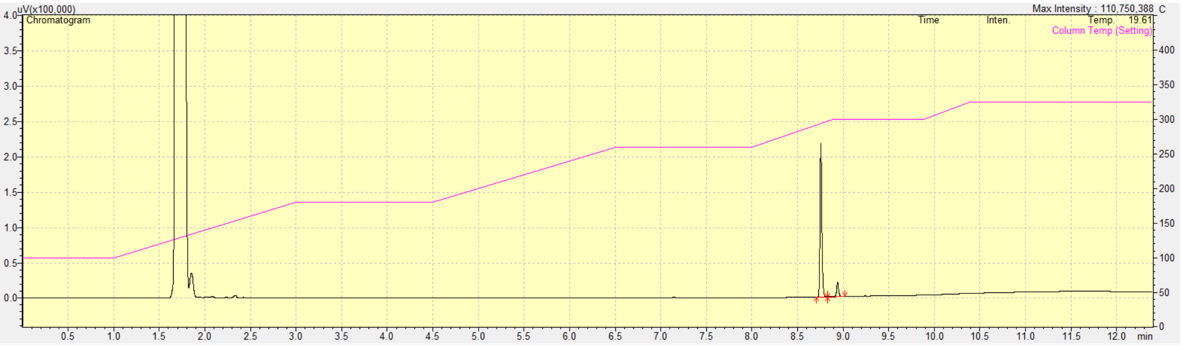

2-Mesityl-2-phenylacetic acid (43)

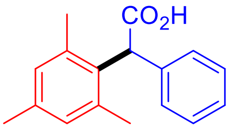

Following General Procedure D, compound **43** was obtained from phenylglycine (151 mg, 1.00 mmol) and mesitylene (2404 mg, 20.00 mmol) with HFIP (6 mL) with Et_3N (140 μL, 1.00 mmol, 1.00 equiv.). Purified by column chromatography (DCM) to afford 197 mg of compound **43** as a white solid. **(Yield = 78%).**

$[\alpha]D_{20}$ = 1.10 (*c* = 0.47 in $CHCl_3$). 10% ee.

^{1}H NMR (400 MHz, $CDCl_3$) δ 11.67 (s, 1H), 7.32 – 7.21 (m, 3H), 7.16 (d, *J* = 7.4 Hz, 2H), 6.93 (s, 2H), 5.46 (s, 1H), 2.31 (s, 3H), 2.22 (s, 6H).

^{13}C NMR (100 MHz, $CDCl_3$) δ 179.2, 137.5, 137.1, 136.1, 131.7, 130.0, 128.7, 128.2, 126.9, 50.7, 20.9.

HRMS (m/z) (ESI) $[M+Na]^+$ calculated for $C_{17}H_{18}O_2Na$: 277.1204, found: 277.1206.

mp: 170.4 – 171.8 °C

To determine the ratio of enantiomers the compound was transformed into a mixture of diasteromeric amides in the reaction with (R)-(+)-α-methylbenzylamine.

***N*-((*R*)-1-phenylethyl)-2-mesityl-2-phenylacetamide (43a)**

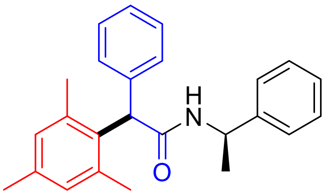

A 25 mL round-bottom flask equipped with a magnetic bar was charged with compound **43** (77 mg, 0.3 mmol, 1.0 equiv.), (R)-(+)-α-methylbenzylamine (121 mg, 1.00 mmol, 3.33 equiv.) and dry THF (1.0 mL). Then 4-dimethylaminopyridine (6.1 mg, 0.05 mmol, 0.17 equiv.) and *N*-(3-dimethylaminopropyl)-*N′*-ethylcarbodiimide hydrochloride (192 mg, 1.00 mmol, 3.33 equiv.) were added and flask was flushed with argon. The reaction mixture was stirred at room temperature for 4 h. Upon completion, the reaction mixture was transferred to separatory funnel with 40 mL Et_2O. Ethereal solution was washed with 3 x 25 mL 0.5 M HCl, 2 x 25 mL saturated $NaHCO_3$. The organic phase was dried with Na_2SO_4, filtered, and evaporated. A crude product was purified using column chromatography (AcOEt/hexanes gradually from 10:90 to 30:70) to

afford 49 mg of mixture of diastereoisomers (1.2:1) of compound **43a** as a yellowish oil. **(Yield = 46%).**

^{1}H NMR (600 MHz, $CDCl_3$) δ 7.32 – 7.14 (m, $10H_{major}$ + $10H_{minor}$), 6.90 – 6.86 (m, $2H_{major}$ + $2H_{minor}$), 5.74 – 5.67 (m, $1H_{major}$ + $1H_{minor}$), 5.28 (s, $1H_{minor}$), 5.26 (s, $1H_{major}$), 5.23 – 5.16 (m, $1H_{major}$ + $1H_{minor}$), 2.30 – 2.27 (m, $3H_{major}$ + $3H_{minor}$), 2.14 (s, $6H_{major}$), 2.10 (s, $6H_{minor}$), 1.40 (t, J = 6.5 Hz, $3H_{major}$ + $3H_{minor}$).

^{13}C NMR (151 MHz, $CDCl_3$) δ 171.2, 171.2, 143.1, 143.0, 137.6, 137.6, 137.2, 137.2, 136.9, 136.9, 132.8, 132.8, 130.1, 130.1, 129.3, 129.3, 128.6, 128.5, 128.3, 128.3, 127.2, 127.2, 126.7, 126.7, 126.1, 126.1, 52.8, 52.7, 48.9, 48.8, 21.7, 21.6, 21.1, 21.0, 20.9, 20.9.

HRMS (m/z) (APCI) $[M+H]^+$ calculated for $C_{25}H_{28}NO$: 358.2171, found: 358.2176.

GC Chromatogram: diastereoisomer peak ratio: 1.2:1.

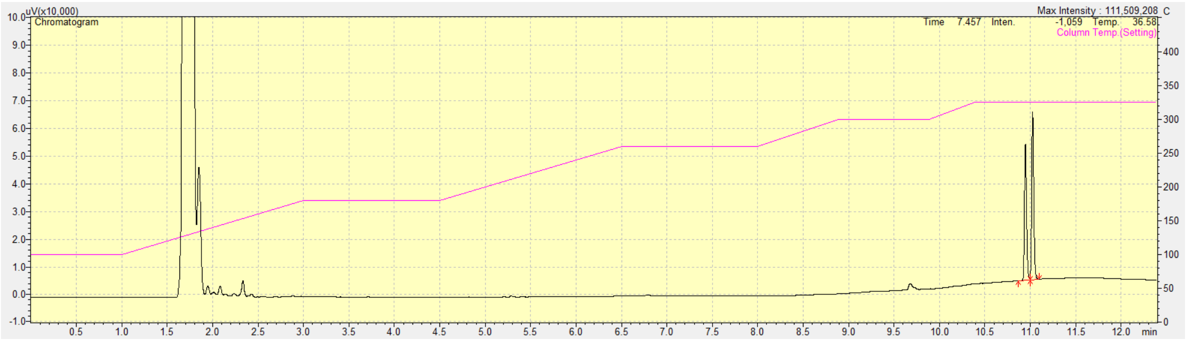

4-Nitrobiphenyl (44)[28]

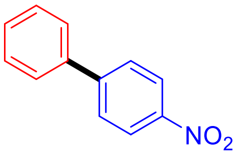

Following General Procedure D, compound **44** was obtained from 4-nitroaniline (138 mg, 1.00 mmol) and benzene (1564 mg, 20.0 mmol) with HFIP (0,5 mL). Purified by column chromatography (AcOEt/hexanes gradually from 0:100 to 3:97) to afford 126 mg of compound **44** as a yellow solid. **(Yield = 63%).**

^{1}H NMR (400 MHz, $CDCl_3$) δ 8.35 – 8.24 (m, 2H), 7.77 – 7.70 (m, 2H), 7.66 – 7.59 (m, 2H), 7.53 – 7.41 (m, 3H).

^{13}C NMR (100 MHz, $CDCl_3$) δ 147.6, 147.1, 138.8, 129.2, 128.9, 127.8, 127.4, 124.1.

4-Hydroxyazobenzene (45)[29]

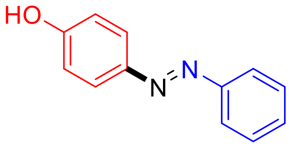

Following General Procedure D, compound **45** was obtained from aniline (93 mg, 1.00 mmol) and phenol (471 mg, 5.00 mmol) with HFIP (2 mL). Purified by column chromatography (AcOEt/hexanes gradually from 5:95 to 10:90) to afford 164 mg of compound **45** as orange solid. **(Yield = 83%).**

^{1}H NMR (400 MHz, DMSO) δ 10.26 (s, 1H), 7.85 – 7.73 (m, 4H), 7.58 – 7.49 (m, 2H), 7.49 – 7.36 (m, 1H), 6.93 (d, J = 8.8 Hz, 2H).

^{13}C NMR (100 MHz, DMSO) δ 161.4, 152.6, 145.7, 130.9, 129.8, 125.3, 122.6, 116.4.

Methyl red (46)[30]

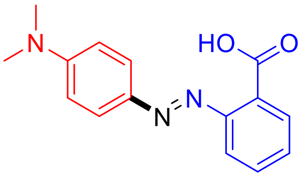

Following General Procedure D, compound **46** was obtained from anthranilic acid (137 mg, 1.00 mmol) and *N*,*N*-dimethylaniline (606 mg, 5.00 mmol) with 2 mL HFIP. Purified by column chromatography (AcOEt/hexanes gradually from 60:40 to 80:20) to afford 245 mg of compound **46** as a dark red solid. **(Yield = 91%).**

^{1}H NMR (400 MHz, $CDCl_3$) δ 14.23 (s, 1H), 8.40 – 8.31 (m, 1H), 8.02 – 7.93 (m, 1H), 7.82 – 7.72 (m, 2H), 7.64 – 7.56 (m, 1H), 7.52 – 7.44 (m, 1H), 6.78 – 6.67 (m, 2H), 3.13 (s, 6H).

^{13}C NMR (100 MHz, $CDCl_3$) δ 167.3, 154.1, 150.4, 142.1, 133.5, 132.6, 129.9, 127.0, 125.2, 115.6, 111.9, 40.3.

Diphenylmethane (47)[2]

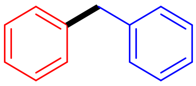

Following General Procedure A, compound **47** was obtained from benzylamine (107 mg, 1.00 mmol) and benzene (391 mg, 5.00 mmol). The reaction mixture was refluxed with MsOH for

4.5 h. Purified by column chromatography (petroleum ether) to afford 102 mg of compound **47** as colorless oil. **(Yield = 61%).**

^{1}H NMR (400 MHz, CDCl$_3$) δ 7.41 – 7.31 (m, 4H), 7.30 – 7.23 (m, 6H), 4.06 (s, 2H).

^{13}C NMR (100 MHz, CDCl$_3$) δ 141.2, 129.0, 128.5, 126.1, 42.0.

2-Benzyl-1,4-dimethylbenzene (8)[16]

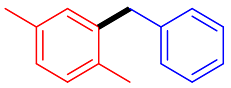

Following General Procedure A, compound **8** was obtained from benzylamine (107 mg, 1.00 mmol) and *p*-xylene (531 mg, 5.00 mmol). The reaction mixture was refluxed with MsOH for 3 h. Purified by column chromatography (petroleum ether) to afford 160 mg of compound **8** as colorless oil. **(Yield = 82%).**

^{1}H NMR (400 MHz, CDCl$_3$) δ 7.37 – 7.29 (m, 2H), 7.27 – 7.21 (m, 1H), 7.21 – 7.15 (m, 2H), 7.12 (d, *J* = 7.6 Hz, 1H), 7.07 – 6.97 (m, 2H), 4.02 (s, 2H), 2.36 (s, 3H), 2.26 (s, 3H).

^{13}C NMR (100 MHz, CDCl$_3$) δ 140.6, 138.7, 135.4, 133.5, 130.8, 130.3, 128.8, 128.4, 127.2, 125.9, 39.5, 21.0, 19.2.

Benzylpentamethylbenzene (48)[17]

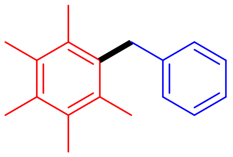

Following General Procedure A, compound **48** was obtained from benzylamine (107 mg, 1.00 mmol) and pentamethylbenzene (741 mg, 5.00 mmol). The reaction mixture was refluxed with MsOH for 3 h. Purified by column chromatography (petroleum ether) to afford 224 mg of compound **48** as a yellowish solid. **(Yield = 94%).**

^{1}H NMR (400 MHz, CDCl$_3$) δ 7.33 – 7.18 (m, 3H), 7.14 – 7.08 (m, 2H), 4.19 (s, 2H), 2.35 (s, 3H), 2.32 (s, 6H), 2.25 (s, 6H).

^{13}C NMR (100 MHz, CDCl$_3$) δ 140.7, 133.9, 133.2, 132.9, 132.5, 128.4, 128.0, 125.6, 36.2, 17.0, 16.9, 16.9.

4-(2-Methylbenzyl)-*tert*-butylbenzene (49)[18]

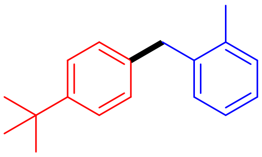

Following General Procedure A, compound **49** was obtained from 2-methylbenzylamine (121 mg, 1.00 mmol) and *tert*-butylbenzene (671 mg, 5.00 mmol). The reaction mixture was refluxed with MsOH for 3 h. Purified by column chromatography (petroleum ether) to afford 182 mg of compound **49** as colorless oil. **(Yield = 76%).**

^{1}H NMR (400 MHz, $CDCl_3$) δ 7.37 – 7.32 (m, 2H), 7.22 – 7.16 (m, 4H), 7.13 – 7.09 (m, 2H), 4.01 (s, 2H), 2.31 (s, 3H), 1.36 (s, 9H).

^{13}C NMR (100 MHz, $CDCl_3$) δ 148.7, 139.2, 137.3, 136.6, 130.3, 130.0, 128.4, 126.4, 126.0, 125.30, 38.9, 34.4, 31.5, 19.7.

(4-Bromobenzyl)benzene (50)[5]

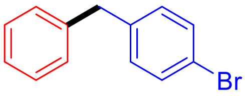

Following General Procedure A, compound **50** was obtained from 4-bromobenzylamine (186 mg, 1.00 mmol) and benzene (391 mg, 5.00 mmol). The reaction mixture was refluxed with MsOH for 4.5 h. Purified by column chromatography (petroleum ether) to afford 207 mg of compound **50** as colorless oil. **(Yield = 84%).**

^{1}H NMR (400 MHz, $CDCl_3$) δ 7.46 – 7.41 (m, 2H), 7.36 – 7.30 (m, 2H), 7.28 – 7.22 (m, 1H), 7.22 – 7.17 (m, 2H), 7.12 – 7.05 (m, 2H), 3.96 (s, 2H).

^{13}C NMR (100 MHz, $CDCl_3$) δ 140.5, 140.1, 131.6, 130.7, 128.9, 128.6, 126.4, 120.0, 41.4.

4-(4-Bromobenzyl)-1,3-difluorobenzene (51)[2]

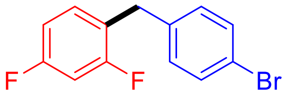

Following General Procedure A, compound **51** was obtained from 4-bromobenzylamine (186 mg, 1.00 mmol) and 1,3-difluorobenzene (570 mg, 5.00 mmol). The reaction mixture with MsOH was refluxed for 21 h. Purified by column chromatography (petroleum ether) to afford 214 mg of compound **51** as colorless oil. **(Yield = 76%).**

^{1}H NMR (400 MHz, $CDCl_3$) δ 7.44 – 7.39 (m, 2H), 7.15 – 7.04 (m, 3H), 6.85 – 6.76 (m, 2H), 3.91 (s, 2H).

^{13}C NMR (100 MHz, $CDCl_3$) δ 162.5 (dd, *J* = 100.9, 11.8 Hz), 160.1 (dd, *J* = 101.8, 11.9 Hz), 138.6, 131.7, 131.4 (dd, *J* = 9.5, 6.1 Hz), 130.4, 123.3 (dd, *J* = 16.0, 3.9 Hz), 120.3, 111.2 (dd, *J* = 21.0, 3.8 Hz), 103.9 (t, *J* = 25.6 Hz), 33.8 (d, *J* = 2.6 Hz).

4,4'-Dianisylmethane (52)[2] (containing 8% ortho isomer)

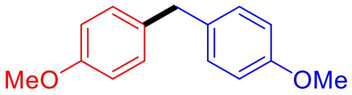

Following General Procedure A, compound **52** was obtained from 4-methoxybenzylamine (137 mg, 1.00 mmol) and anisole (541 mg, 5.00 mmol). The reaction mixture was stirred with MsOH at room temperature for 16 h. Purified by column chromatography (AcOEt/hexanes gradually from 0:100 to 3:97) to afford 182 mg of compound **52** (containing 8% *ortho* isomer) as yellowish oil. **(Yield = 80%).**

^{1}H NMR (400 MHz, $CDCl_3$) δ 7.17 – 7.09 (m, 4H), 6.89 – 6.82 (m, 4H), 3.90 (s, 2H), 3.80 (s, 6H).

^{13}C NMR (100 MHz, $CDCl_3$) δ 158.0, 133.8, 129.9, 129.8, 113.9, 113.6, 55.3, 40.2.

GC Chromatogram: peak ratio: 11.5:1 (8% *ortho* isomer).

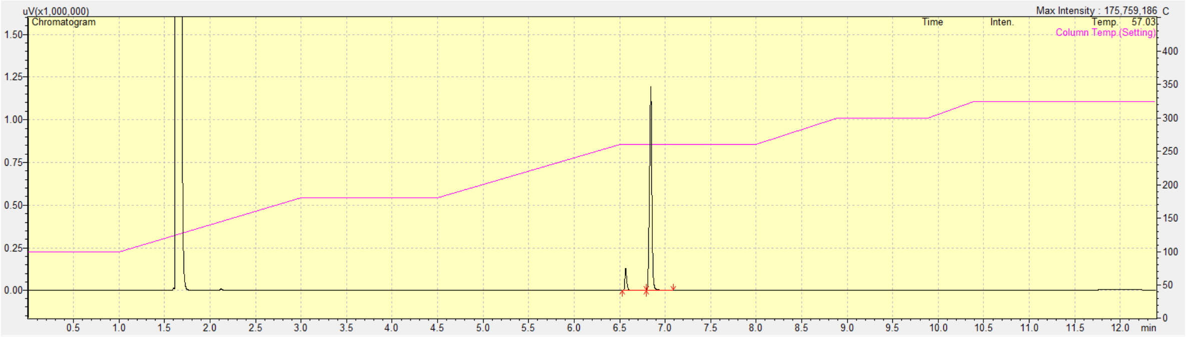

1-(4-Methoxyphenyl)adamantane (53)[19]

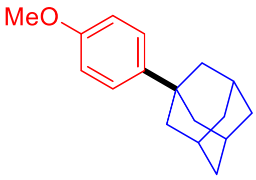

Following General Procedure A, compound **53** was obtained from 1-adamantylamine (151 mg, 1.00 mmol) and anisole (541 mg, 5.00 mmol). The reaction mixture was stirred at room temperature for 16 h with MsOH. Purified by column chromatography (AcOEt/hexanes

gradually from 0:100 to 3:97) to afford 240 mg of compound **53** as a white solid. **(Yield = 99%).**

^{1}H NMR (400 MHz, CDCl$_3$) δ 7.32 – 7.27 (m, 2H), 6.90 – 6.85 (m, 2H), 3.80 (s, 3H), 2.13 – 2.06 (m, 3H), 1.94 – 1.88 (m, 6H), 1.83 – 1.71 (m, 6H).

^{13}C NMR (100 MHz, CDCl$_3$) δ 157.4, 143.7, 125.8, 113.4, 77.3, 77.0, 76.7, 55.2, 43.4, 36.8, 35.6, 29.0.

4-(Adamantan-1-yl)acetanilide (54)[20]

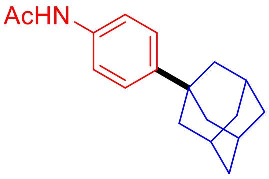

Following General Procedure A, compound **54** was obtained from 1-adamantylamine (151 mg, 1.00 mmol) and acetanilide (676 mg, 5.00 mmol). The reaction mixture was refluxed with MsOH for 16 h. Purified by column chromatography (AcOEt/hexanes gradually from:70 to 50:50) to afford 214 mg of compound **54** as a white solid. **(Yield = 79%).**

^{1}H NMR (400 MHz, CDCl$_3$) δ 7.41 (d, J = 8.7 Hz, 2H), 7.30 (d, J = 8.7 Hz, 2H), 7.22 (s, 1H), 2.15 (s, 3H), 2.08 (s, 3H), 1.91 – 1.85 (m, 6H), 1.81 – 1.70 (m, 6H).

^{13}C NMR (100 MHz, CDCl$_3$) δ 168.2, 147.6, 135.3, 125.4, 119.8, 43.2, 36.8, 35.9, 28.9, 24.5.

2-(Adamantan-1-yl)-4-bromoanisole (55)[27]

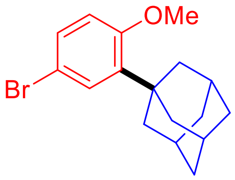

Following General Procedure B, compound **55** was obtained from 1-adamantylamine (151 mg, 1.00 mmol) and 4-bromoanisole (935 mg, 5.00 mmol). The reaction mixture was stirred with MsOH at room temperature for 36 h. Purified by column chromatography (AcOEt/hexanes gradually from 0:100 to 3:97) to afford 219 mg of compound **55** as a white solid. **(Yield = 68%).**

^{1}H NMR (400 MHz, CDCl$_3$) δ (d, J = 2.5 Hz, 1H), 7.26 (dd, J = 8.6, 2.5 Hz, 1H), 6.73 (d, J = 8.6 Hz, 1H), 3.81 (s, 3H), 2.06 (s, 9H), 1.77 (s, 6H).

^{13}C NMR (100 MHz, CDCl$_3$) δ 157.9, 140.8, 129.8, 129.3, 113.3, 113.3, 55.2, 40.4, 37.2, 37.0, 29.0.

1-(4-Hydroxyphenyl)adamantane (56)[12,21]

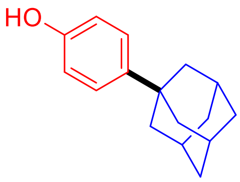

Following General Procedure B, compound **56** was obtained from 1-adamantylamine (151 mg, 1.00 mmol) and phenol (471 mg, 5.00 mmol). The reaction mixture was stirred with MsOH at room temperature for 16 h. The reaction mixture was quenched with water (15 mL), the resulting precipitate was filtered and washed with water/MeOH (4:1, 50 mL) to afford 219 mg of compound **56** as a white solid. **(Yield = 96%).**

^{1}H NMR (400 MHz, $CDCl_3$) δ 7.24 (d, *J* = 8.5 Hz, 2H), 6.81 (d, *J* = 8.3 Hz, 2H), 4.72 (s, 1H), 2.18 – 1.99 (m, 3H), 1.98 – 1.84 (m, 6H), 1.83 – 1.67 (m, 6H).

^{13}C NMR (100 MHz, $CDCl_3$) δ 153.1, 144.0, 126.0, 114.8, 43.4, 36.8, 35.6, 29.0.

3-(Adamantan-1-yl)-2,6-dihydroxybenzaldehyde (57)

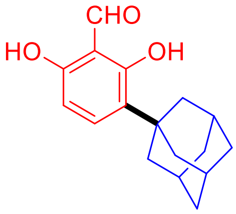

Following General Procedure B, compound **57** was obtained from 1-adamantylamine (151 mg, 1.00 mmol) and 2,6-dihydroxybenzaldehyde (691 mg, 5.00 mmol). The reaction mixture was stirred with MsOH at room temperature for 30 min. Purified by column chromatography (AcOEt/toluene gradually from 0:100 to 5:95) to afford 177 mg of compound **57** as a yellow solid. **(Yield = 65%)**

^{1}H NMR (400 MHz, DMSO) δ 12.57 (s, 1H), 10.60 (s, 1H), 10.21 (s, 1H), 7.24 (d, *J* = 8.7 Hz, 1H), 6.33 (d, *J* = 8.7 Hz, 1H), 1.99 (s, 9H), 1.69 (s, 6H).

^{13}C NMR (100 MHz, DMSO) δ 195.6, 162.0, 160.3, 136.6, 127.4, 110.3, 105.9, 37.0, 36.1, 28.8.

HRMS (m/z) (APCI) $[M+H]^+$ calculated for $C_{17}H_{21}O_3$: 273.1491, found: 273.1490.

mp: – decomposes before melting

3-(Adamantan-1-yl)-2,6-dihydroxyacetophenone (58)

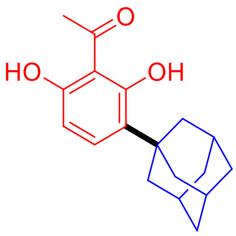

Following General Procedure B, compound **58** was obtained from 1-adamantylamine (151 mg, 1.00 mmol) and 2,6-dihydroxyacetophenone (761 mg, 5.00 mmol). The reaction mixture was stirred with MsOH at room temperature for 16 h. Purified by column chromatography (AcOEt/toluene gradually from 0:100 to 5:95) to afford 257 mg of compound **58** as a yellow solid. **(Yield = 90%)**

^{1}H NMR (400 MHz, DMSO) δ 13.96 (s, 1H), 10.54 (s, 1H), 7.15 (d, *J* = 8.5 Hz, 1H), 6.34 (d, *J* = 8.6 Hz, 1H), 2.63 (s, 3H), 1.99 (s, 9H), 1.68 (s, 6H).

^{13}C NMR (100 MHz, DMSO**)** δ 206.6, 163.2, 159.2, 134.1, 127.6, 110.2, 106.0, 37.1, 36.2, 33.9, 28.9.

HRMS (m/z) (APCI) $[M+H]^+$ calculated for $C_{18}H_{23}O_3$: 287.1647, found: 287.1644.

mp: – decomposes before melting

Methyl 3-(adamantan-1-yl)-2,6-dihydroxybenzoate (59)

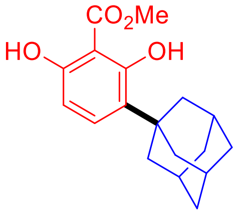

Following General Procedure B, compound **59** was obtained from 1-adamantylamine (151 mg, 1.00 mmol) and methyl 2,6-dihydroxybenzoate (841 mg, 5.00 mmol). The reaction mixture was stirred with MsOH at room temperature for 16 h. Purified by column chromatography (30:70 toluene/hexanes) to afford 204 mg of compound **59** as a white solid. **(Yield = 67%)**

^{1}H NMR (400 MHz, $CDCl_3$) δ 10.72 (s, 1H), 9.18 (s, 1H), 7.29 (d, *J* = 8.8 Hz, 1H), 6.43 (d, *J* = 8.8 Hz, 1H), 4.07 (s, 3H), 2.08 (s, 9H), 1.76 (s, 6H).

^{13}C NMR (100 MHz, $CDCl_3$) δ 170.7, 160.5, 158.2, 134.3, 128.6, 107.0, 99.9, 52.8, 40.4, 37.1, 36.5, 29.1.

HRMS (m/z) (APCI) $[M+H]^+$ calculated for $C_{18}H_{23}O_4$: 303.1596, found: 303.1600.

mp: 151.9 – 152.6 °C

4-(Adamantan-1-yl)-2-nitroresorcinol (60)

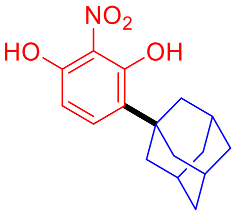

Following General Procedure B, compound **60** was obtained from 1-adamantylamine (151 mg, 1.00 mmol) and 2-nitroresorcinol (776 mg, 5.00 mmol). The reaction mixture was stirred with MsOH at room temperature for 16 h. Purified by column chromatography (10:90 toluene/hexanes) to afford 226 mg of compound **60** as an orange solid. **(Yield = 78%)**

^{1}H NMR (400 MHz, $CDCl_3$) δ 11.68 (s, 1H), 10.53 (s, 1H), 7.43 (d, *J* = 9.0 Hz, 1H), 6.55 (d, *J* = 9.0 Hz, 1H), 2.08 (s, 9H), 1.77 (s, 6H).

^{13}C NMR (100 MHz, $CDCl_3$) δ 155.6, 154.2, 137.0, 129.9, 124.3, 107.9, 40.3, 36.9, 29.0.

HRMS (m/z) (APCI) $[M-H]^-$ calculated for $C_{16}H_{18}NO_4$: 288.1236, found: 288.1237.

mp: 147.4 – 148.8 °C

(8*R*,9*S*,13*S*,14*S*)-2-Benzhydryl-3-hydroxy-13-methyl-6,7,8,9,11,12,13,14,15,16-decahydro-17H-cyclopenta[a]phenanthren-17-one (61)[2]

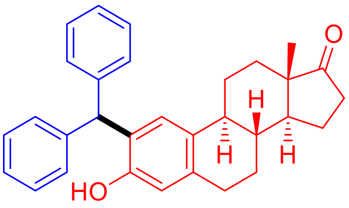

Following General Procedure C, compound **61** was obtained from benzhydrylamine (183 mg, 1.00 mmol) and estrone (811 mg, 3.00 mmol). The reaction mixture was stirred with MsOH at room temperature for 15 min. Purified by column chromatography (DCM/hexanes gradually from 80:20 to 100:0) to afford 415 mg of compound **61** as white solid. **(Yield = 95%).**

^{1}H NMR (400 MHz, DMSO) δ 9.08 (s, 1H), 7.32 – 7.22 (m, 4H), 7.22 – 7.13 (m, 2H), 7.06 (t, *J* = 7.7 Hz, 4H), 6.68 (s, 1H), 6.52 (s, 1H), 5.80 – 5.73 (m, 1H), 2.84 – 2.66 (m, 2H), 2.41 (dd, *J* = 18.8, 8.4 Hz, 1H), 2.17 – 1.83 (m, 5H), 1.69 – 1.60 (m, 1H), 1.60 – 1.12 (m, 6H), 0.79 (s, 3H).

^{13}C NMR (100 MHz, DMSO) δ 152.9, 144.5, 144.4, 135.4, 129.9, 129.5, 129.5, 128.5, 128.5, 127.9, 127.1, 126.3, 115.4, 50.0, 49.7, 47.8, 44.0, 38.4, 35.8, 31.7, 29.2, 26.6, 26.0, 21.6, 14.0.

2-(4-Methoxybenzyl)-4-methylaniline (62)

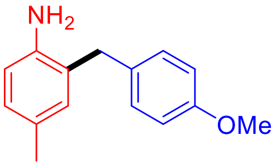

Following General Procedure B, compound **62** was obtained from 4-methoxybenzylamine (137 mg, 1.00 mmol) and 4-methylaniline (536 mg, 5.00 mmol). The reaction mixture was stirred with TfOH at 140 °C for 16 h. Purified by column chromatography (AcOEt/hexanes gradually from 5:95 to 15:85) to afford 173 mg of compound **62** as yellowish oil. **(Yield = 76%)**

^{1}H NMR (400 MHz, CDCl$_3$) δ 7.17 – 7.11 (m, 2H), 6.97 – 6.90 (m, 2H), 6.89 – 6.84 (m, 2H), 6.62 (d, *J* = 7.8 Hz, 1H), 3.85 (s, 2H), 3.80 (s, 3H), 3.42 (s, 2H), 2.29 (s, 3H).

^{13}C NMR (100 MHz, CDCl$_3$) δ 158.2, 142.2, 131.6, 131.4, 129.5, 128.1, 127.9, 125.7, 116.1, 114.1, 55.3, 37.3, 20.5.

HRMS (m/z) (EI) [M] calculated for $C_{15}H_{17}NO$: 227.1310, found: 227.1313.

2-(4-Methoxybenzyl)-4,*N*,*N*-trimethylaniline (63)

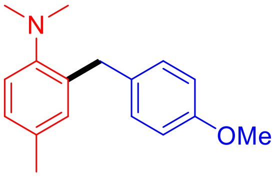

Following General Procedure C, compound **63** was obtained from 4-methoxybenzylamine (137 mg, 1.00 mmol) and 4,*N*,*N*-trimethylaniline (676 mg, 5.00 mmol). The reaction mixture was refluxed with TfOH for 96 h. Purified by column chromatography (AcOEt/toluene gradually from 0:100 to 10:90) to afford 184 mg of compound **63** as yellowish oil. **(Yield = 72%)**

^{1}H NMR (400 MHz, CDCl$_3$) δ 7.15 – 7.11 (m, 2H), 7.06 (d, *J* = 8.1 Hz, 1H), 7.02 – 6.97 (m, 1H), 6.88 – 6.85 (m, 1H), 6.85 – 6.80 (m, 2H), 4.02 (s, 2H), 3.79 (s, 3H), 2.65 (s, 6H), 2.23 (s, 3H).

^{13}C NMR (100 MHz, CDCl$_3$) δ 157.7, 150.4, 136.3, 133.9, 132.8, 131.3, 120.0, 127.4, 119.5, 113.7, 55.2, 45.4, 35.6, 20.8.

HRMS (m/z) (APCI) $[M+H]^+$ calculated for $C_{17}H_{22}NO$: 256.1701, found: 256.1702.

2-Benzhydryl-4-methoxyaniline (64)[22]

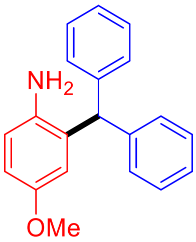

Following General Procedure C, compound **64** was obtained from benzhydrylamine (183 mg, 1.00 mmol) and 4-methoxyaniline (616 mg, 5.00 mmol). The reaction mixture was refluxed with TfOH for 16 h. Purified by column chromatography (AcOEt/toluene gradually from 0:100 to 10:90) to afford 245 mg of compound **64** as a brown solid. **(Yield = 85%).**

^{1}H NMR (400 MHz, $CDCl_3$) δ 7.36 – 7.29 (m, 4H), 7.28 – 7.22 (m, 2H), 7.20 – 7.12 (m, 4H), 6.73 – 6.62 (m, 2H), 6.39 – 6.30 (m, 1H), 5.53 (s, 1H), 3.64 (s, 3H), 3.21 (s, 2H).

^{13}C NMR (100 MHz, $CDCl_3$) δ 152.8, 142.4, 137.9, 131.2, 129.5, 128.6, 126.7, 117.3, 116.8, 112.1, 55.5, 52.4.

2-Benzhydryl-4-bromoaniline (65)

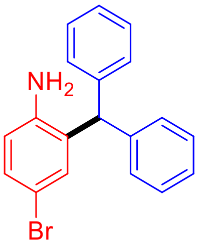

Following General Procedure C, compound **65** was obtained from benzhydrylamine (183 mg, 1.00 mmol) and 4-bromoaniline (860 mg, 5.00 mmol). The reaction mixture was refluxed with TfOH for 16 h. Purified by column chromatography (toluene/hexanes gradually from 50:50 to 100:0) to afford 310 mg of compound **65** as a brown solid. **(Yield = 92%)**

^{1}H NMR (400 MHz, $CDCl_3$) δ 7.37 – 7.24 (m, 6H), 7.19 (dd, *J* = 8.4, 2.3 Hz, 1H), 7.15 – 7.10 (m, 4H), 6.77 (d, *J* = 2.1 Hz, 1H), 6.56 (d, *J* = 8.4 Hz, 1H), 5.42 (s, 1H), 3.47 (s, 2H).

^{13}C NMR (100 MHz, $CDCl_3$) δ 143.4, 141.6, 132.5, 131.3, 130.3, 129.4, 128.8, 127.0, 117.8, 110.7, 52.2.

HRMS (m/z) (APCI) $[M+H]^+$ calculated for $C_{19}H_{17}NBr$: 338.0544, found: 338.0543.

mp: 94.4 – 96.1 °C

2-Benzhydryl-4-nitroaniline (66)

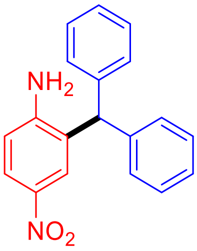

Following General Procedure C, compound **66** was obtained from benzhydrylamine (183 mg, 1.00 mmol) and 4-nitroaniline (691 mg, 5.00 mmol). The reaction mixture was refluxed with TfOH for 16 h. Purified by column chromatography (toluene) to afford 275 mg of compound **66** as a yellow solid. **(Yield = 91%)**

^{1}H NMR (400 MHz, CDCl$_3$) δ 8.01 (dd, *J* = 8.8, 2.6 Hz, 1H), 7.62 (d, *J* = 2.6 Hz, 1H), 7.41 – 7.23 (m, 6H), 7.20 – 7.06 (m, 4H), 6.64 (d, *J* = 8.8 Hz, 1H), 5.38 (s, 1H), 4.24 (s, 2H).

^{13}C NMR (100 MHz, CDCl$_3$) δ 150.5, 140.7, 139.3, 129.2, 129.0, 127.9, 127.4, 126.5, 124.4, 114.7, 52.2.

HRMS (m/z) (APCI) $[M+H]^+$ calculated for $C_{19}H_{17}N_2O_2$: 305.1290, found: 305.1289.

mp: 179.8 – 181.1 °C

3-(4-Methoxybenzyl)indole (67)[23]

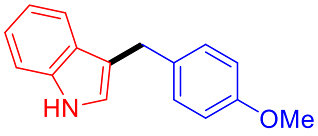

Following General Procedure C, compound **67** was obtained from 4-methoxybenzylamine (137 mg, 1.00 mmol) and indole (586 mg, 5.00 mmol). The reaction mixture was refluxed with MsOH for 1 h. Purified by column chromatography (AcOEt/hexanes gradually from 5:95 to 15:85) to afford 173 mg of compound **67** as a red solid. **(Yield = 73%).**

^{1}H NMR (400 MHz, CDCl$_3$) δ 7.99 – 7.82 (m, 1H), 7.57 – 7.52 (m, 1H), 7.38 – 7.32 (m, 1H), 7.25 – 7.17 (m, 3H), 7.14 – 7.07 (m, 1H), 6.91 – 6.88 (m, 1H), 6.88 – 6.83 (m, 2H), 4.09 (s, 2H), 3.80 (s, 3H).

^{13}C NMR (100 MHz, CDCl$_3$) δ 157.9, 136.5, 133.4, 129.6, 127.5, 122.2, 122.0, 119.3, 119.2, 116.3, 113.8, 111.1, 55.3, 30.7.

3-(4- Methoxybenzyl)-1-methylindole (68)[24]

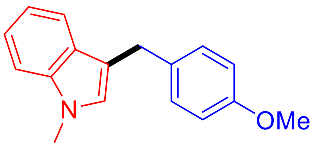

Following General Procedure C, compound **68** was obtained from 4-methoxybenzylamine (137 mg, 1.00 mmol) and 1-methylindole (656 mg, 5.00 mmol). The reaction mixture was refluxed with MsOH for 1 h. Purified by column chromatography (AcOEt/hexanes gradually from 5:95 to 15:85) to afford 174 mg of compound **68** as brown oil. **(Yield = 69%).**

^{1}H NMR (400 MHz, $CDCl_3$) δ 7.55 (d, *J* = 7.9 Hz, 1H), 7.34 – 7.29 (m, 1H), 7.28 – 7.21 (m, 3H), 7.13 – 7.07 (m, 1H), 6.89 – 6.84 (m, 2H), 6.76 (s, 1H), 4.08 (s, 2H), 3.81 (s, 3H), 3.74 (s, 3H).

^{13}C NMR (100 MHz, $CDCl_3$) δ 157.9, 137.2, 133.6, 129.6, 127.9, 127.0, 121.6, 119.2, 118.8, 114.8, 113.8, 109.2, 55.3, 32.6, 30.7.

3-(4- Methoxybenzyl)-1-tosylindole (69)[25]

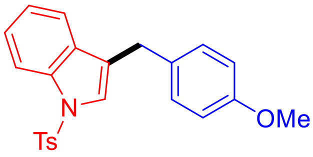

Following General Procedure A, compound **69** was obtained from 4-methoxybenzylamine (137 mg, 1.00 mmol) and 1-tosylindole (1357 mg, 5.00 mmol). The reaction mixture was stirred with MsOH at room temperature for 16 h. Purified by column chromatography (50:50 toluene/hexanes) to afford 300 mg of compound **69** as a yellowish solid. **(Yield = 77%).**

^{1}H NMR (400 MHz, $CDCl_3$) δ 8.01 (d, *J* = 8.3 Hz, 1H), 7.75 (s, 1H), 7.73 (s, 1H), 7.39 (d, *J* = 7.8 Hz, 1H), 7.34 – 7.25 (m, 2H), 7.23 – 7.16 (m, 3H), 7.14 (s, 1H), 7.12 (s, 1H), 6.84 (d, *J* = 8.6 Hz, 2H), 3.95 (s, 2H), 3.80 (s, 3H), 2.33 (s, 3H).

^{13}C NMR (100 MHz, $CDCl_3$) δ 158.2, 144.7, 135.6, 135.4, 131.0, 130.9, 129.8, 129.6, 126.8, 124.7, 123.9, 123.1, 123.0, 119.8, 114.0, 113.8, 55.3, 30.5, 21.5.

2,5-Dimethyl-3-(naphthalen-1-ylmethyl)thiophene (70)

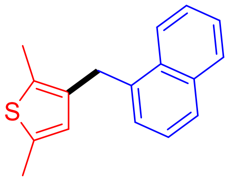

Following General Procedure C, compound **70** was obtained from 1-naphthylmethylamine (157 mg, 1.00 mmol) and 2,5-dimethylthiophene (561 mg, 5.00 mmol). The reaction mixture was stirred with MsOH at room temperature for 1 h. Purified by column chromatography (petroleum ether) to afford 222 mg of compound **70** as yellowish oil. **(Yield = 88%)**

^{1}H NMR (400 MHz, $CDCl_3$) δ 8.09 – 8.04 (m, 1H), 7.92 – 7.87 (m, 1H), 7.77 (d, *J* = 8.2 Hz, 1H), 7.58 – 7.48 (m, 2H), 7.46 – 7.39 (m, 1H), 7.24 – 7.19 (m, 1H), 6.32 (s, 1H), 4.26 (s, 2H), 2.41 (s, 3H), 2.35 (s, 3H).

^{13}C NMR (100 MHz, $CDCl_3$) δ 136.7, 135.3, 135.2, 133.8, 132.1, 131.0, 128.7, 127.6, 126.9, 126.2, 126.0, 125.7, 125.6, 123.8, 31.5, 15.2, 13.1.

HRMS (m/z) (EI) [M] calculated for $C_{17}H_{16}S$: 252.0973, found: 252.0979.

3-(Adamantan-1-yl)benzothiophene (71)[26]

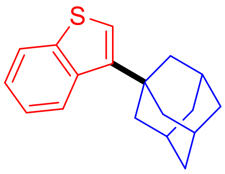

Following General Procedure B, compound **71** was obtained from 1-adamantylamine (151 mg, 1.00 mmol) and benzothiophene (671 mg, 5.00 mmol). The reaction mixture was stirred with MsOH at room temperature for 15 min. Purified by column chromatography (petroleum ether) to afford 254 mg of compound **71** as white solid. **(Yield = 94%).**

^{1}H NMR (400 MHz, $CDCl_3$) δ 8.24 – 8.18 (m, 1H), 7.91 – 7.86 (m, 1H), 7.39 – 7.27 (m, 2H), 7.08 (s, 1H), 2.24 – 2.14 (m, 9H), 1.92 – 1.85 (m, 6H).

^{13}C NMR (100 MHz, $CDCl_3$) δ 146.2, 141.8, 137.5, 124.7, 123.4, 123.4, 123.0, 119.8, 42.1, 37.1, 37.0, 28.9.

GC Chromatogram: purity: 99.6%

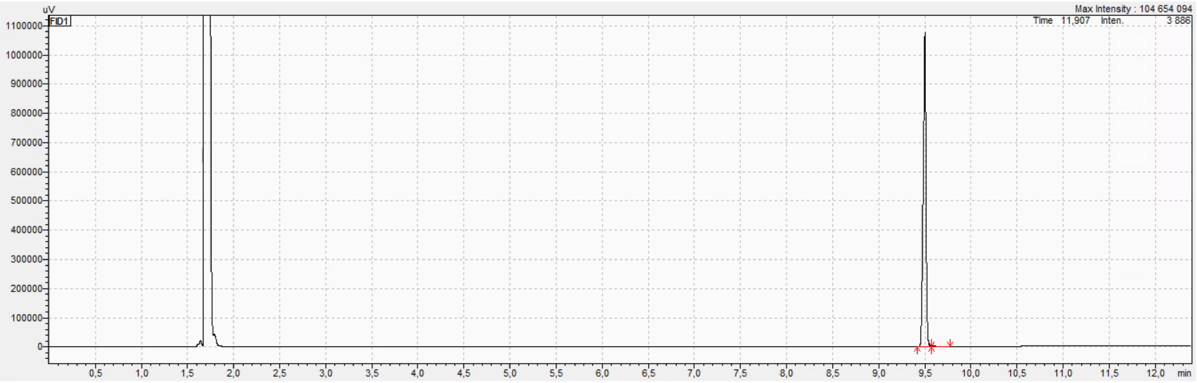

Reaction scale up

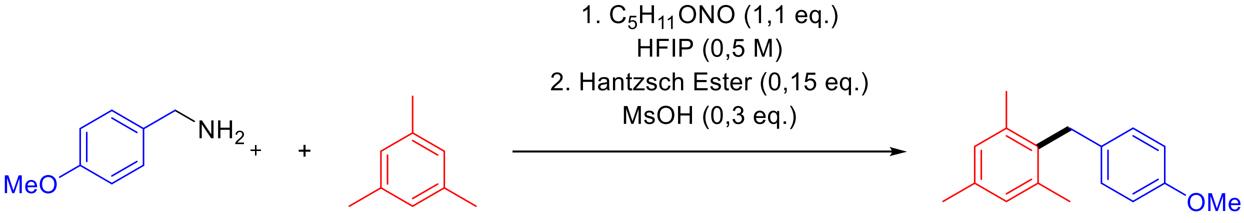

11.

10 mmol	5.0 eq.	2.38 g (99%), 17.9 mL (90%) HFIP recovered
10 mmol	2.5 eq.	2.24 g (93%), 17.7 mL (89%) HFIP recovered
200 mmol	2.5 eq.	45.19 g (94%), 356 mL (89%) HFIP recovered

10 mmol scale

A 50 mL round-bottom flask equipped with a magnetic bar was charged with 4-methoxybenzylamine (1.37 g, 10.0 mmol, 1.00 equiv.), mesitylene (7.0 mL, 50 mmol, 5.0 equiv.), HFIP (20 mL), and *iso*-pentyl nitrite (1.5 mL, 11 mmol, 1.1 equiv.). The reaction mixture was stirred at room temperature for 2 h. No significant increase in temperature was observed. Then, Hantzsch ester (380 mg, 1.50 mmol, 0.150 equiv.), and MsOH (195 μL, 3.00 mmol, 0.300 equiv.) were added. The reaction mixture was stirred for 1 h at room temperature. Then, triethylamine (0.42 mL, 3.0 mmol, 0.3 equiv.) was added to quench the reaction. The distillation kit was assembled and HFIP was distilled directly from the reaction flask, obtaining 17.9 mL (90%) of the solvent used. The rest of the reaction mixture was evaporated under high vacuum, removing most of the mesitylene. Methanol:water:concentared HCl solution (5:1.5:1, 7.5 mL) was added to the remaining oil and left in the cold to crystallize. The resulting crystals were filtered, washed with a water:methanol solution (1:1) and dried to afford 2.38 g of compound **11** as an off-white solid. **(Yield = 99%).**

The reaction with 2.5 equiv. (3.5 mL, 25 mmol) of mesitylene was performed similarly. The same workup allowed recovery of HFIP (17.7 mL, 89%) of used and afforded 2.38 g of off-white solid product, which was 94% pure (GC). To further purify, the solid was subjected to a short silica plug and was eluted with hexane to afford 2.24 g of pure, white product. **(Yield = 93%).**

200 mmol scale

A 1000 mL round-bottom flask equipped with a magnetic bar was charged with 4- methoxybenzylamine (27.4 g, 200 mmol, 1.0 equiv.), mesitylene (70 mL, 500 mmol, 2.5 equiv.), HFIP (400 mL, including the recycled from previous runs), and isopentyl nitrite (30 mL, 220 mmol, 1.1 equiv.). The reaction mixture was stirred at room temperature for 2 h. The flask was placed in a vessel of water to absorb the heat of the reaction, but this procedure did not appear to have been necessary. Then, Hantzsch ester (7.6 g, 30 mmol, 0.15 equiv.), and MsOH (3.9 mL, 60 mmol, 0.3 equiv.) were added. The reaction mixture was stirred for 1 h at room temperature. Then, triethylamine (8.4 mL, 60 mmol, 0.3 equiv.) was added to quench the reaction. The distillation kit was assembled and HFIP was distilled directly from the reaction flask, obtaining 356 mL (89%) of the solvent used. The rest of the reaction mixture was evaporated under high vacuum, removing most of the mesitylene. Methanol:water:concentared HCl solution (5:1.5:1, 150 mL) was added to the remaining oil and left in the cold to crystallize. The resulting crystals were filtered, washed with a water:methanol solution (1:1) and dried to afford 47.5 g of off-white solid product, which was 95% pure (GC). **(Yield = 94%).**

Other nucleophiles

4-Bromobenzyl chloride (74)[32]

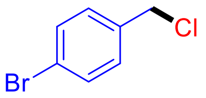

Following General Procedure B, compound **74** was obtained from 4-bromobenzylamine (223 mg, 1.00 mmol) using concentrated hydrochloric acid (36%, 258 μL, 3.00 mmol, 3.00 equiv.) as both a chloride source and an acid, instead of an arene and MsOH. HCl was added after diazotization and the reaction mixture was stirred at 60 °C for 16 h in a screw-cap vial. Purified by column chromatography (petroleum ether) to afford 189 mg of compound **74** as a white solid. **(Yield = 92%).**

^{1}H NMR (400 MHz, $CDCl_3$) δ 7.51 – 7.45 (m, 2H), 7.29 – 7.23 (m, 2H), 4.53 (s, 2H).

^{13}C NMR (100 MHz, $CDCl_3$) δ 136.5, 131.9, 130.2, 122.5, 45.4.

4-Bromobenzyl bromide (75)[33]

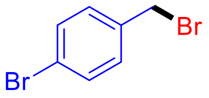

Following General Procedure B, compound **75** was obtained from 4-bromobenzylamine (223 mg, 1.00 mmol) using concentrated hydrobromic acid (48%, 339 μL, 3.00 mmol, 3.00 equiv) as both bromide source and an acid, instead of an arene and MsOH. HBr was added after diazotization and the reaction mixture was stirred at 60 °C for 16 h in a screw-cap vial. Purified by column chromatography (petroleum ether) to afford 247 mg of compound **75** as a white solid. **(Yield = 99%).**

^{1}H NMR (400 MHz, $CDCl_3$) δ 7.51 – 7.43 (m, 2H), 7.30 – 7.23 (m, 2H), 4.43 (s, 2H).

^{13}C NMR (100 MHz, $CDCl_3$) δ 136.8, 132.0, 130.7, 122.5, 32.4.

2-(4-Chlorophenyl)ethyl chloride (76)[34]

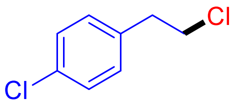

Following General Procedure D, compound **76** was obtained from 4-chlorophenethylamine (156 mg, 1.00 mmol) using concentrated hydrochloric acid (36%, 64 μL, 0.75 mmol, 0.75

equiv.) and tetraethylammonium chloride (829 mg, 5.00 mmol, 5.00 equiv.) as chloride sources, instead of an arene, with HFIP (5 mL). Purified by column chromatography (petroleum ether) to afford 135 mg of compound **76** as colourless oil. **(Yield = 77%).**

^{1}H NMR (400 MHz, CDCl$_3$) δ 7.34 – 7.25 (m, 2H), 7.20 – 7.01 (m, 2H), 3.69 (t, *J* = 7.2 Hz, 2H), 3.04 (t, *J* = 7.2 Hz, 2H).

^{13}C NMR (100 MHz, CDCl$_3$) δ 136.5, 132.8, 130.2, 128.7, 44.7, 38.4.

2-(4-Chlorophenyl)ethyl bromide (77)[35]

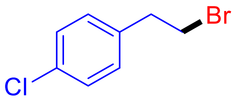

Following General Procedure D, compound **77** was obtained from 4-chlorophenethylamine (156 mg, 1.00 mmol) using concentrated hydrobromic acid (48%, 85 μL, 0.75 mmol, 0.75 equiv.) and tetrabutylammonium bromide (1612 mg, 5.00 mmol, 5.00 equiv.) as bromide sources, instead of an arene, with HFIP (5 mL). Purified by column chromatography (petroleum ether) to afford 184 mg of compound **77** as colourless oil. **(Yield = 84%).**

^{1}H NMR (400 MHz, CDCl$_3$) δ 7.33 – 7.22 (m, 2H), 7.22 – 7.11 (m, 2H), 3.54 (t, *J* = 7.4 Hz, 2H), 3.13 (t, *J* = 7.4 Hz, 2H).

^{13}C NMR (100 MHz, CDCl$_3$) δ 137.3, 132.8, 130.0, 128.7, 38.6, 32.6.

3-(1-Adamantyl)pentane-2,4-dione (78)[36]

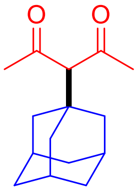

Following General Procedure B, compound **78** was obtained from 1-adamantylamine (151 mg, 1.00 mmol) and acetylacetone (501 mg, 5.00 mmol), used instead of an arene, added after diazotization. The reaction mixture was stirred with MsOH at room temperature for 16 h. Purified by column chromatography (AcOEt/hexanes gradually from 0:100 to 5:95) to afford 206 mg of compound **78** as a white solid. **(Yield = 88%).**

^{1}H NMR (400 MHz, CDCl$_3$) δ 3.47 (s, 1H), 2.13 (s, 6H), 1.91 (s, 3H), 1.70 – 1.52 (m, 12H).

^{13}C NMR (100 MHz, CDCl$_3$) δ 204.3, 78.1, 40.3, 38.4, 36.5, 33.0, 28.5.

Ethyl 2-(adamantan-1-yl)-3-oxobutanoate (79)[37]

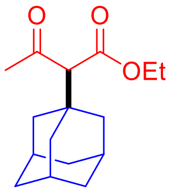

Following General Procedure B, compound **79** was obtained from 1-adamantylamine (151 mg, 1.00 mmol) and ethyl acetoacetate (651 mg, 5.00 mmol), used instead of an arene, added after diazotization. The reaction mixture was stirred with MsOH at room temperature for a week. Purified by column chromatography (AcOEt/hexanes gradually from 0:100 to 5:95) to afford 219 mg of compound **79** as yellowish oil. **(Yield = 83%).**

^{1}H NMR (400 MHz, $CDCl_3$) δ 4.14 (q, *J* = 7.2 Hz, 2H), 3.16 (s, 1H), 2.20 (s, 3H), 1.99 – 1.91 (m, 3H), 1.81 – 1.57 (m, 12H), 1.24 (t, *J* = 7.1 Hz, 3H).

^{13}C NMR (100 MHz, $CDCl_3$) δ 203.2, 168.6, 69.9, 60.7, 40.0, 36.9, 36.6, 32.1, 28.6, 14.2.

1-Adamantanecarboxylic acid (80)[38]

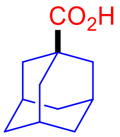

A 10 mL vial equipped with a magnetic bar was charged with 1-adamantylamine (151 mg, 1.00 mmol), HFIP (5.0 mL), and isopentyl nitrite (148 µL, 1.1 mmol, 1.1 equiv.). The reaction mixture was stirred at room temperature for 2 h. Then, Hantzsch ester (38 mg, 0.15 mmol, 0.15 equiv.), formic acid (189 µL, 5.00 mmol, 5.00 equiv.) and H_2SO_4 (98%, 820 µL, 15.0 mmol, 15.0 equiv.) were added. The vial was sealed with a cup and the reaction mixture was stirred at room temperature for 16 h. Then, the reaction mixture was transfered to 100 mL round bottom flask and stirred with 25 mL 2 M aqueous NaOH solution for 16 h. The mixture was then acidified with 2 M HCl, and extracted with 3 x 25 mL DCM. The organic phase was dried with Na_2SO_4, filtered, and evaporated to afford 180 mg of compound **80** as a yellowish solid. **(Yield = 100%).**

^{1}H NMR (400 MHz, $CDCl_3$) δ 11.90 (s, 1H), 2.06 – 1.97 (m, 3H), 1.94 – 1.86 (m, 6H), 1.78 – 1.65 (m, 6H).

^{13}C NMR (100 MHz, $CDCl_3$) δ 184.5, 40.5, 38.6, 36.4, 27.8.

5. Mechanistic studies

5.1 Kinetic studies

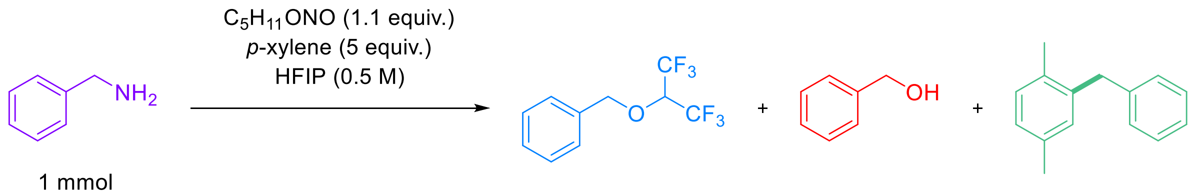

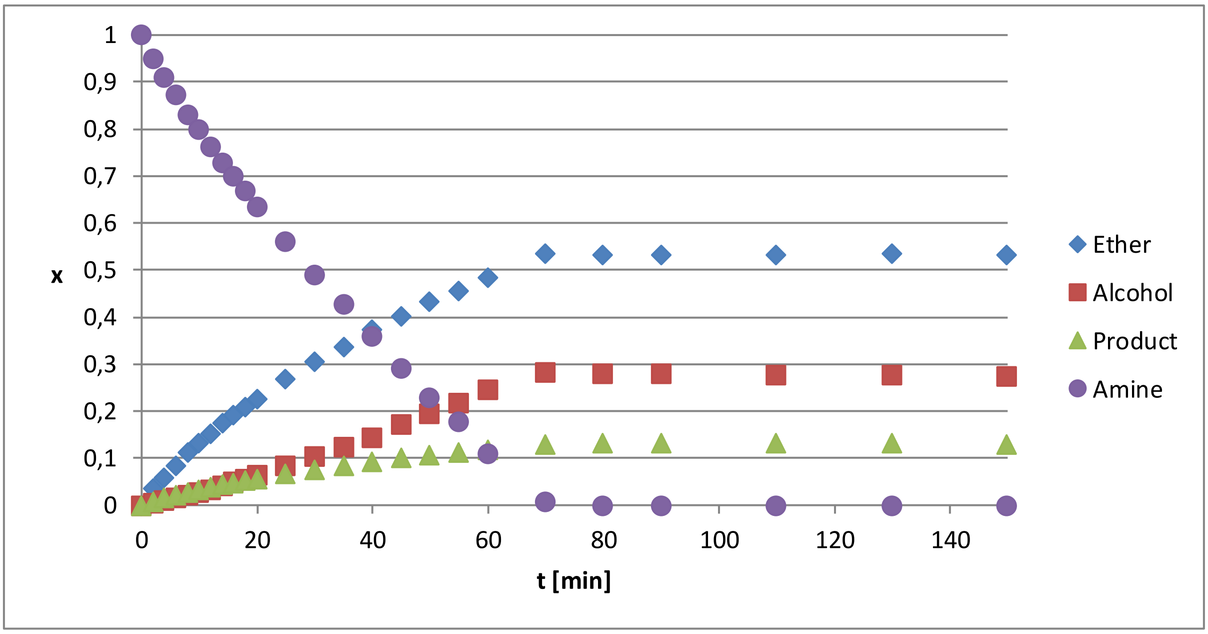

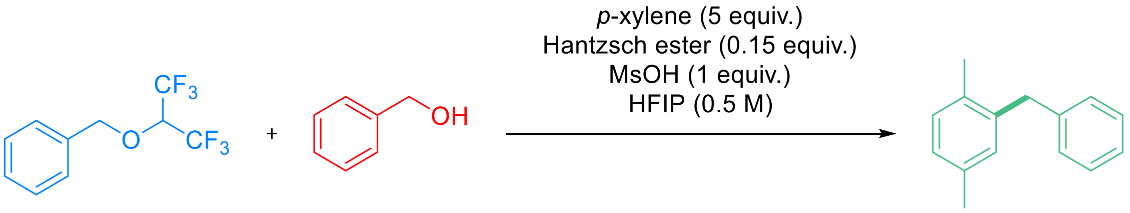

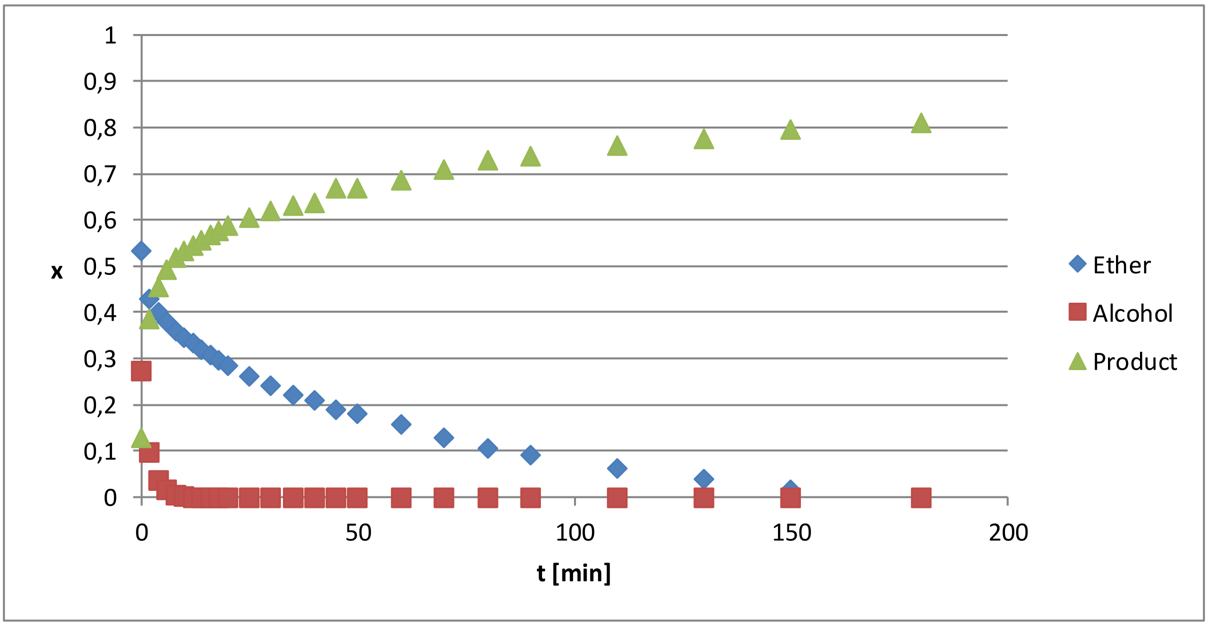

The kinetic profile of the diazotization shows that hexafluoro *iso*-propyl ether and the alkylated xylene are produced proportionally over time, while the rate of the benzyl alcohol formation slightly accelerates as the concentration of water increases during the reaction course. The resulting products are stable in the reaction medium. Thus, to push forward the Friedel-Crafts alkylation with transient ethers, the addition of an acid seemed required. Kinetic studies revealed that electrophilic aromatic substitution is much faster for alcohol than for electron deficient hexafluoro isopropyl ether. This fact explains why, in the case of some substrates, the use of acetates obtained in the diazotization reaction with the addition of acetic acid proved beneficial over ethers formed in the typical reaction carried out in HFIP.

5.2 Influence of the acidity of alcoholic solvent

Entry	Alcoholic solvent	pK_a	Conversion of the amine
1	EtOH	15.9	None
2	TFE	12.5	Traces
3	HFIP	9.3	Full

Reaction conditions: 1) $BnNH_2$ (1 mmol), p-xylene (5 equiv.), $C_5H_{11}ONO$ (1.1 equiv.), alcohol (c = 0.5 M), 2 h, RT.

These experiments prove that the appropriate acidity of the solvent is necessary for the diazotization process to occur.

6. NMR spectra

2-Benzylmesitylene (**10**)

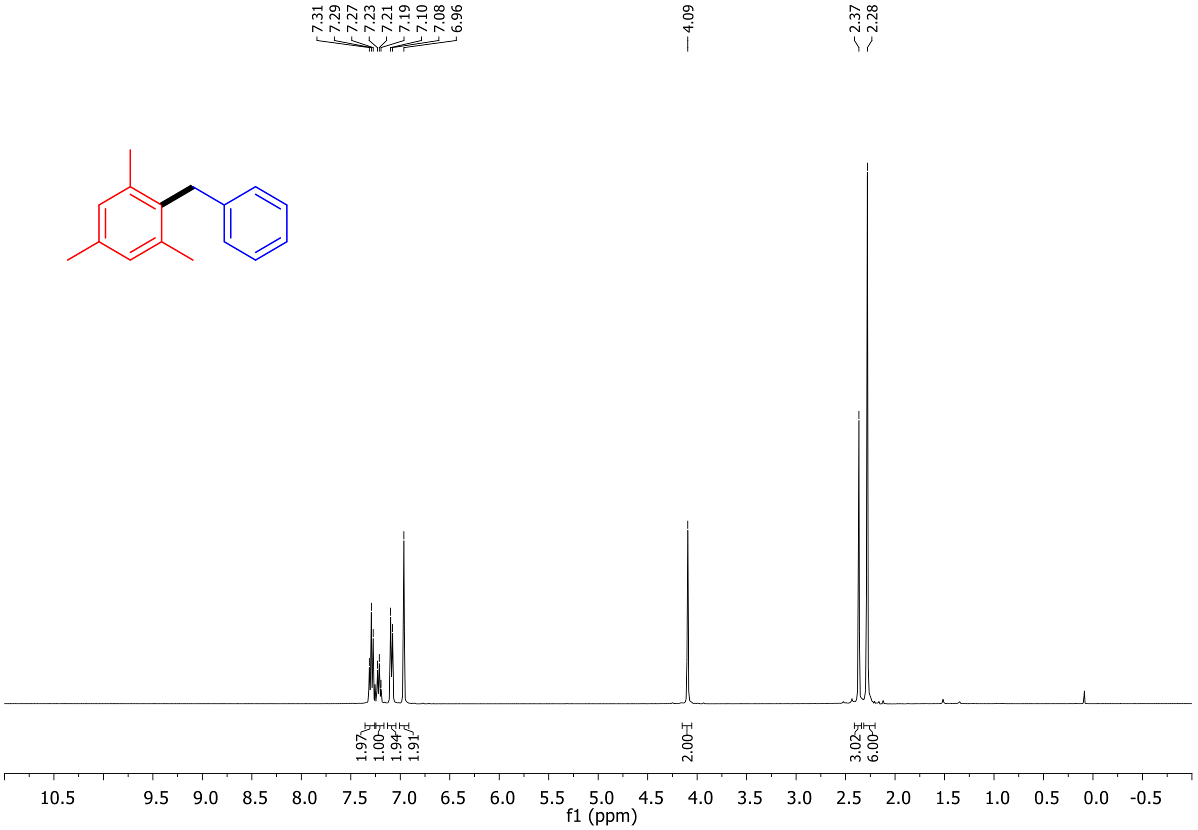

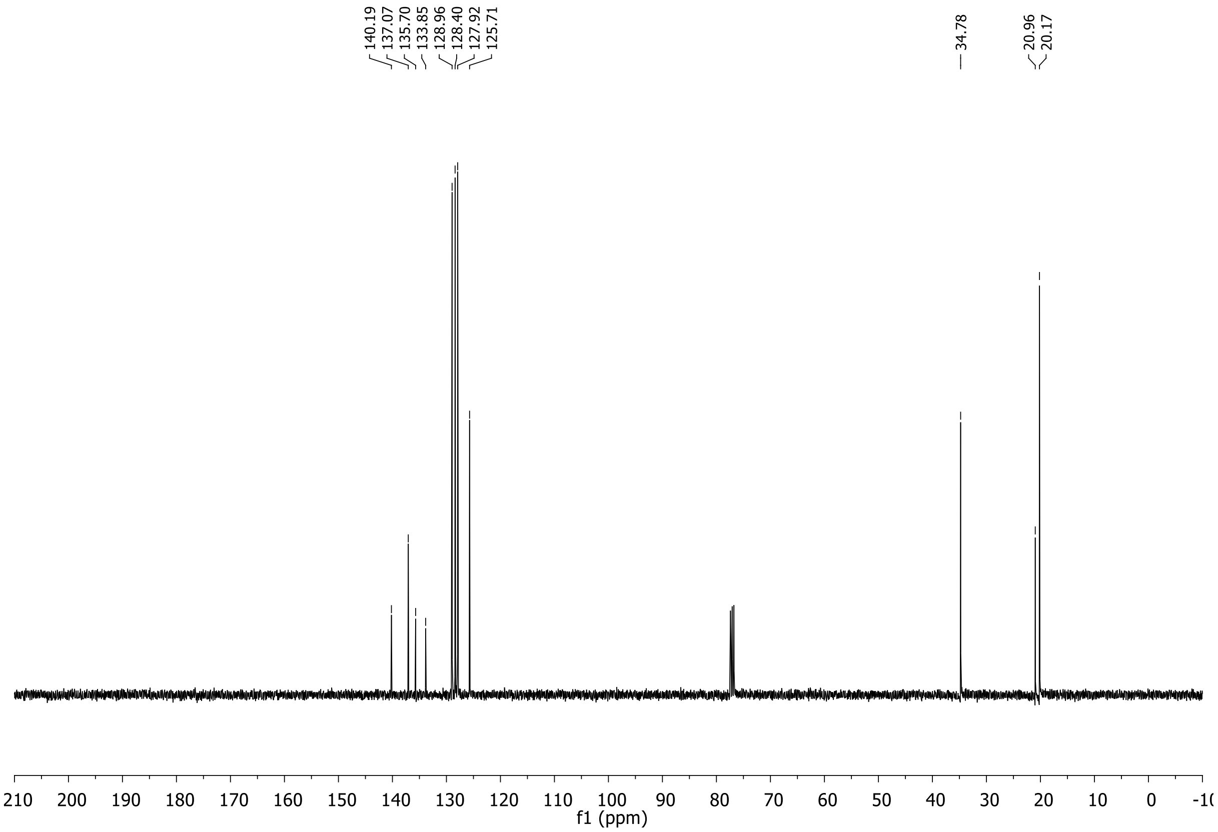

2-(4-Methoxybenzyl)mesitylene (**11**)

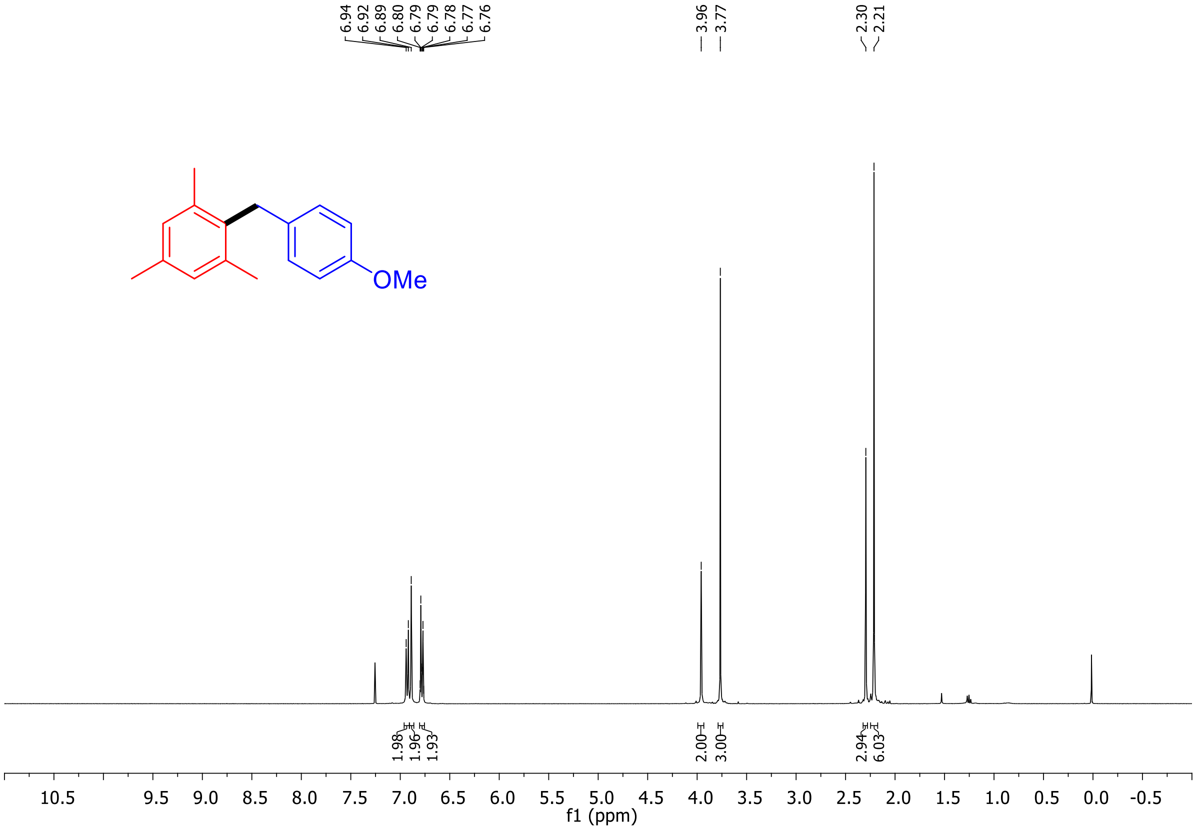

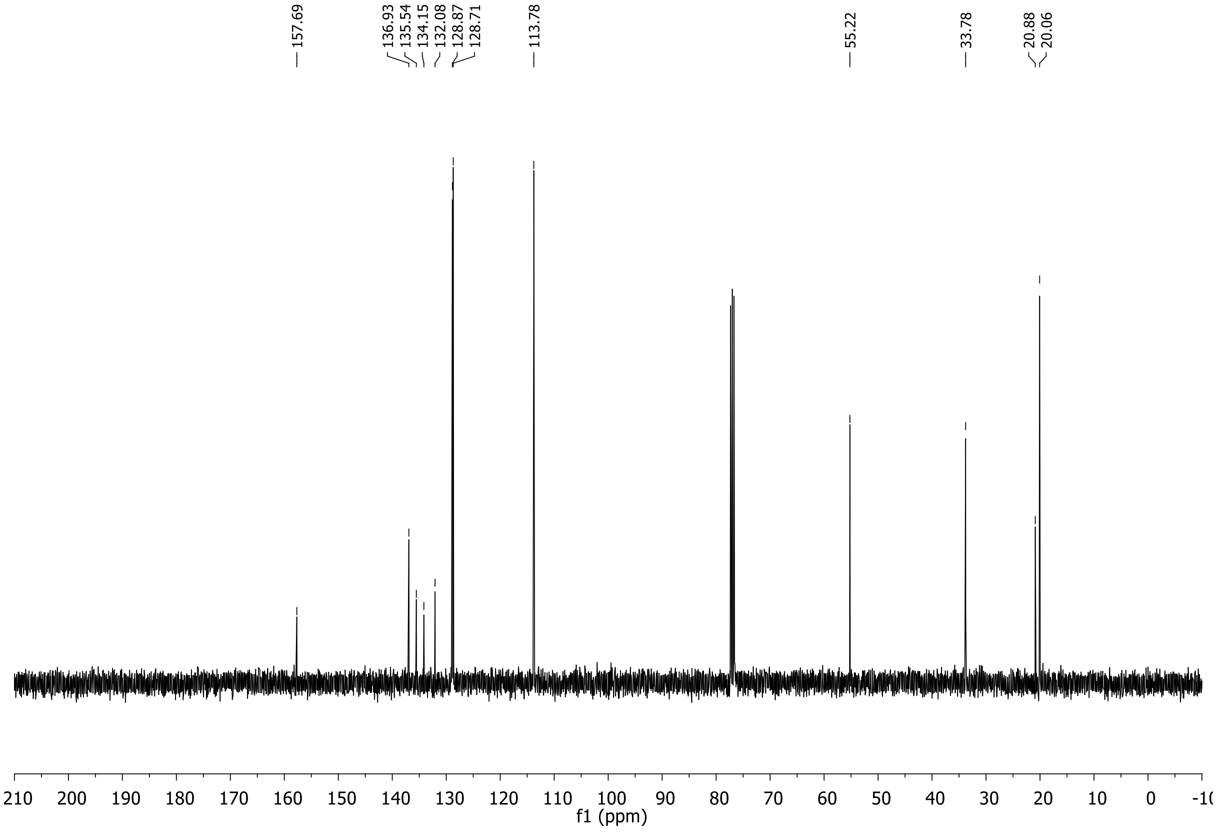

2-(4-Methylbenzyl)mesitylene (**12**)

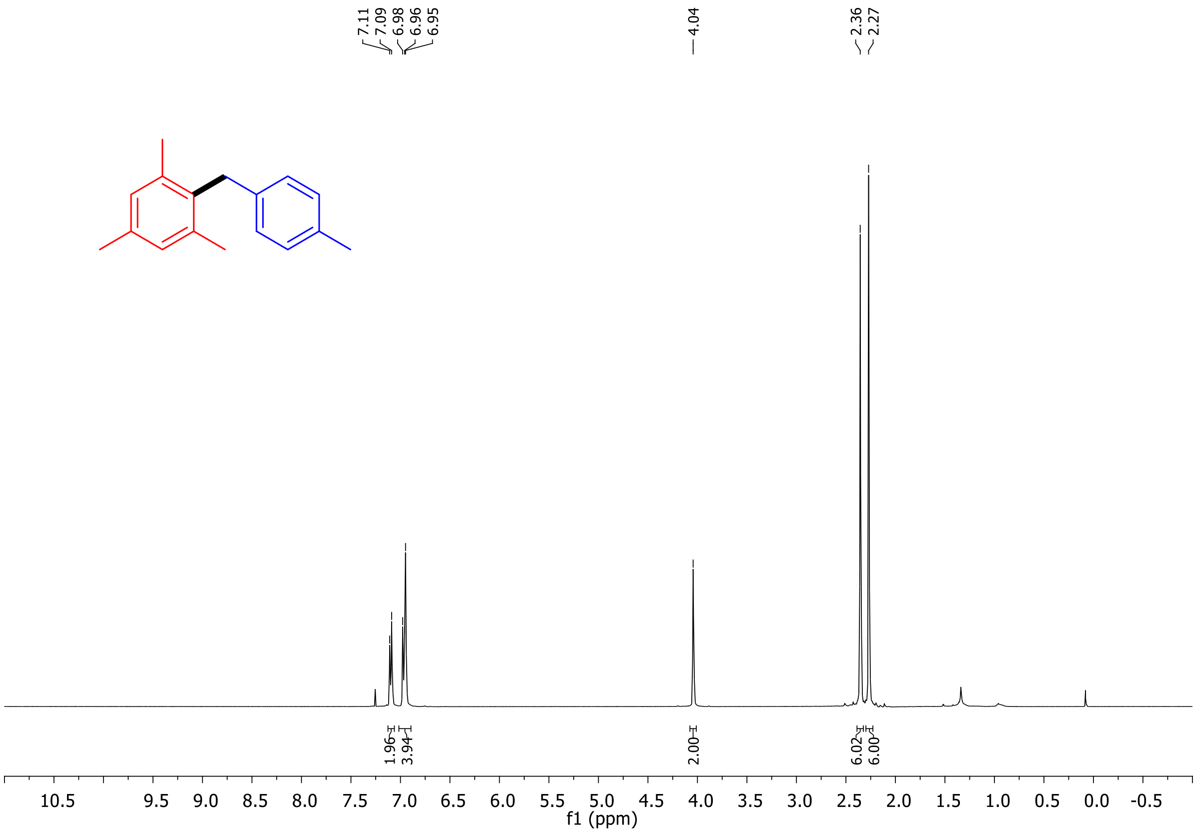

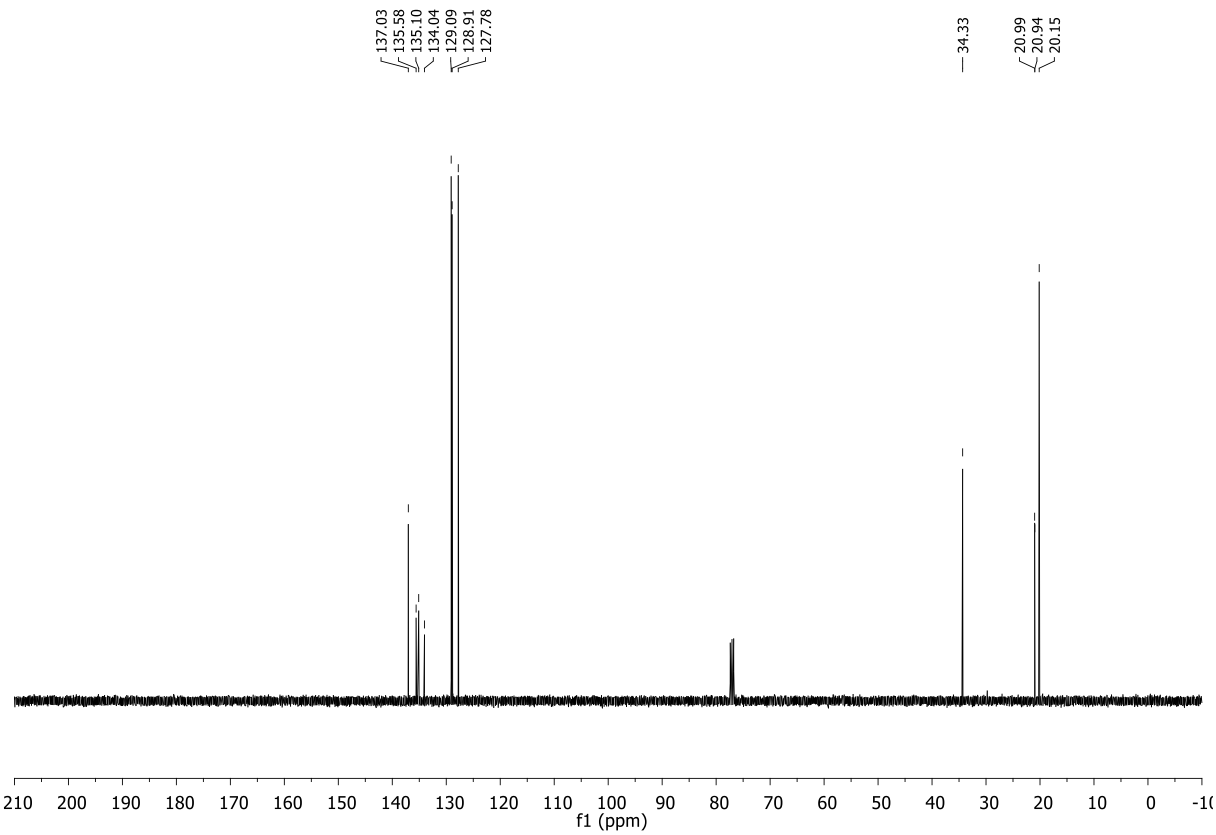

2-(4-*tert*-Butylbenzyl)mesitylene (**13**)

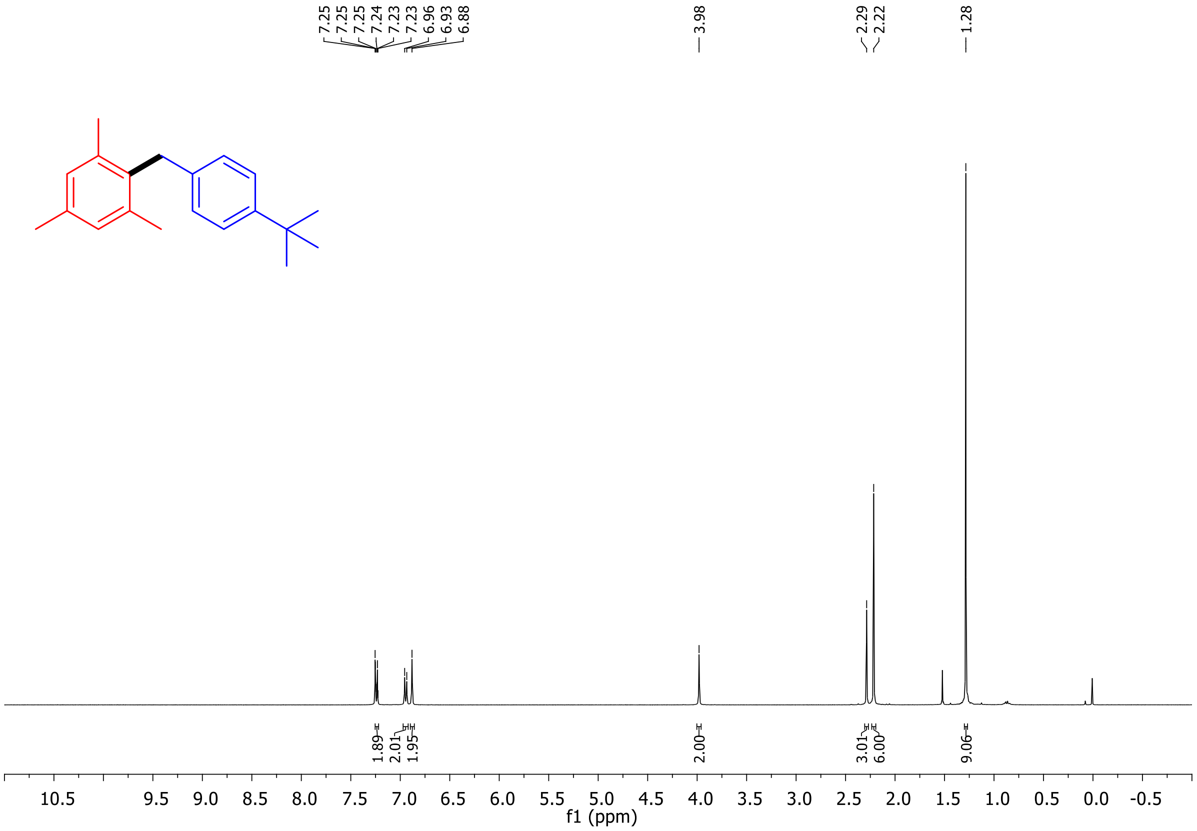

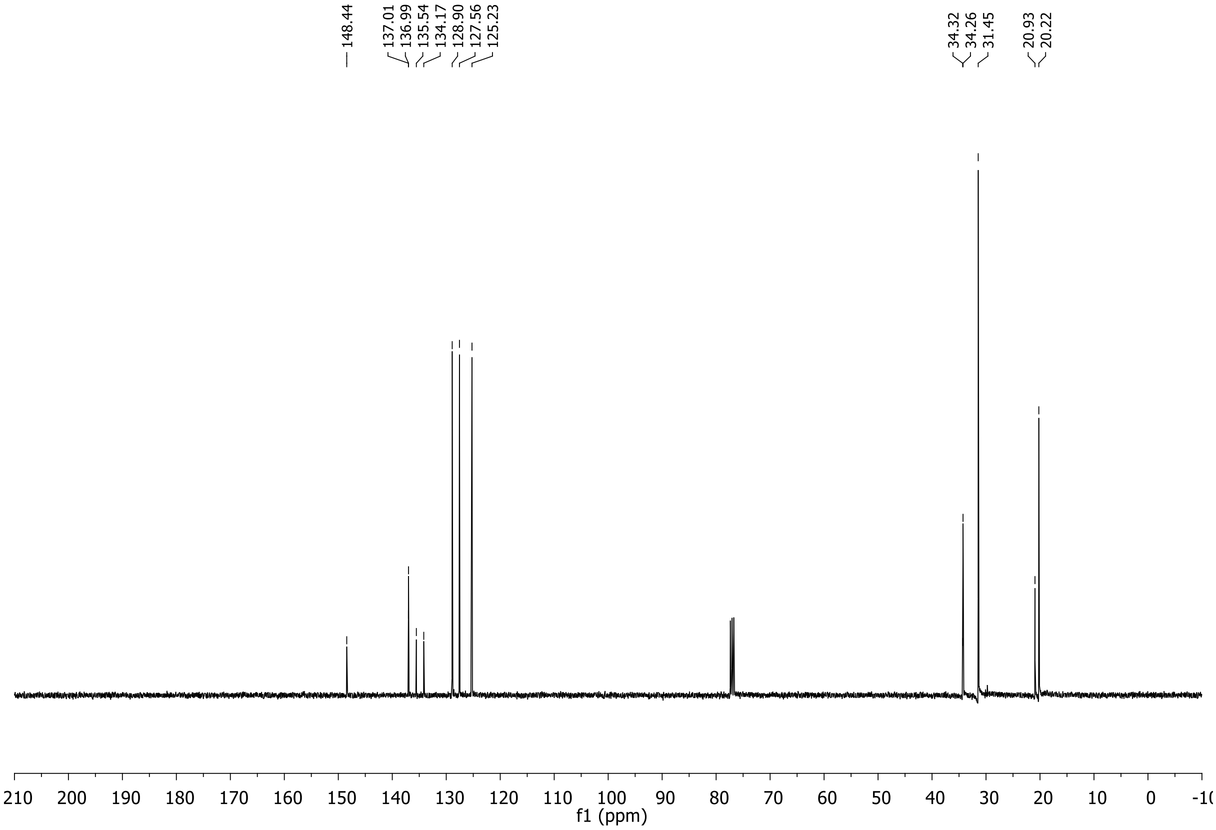

2-(4-Fluorobenzyl)mesitylene (**14**)

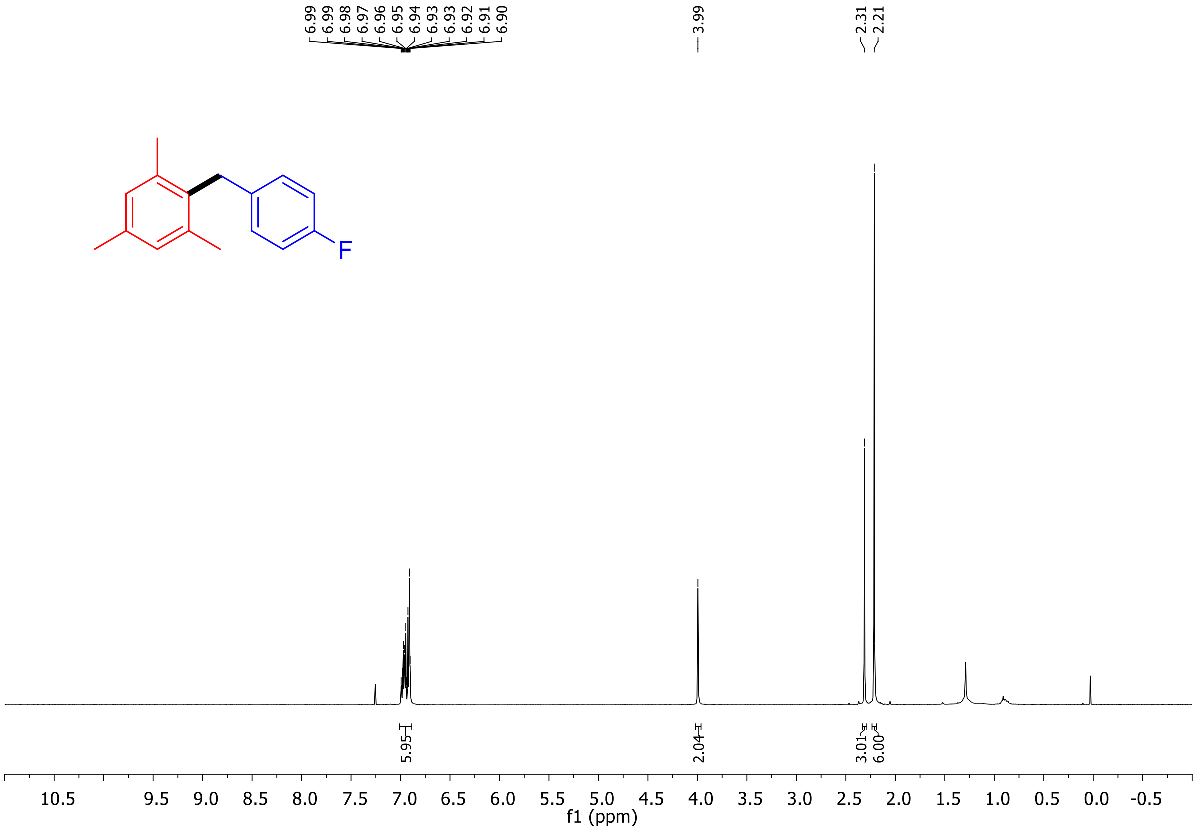

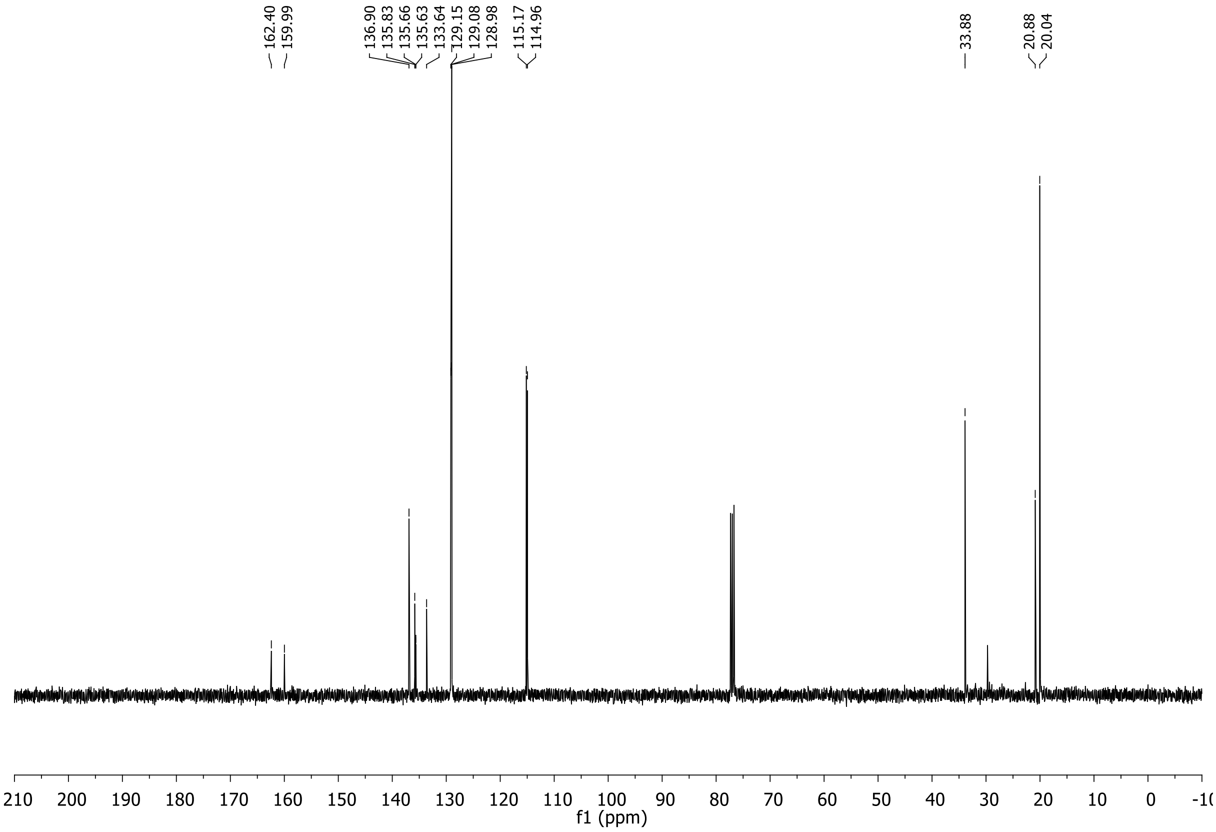

2-(4-Bromobenzyl)mesitylene (**15**)

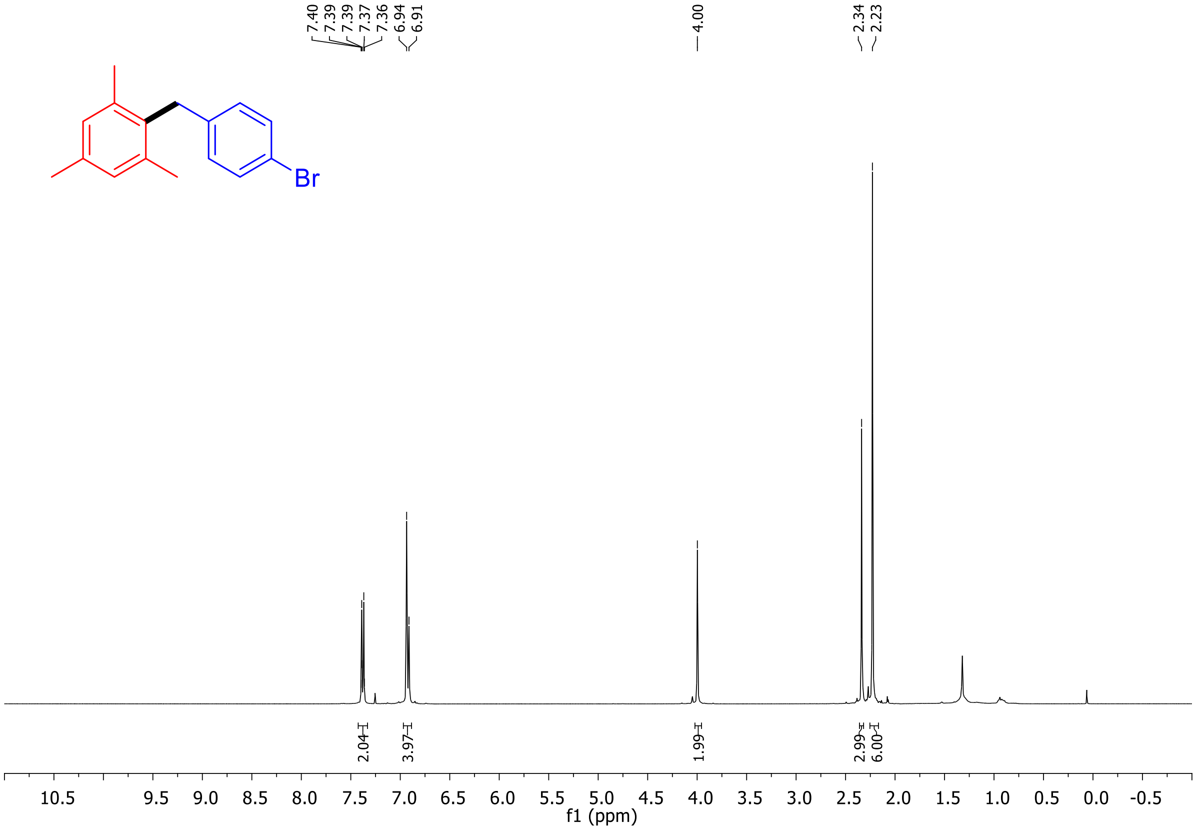

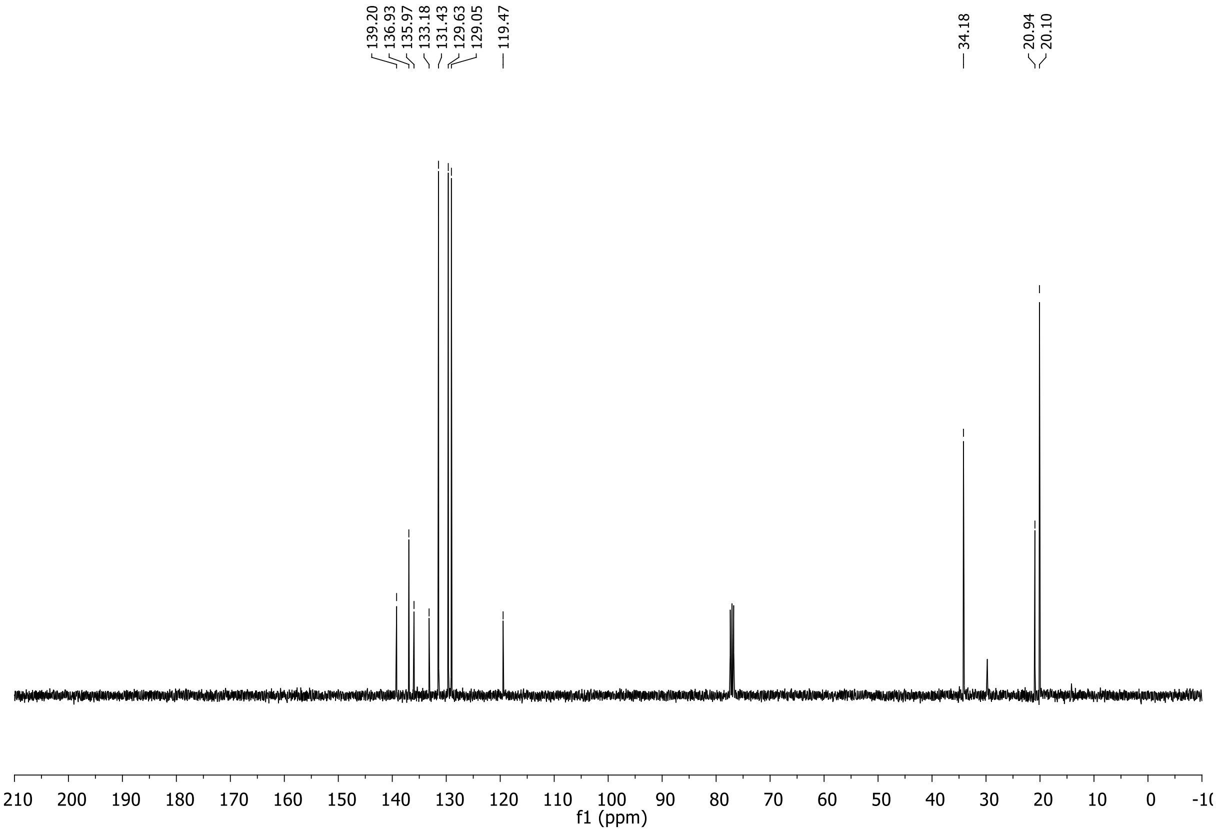

2-(4-Trifluoromethylbenzyl)mesitylene (**16**)

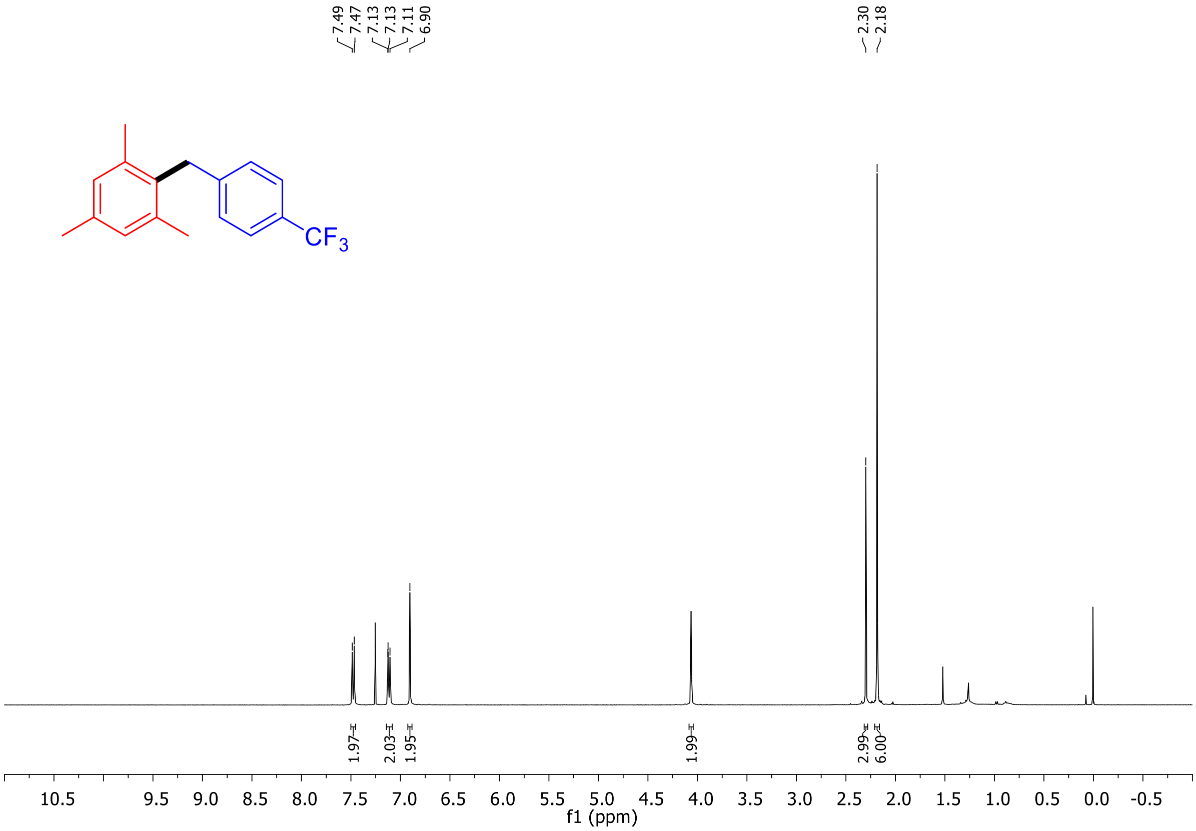

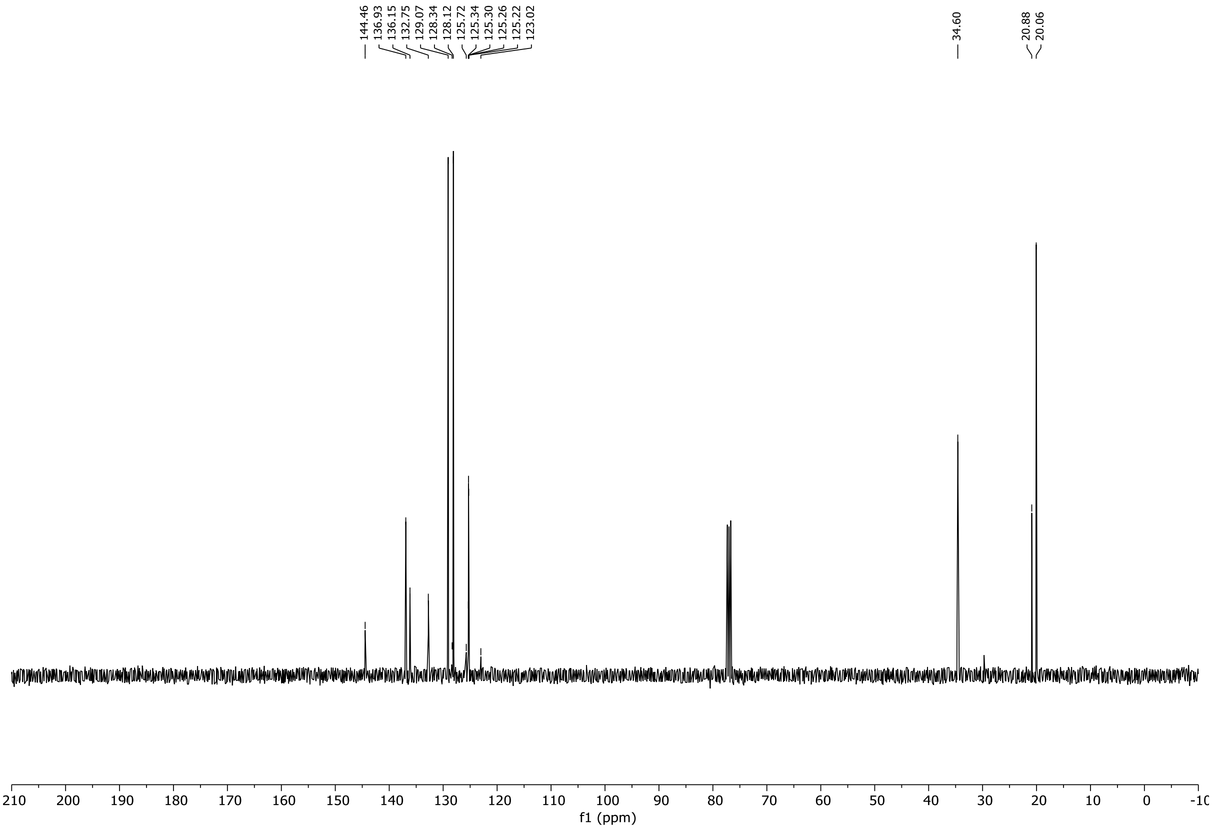

2-(4-Nitrobenzyl)mesitylene (**17**)

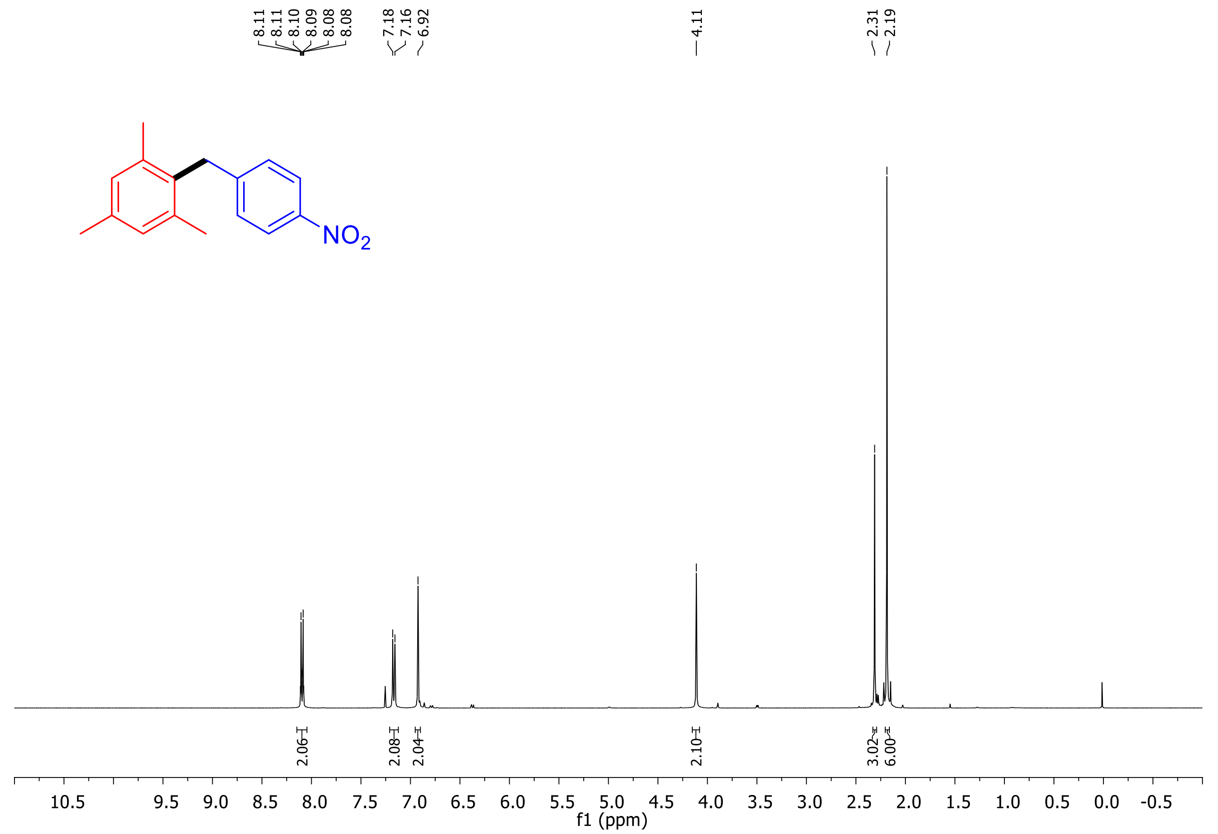

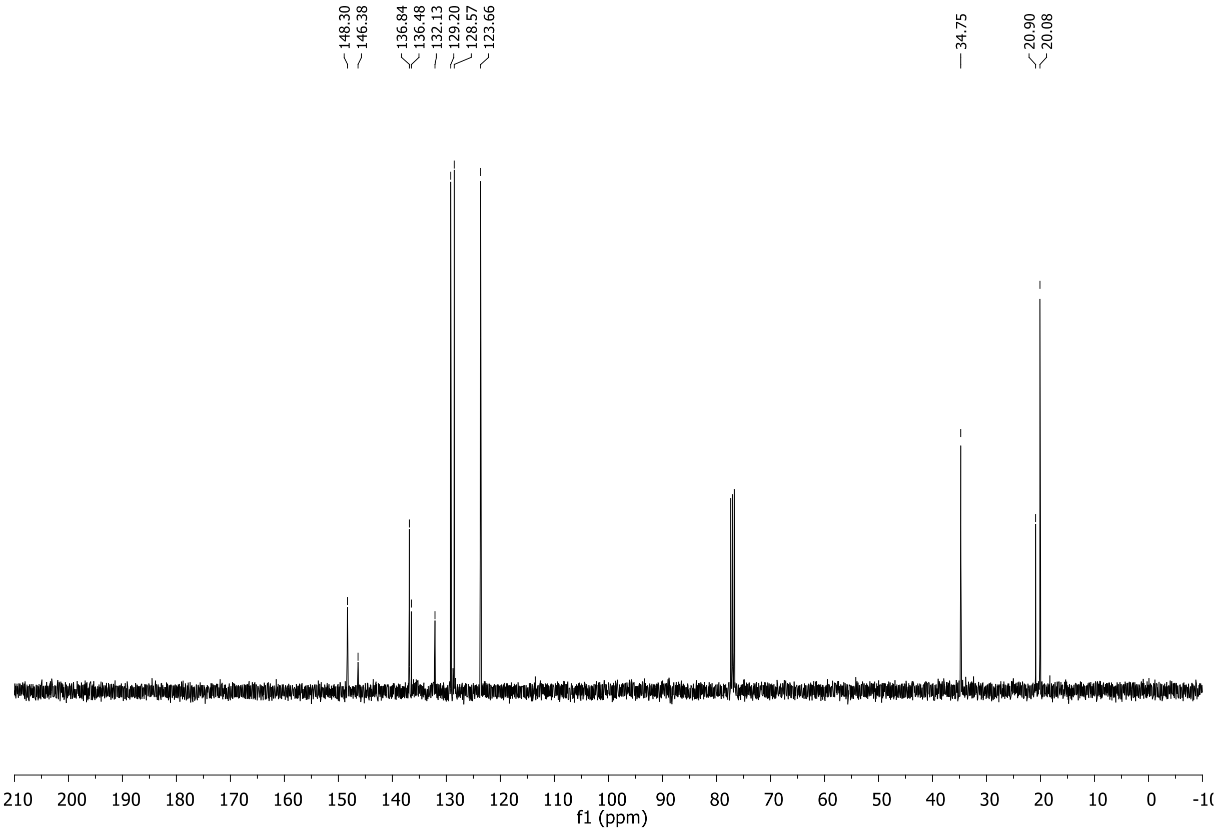

2-(2-Methylbenzyl)mesitylene (**18**)

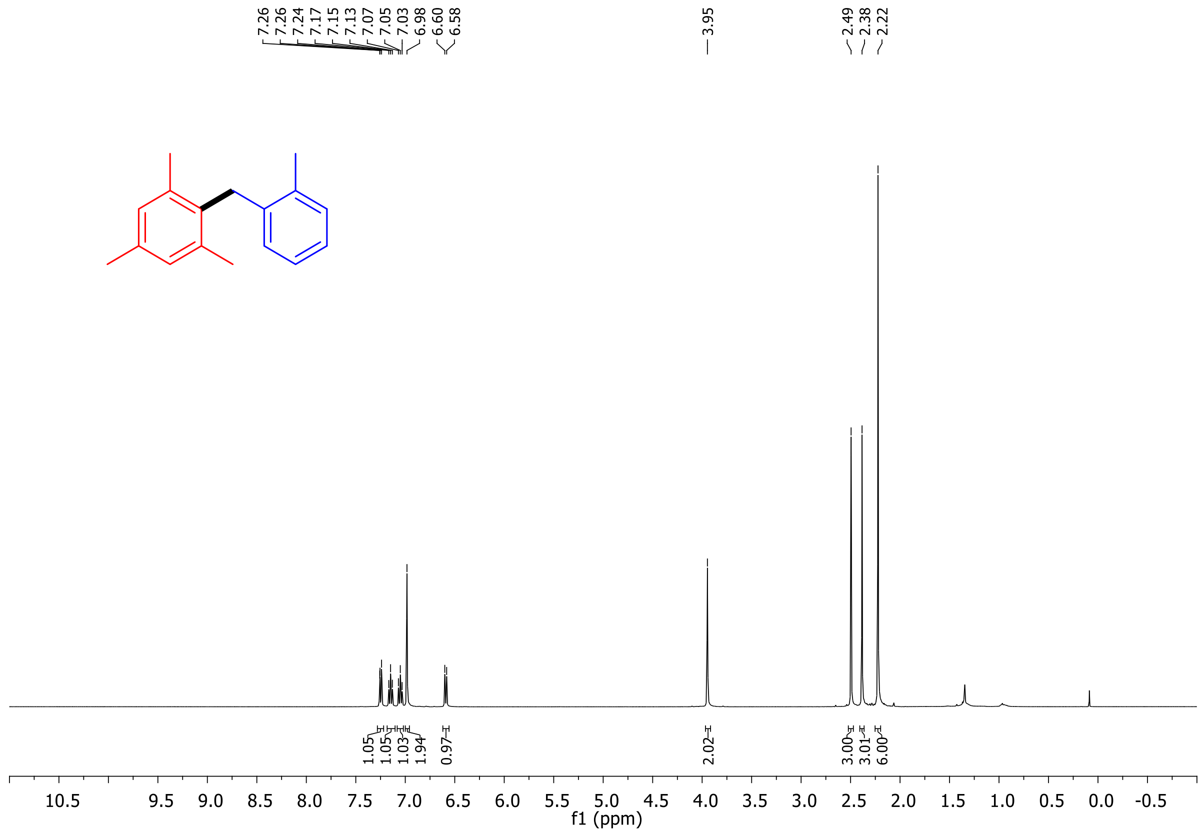

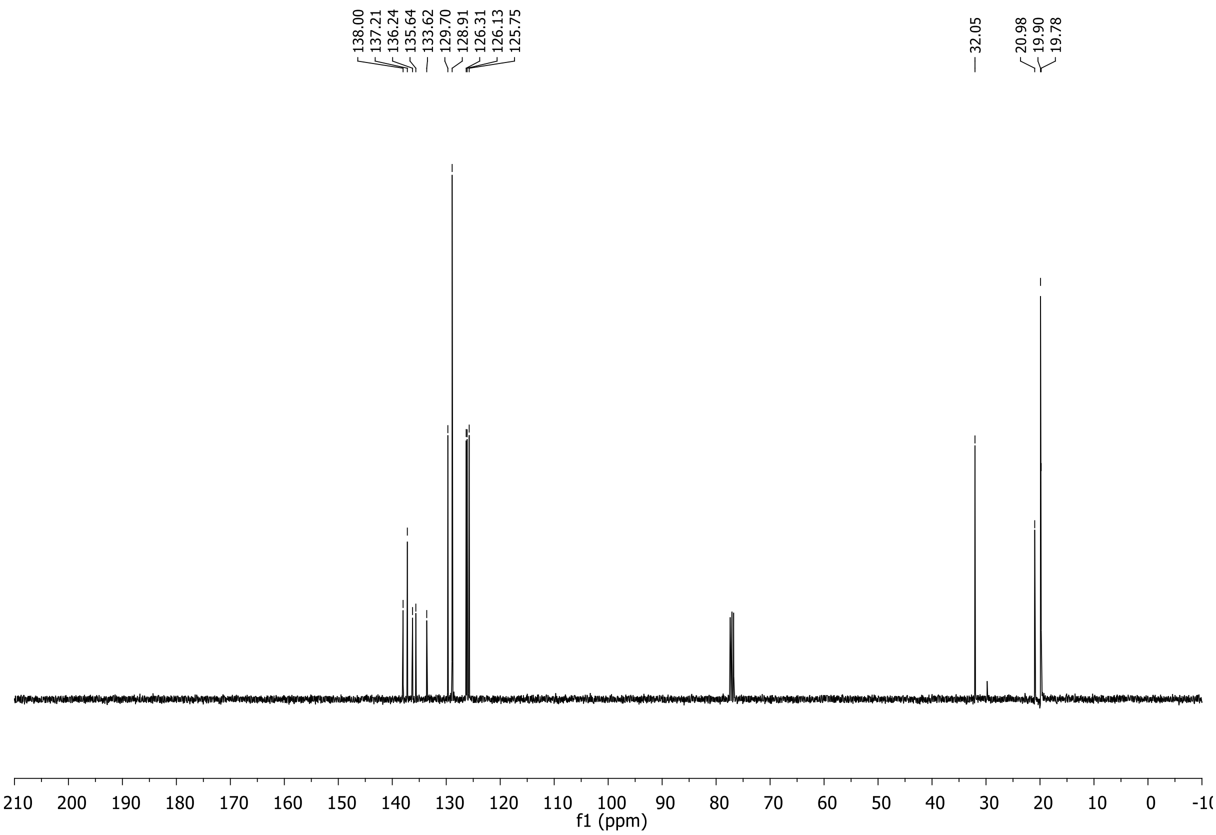

2-(Naphthalen-1-ylmethyl)mesitylene (**19**)

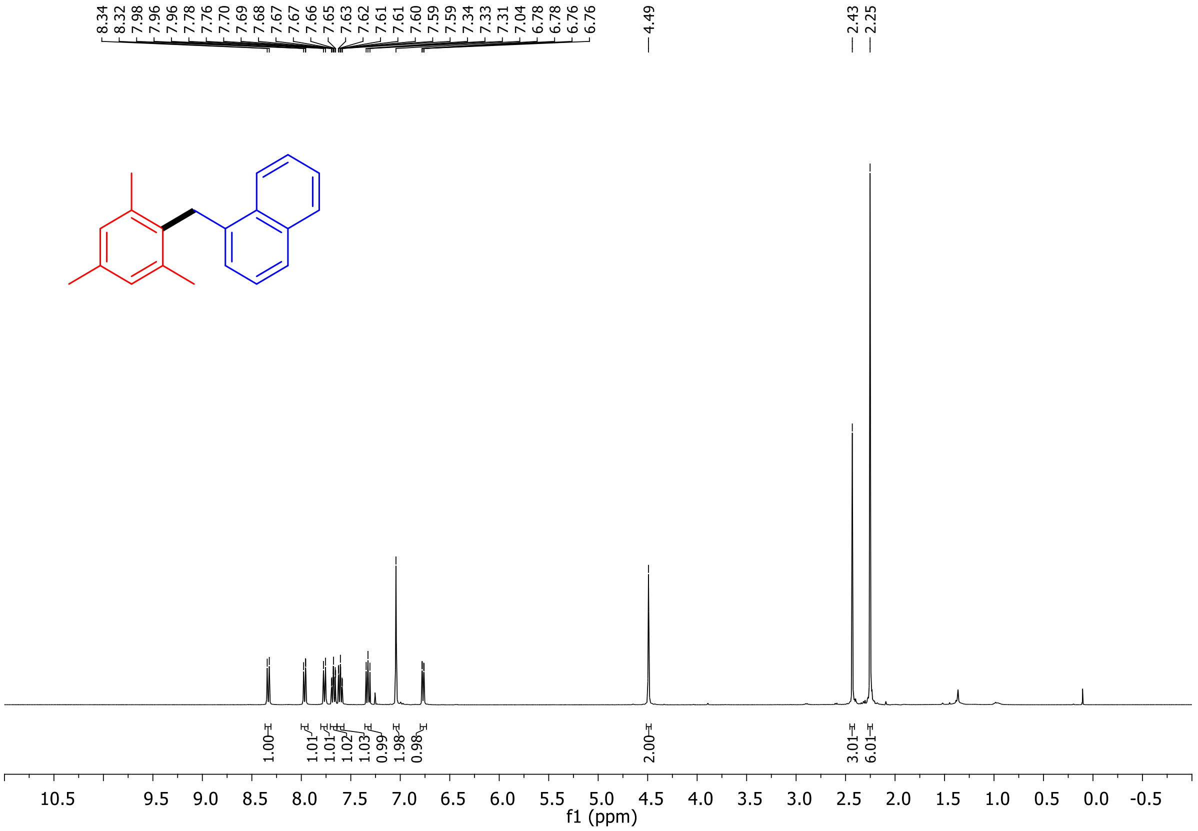

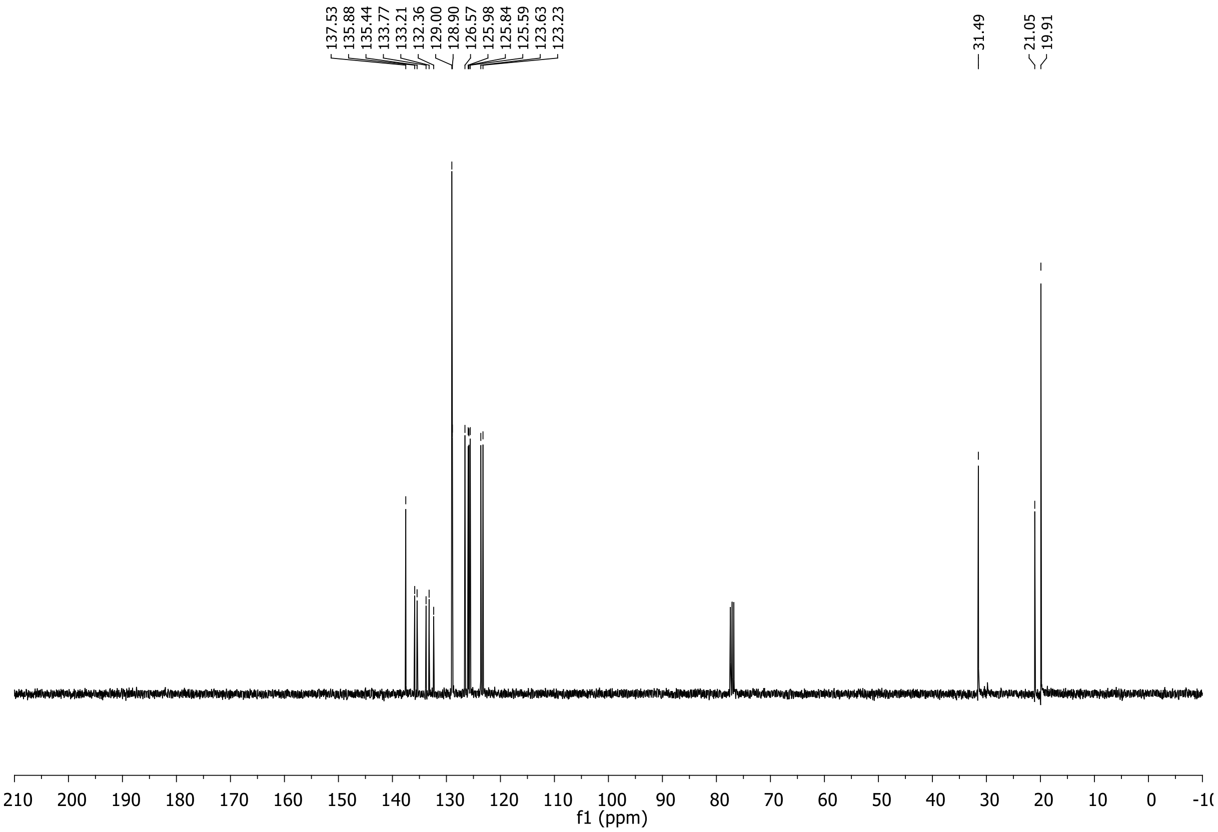

2-Benzhydrylmesitylene (**20**)

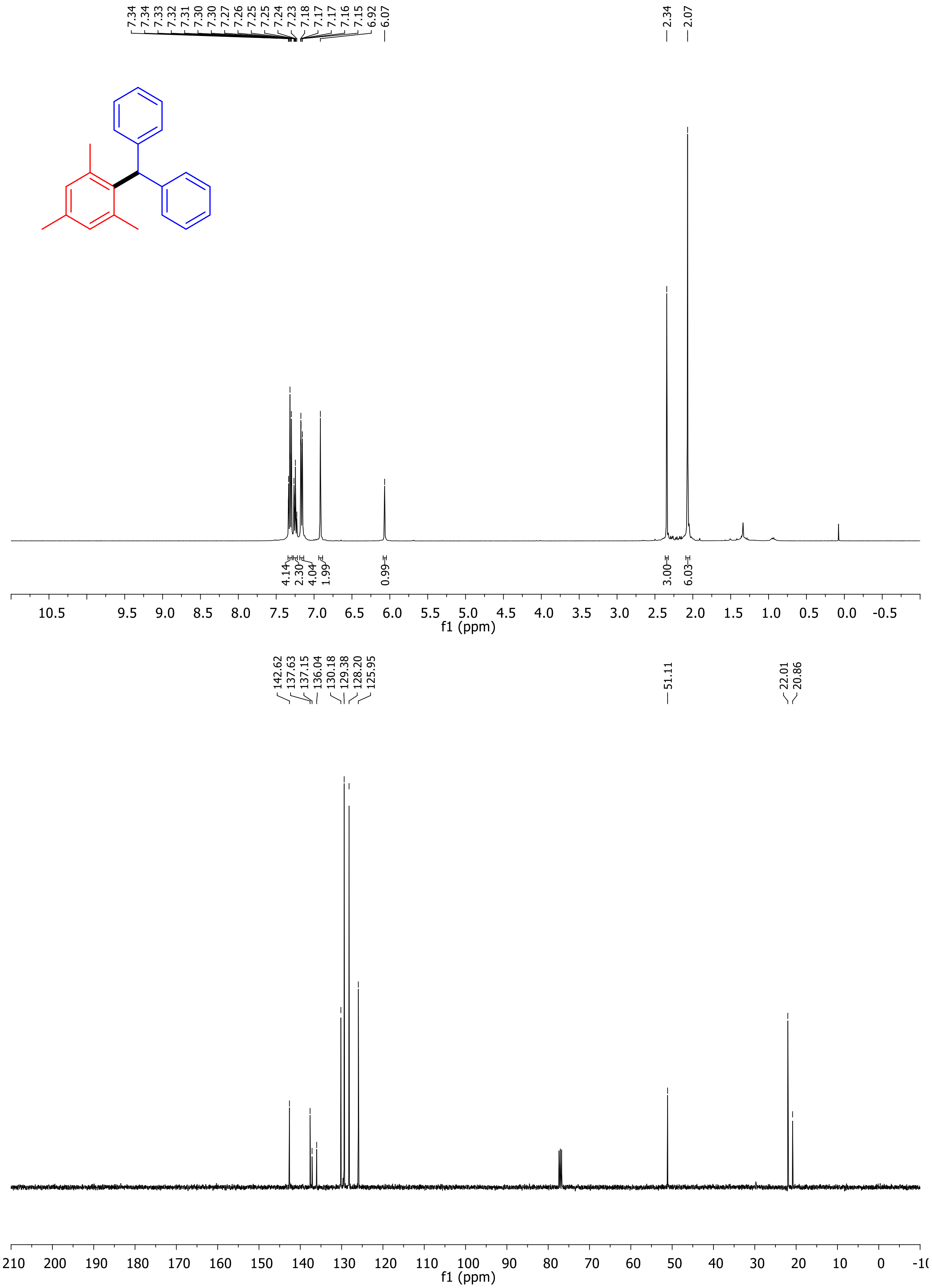

2-(3,4-Dimethoxybenzyl)mesitylene (**21**)

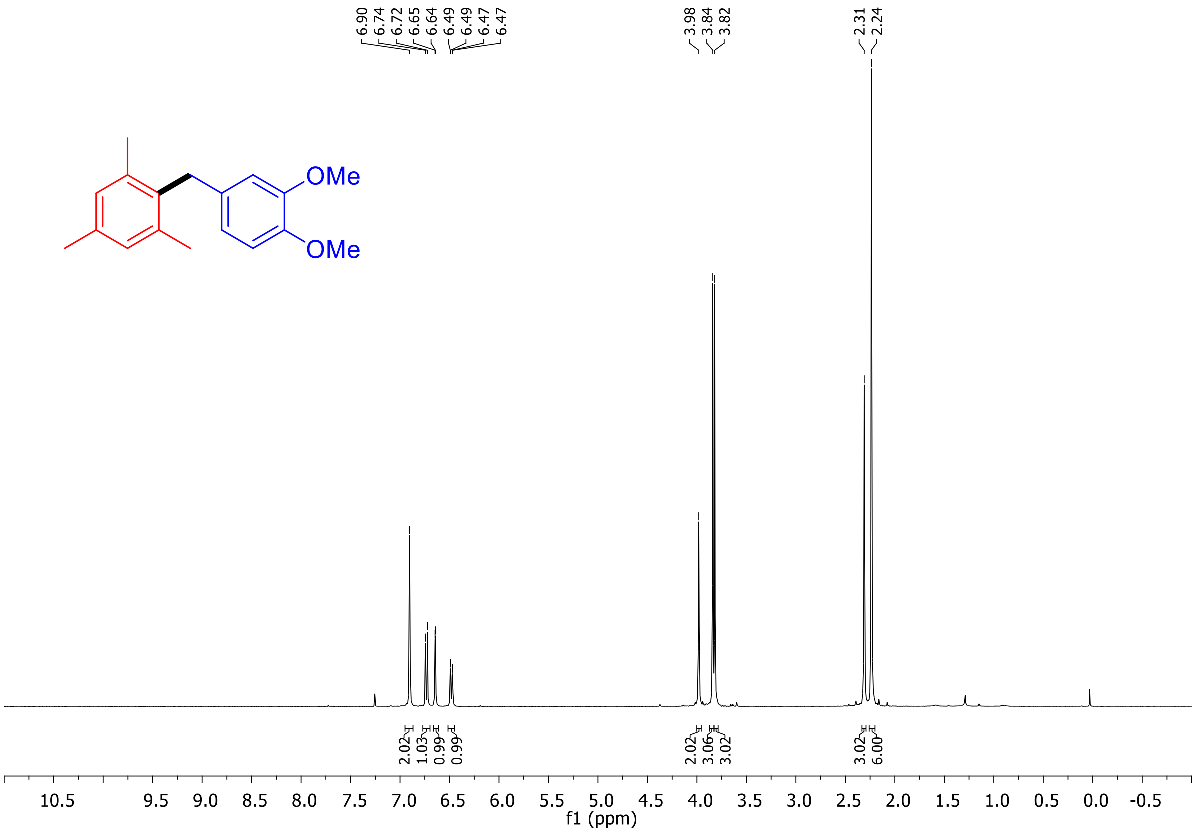

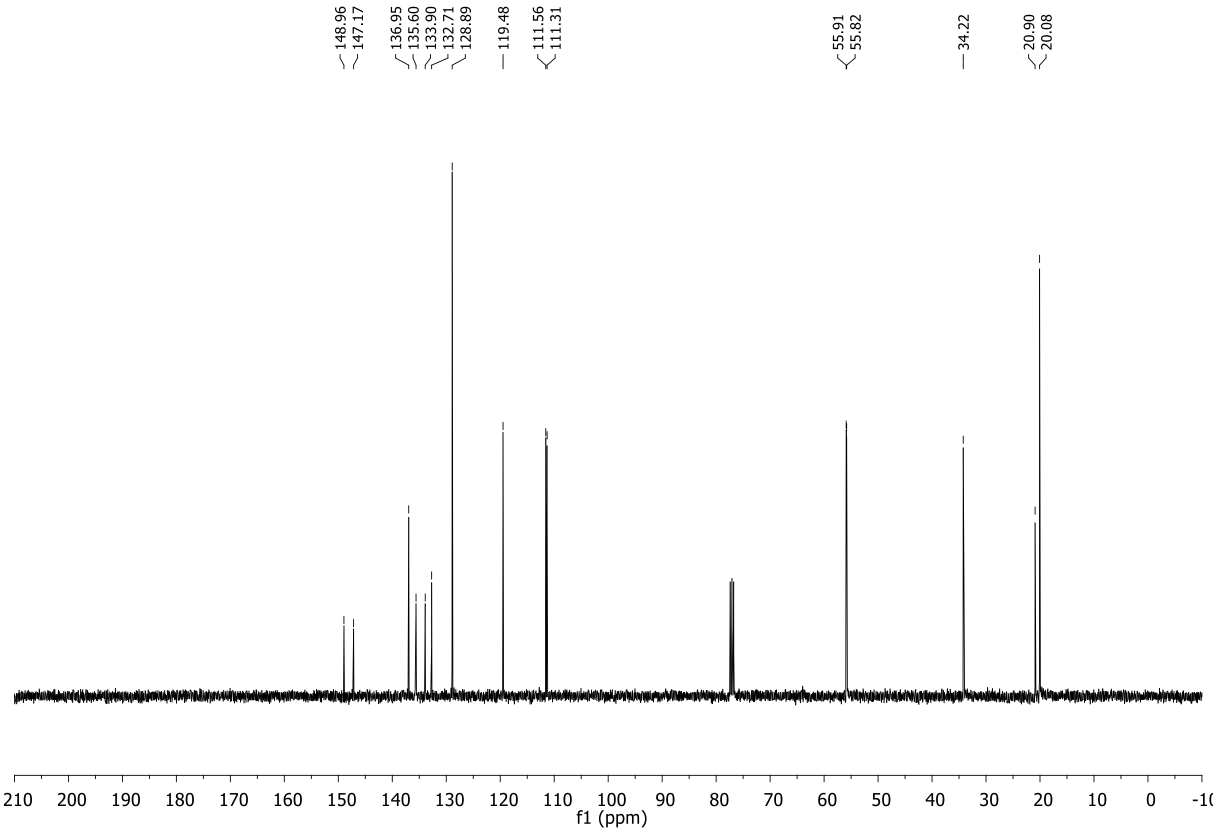

1,3-Bis(2,4,6-trimethylbenzyl)benzene (**22**)

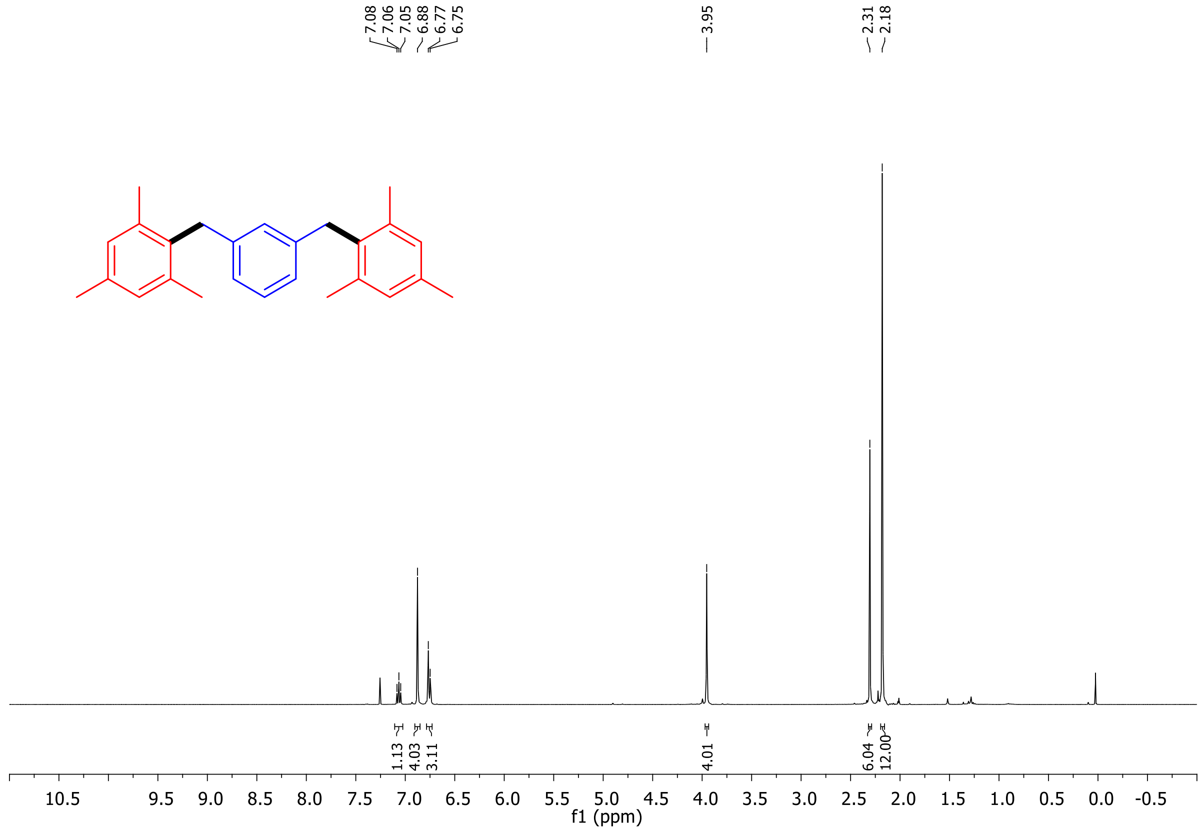

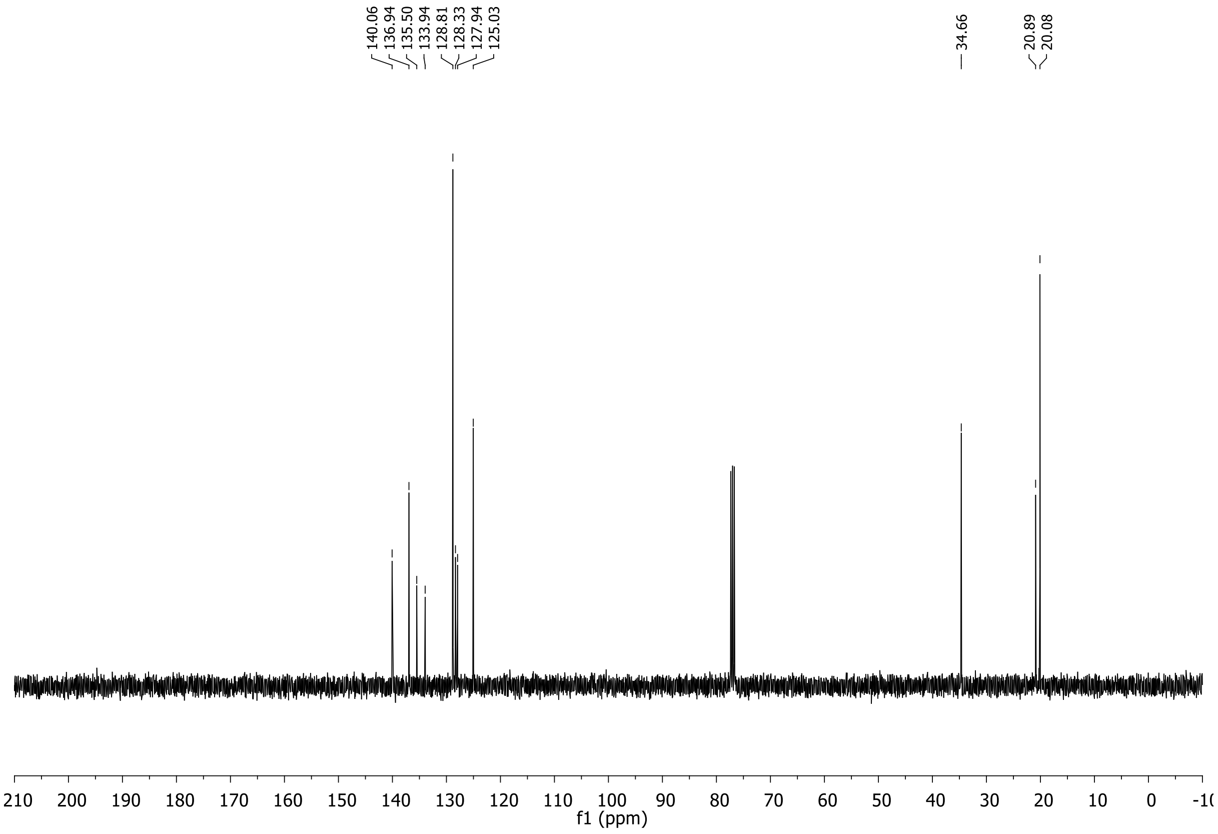

2-Piperonylmesitylene (**23**)

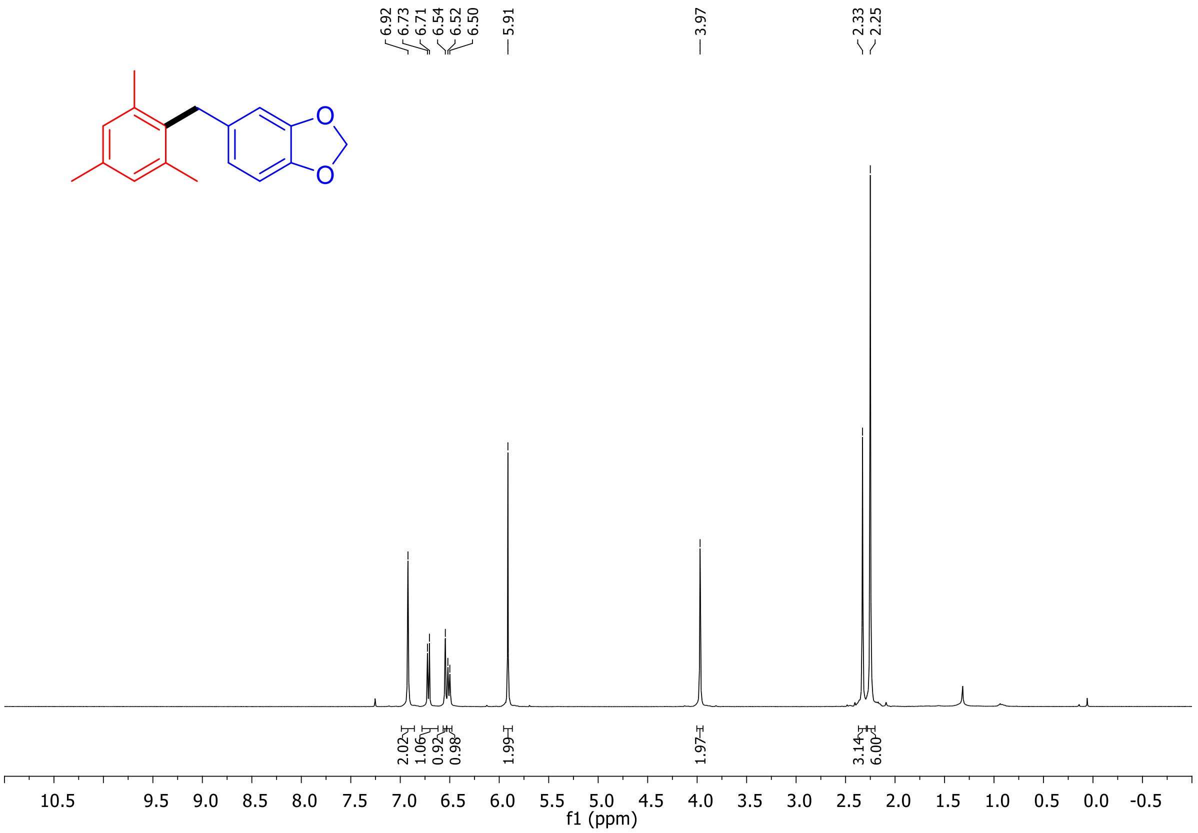

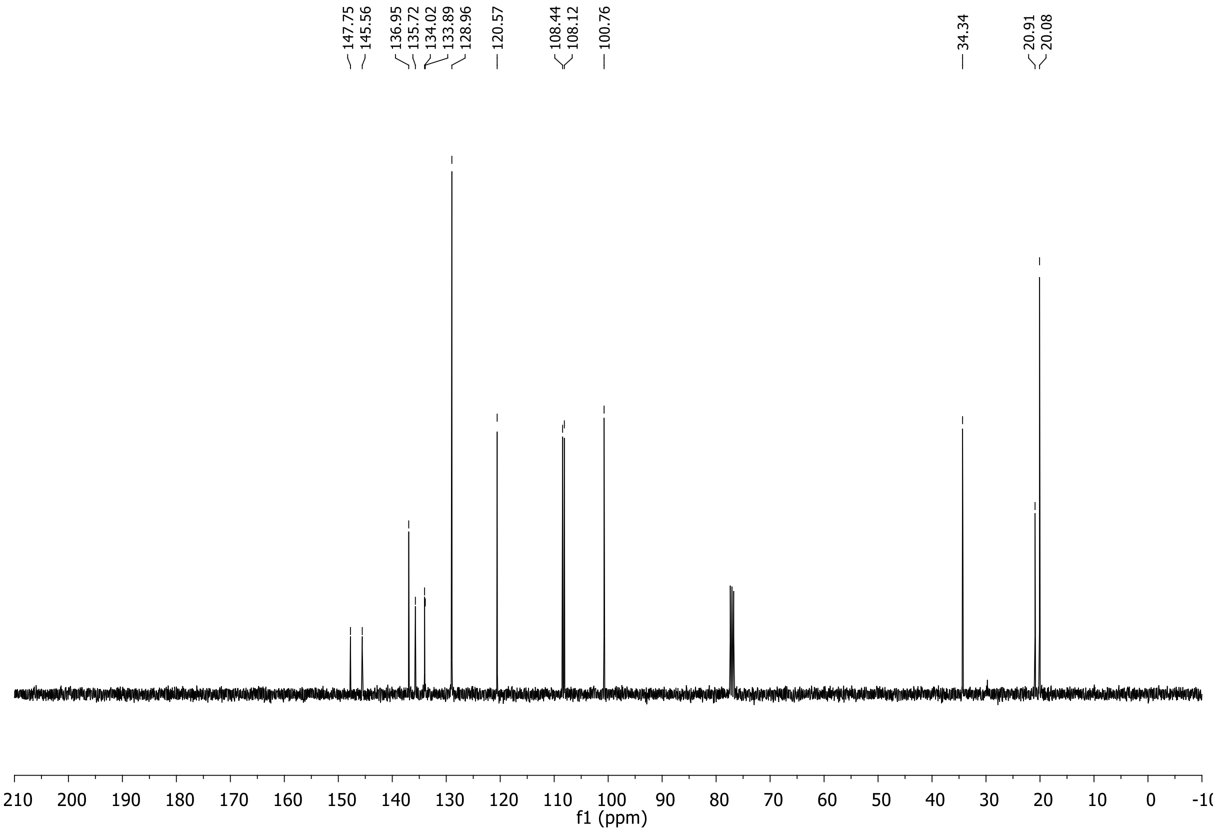

1,4-Dimethyl-2-(2,4-difluorobenzyl)benzene (**24**)

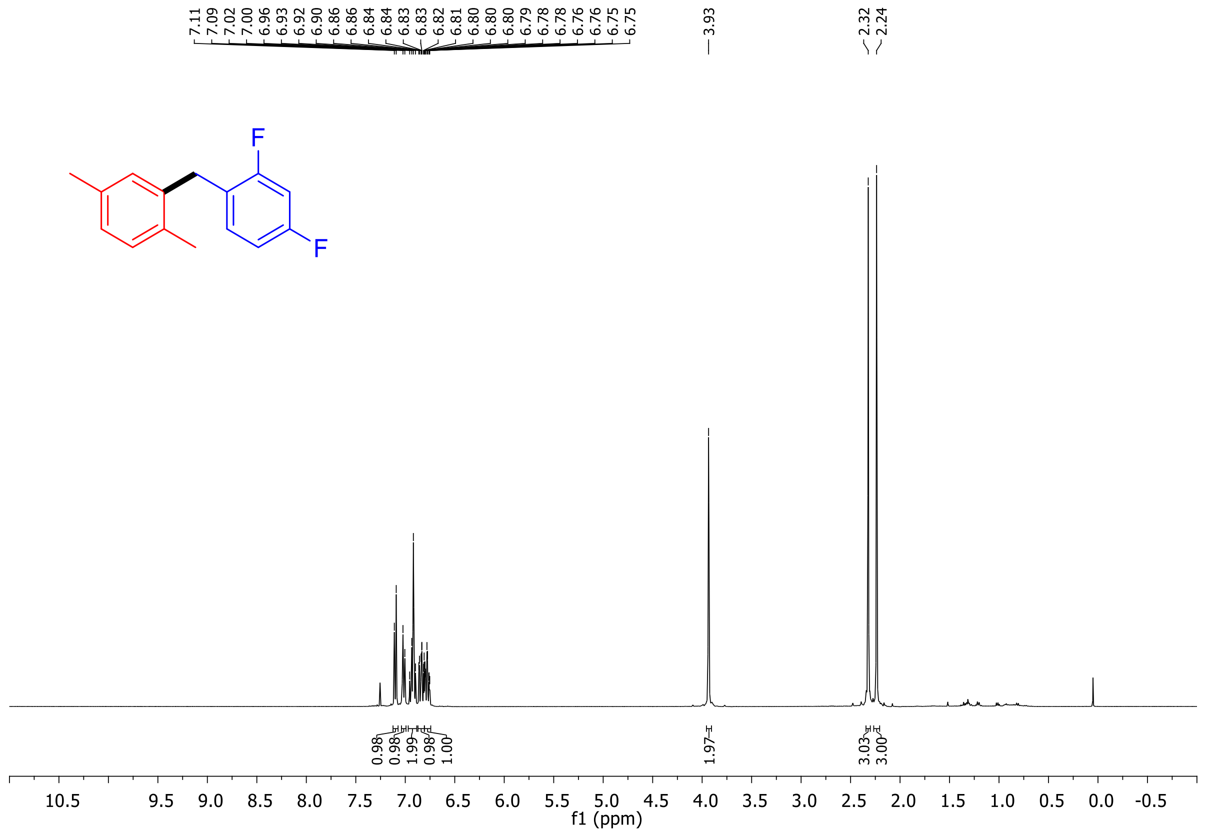

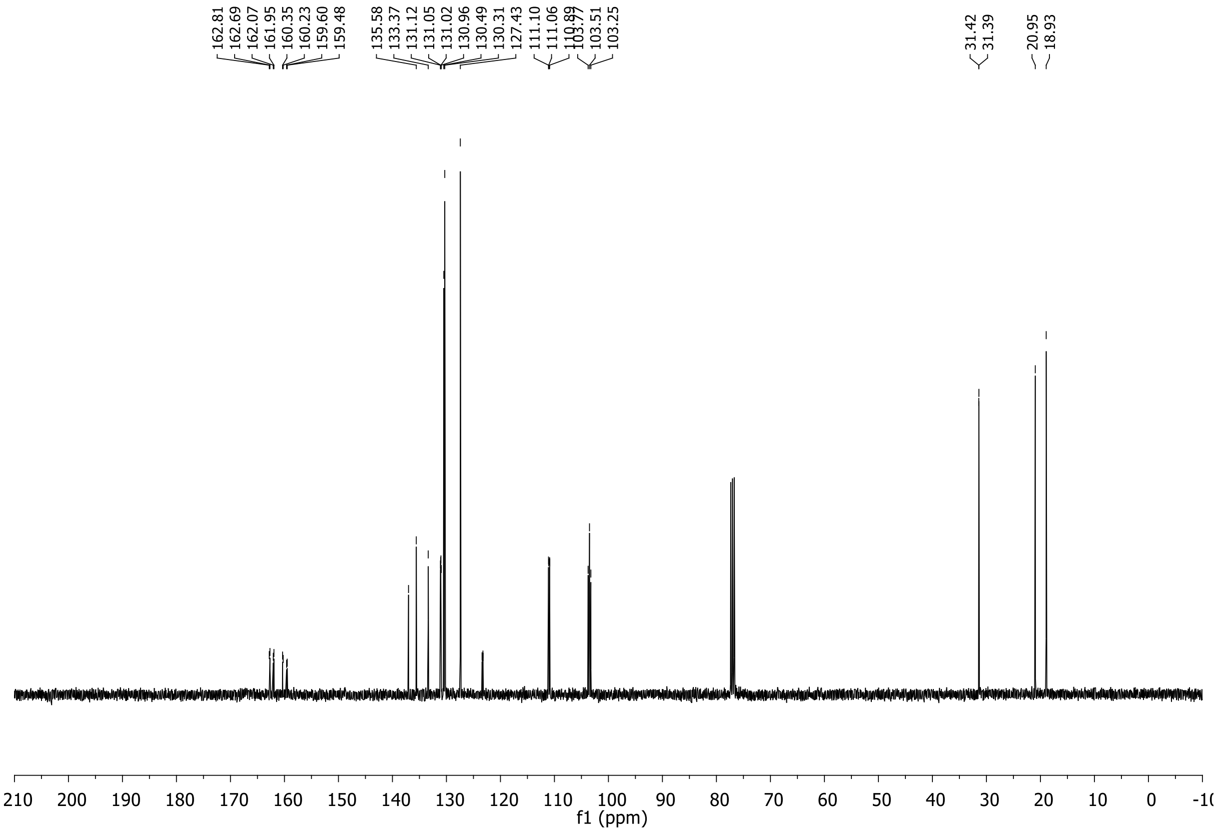

1,4-dimethyl-2-(3-trifluoromethylbenzyl)benzene (**25**)

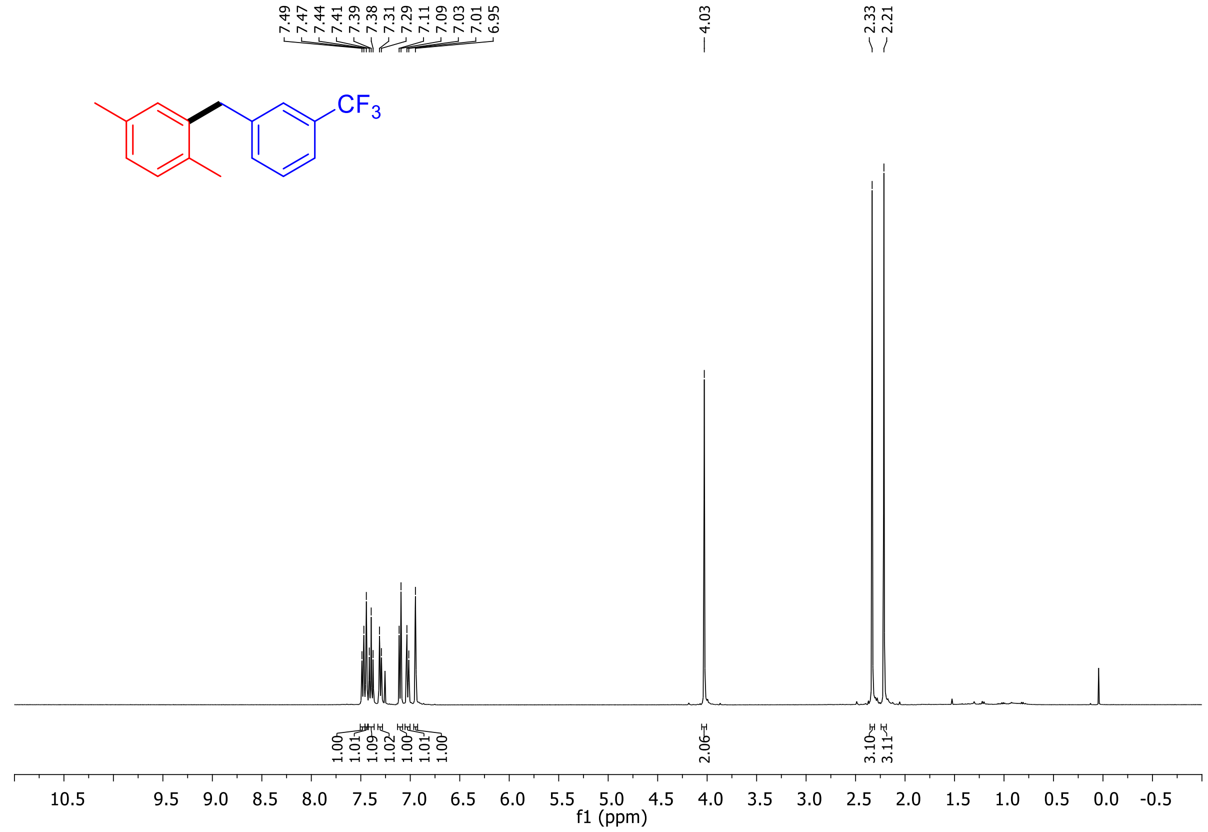

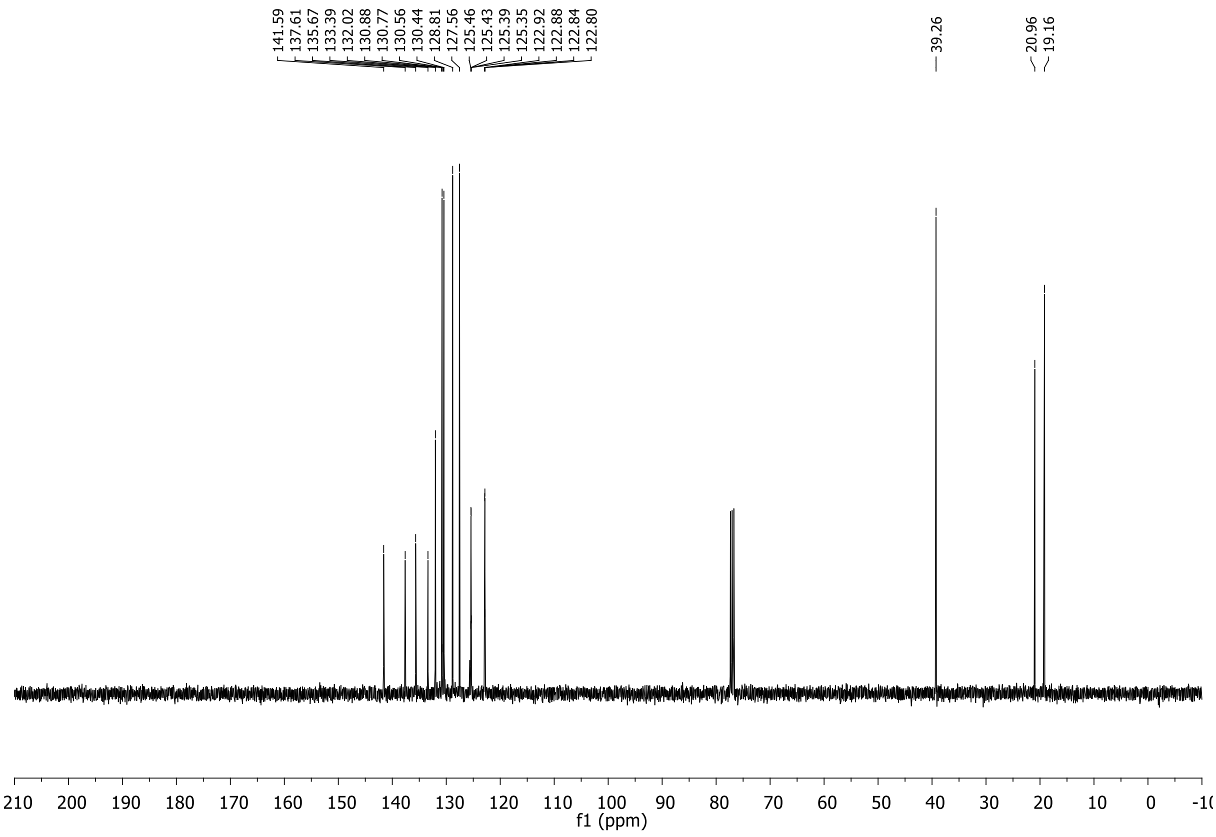

1,4-dimethyl-2-(3,5-bis(trifluoromethyl)benzyl)benzene (**26**)

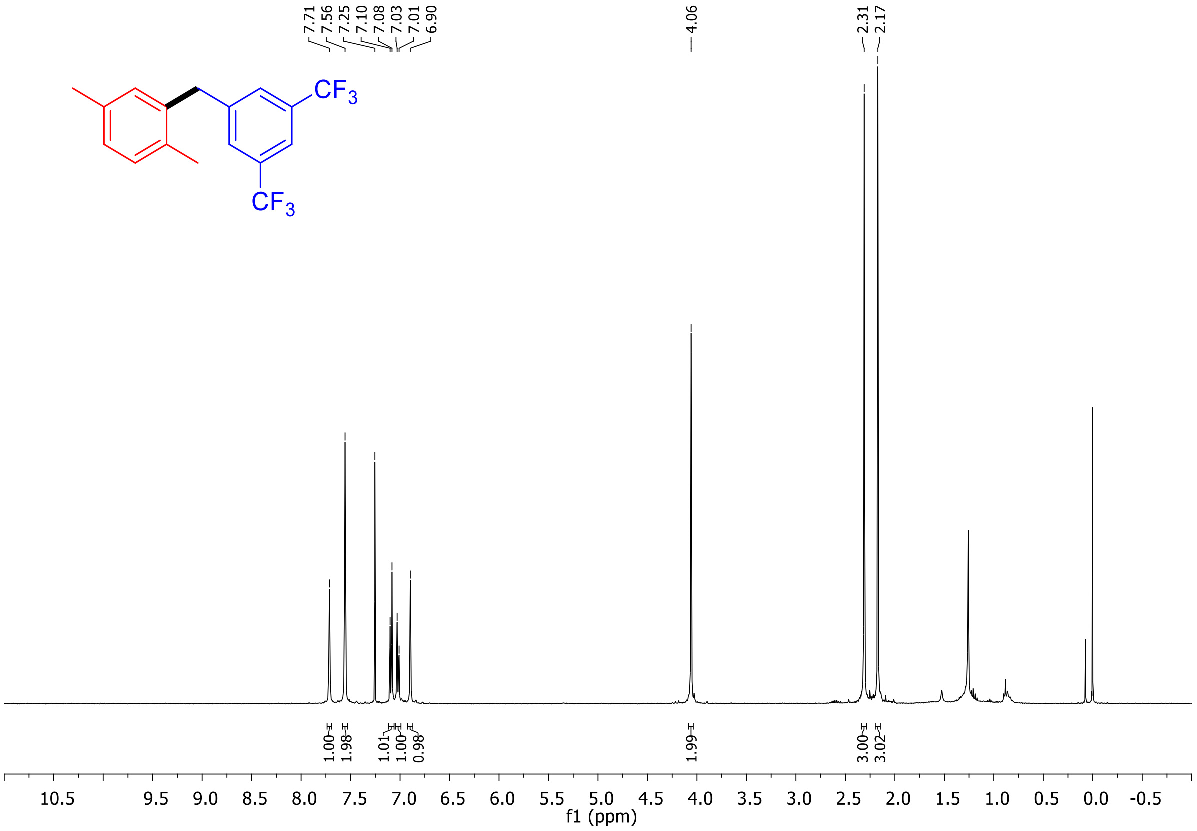

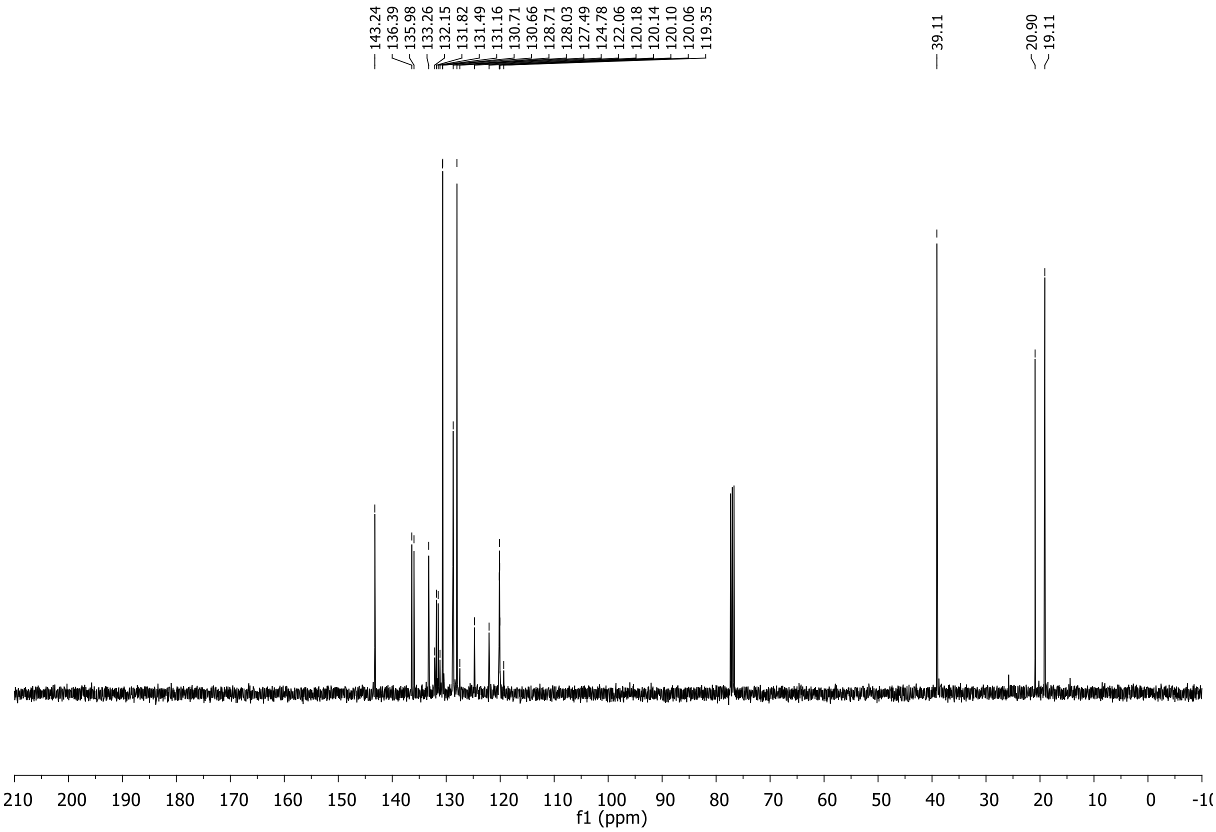

2-dodecylmesitylene (**27**)

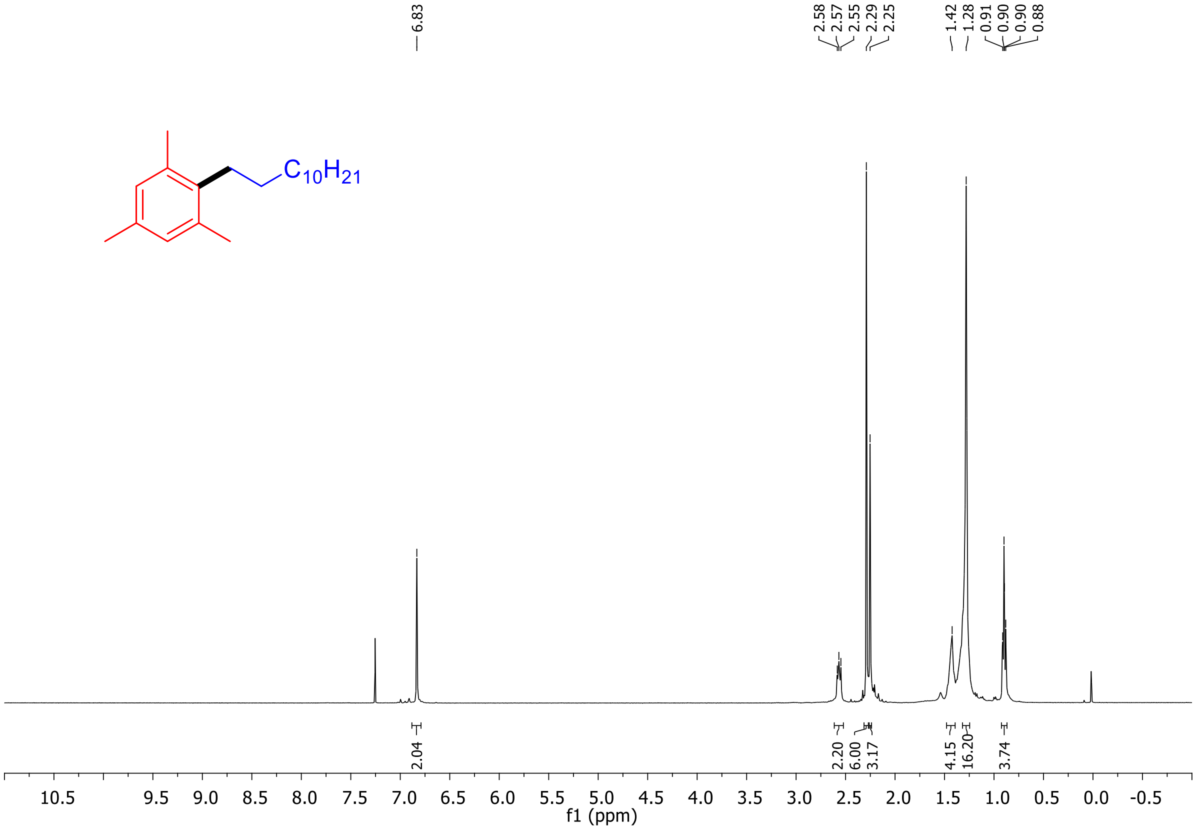

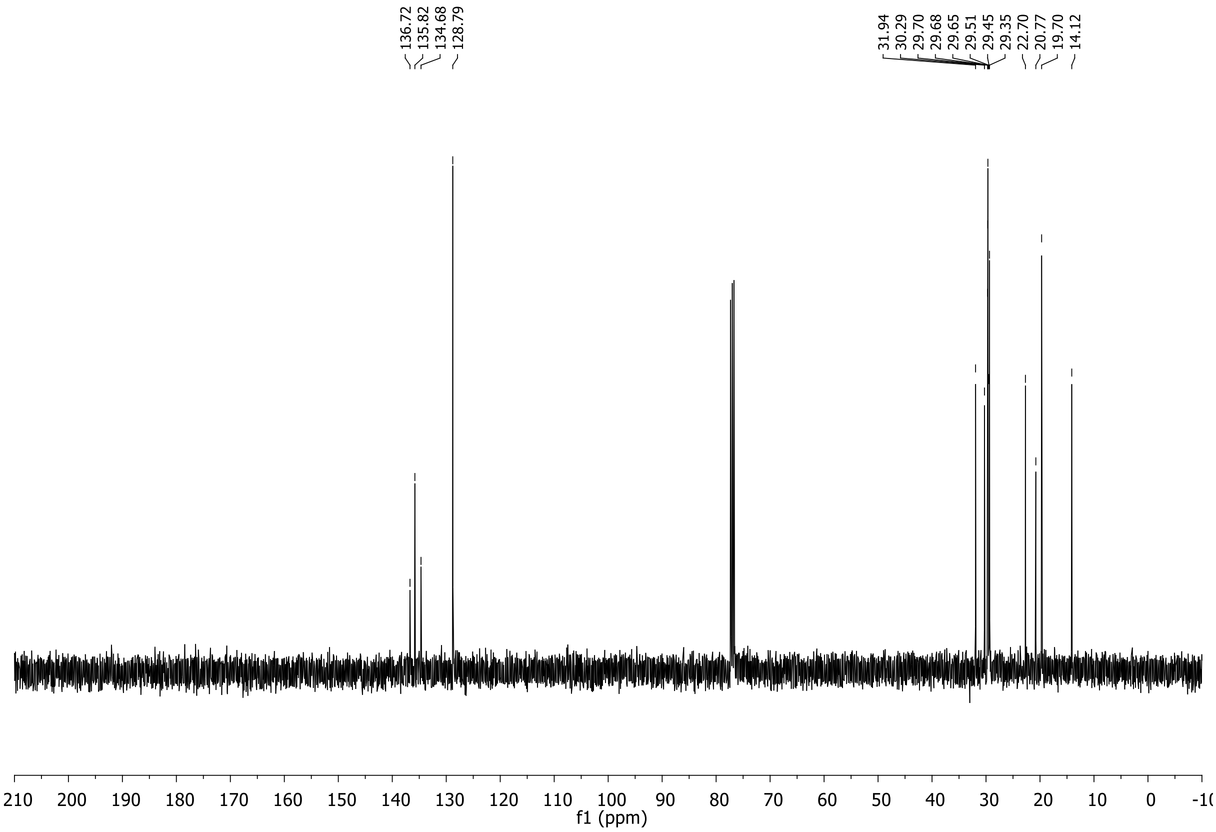

2-hexadecylmesitylene (**28**)

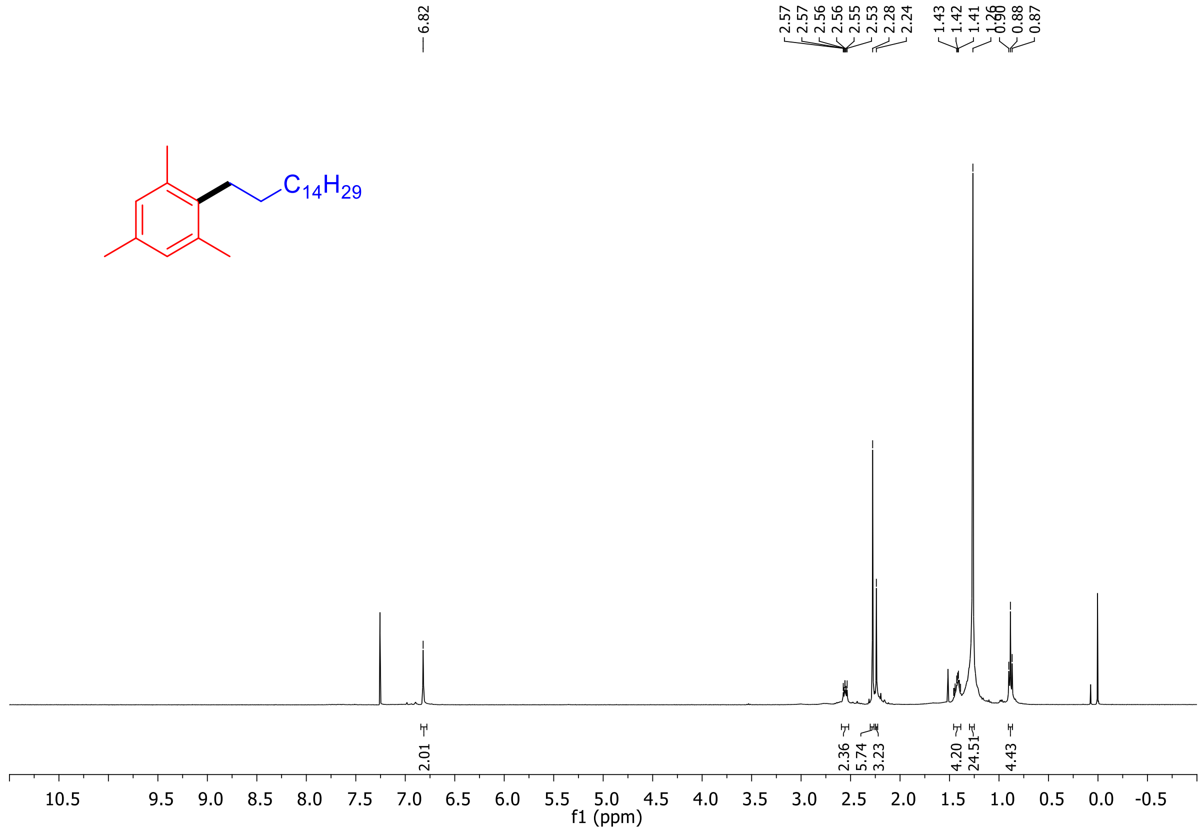

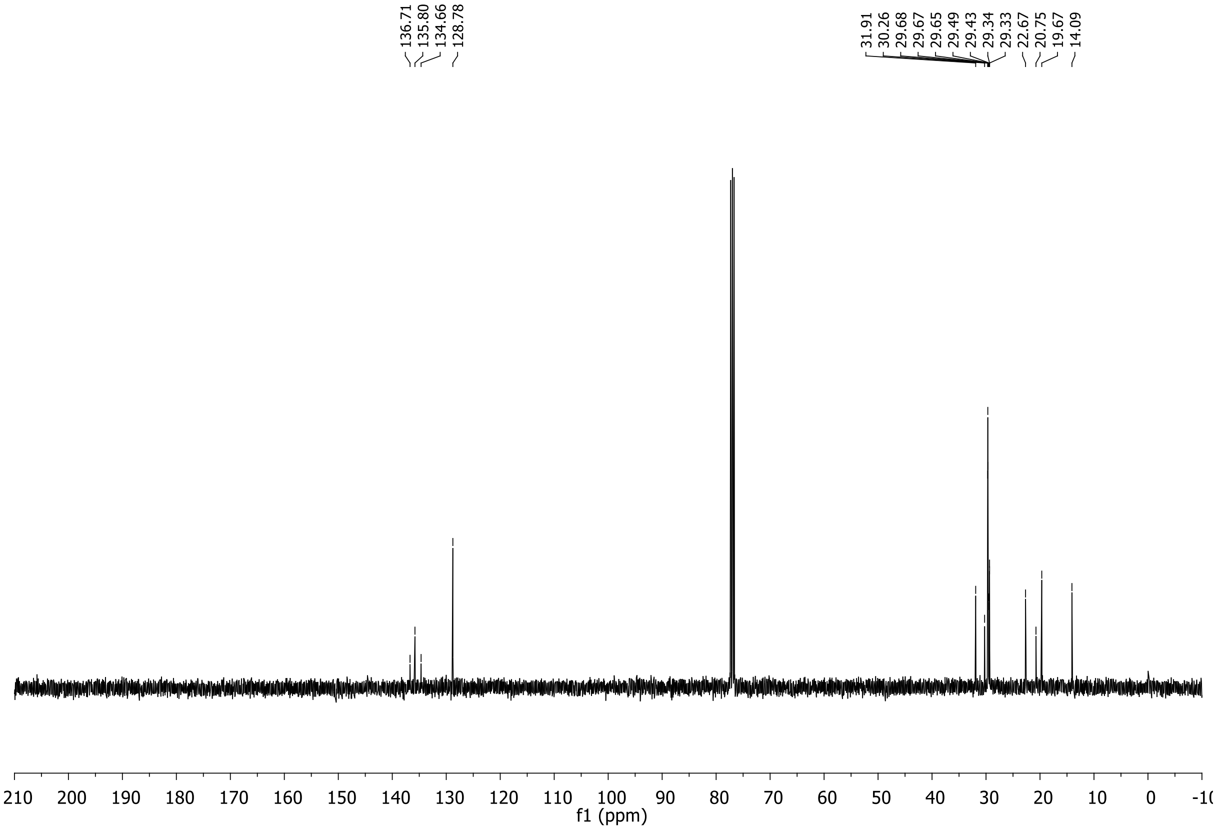

2-(4-methoxyphenethyl)mesitylene (**29**)

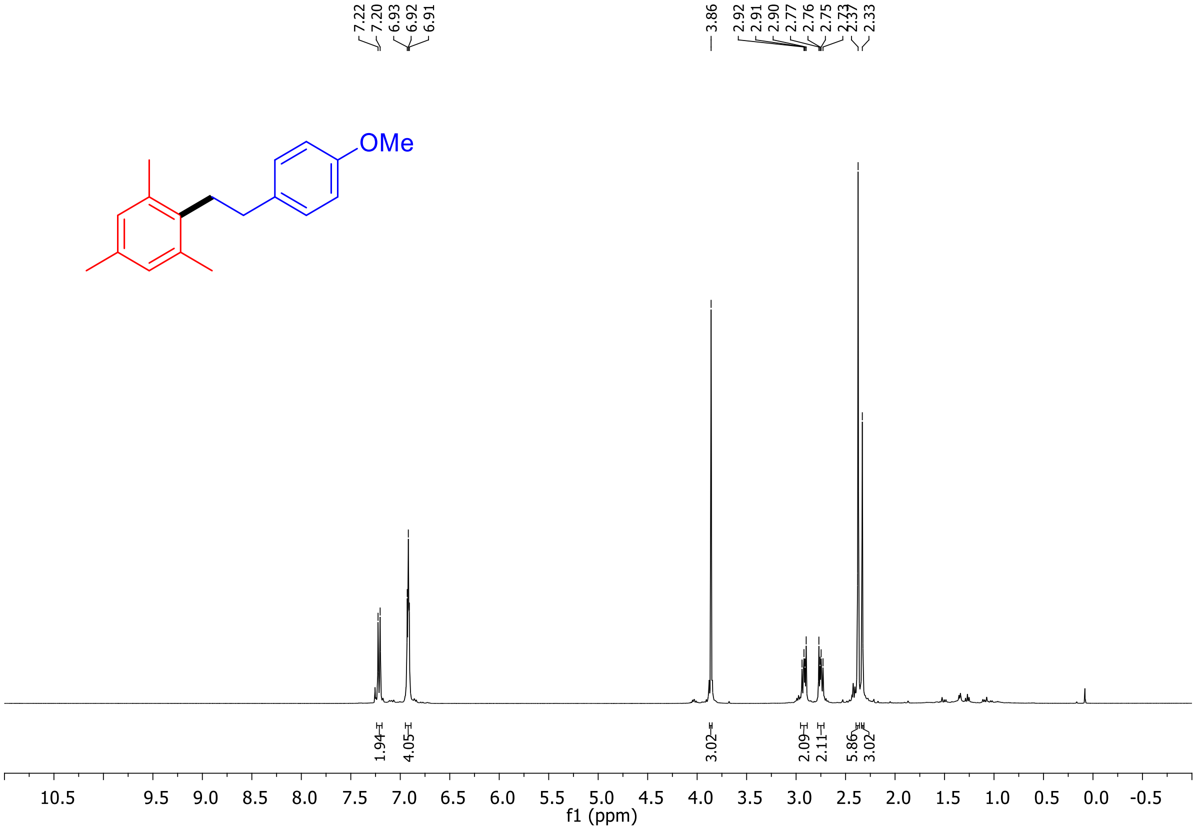

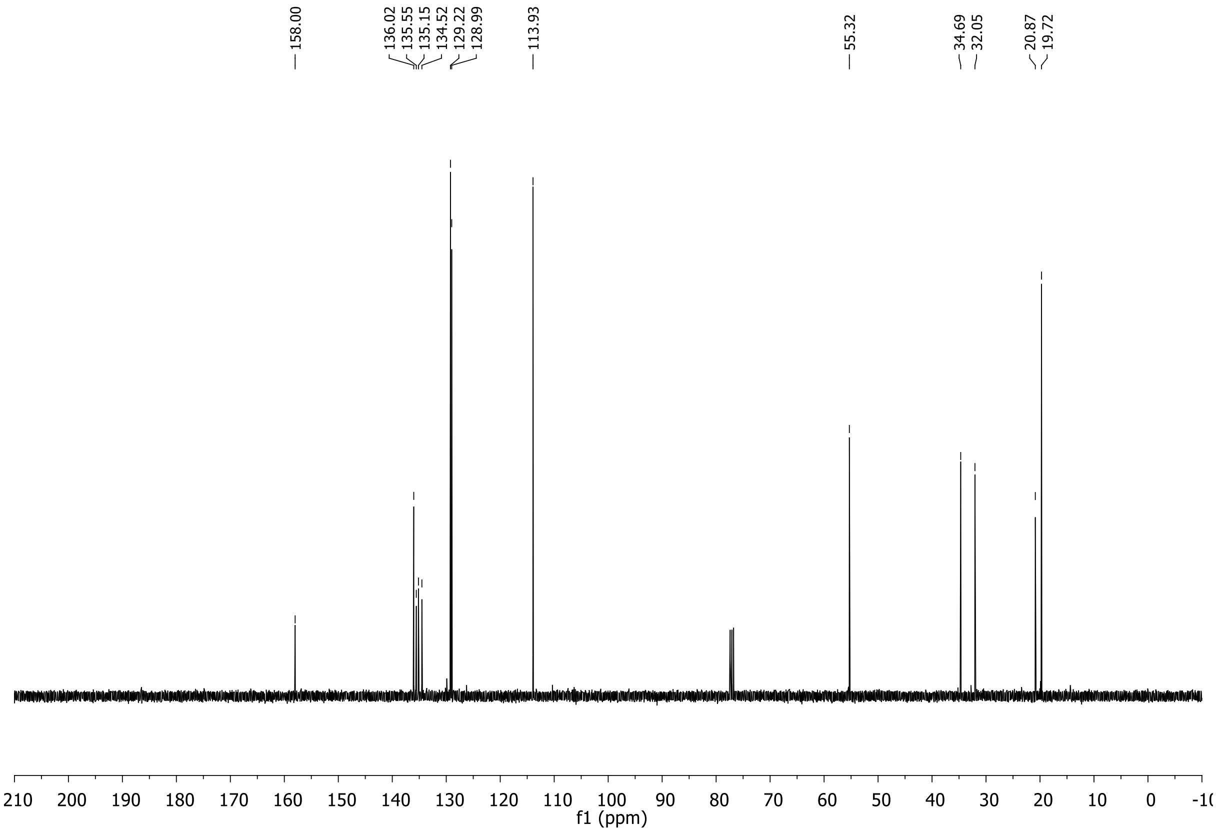

2-(4-chlorophenethyl)mesitylene (**30**)

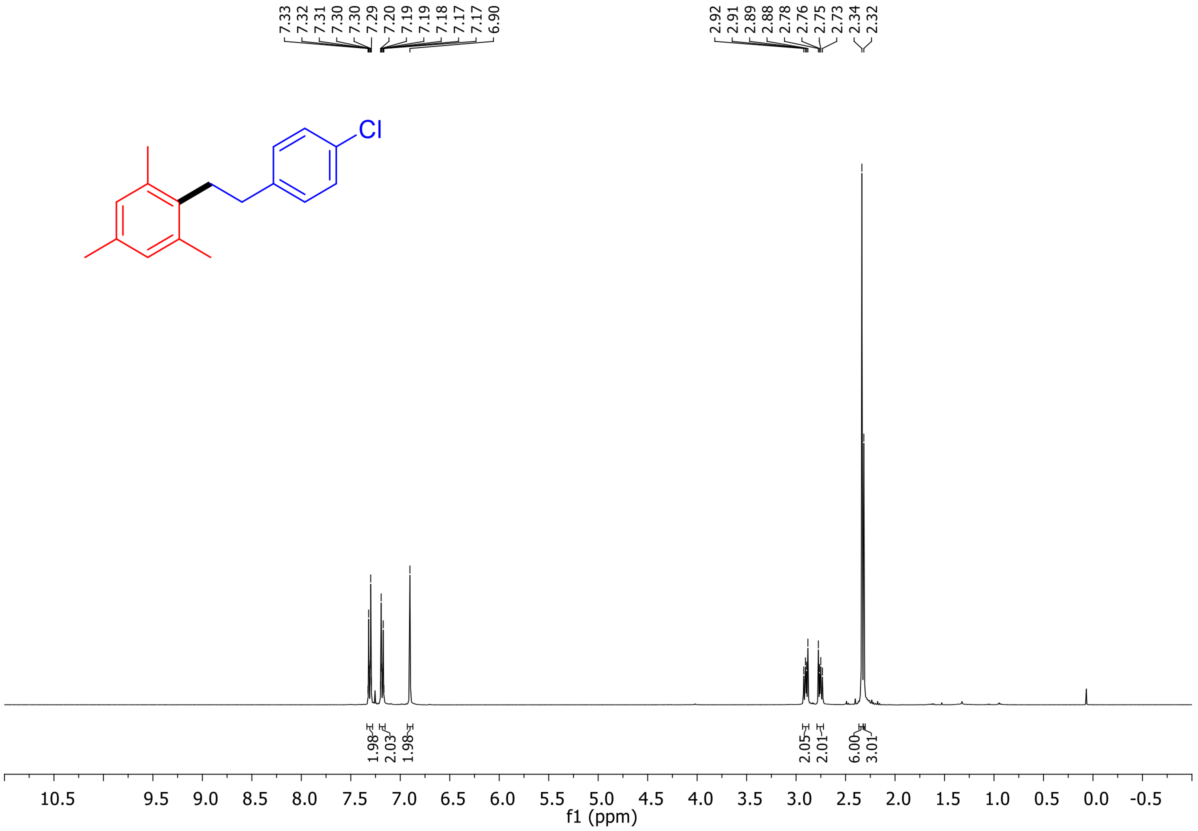

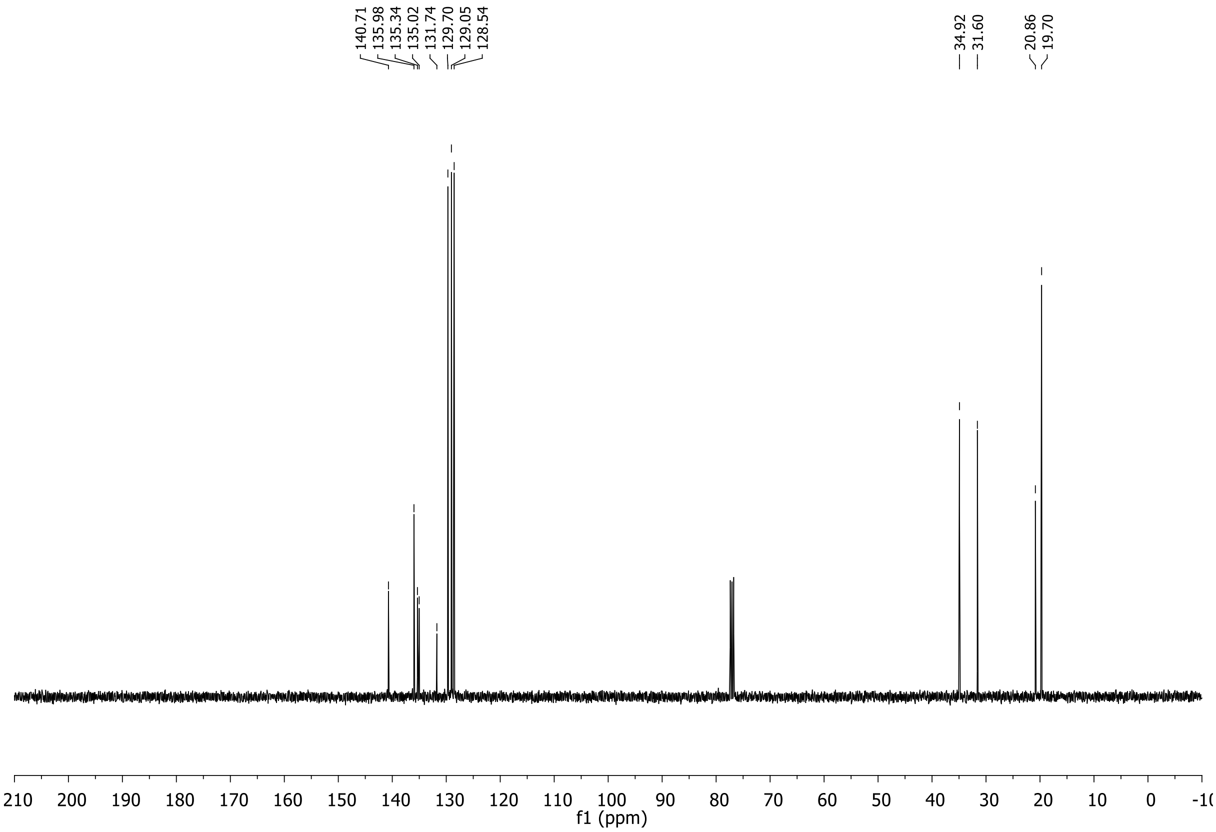

2-(4-trifluoromethylphenethyl)mesitylene (**31**)

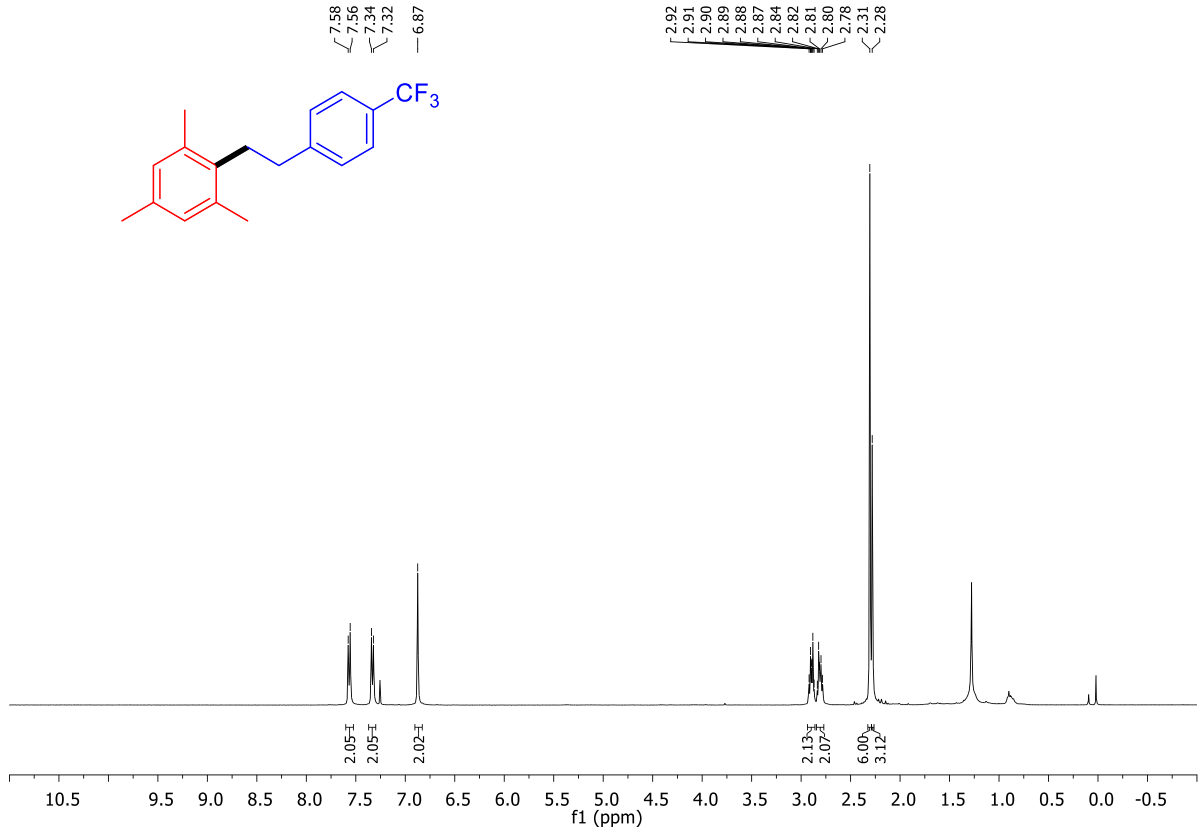

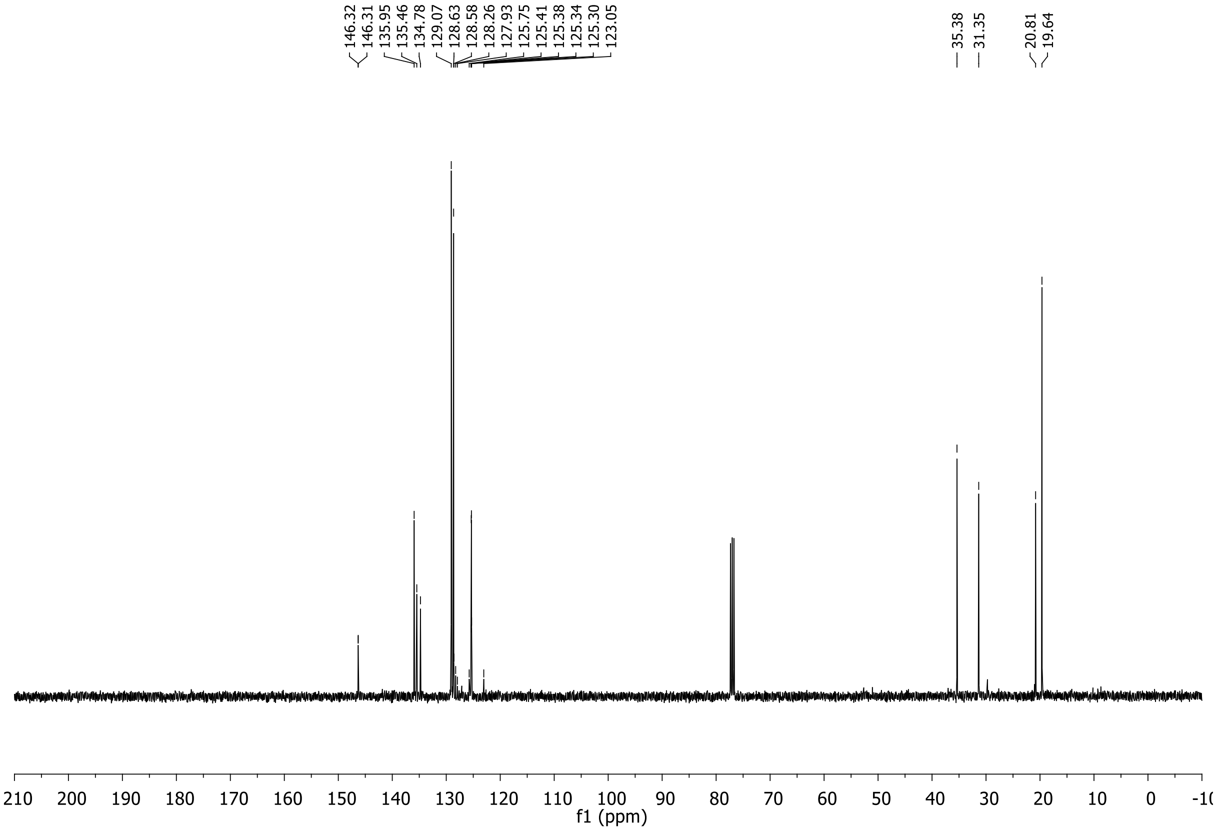

2-cyclohexylmesitylene (**32**)

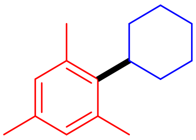

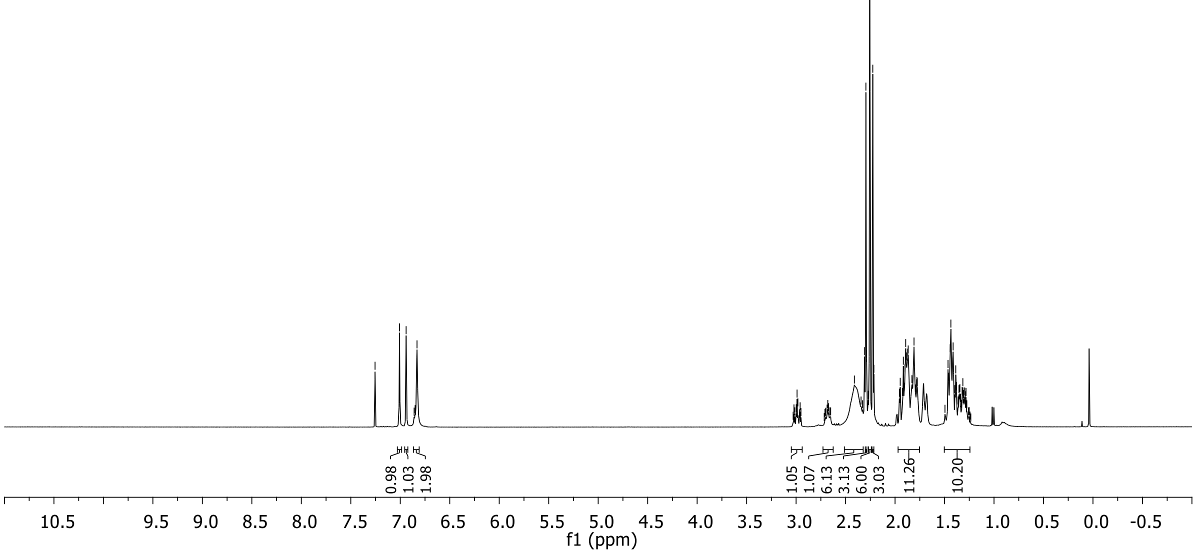

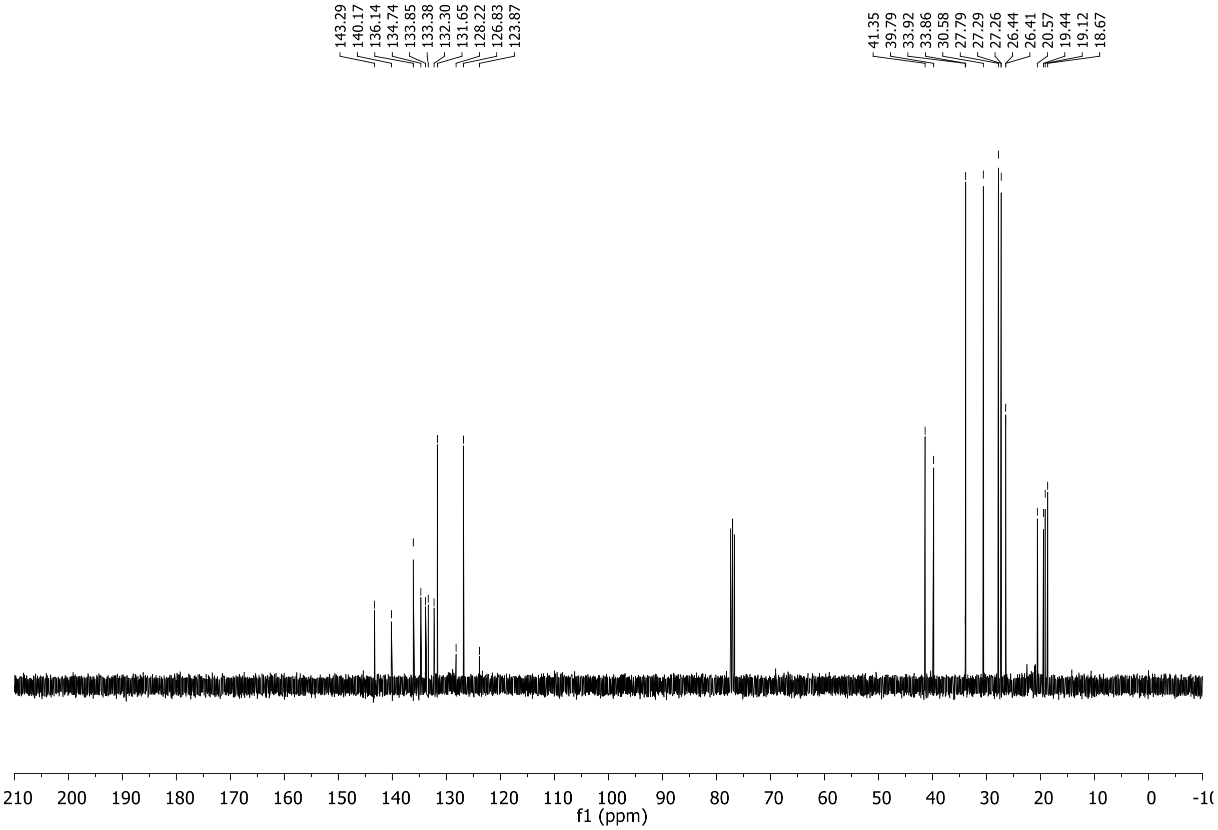

2-cyclododecylmesitylene (**33**)

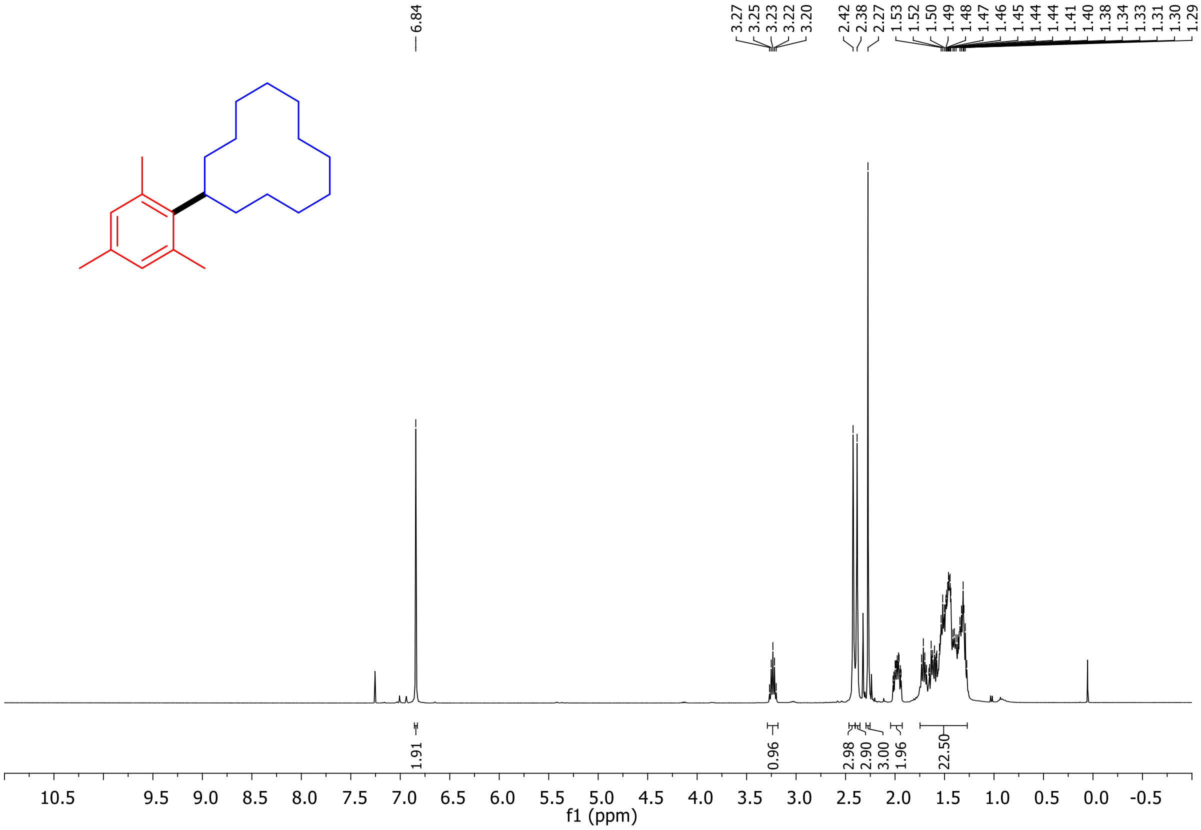

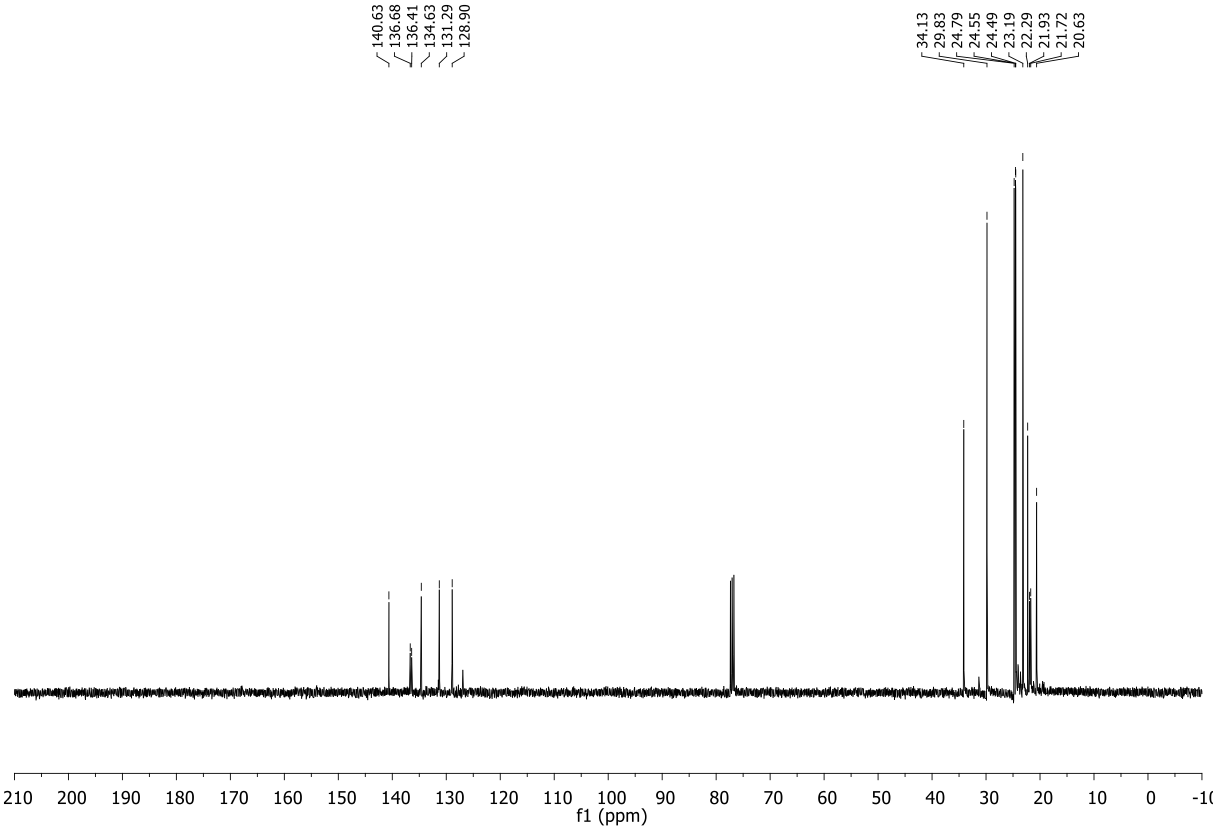

2-(1-phenylethyl)mesitylene (**34**)

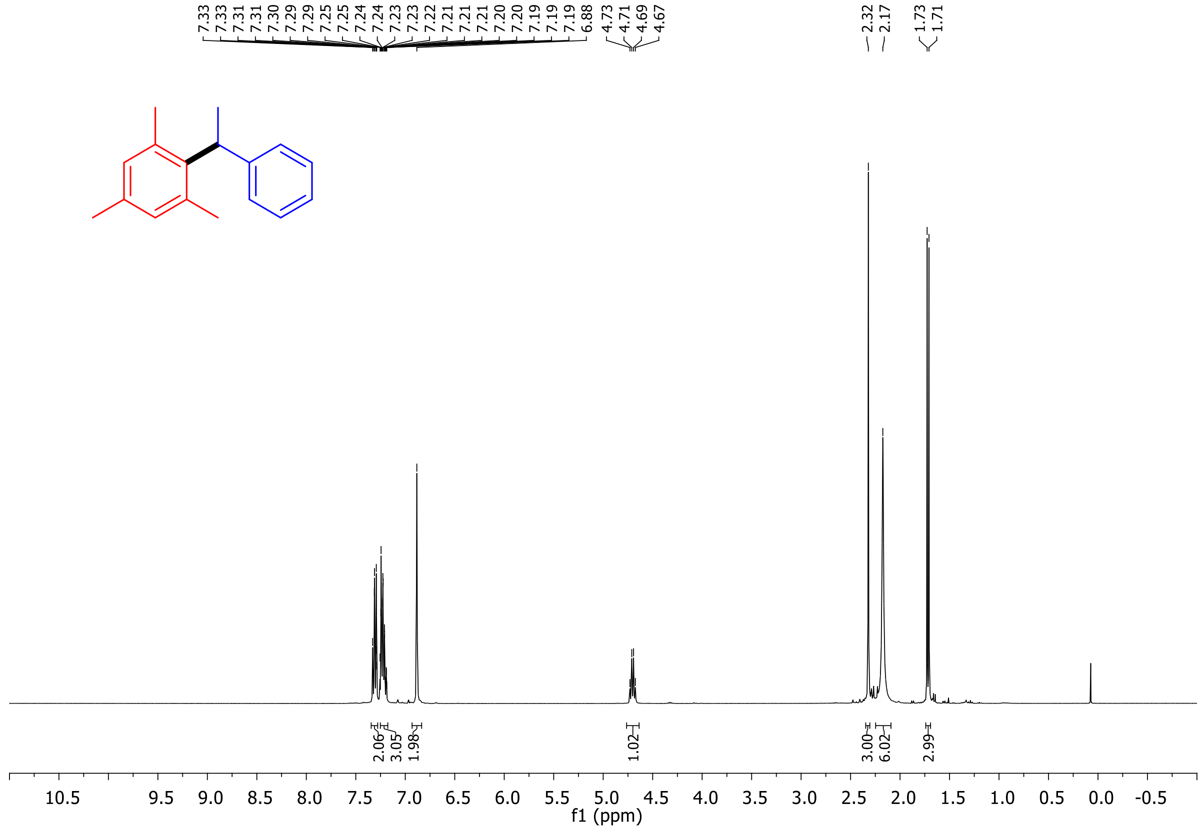

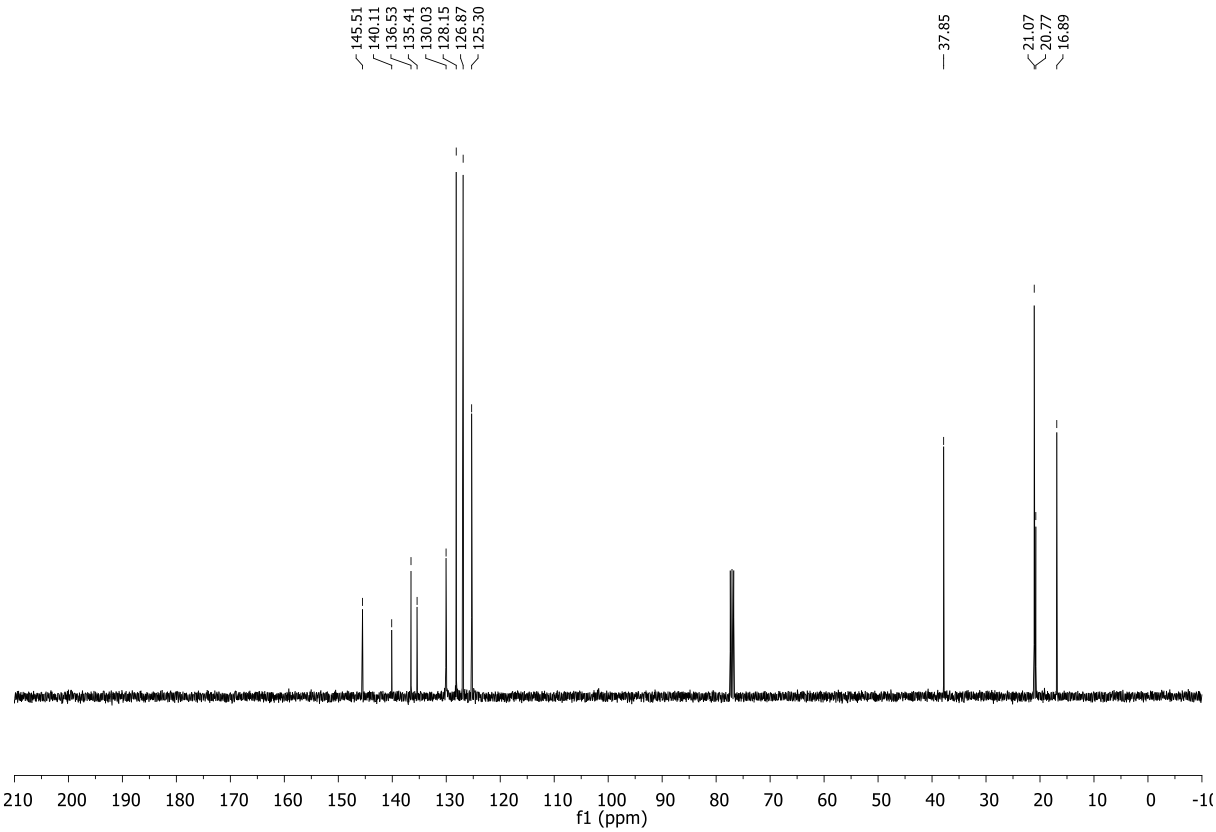

2-(1-(4-nitrophenyl)ethyl)mesitylene (**35**)

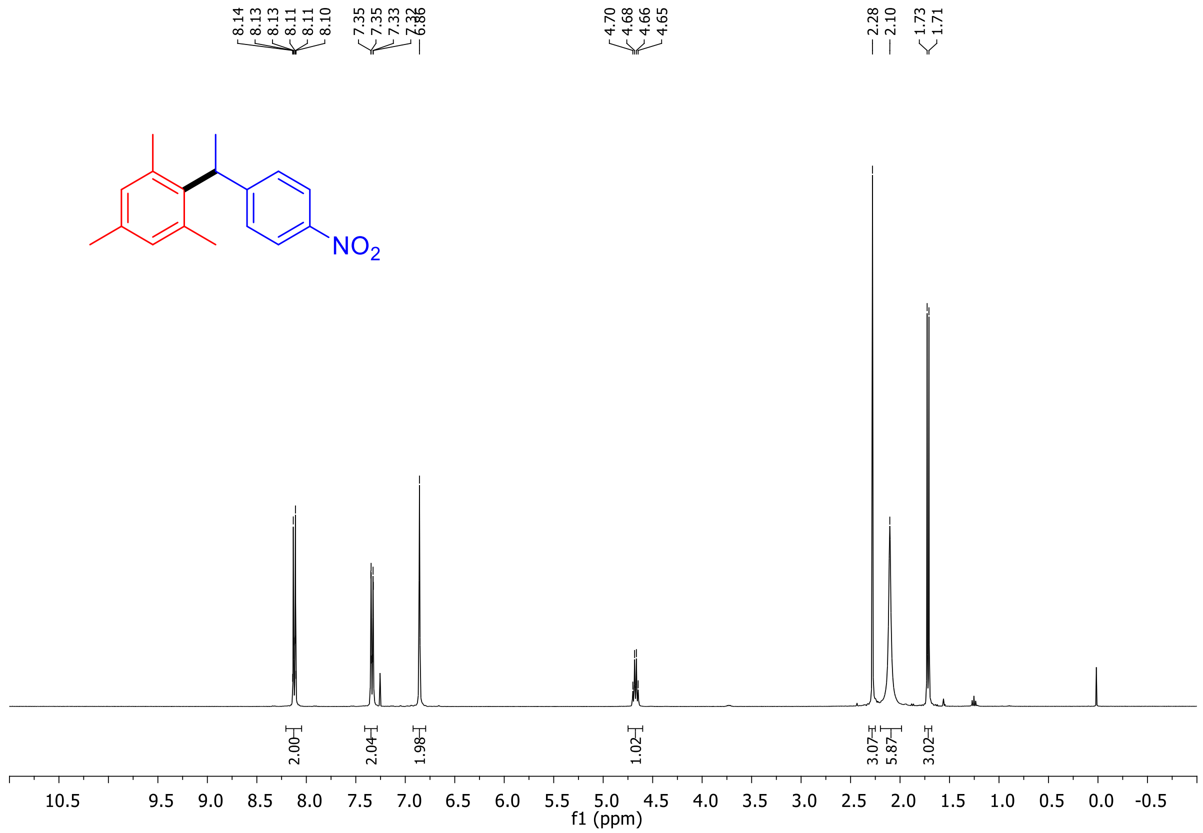

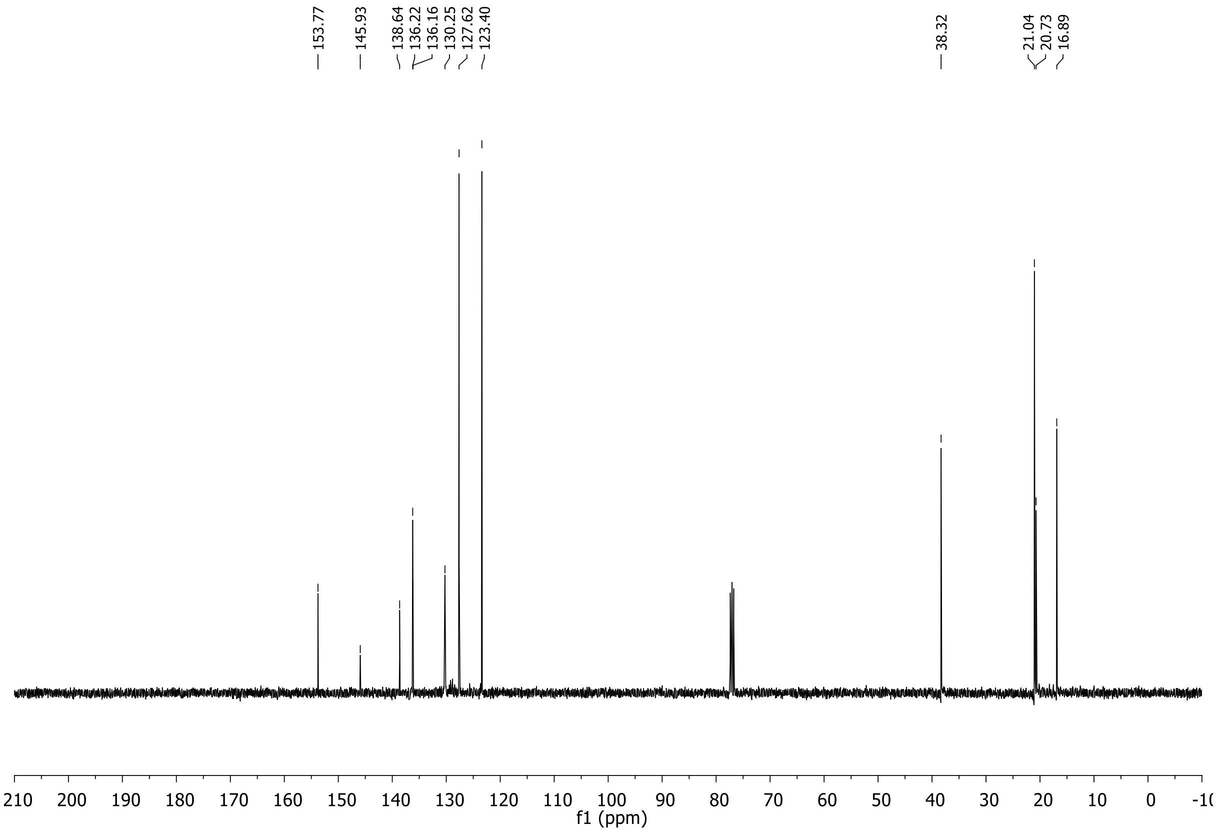

3-mesityl-1-phenylbutane (**37A**), 2-mesityl-1-phenylbutane (**37B**)

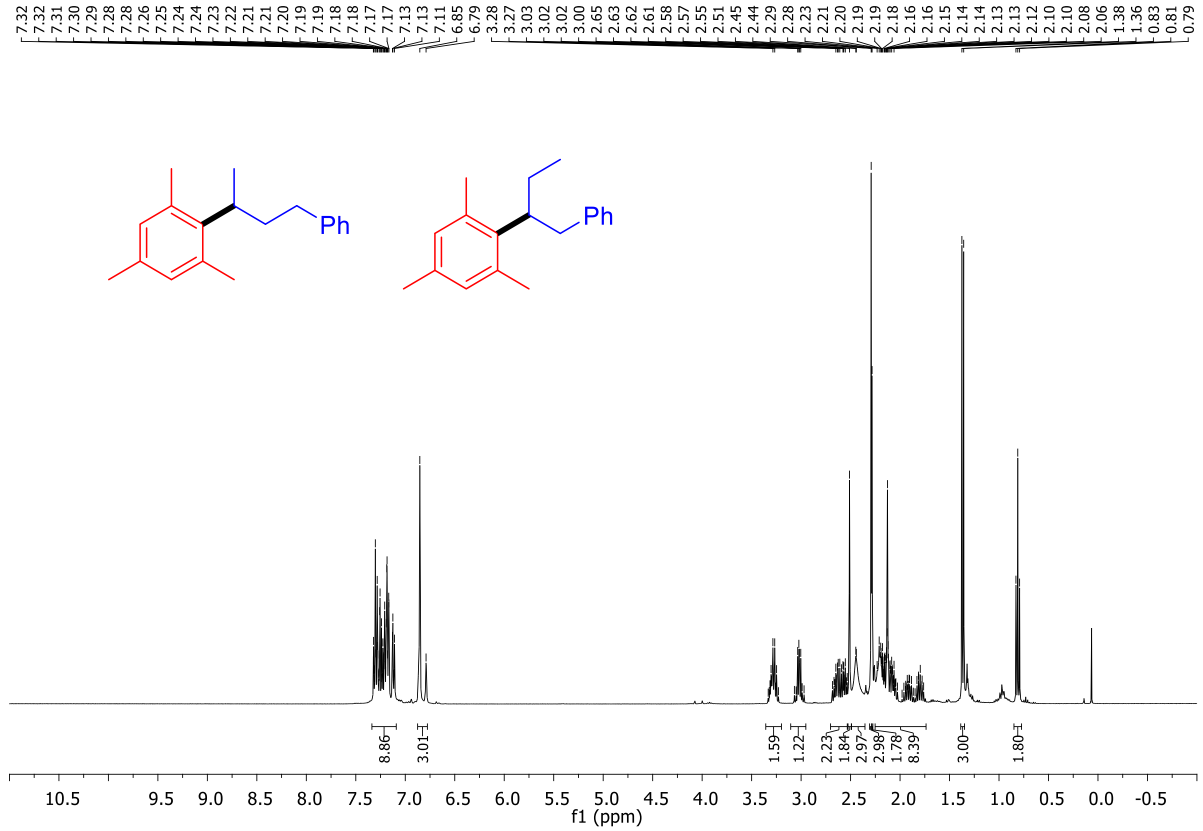

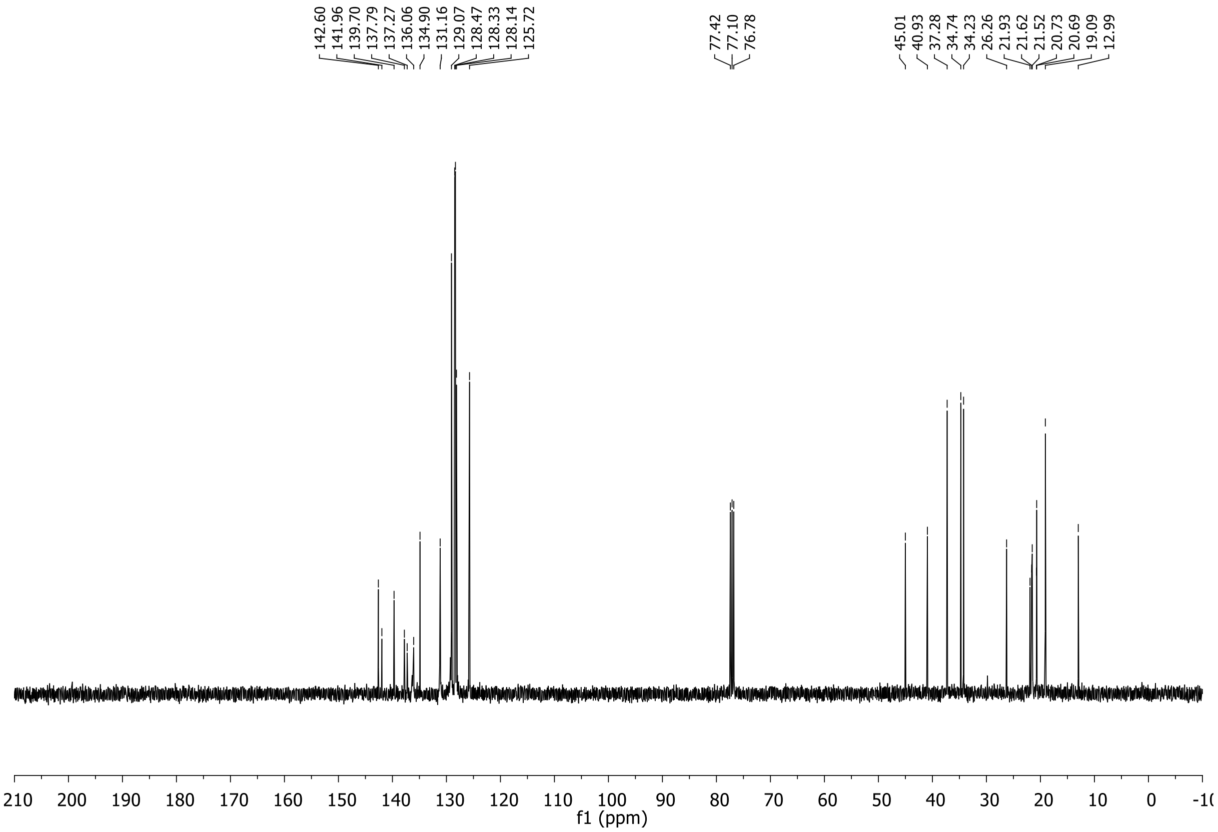

1-(3,4-dimethylphenyl)adamantane (**38**)

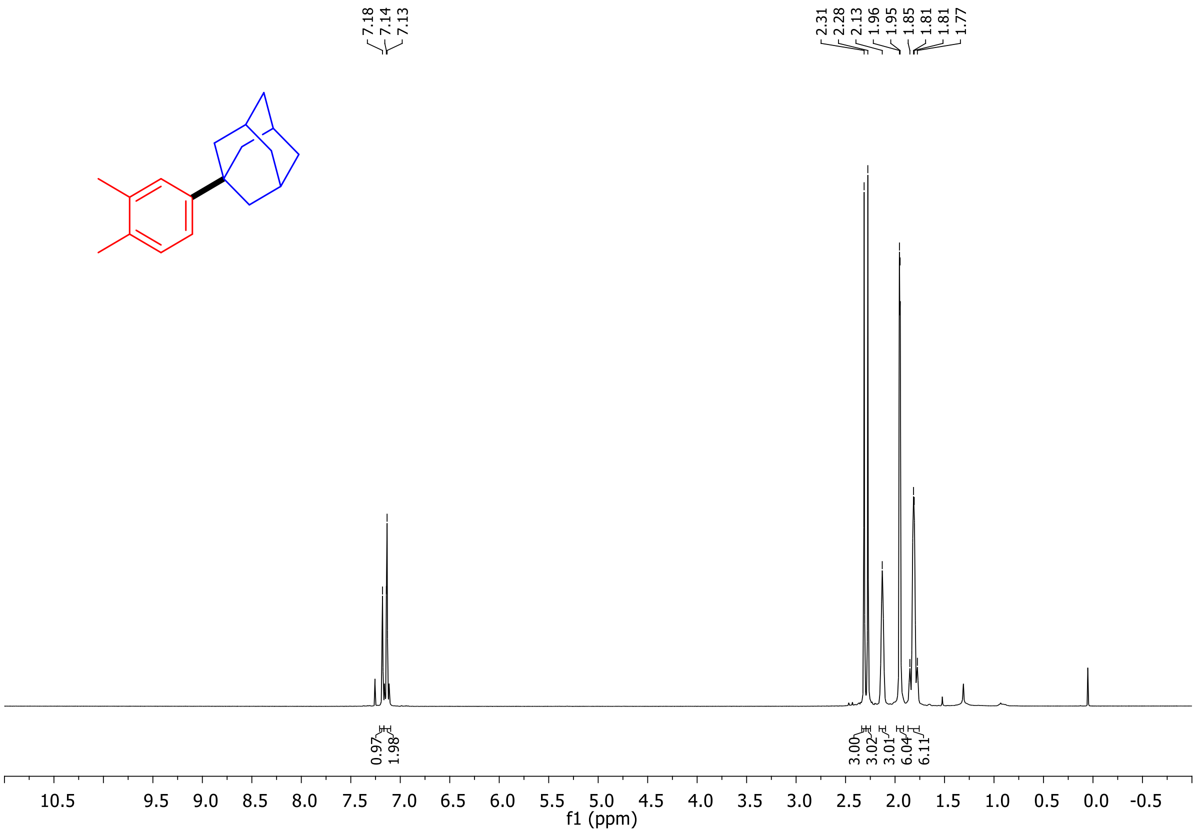

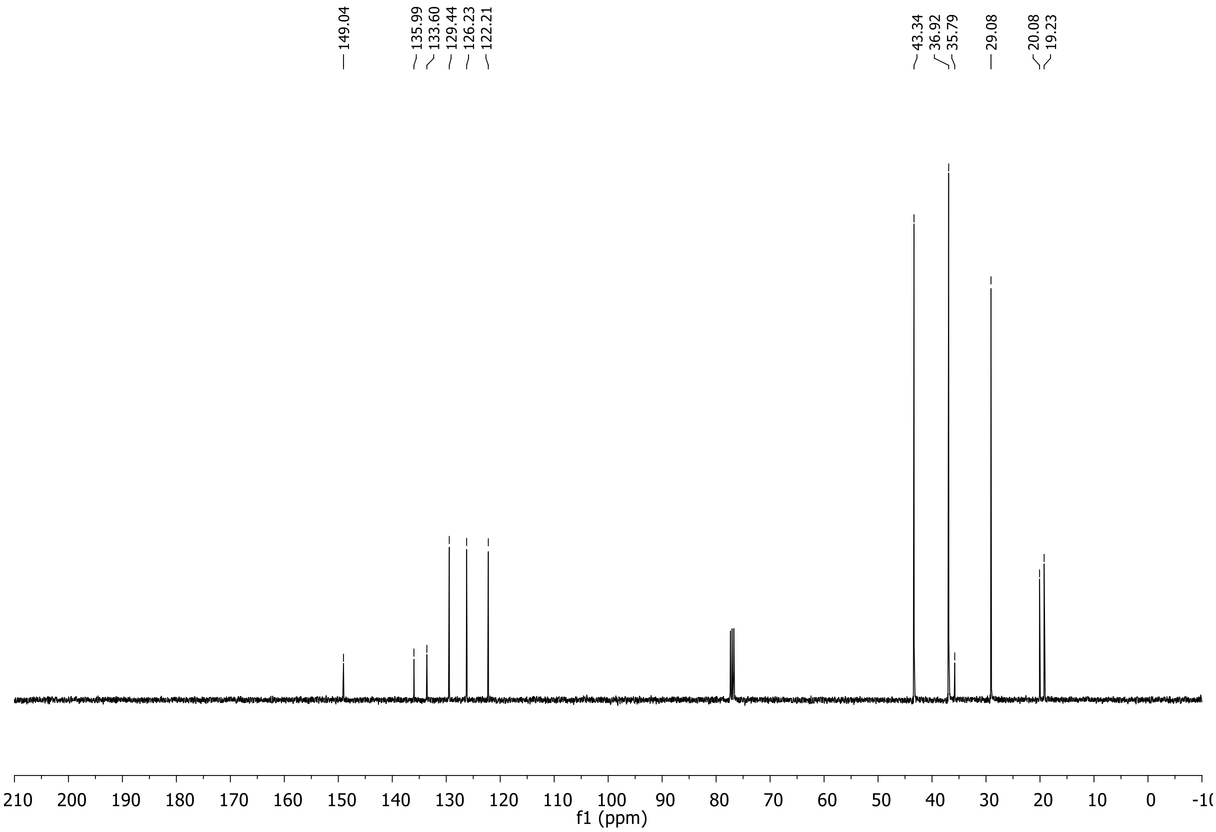

1-(4-methylphenyl)adamantane (**39**)

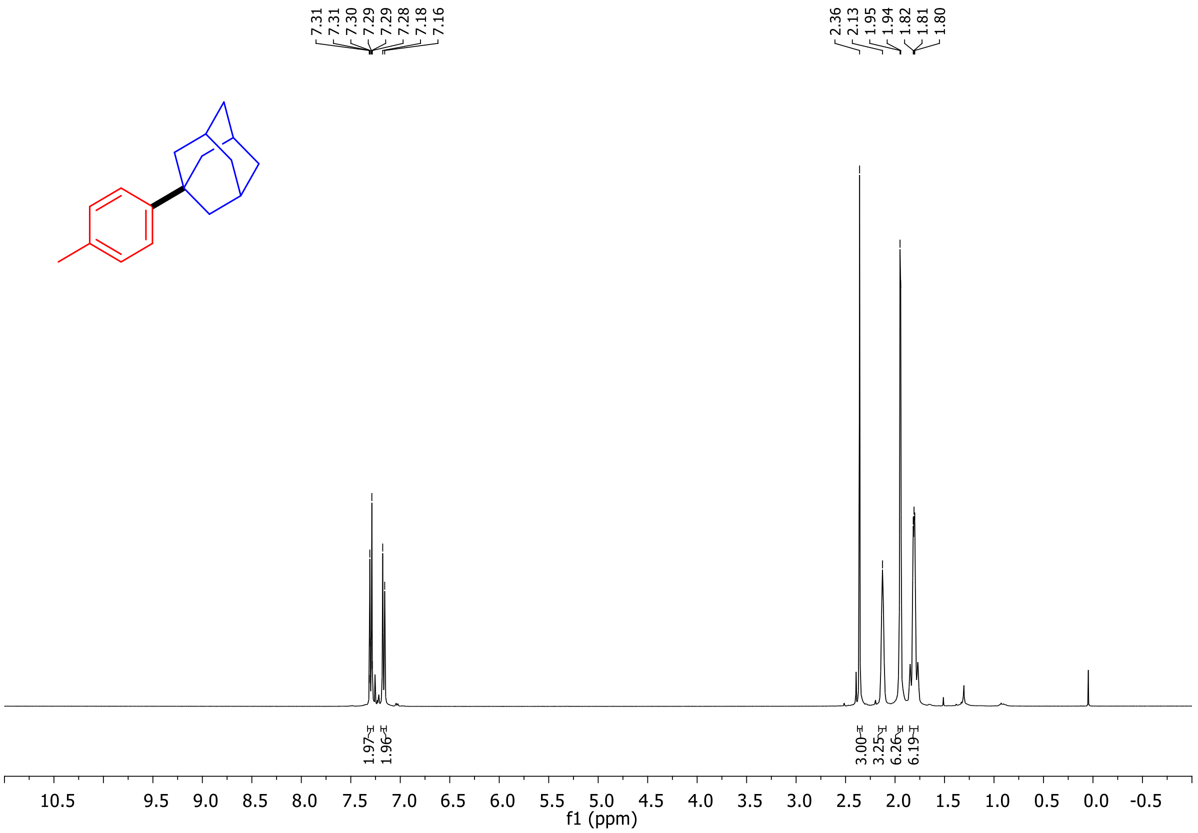

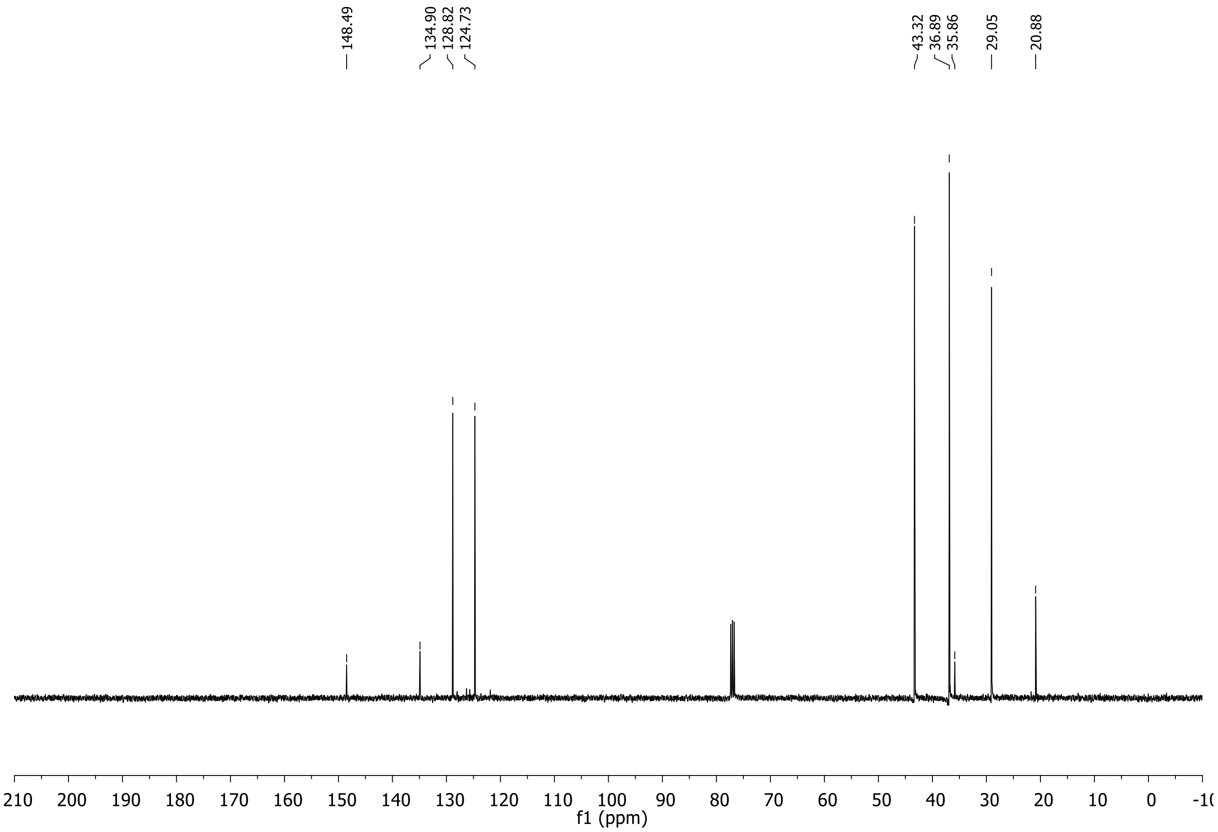

4-cumylanisole (**40**)

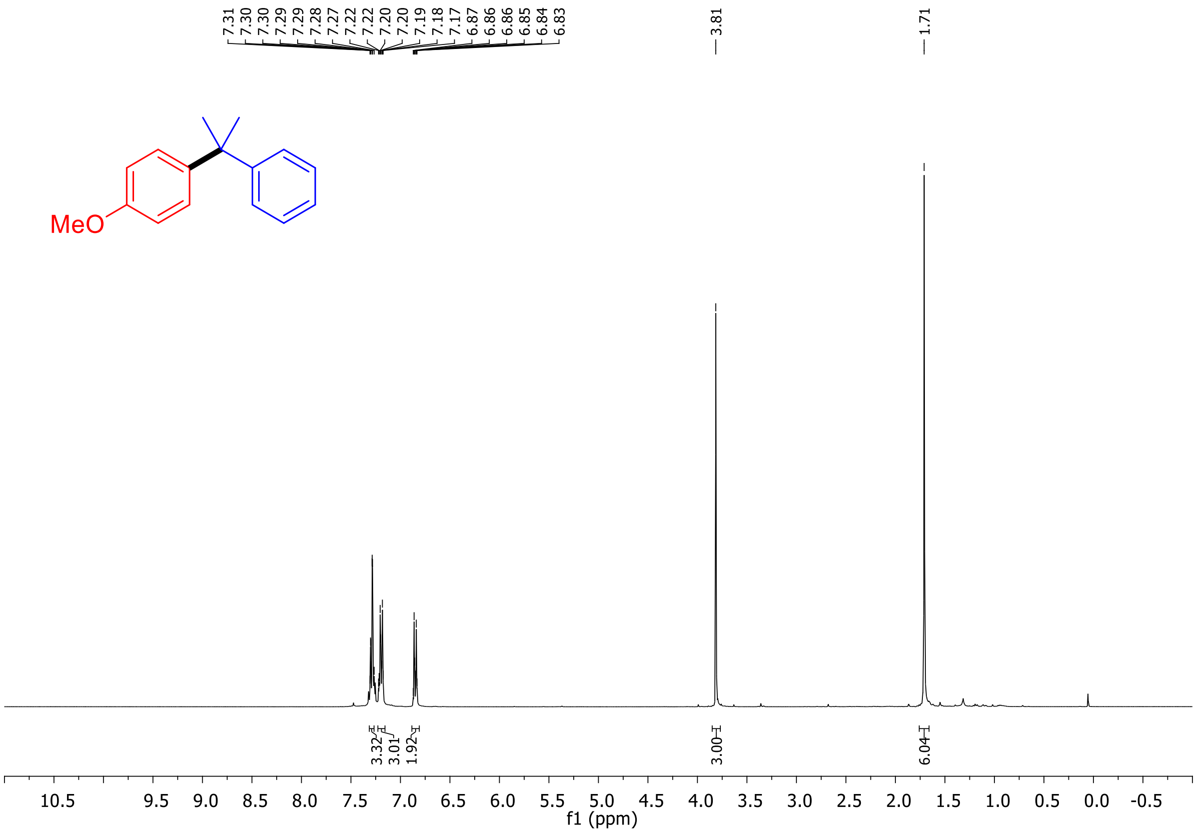

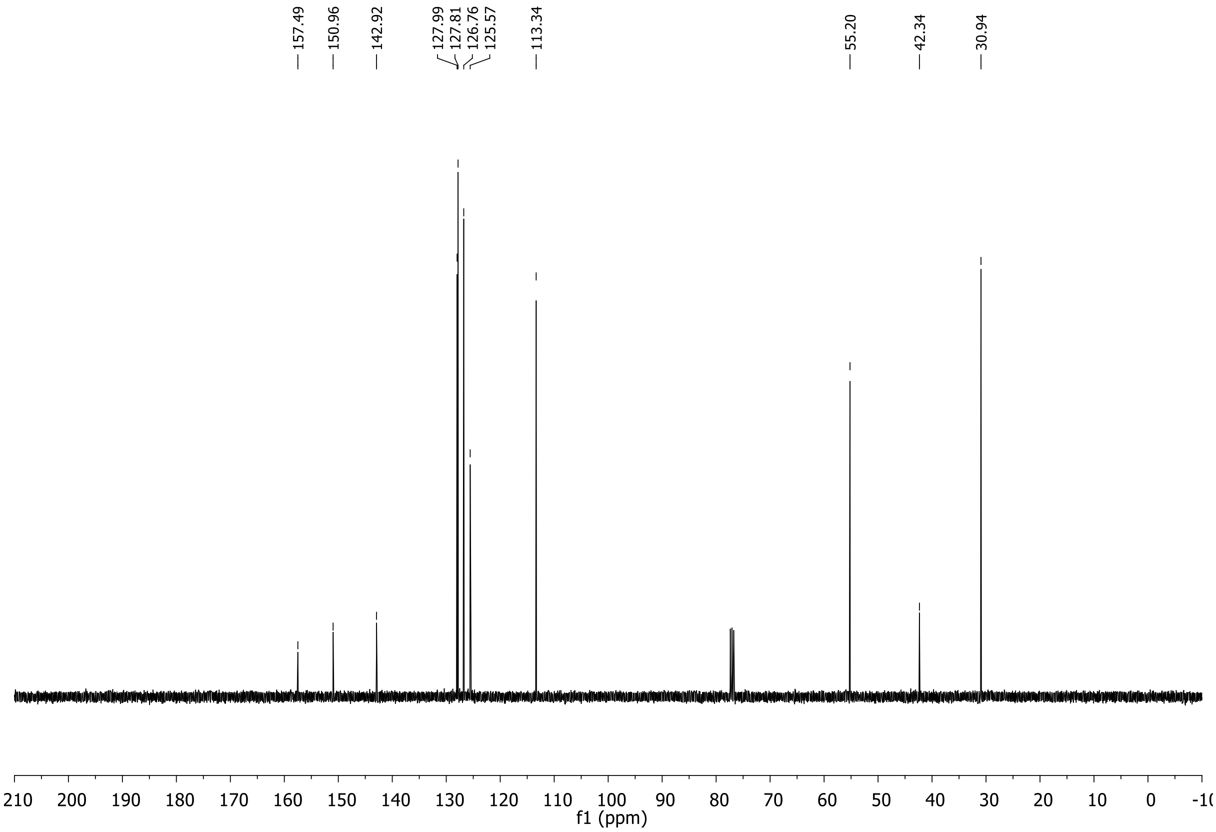

1-ethoxy-4-(2,4,4-trimethylpentan-2-yl)benzene (**41**)

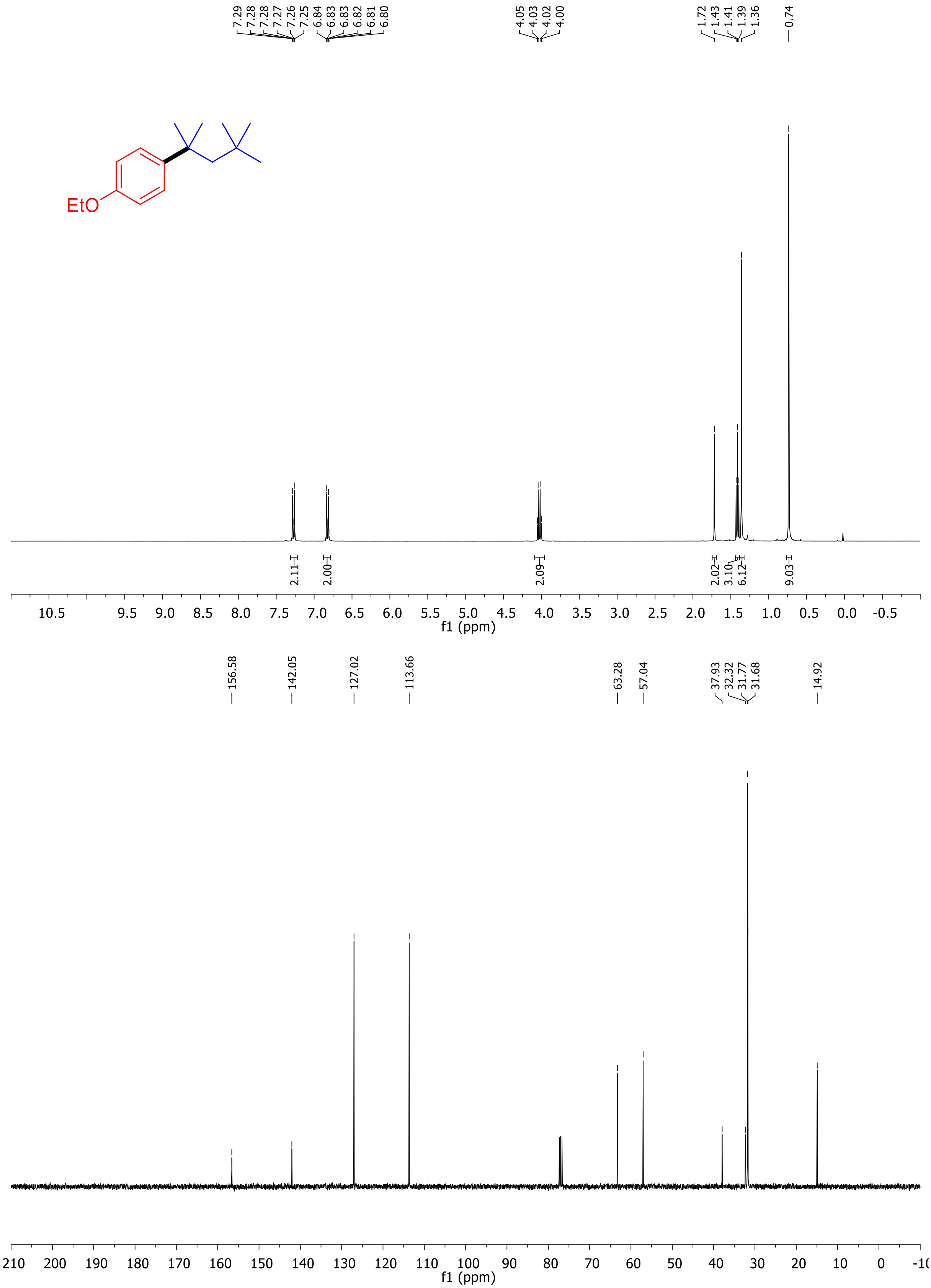

2-Mesitylpropanoic acid (**42**)

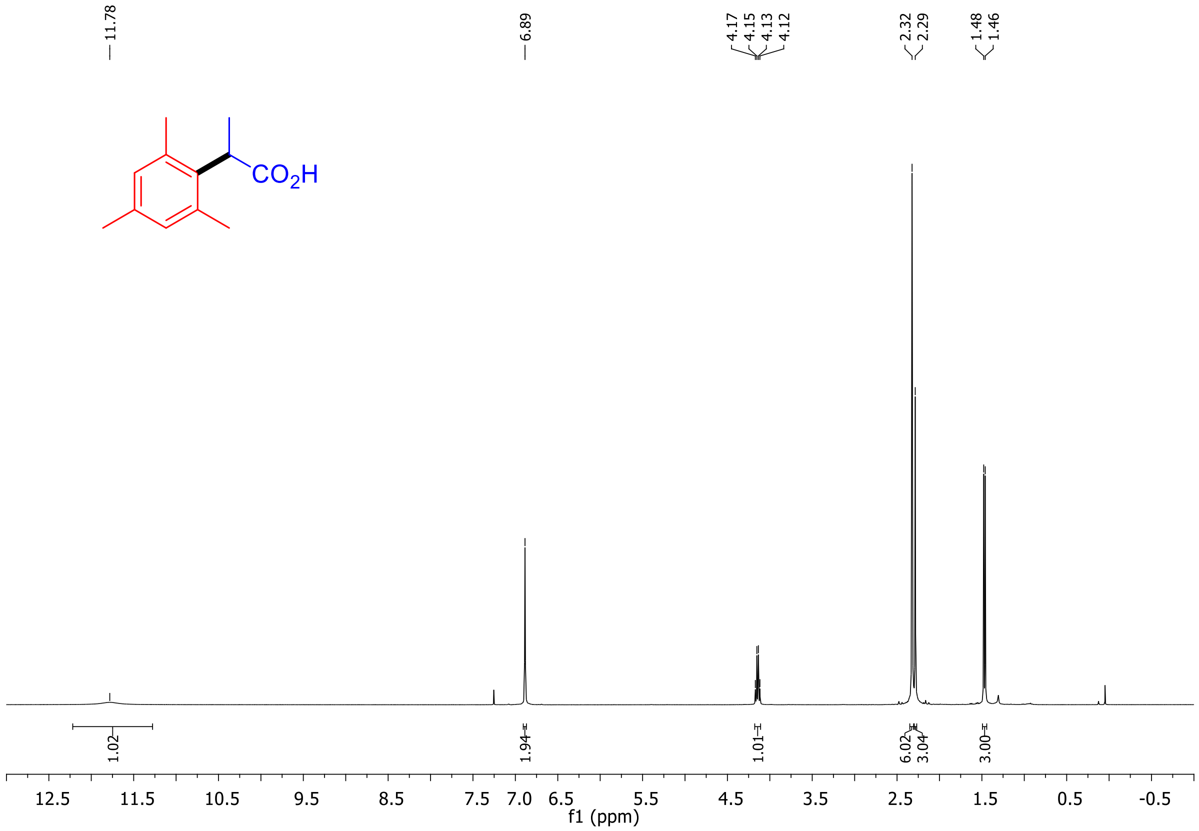

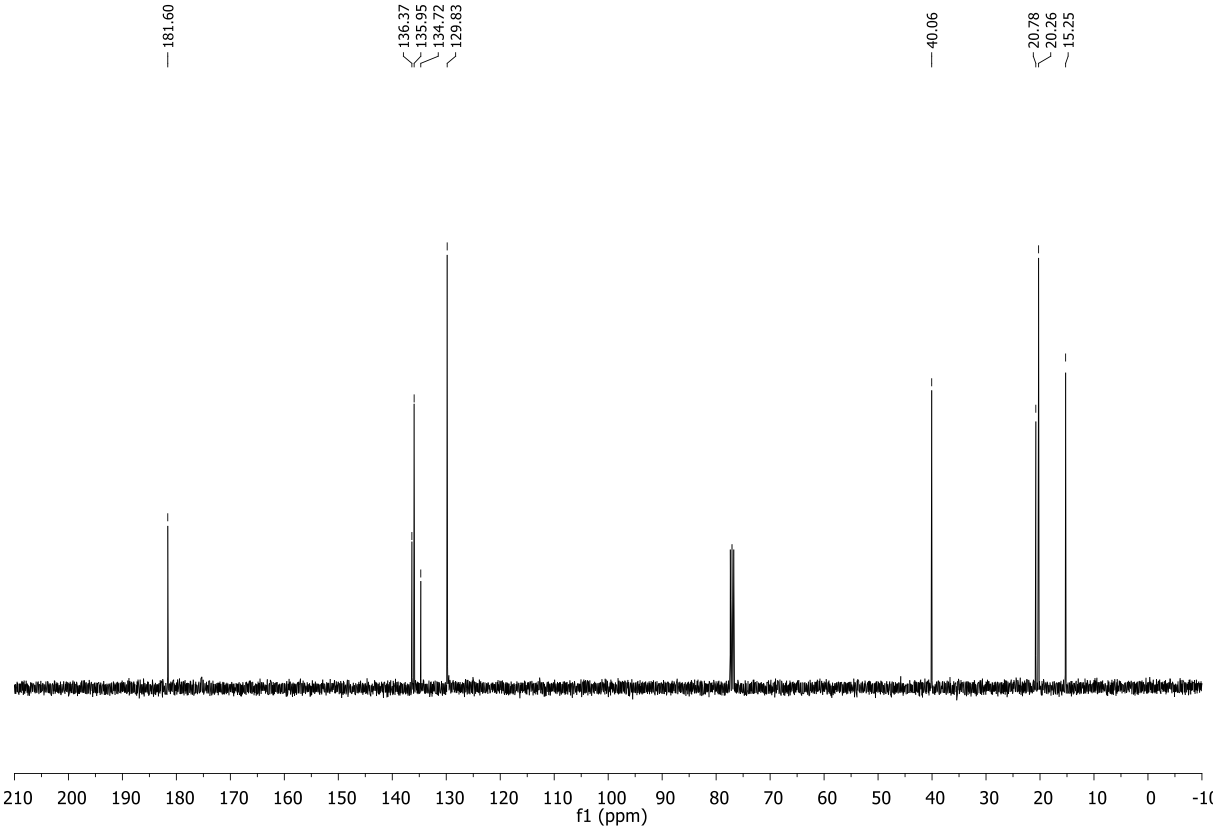

N-((*R*)-1-phenylethyl)-2-mesitylpropanamide (**42a**)

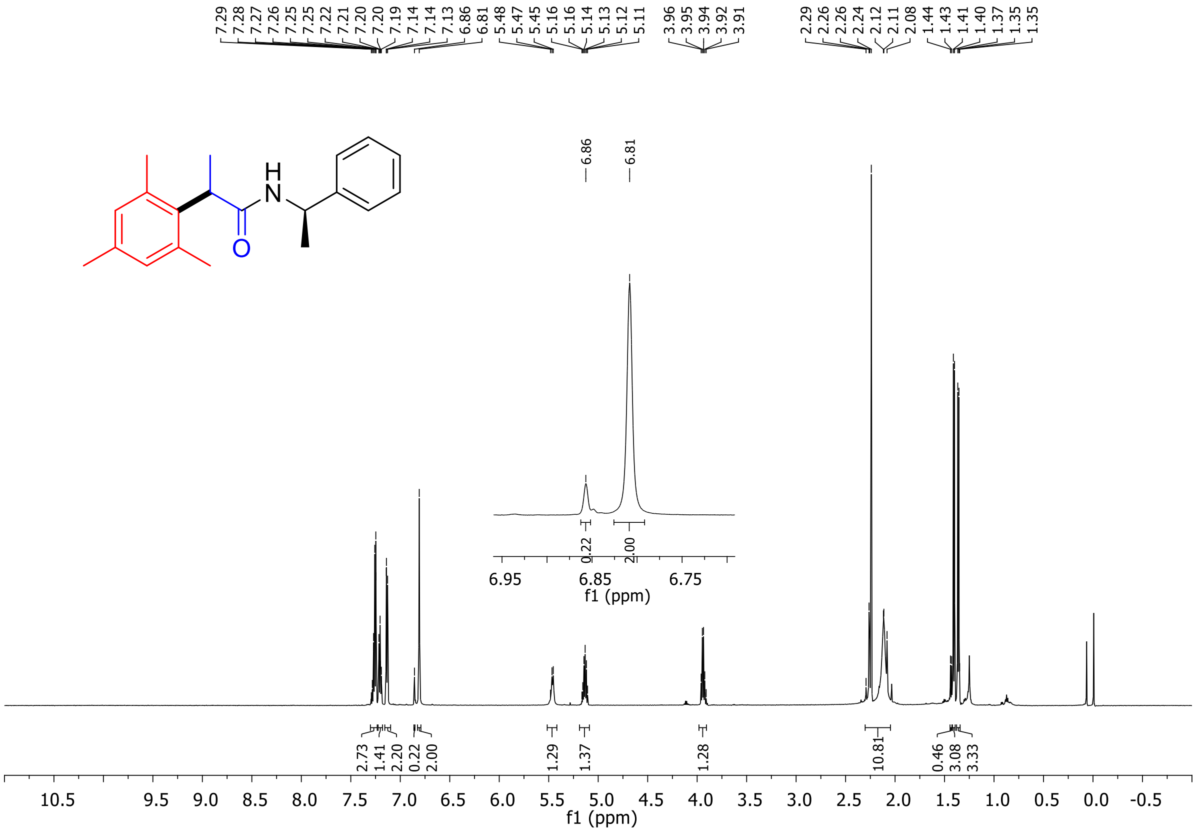

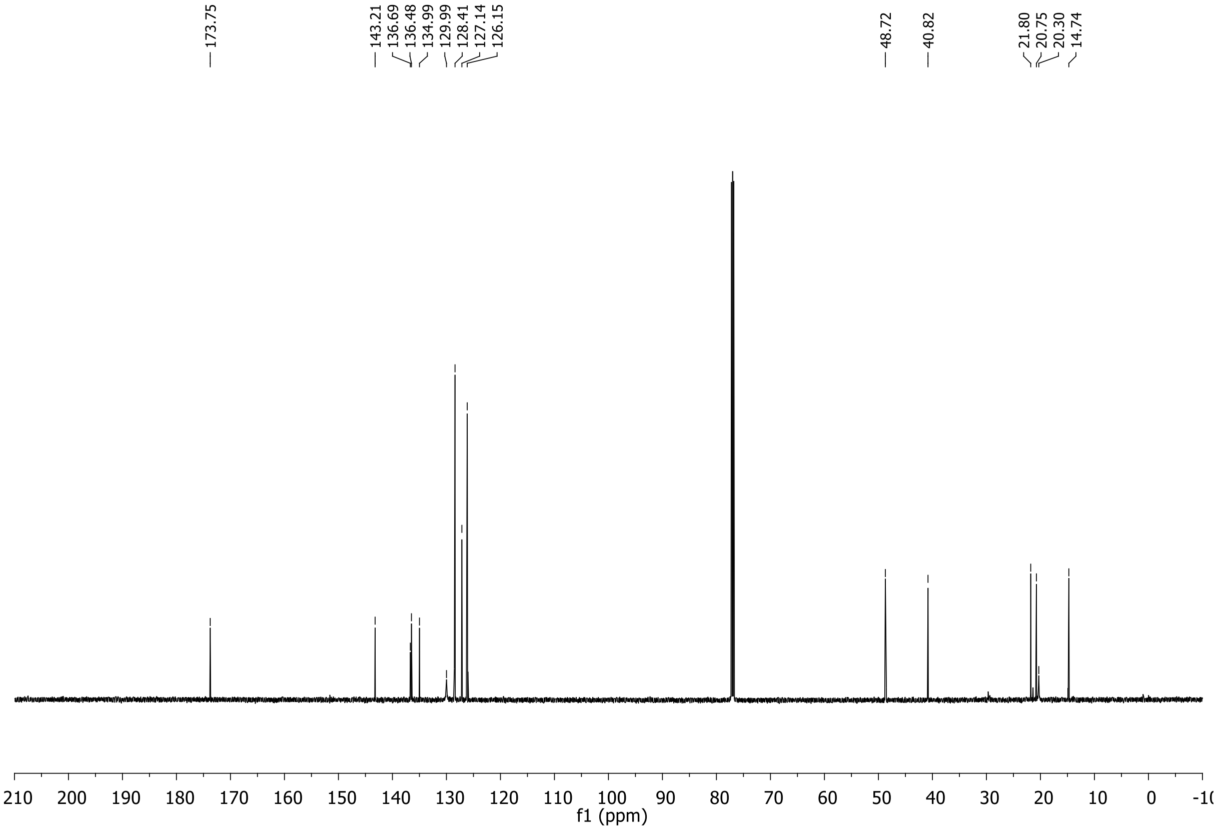

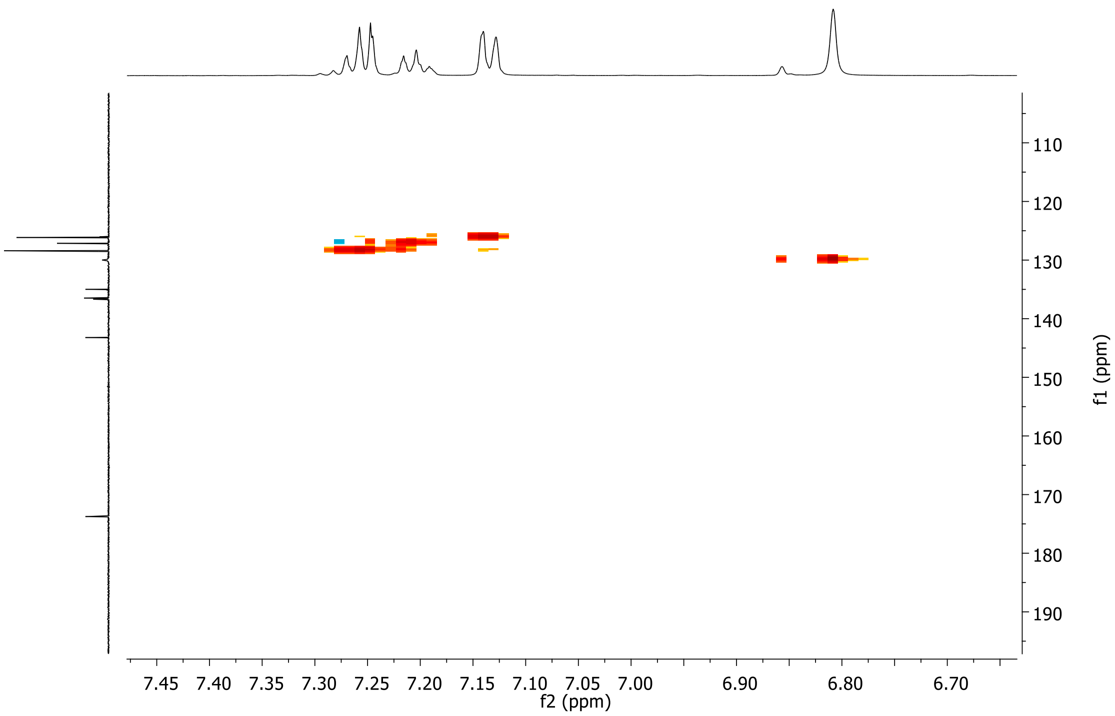

HSQC showing coupling between that small peaks on ^{1}H spectra and aromatic peaks on ^{13}C spectra, thus proving that the small peaks on ^{1}H spectra represent the minor diastereoisomer and not some impurity.

2-Mesityl-2-phenylacetic acid (**43**)

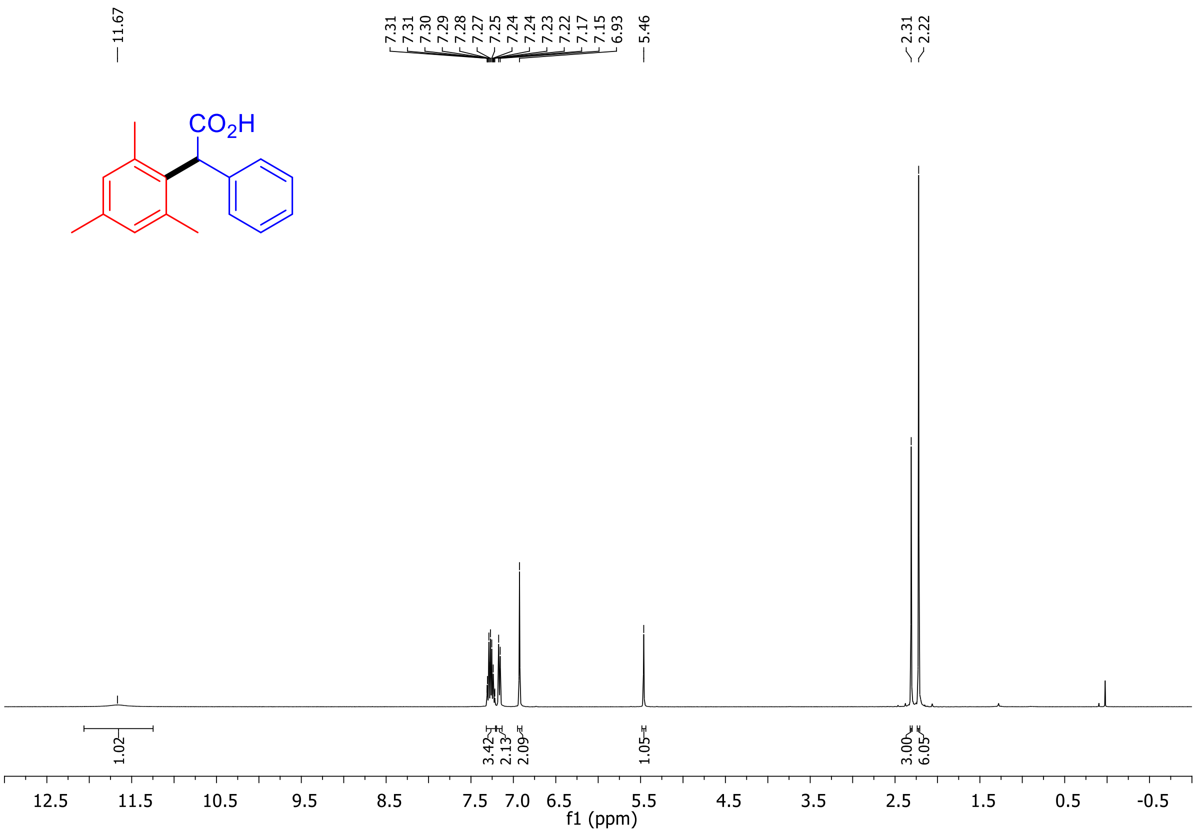

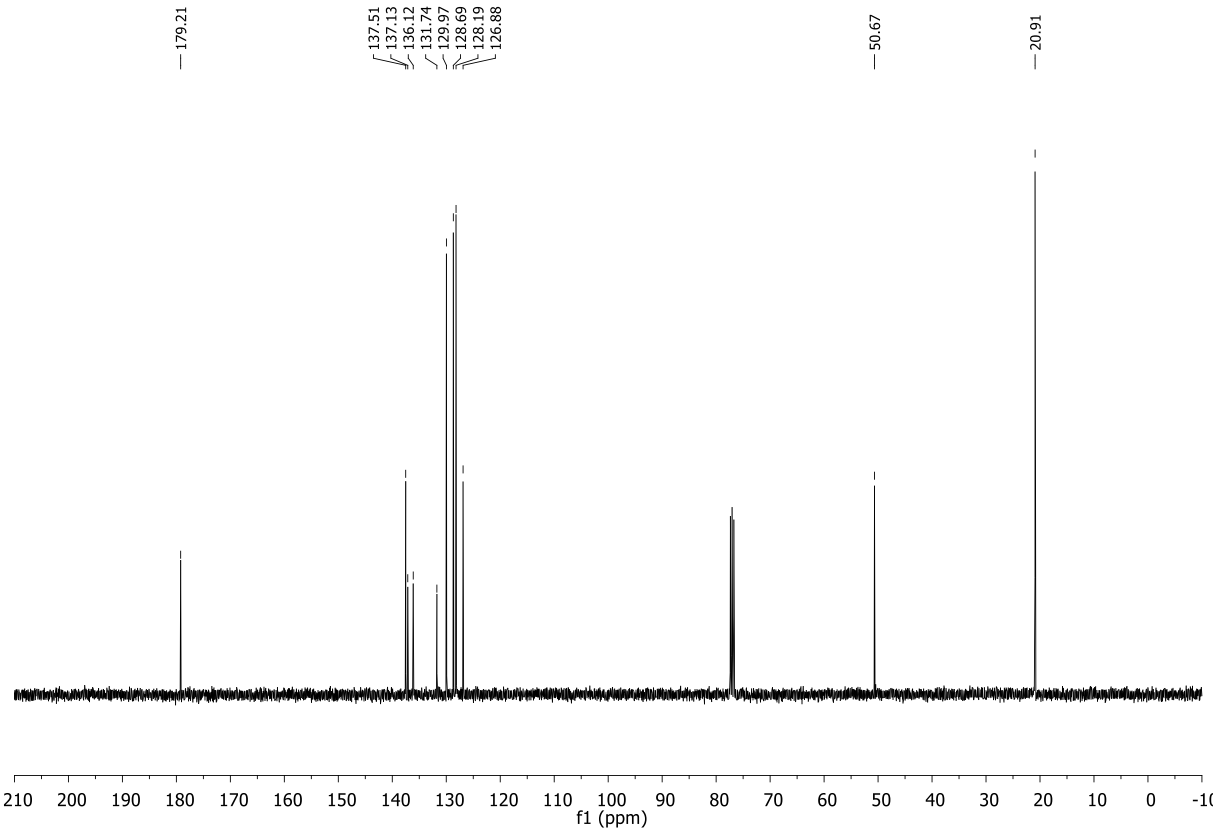

N-((*R*)-1-phenylethyl)-2-mesityl-2-phenylacetamide (**43a**)

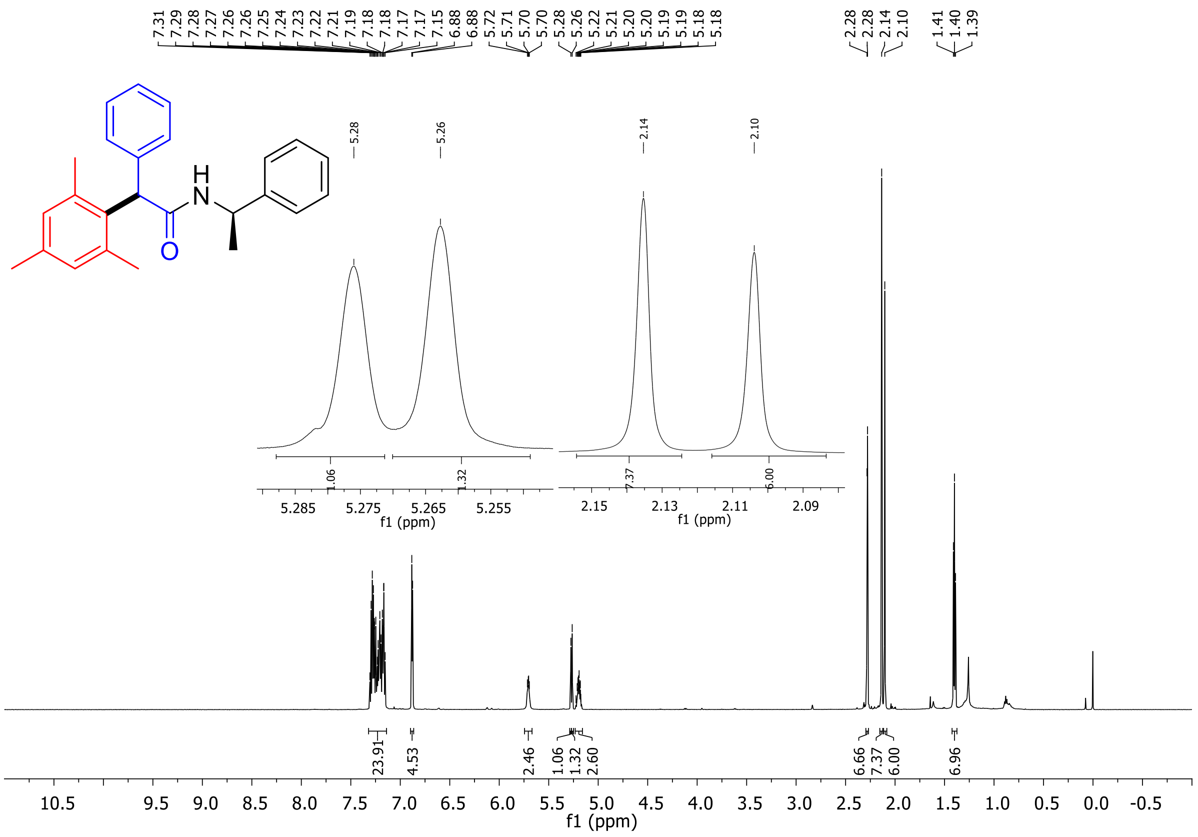

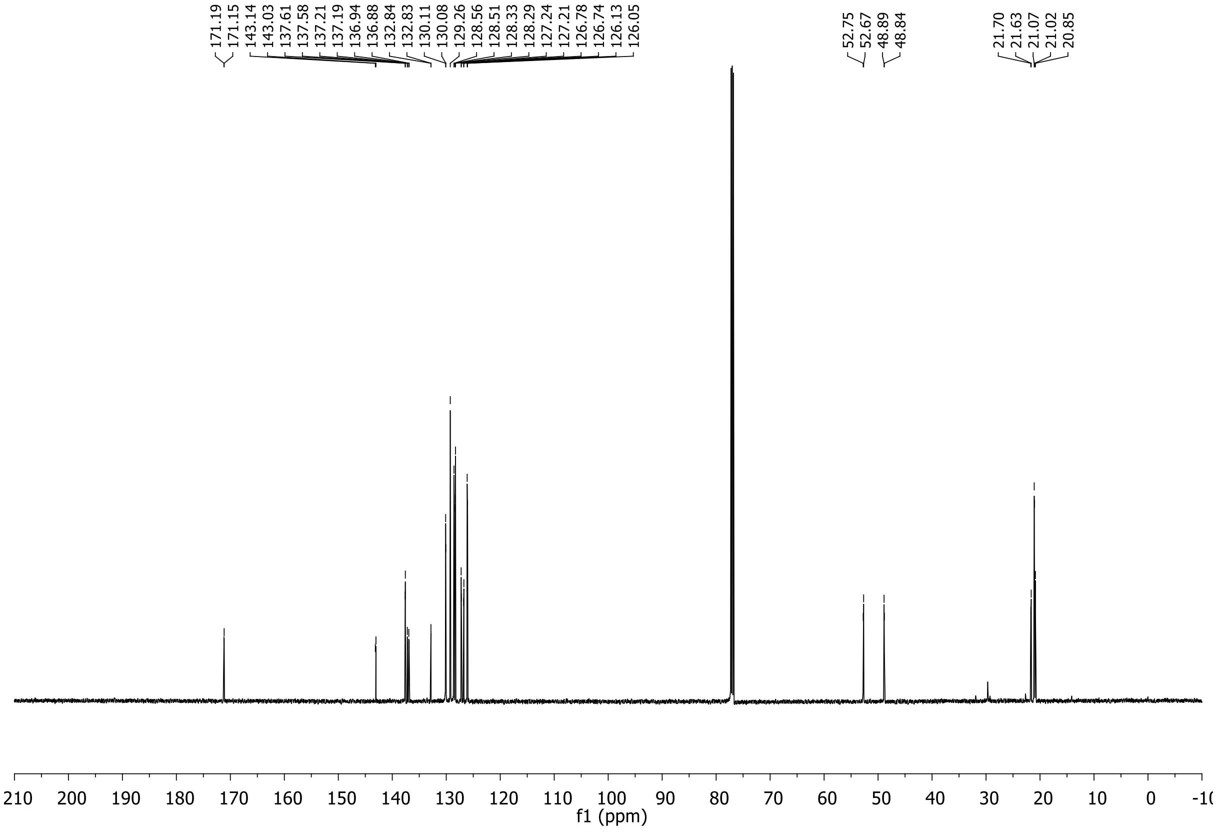

4-Nitrobiphenyl (**44**)

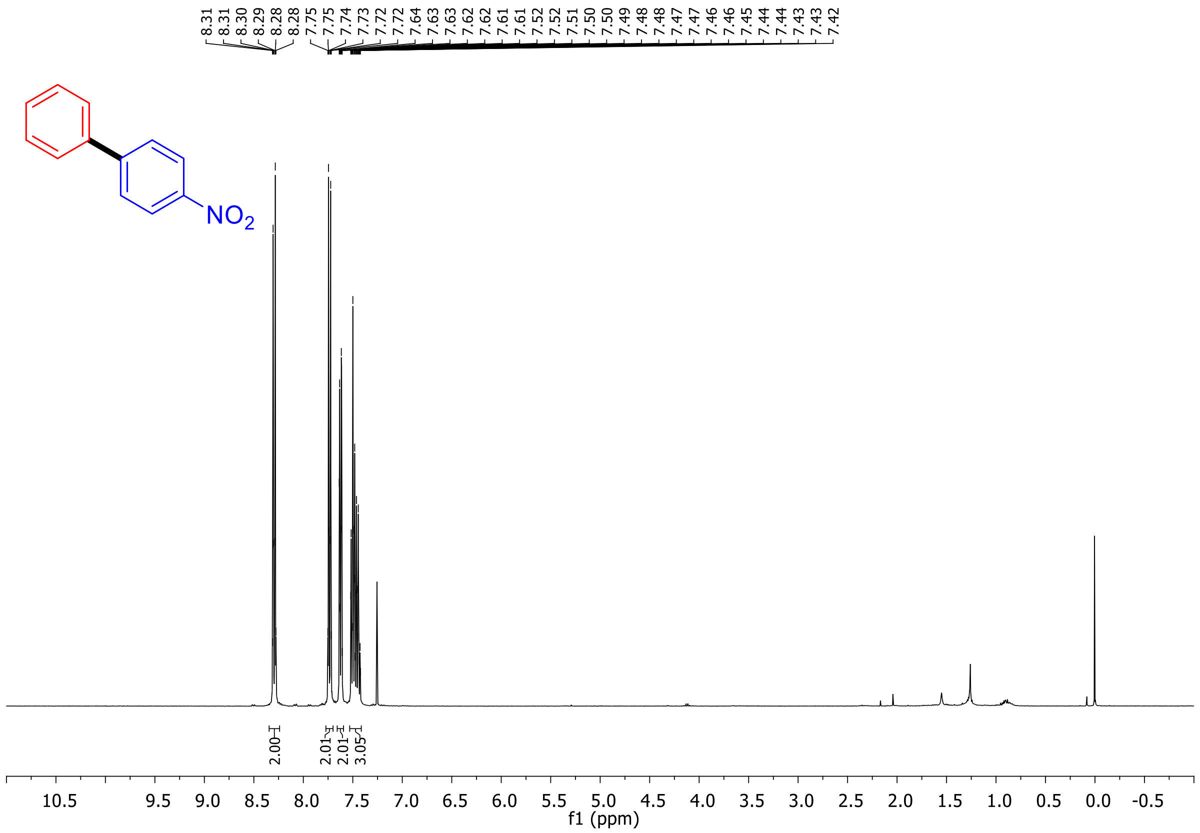

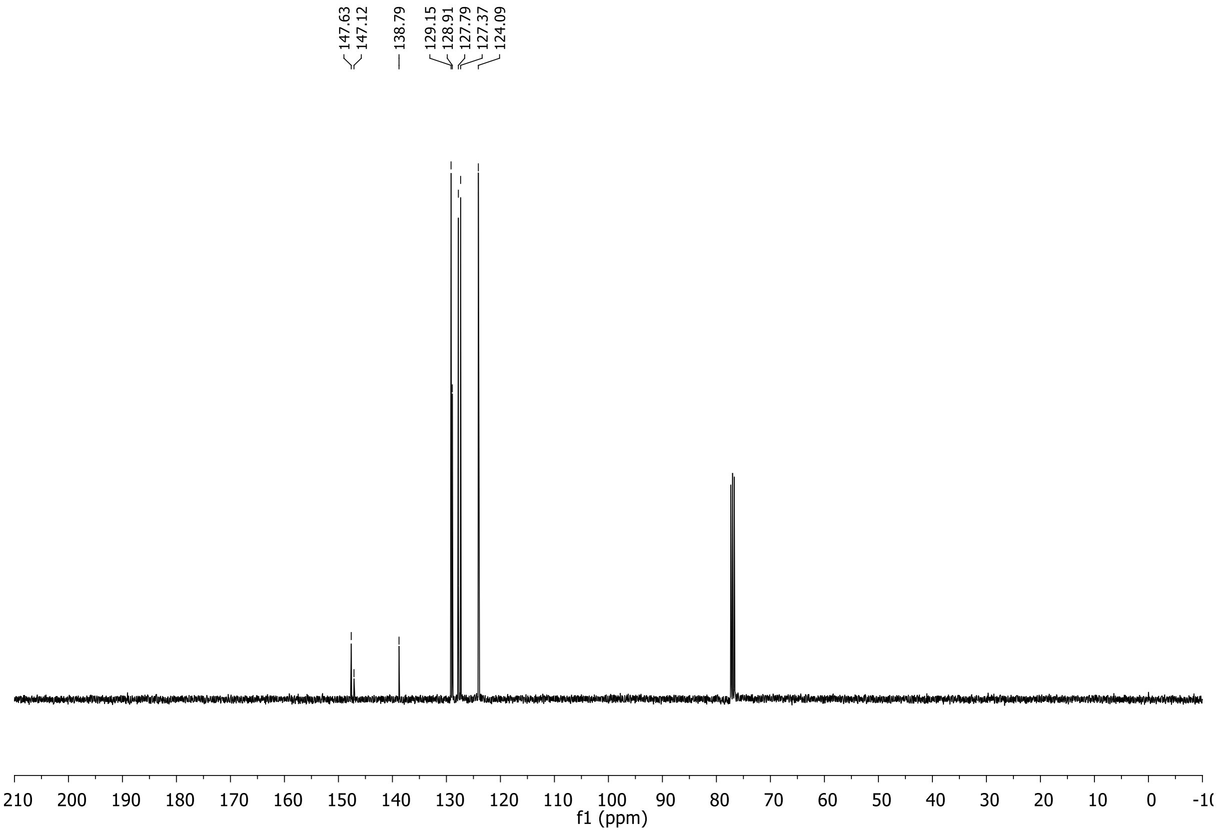

4-Hydroxyazobenzene (**45**)

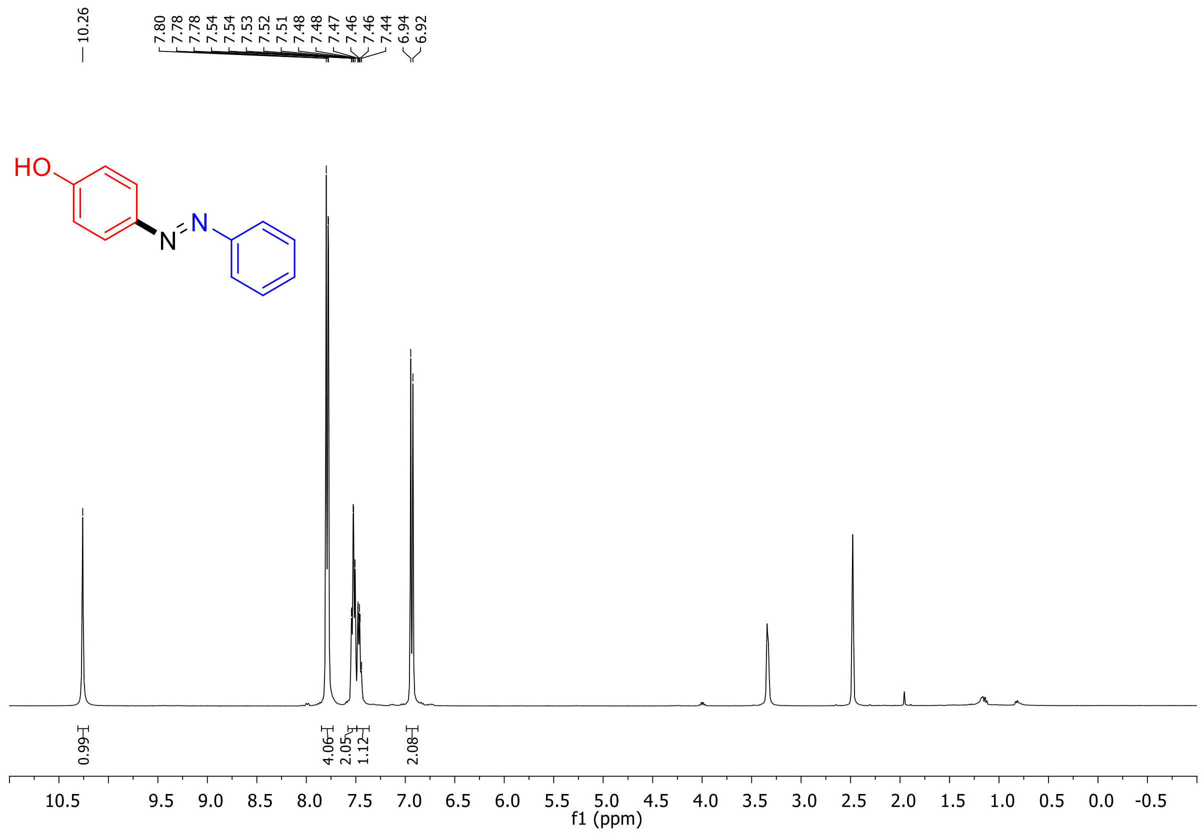

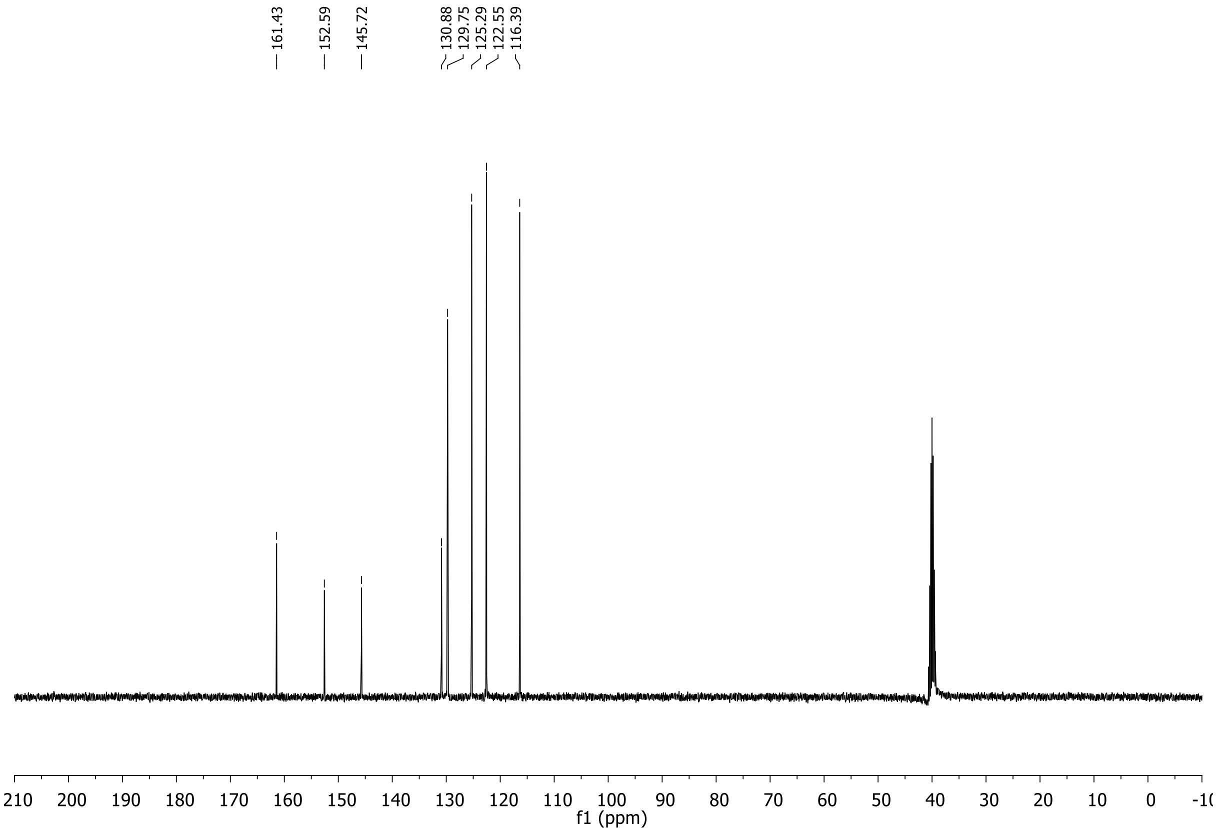

Methyl red (**46**)

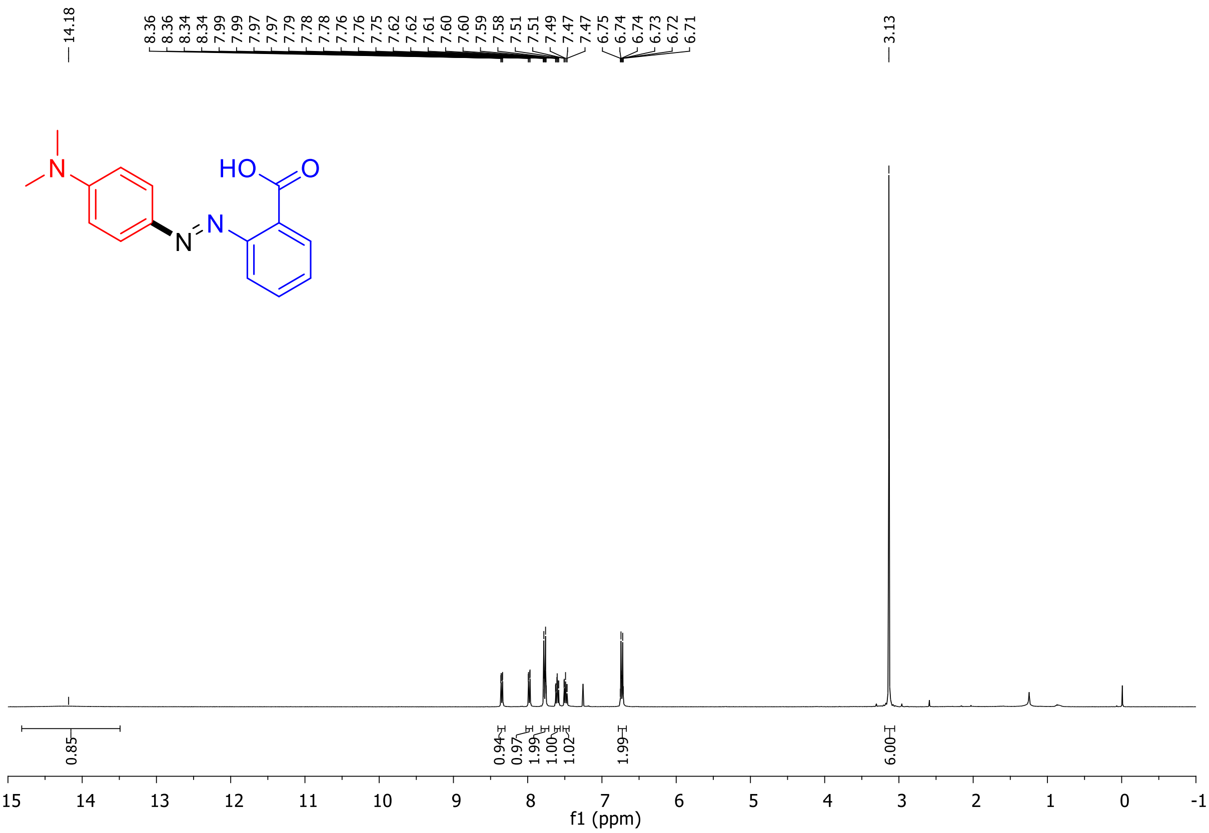

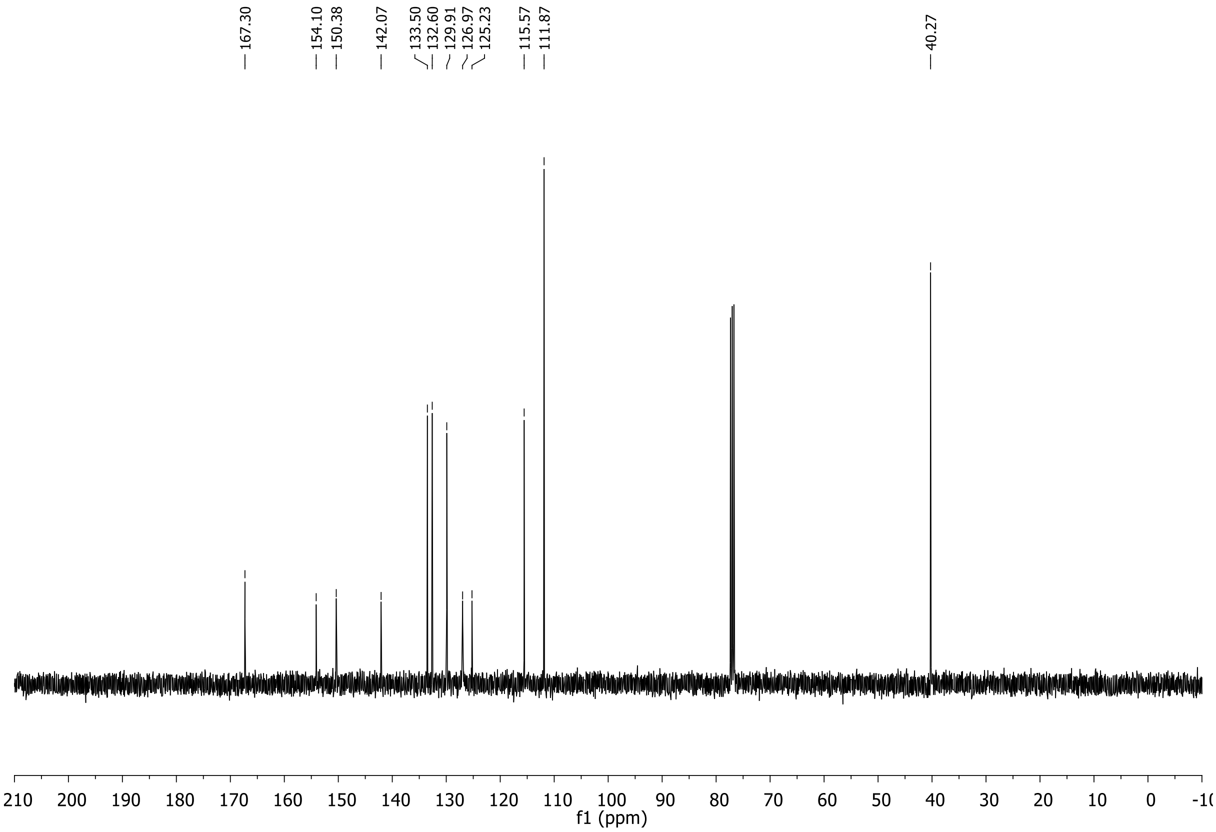

Diphenylmethane (**47**)

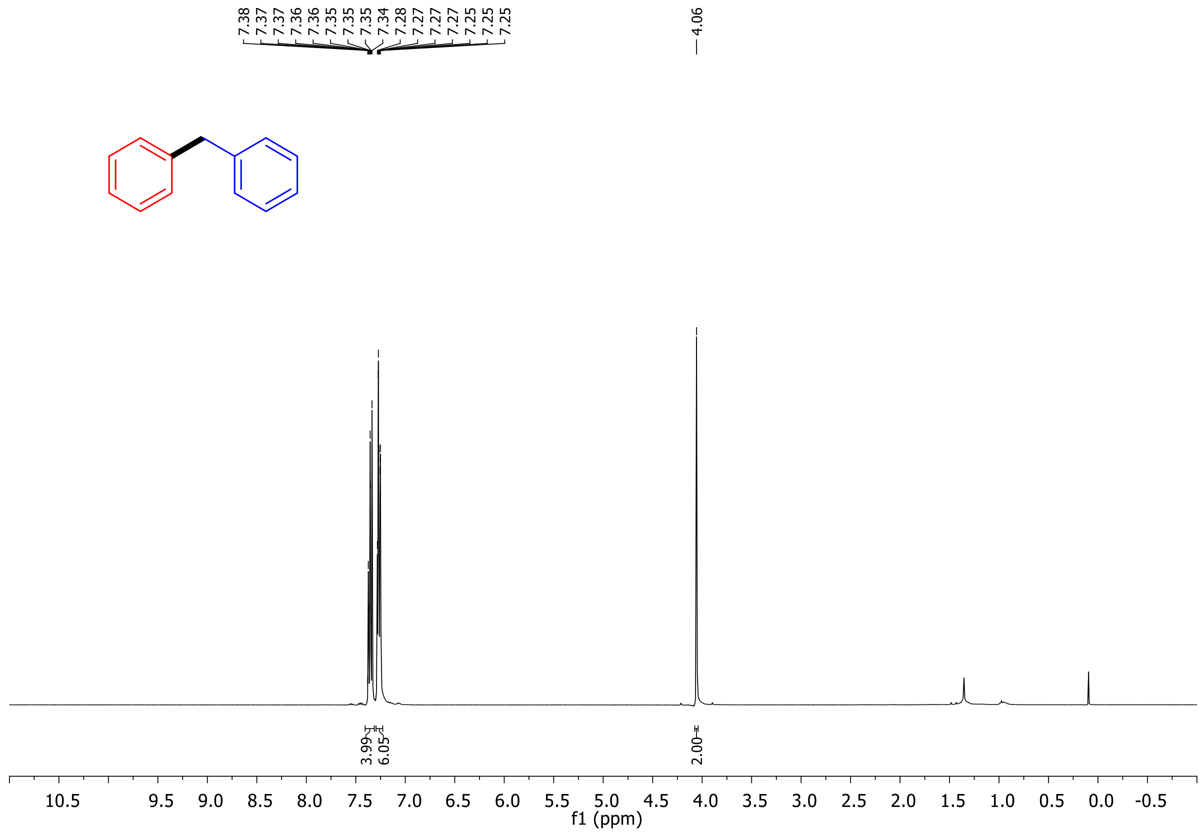

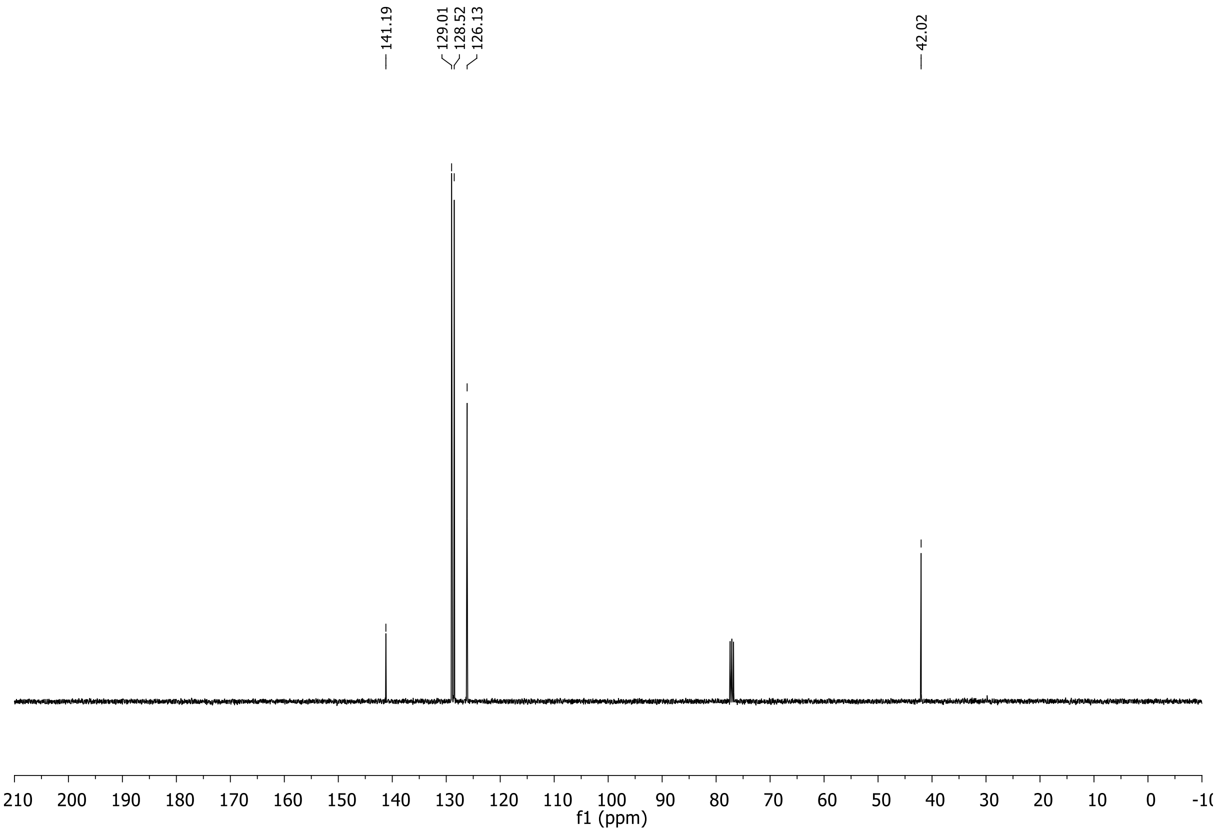

2-Benzyl-1,4-dimethylbenzene (**8**)

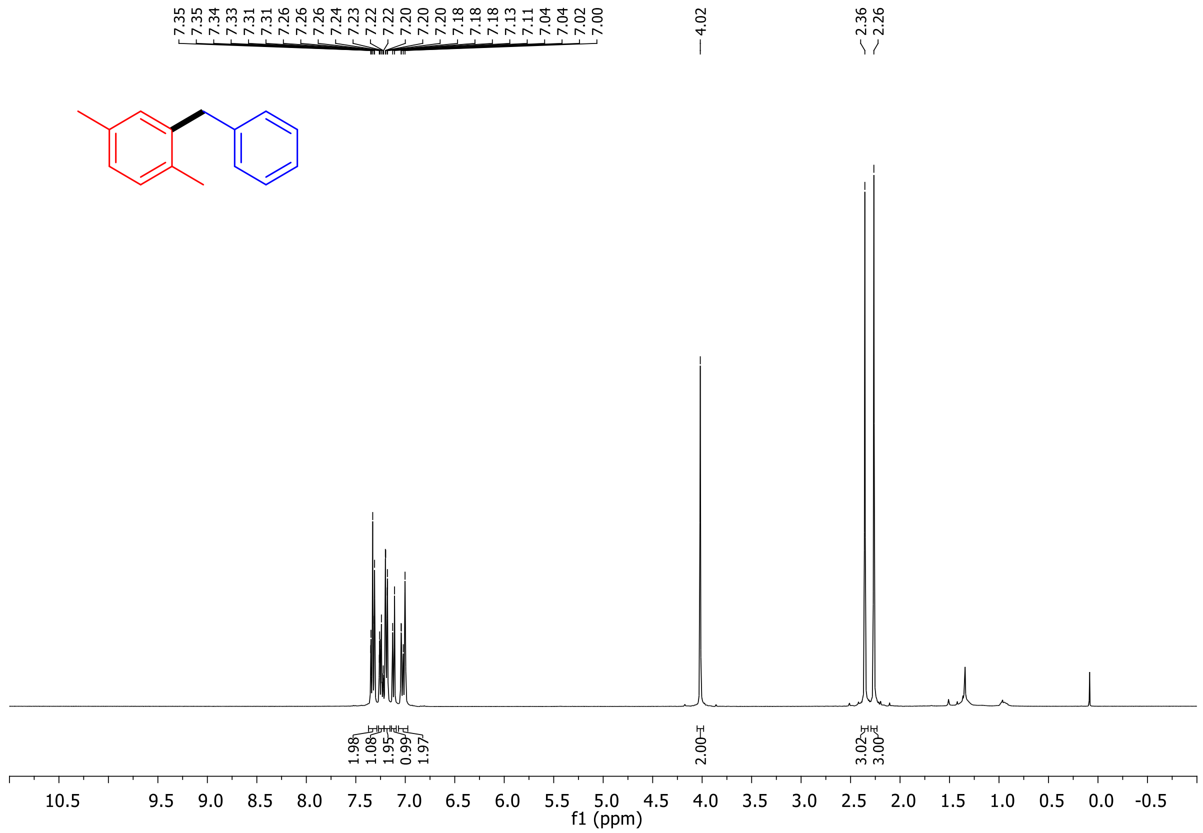

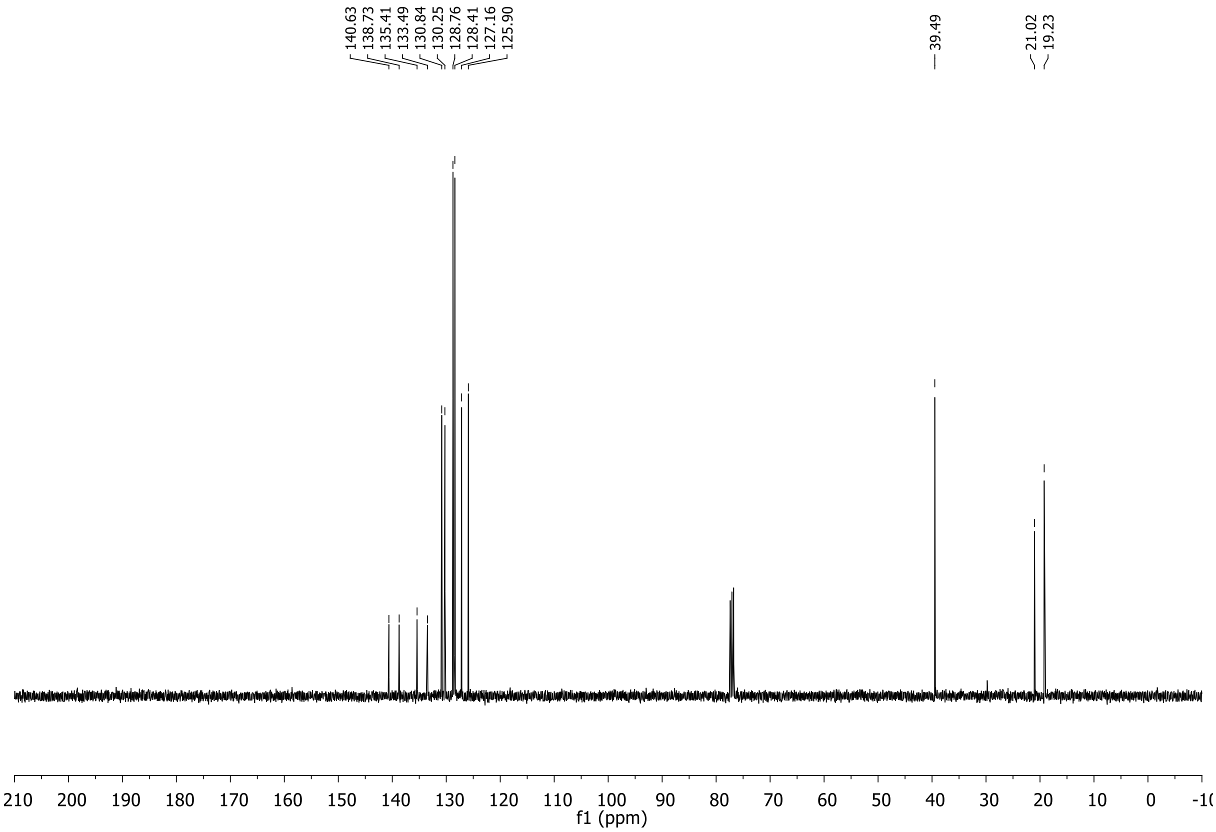

Benzylpentamethylbenzene (**48**)

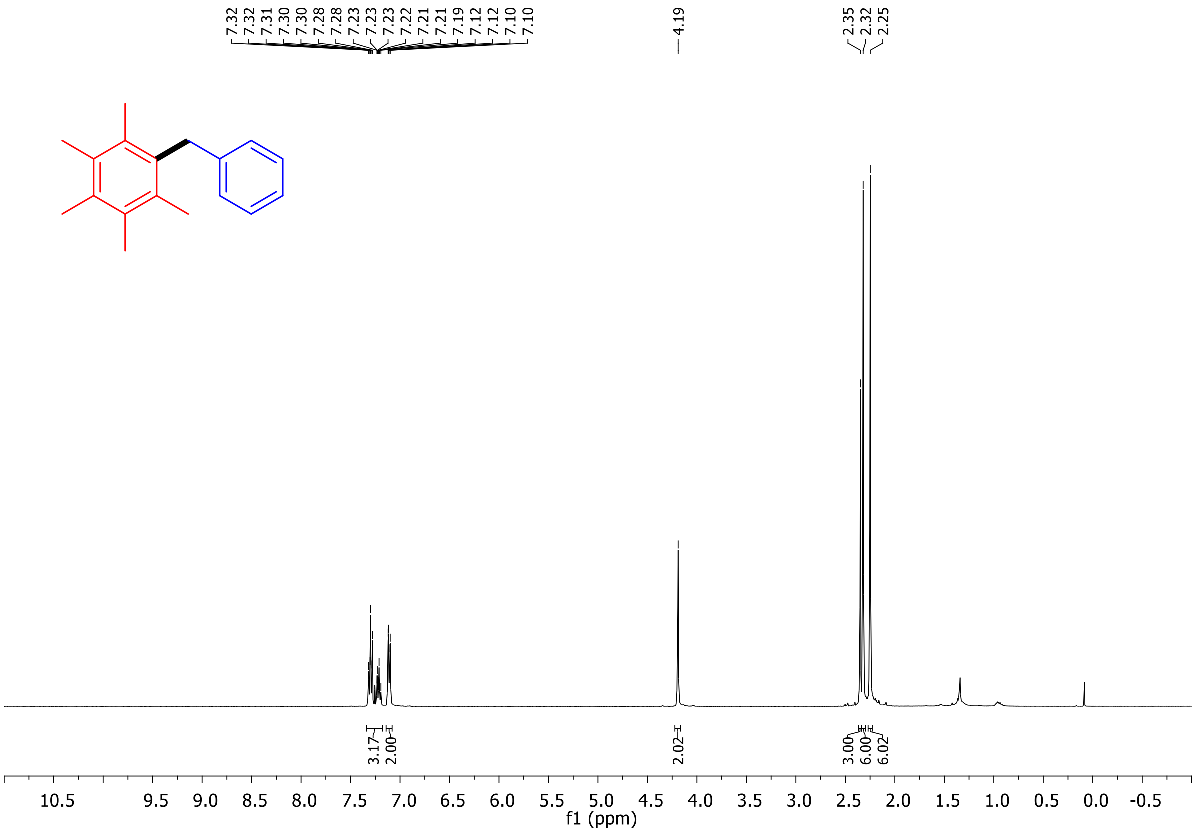

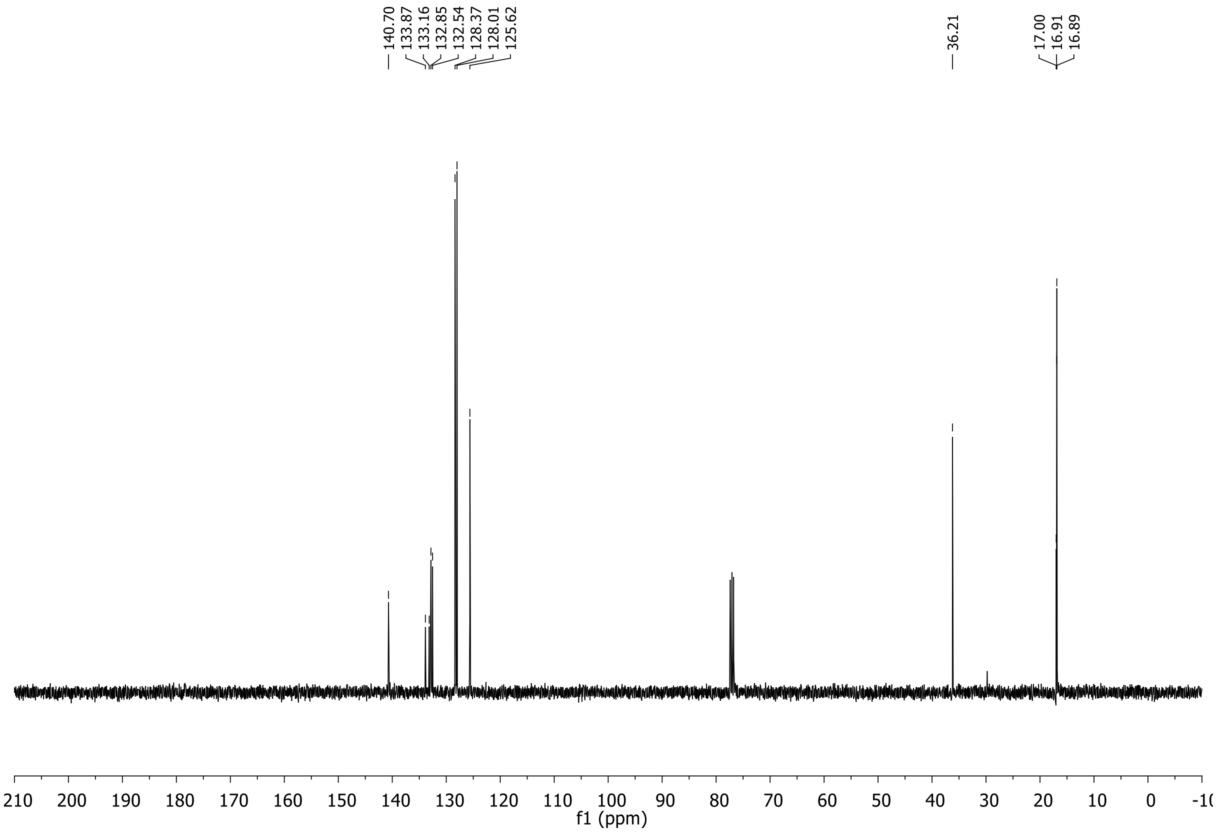

4-(2-Methylbenzyl)-tert-butylbenzene (**49**)

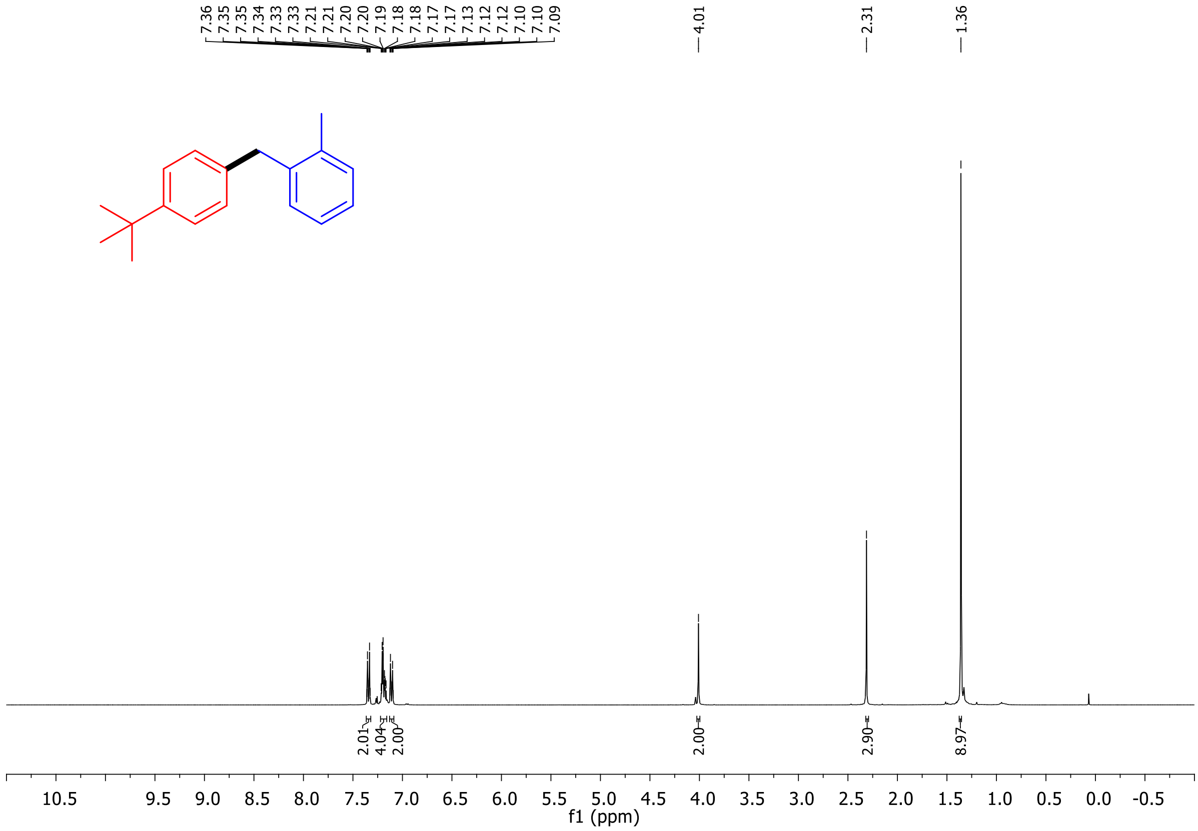

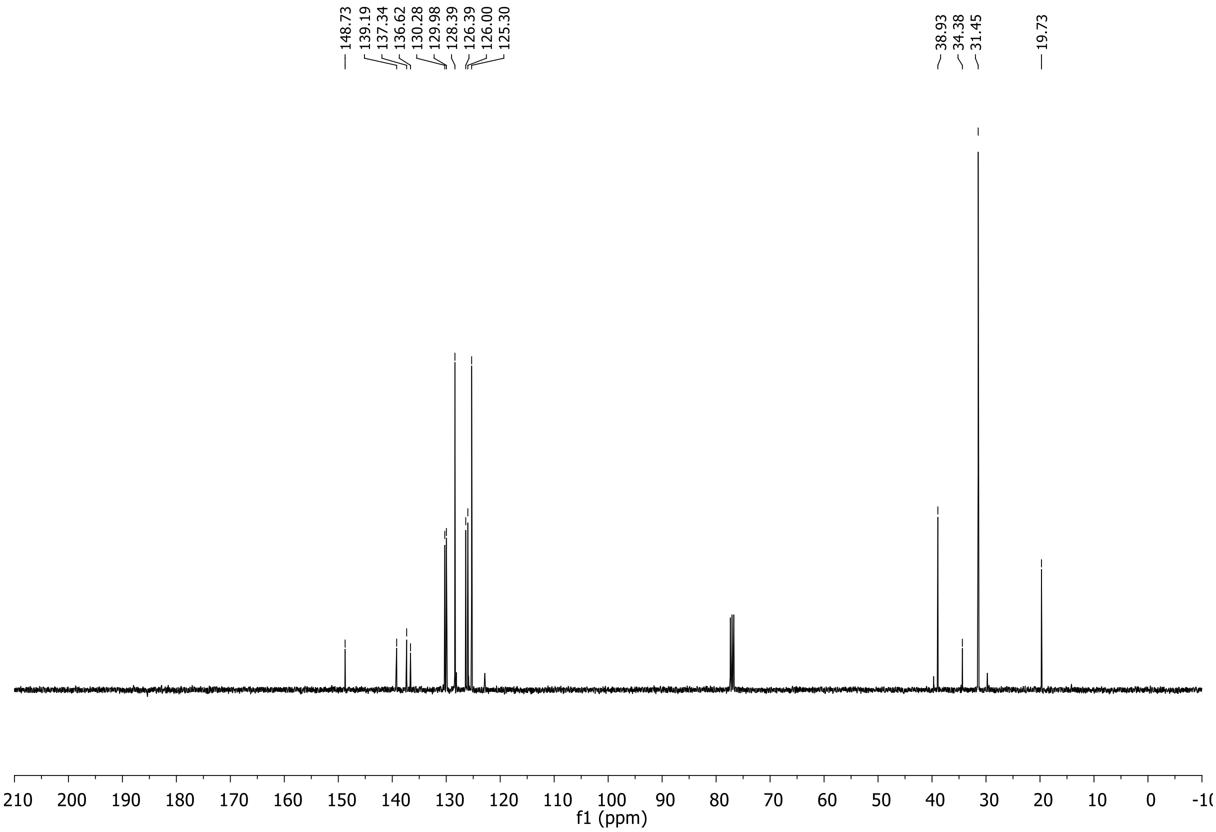

(4-Bromobenzyl)benzene (**50**)

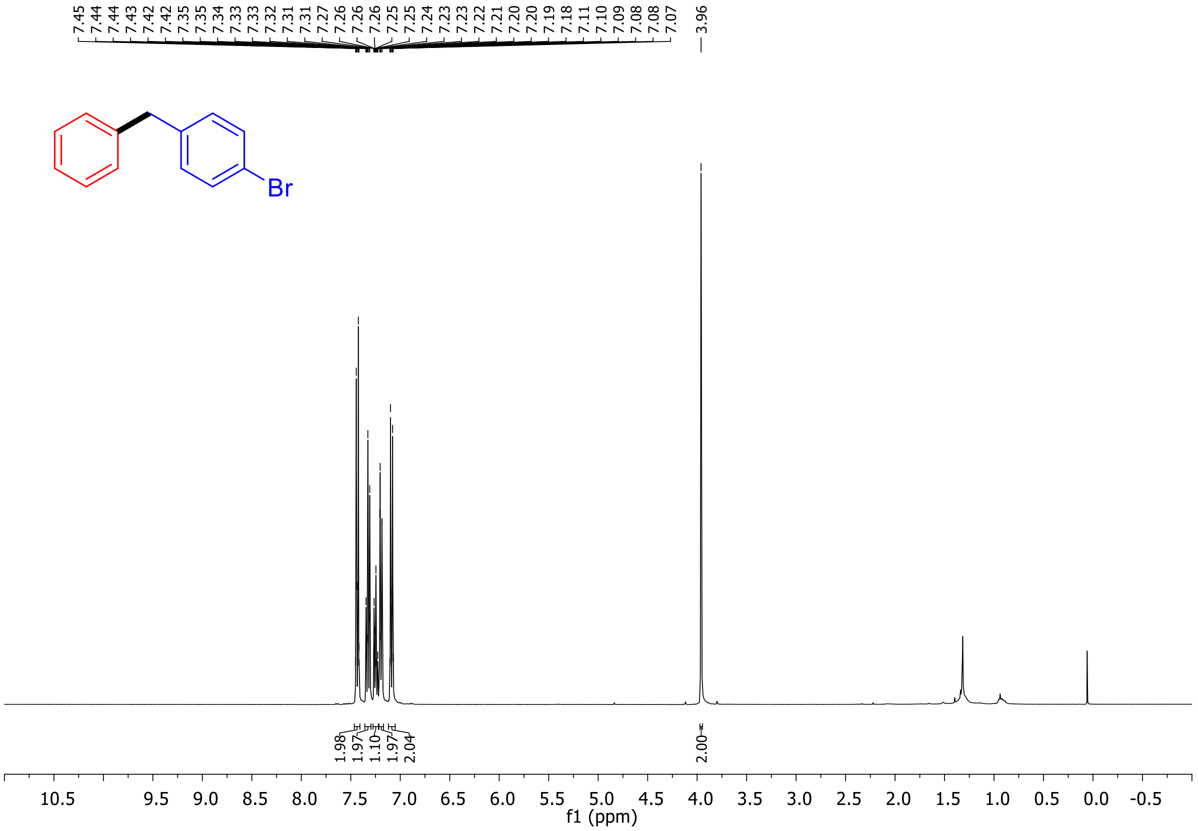

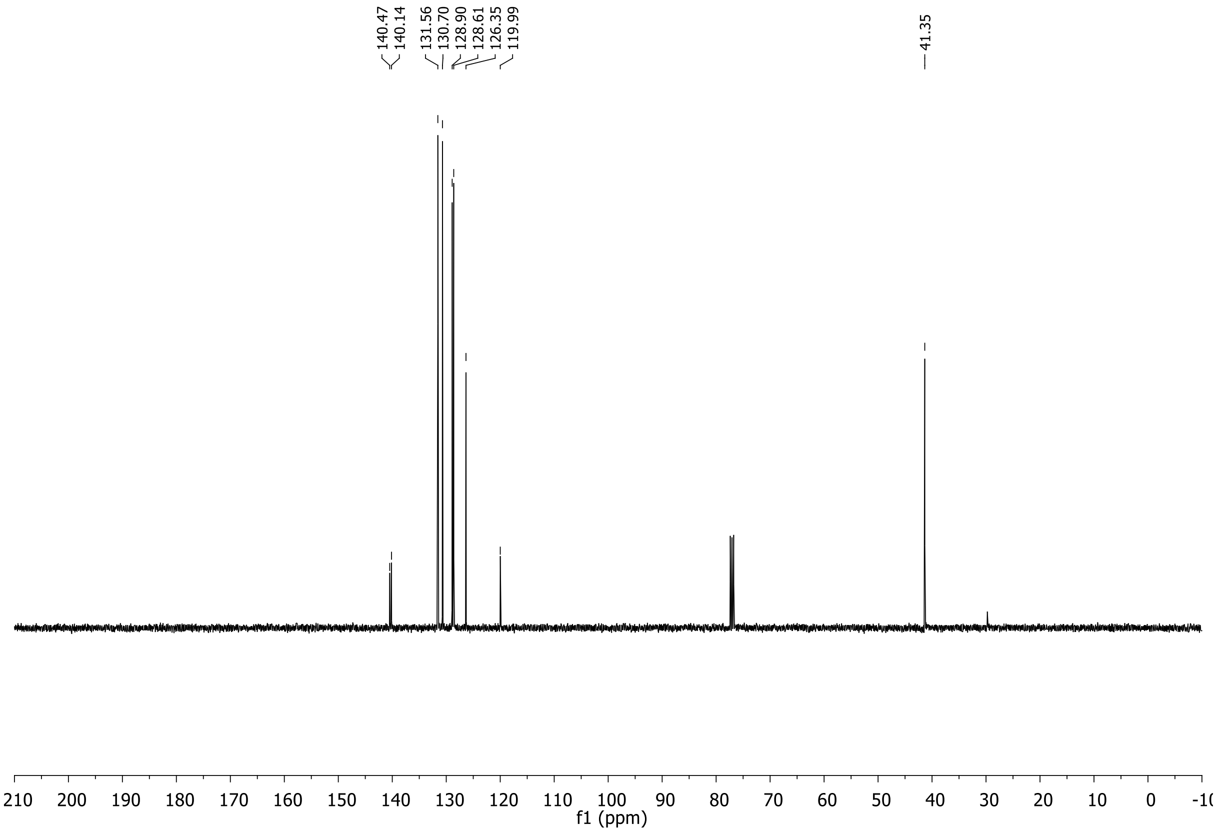

4-(4-Bromobenzyl)-1,3-difluorobenzene (**51**)

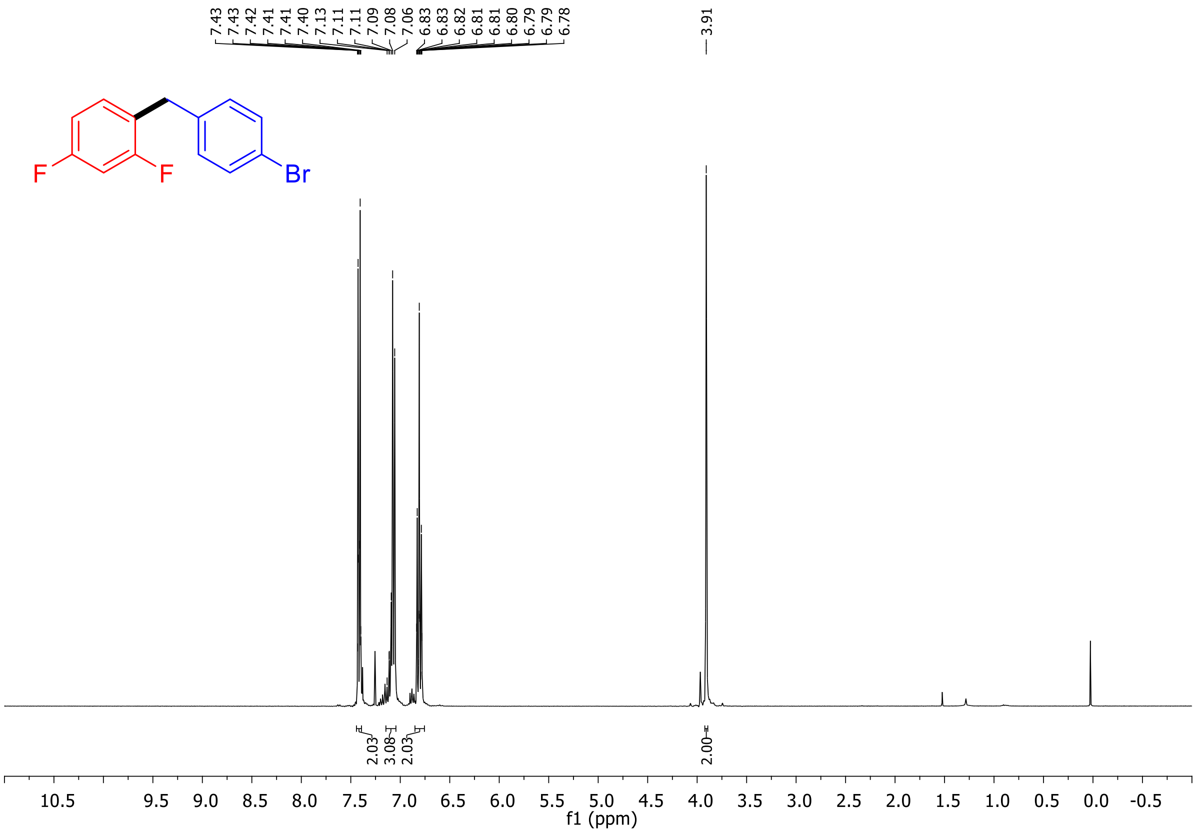

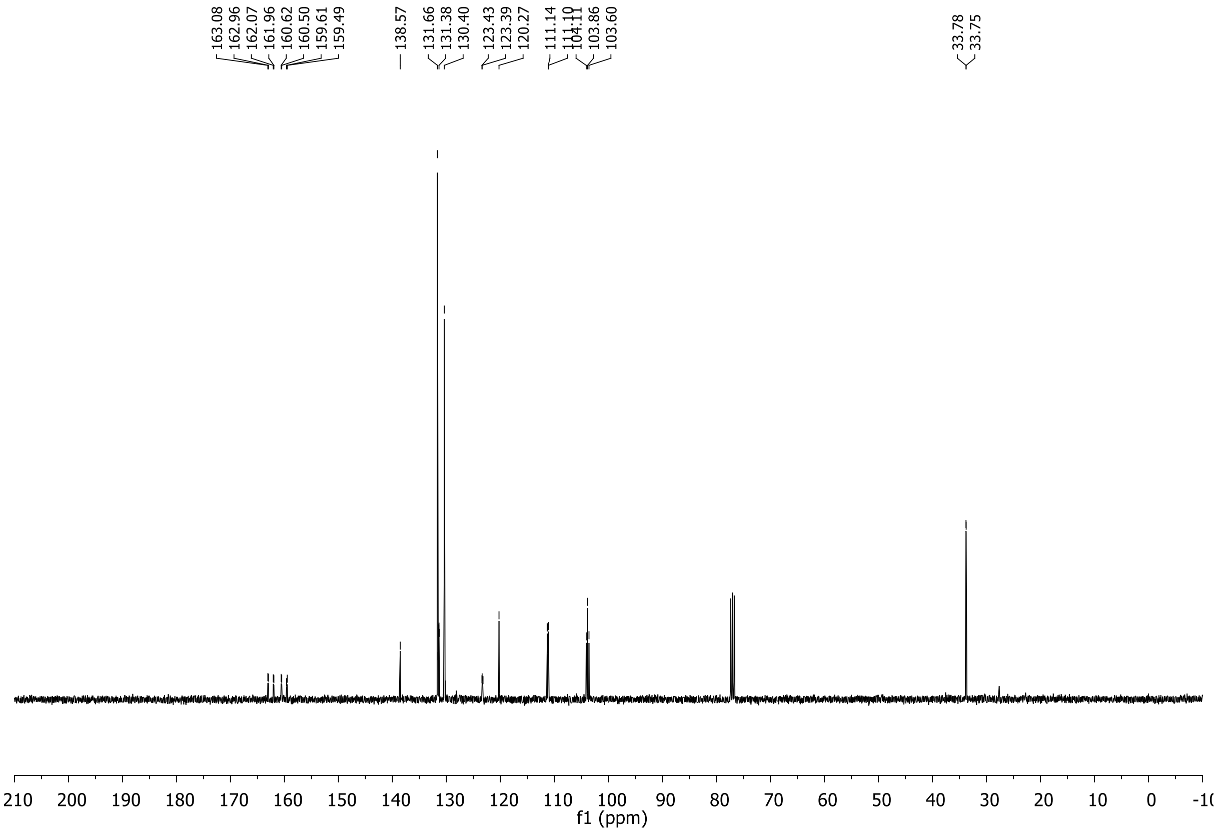

4,4'-Dianisylmethane (**52**)

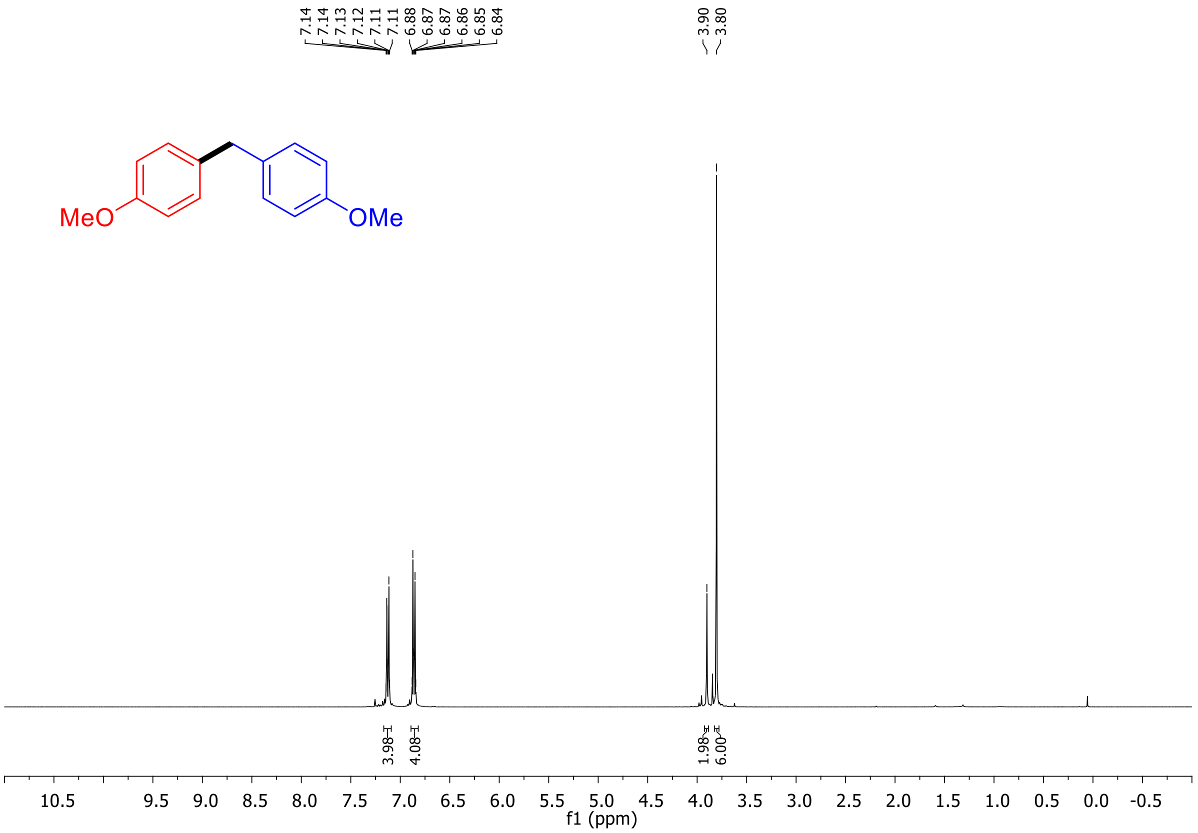

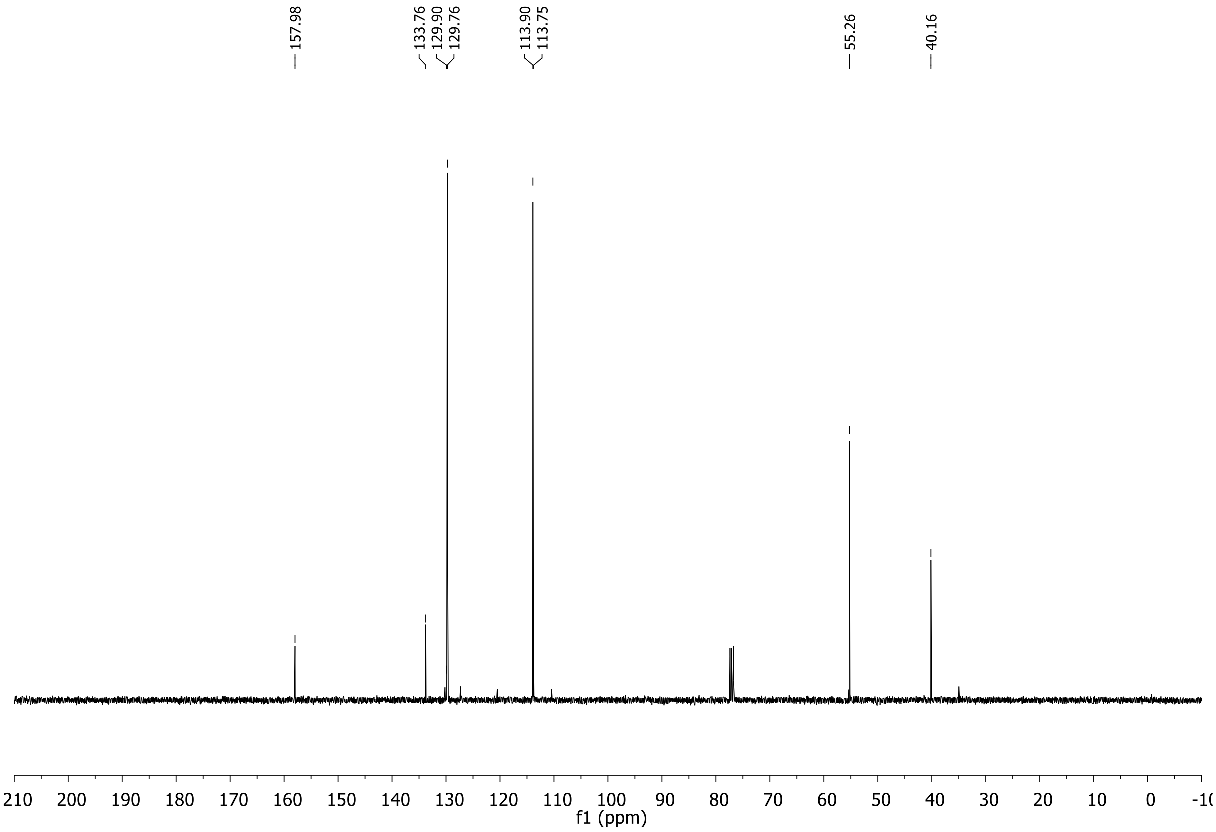

1-(4-Methoxyphenyl)adamantane (**53**)

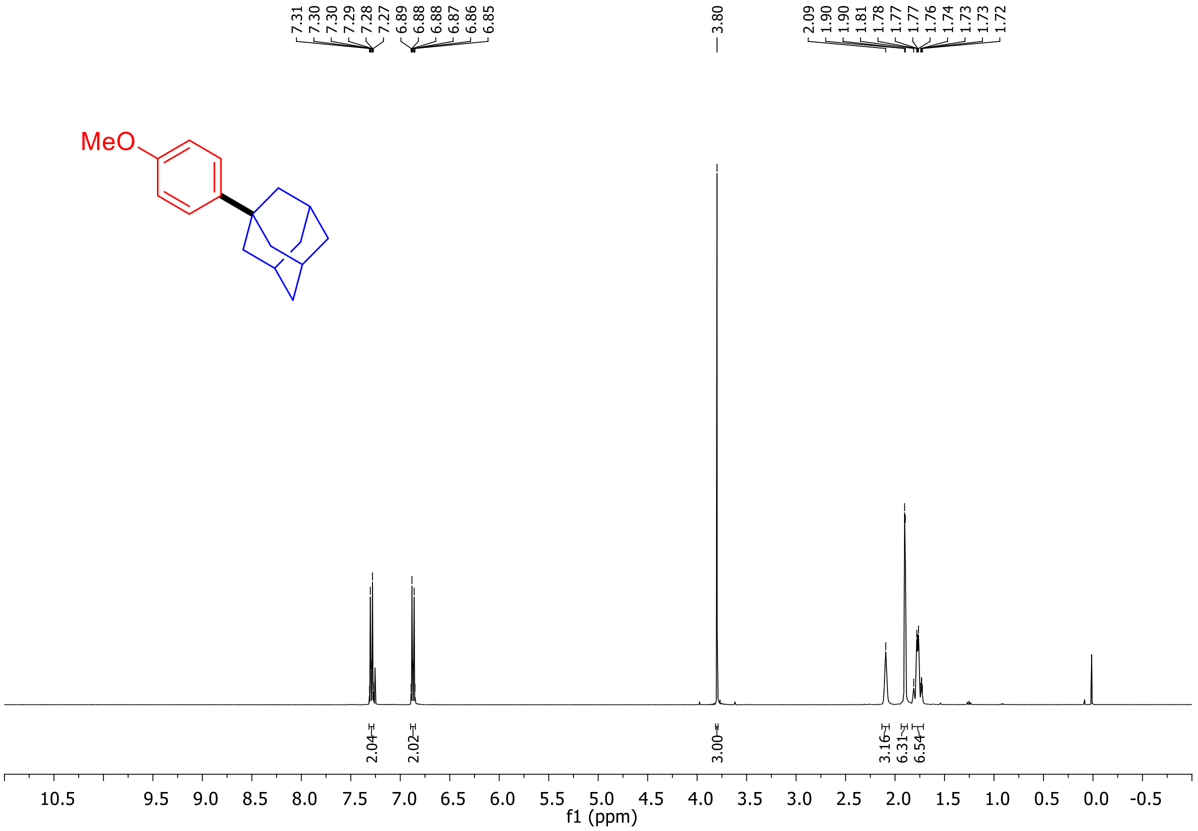

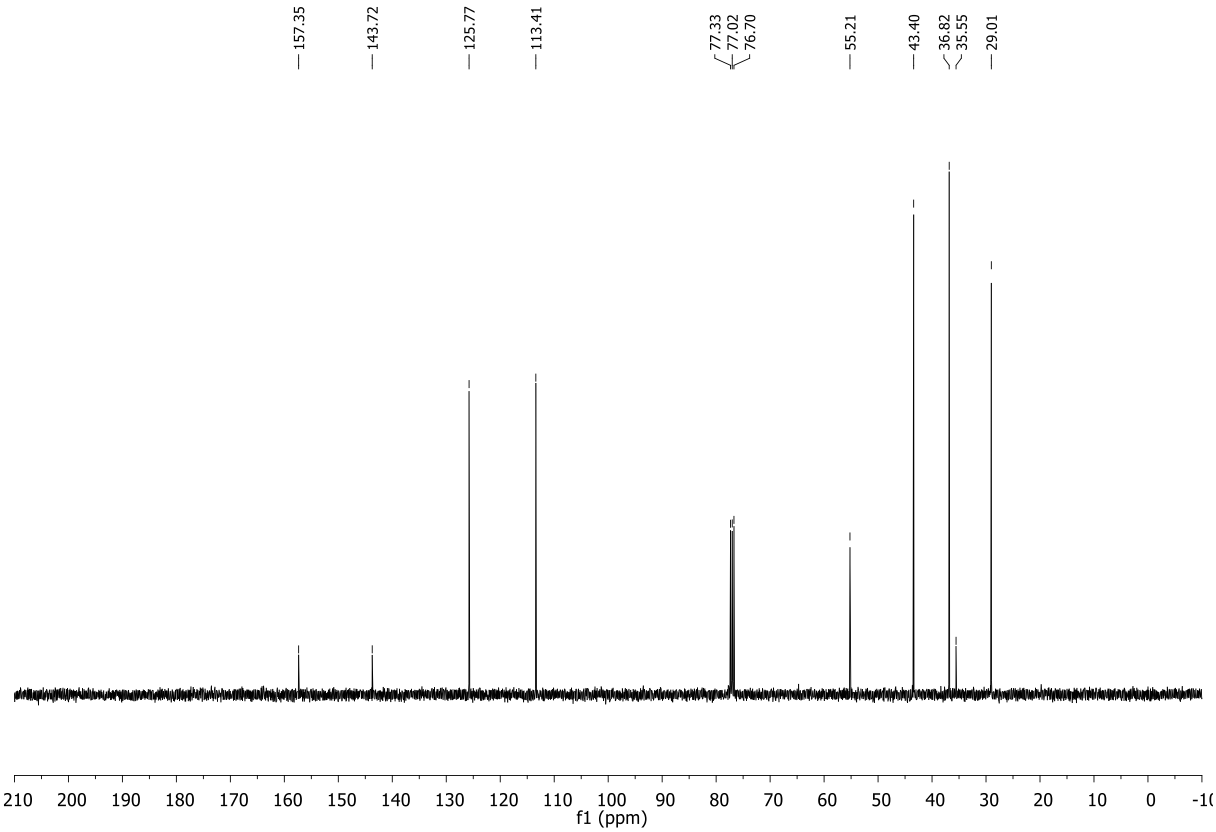

4-(Adamantan-1-yl)acetanilide (**54**)

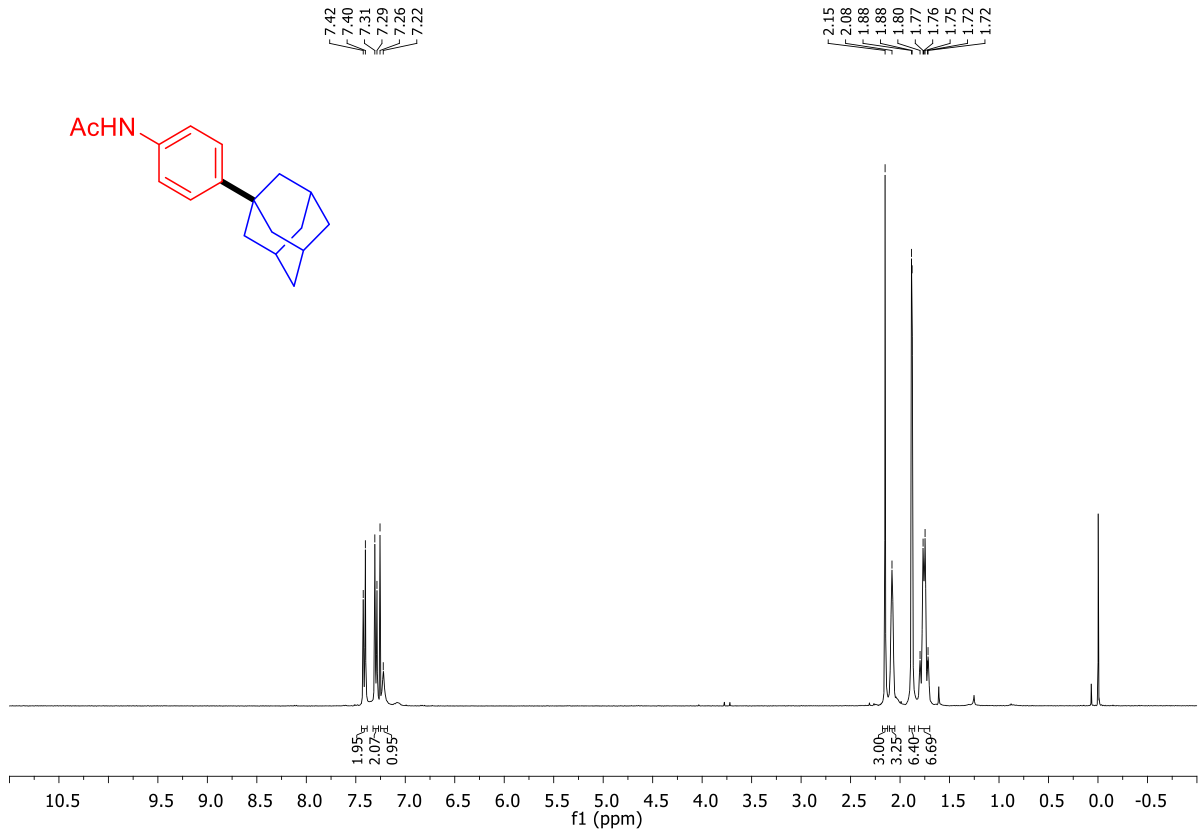

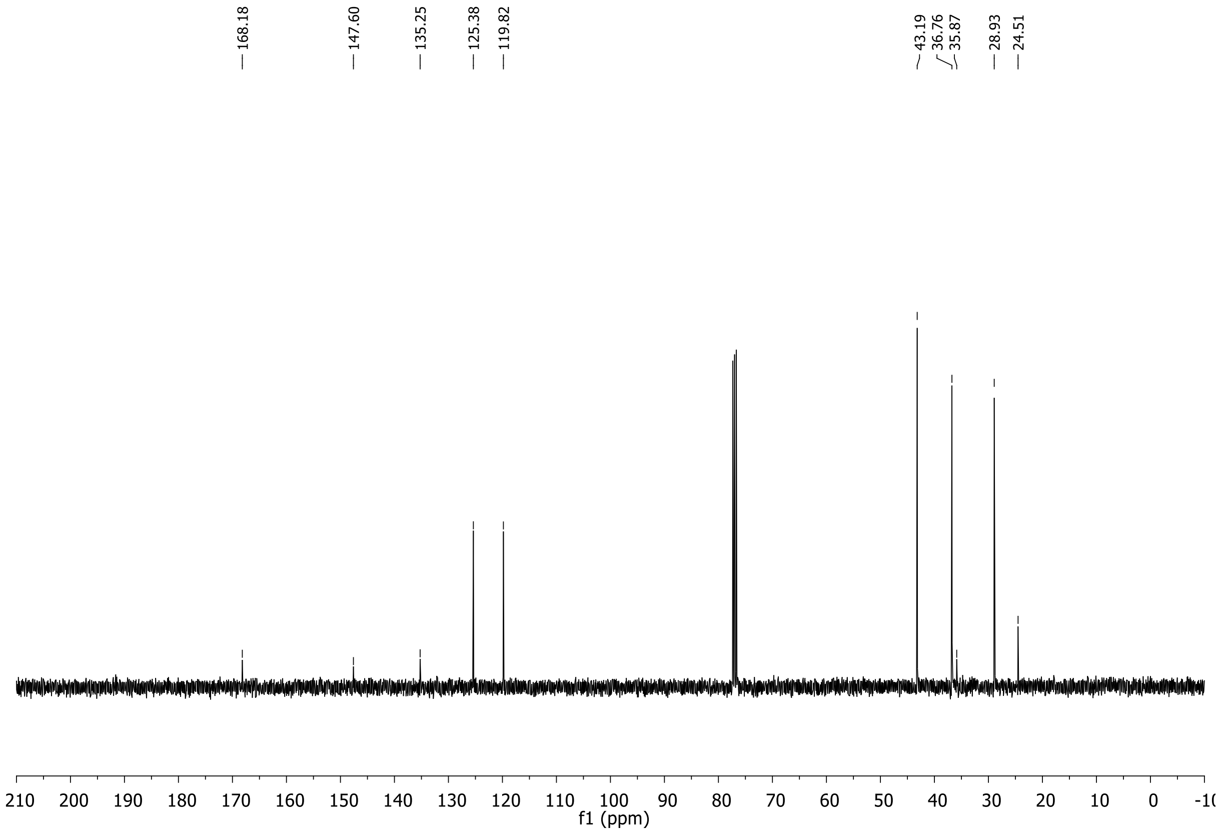

2-(Adamantan-1-yl)-4-bromoanisole (**55**)

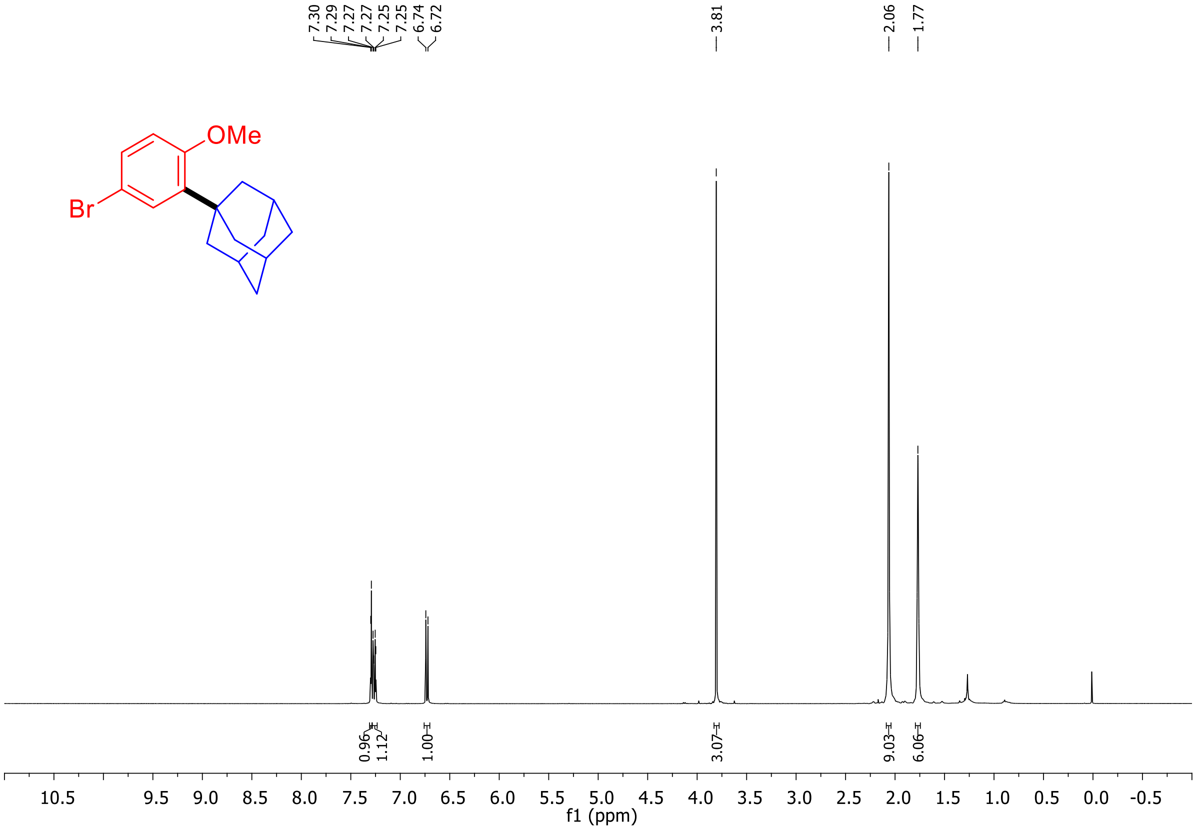

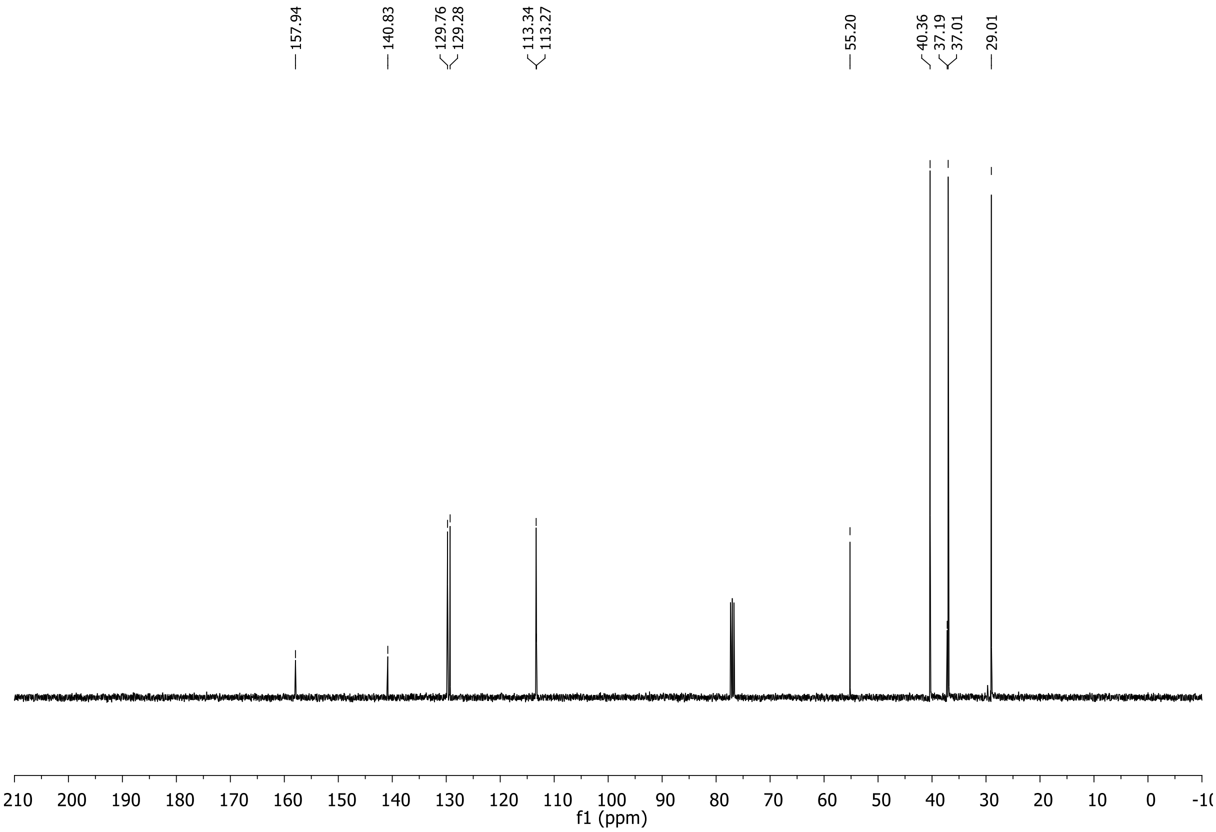

1-(4-Hydroxyphenyl)adamantane (**56**)

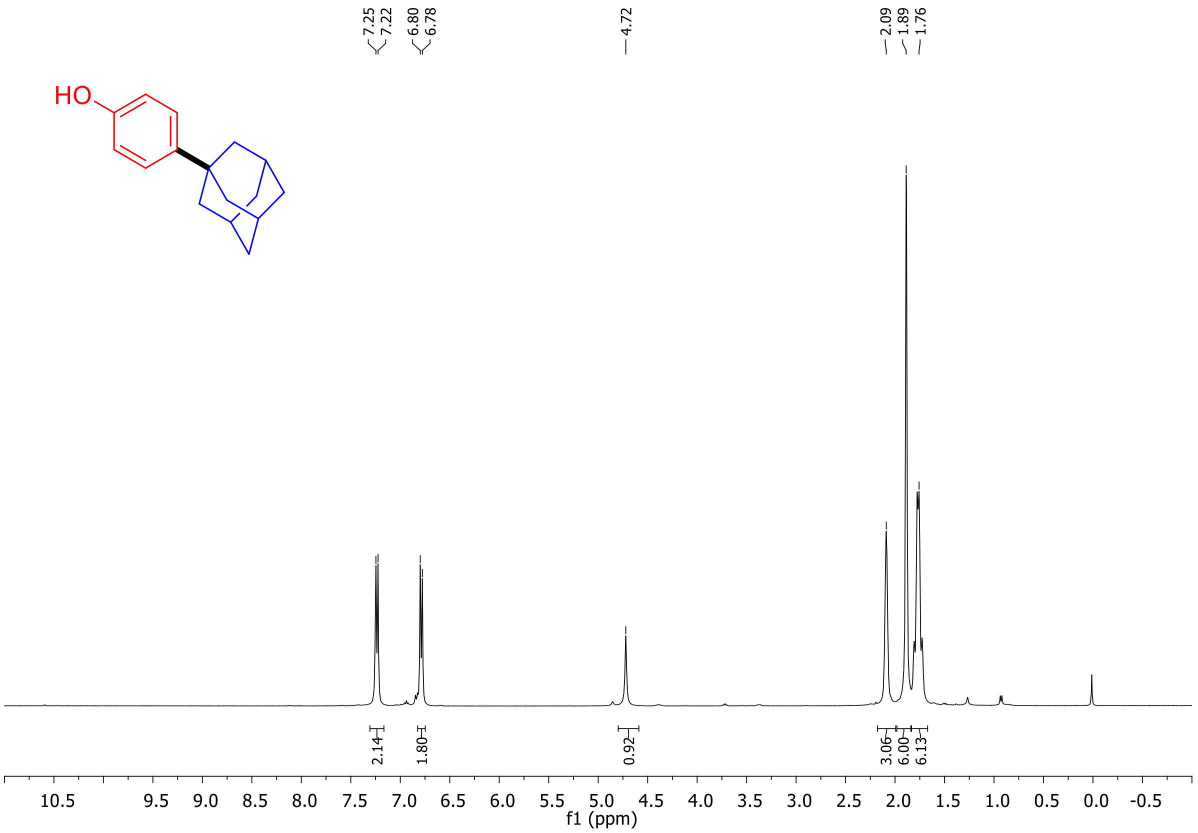

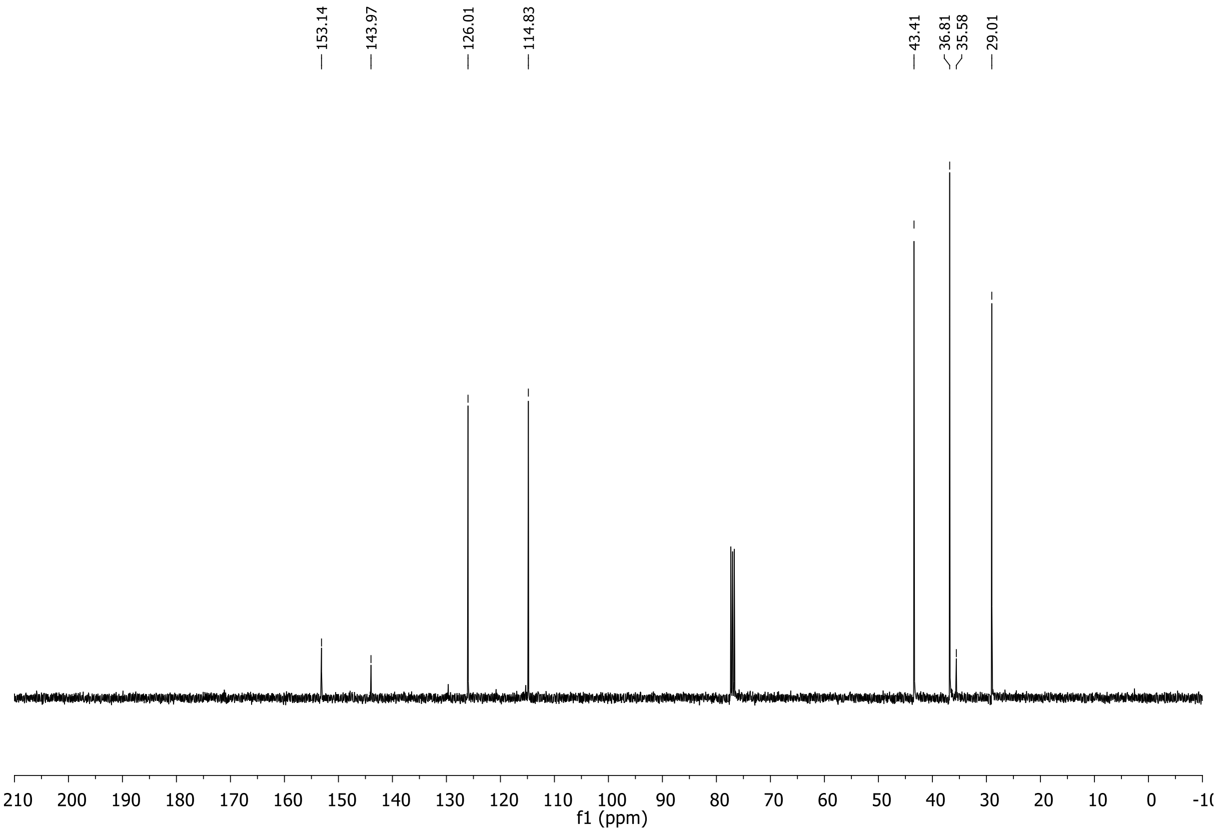

3-(Adamantan-1-yl)-2,6-dihydroxybenzaldehyde (**57**)

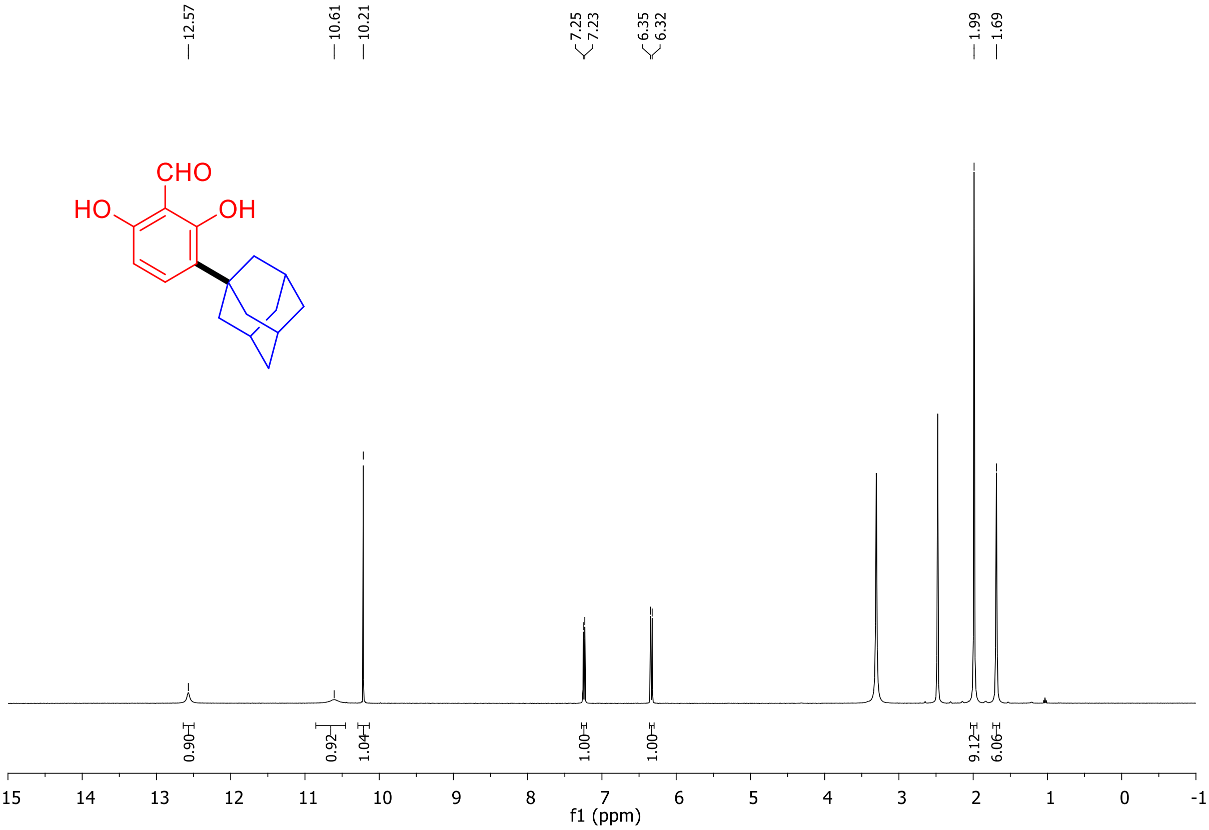

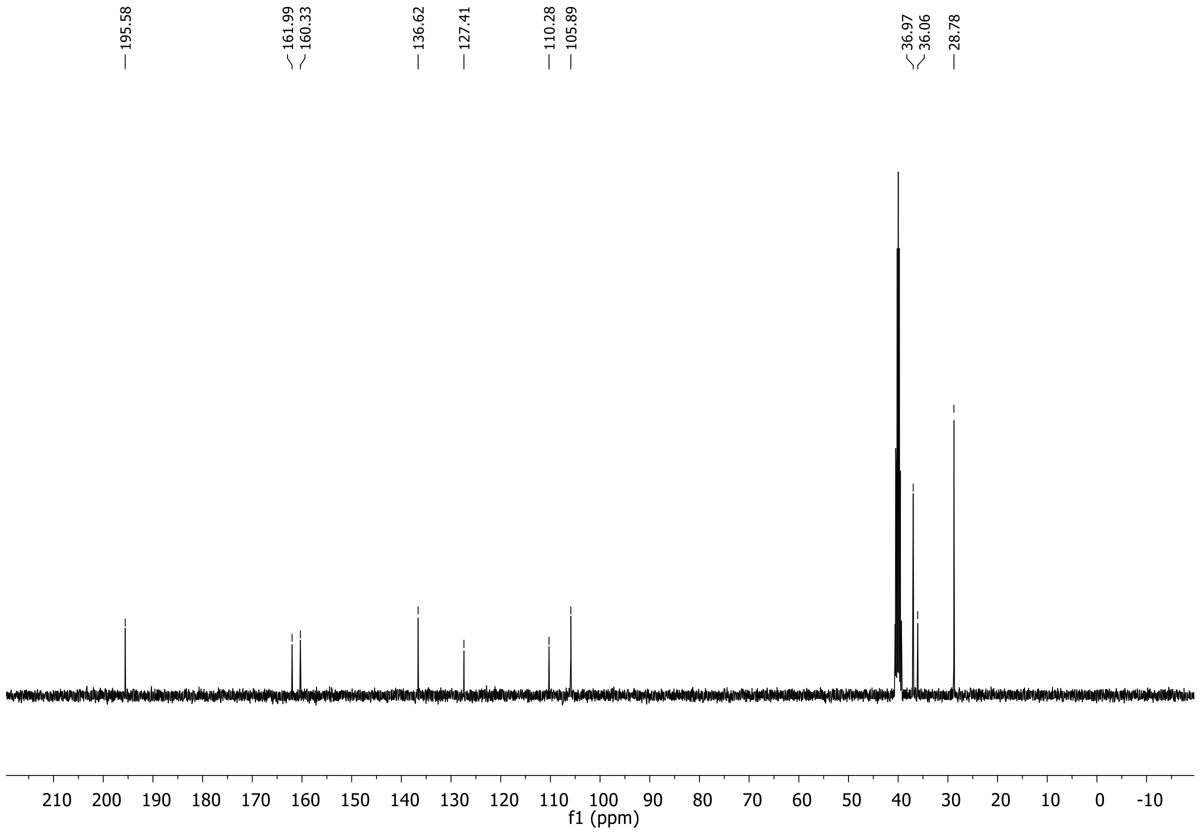

3-(Adamantan-1-yl)-2,6-dihydroxyacetophenone (**58**)

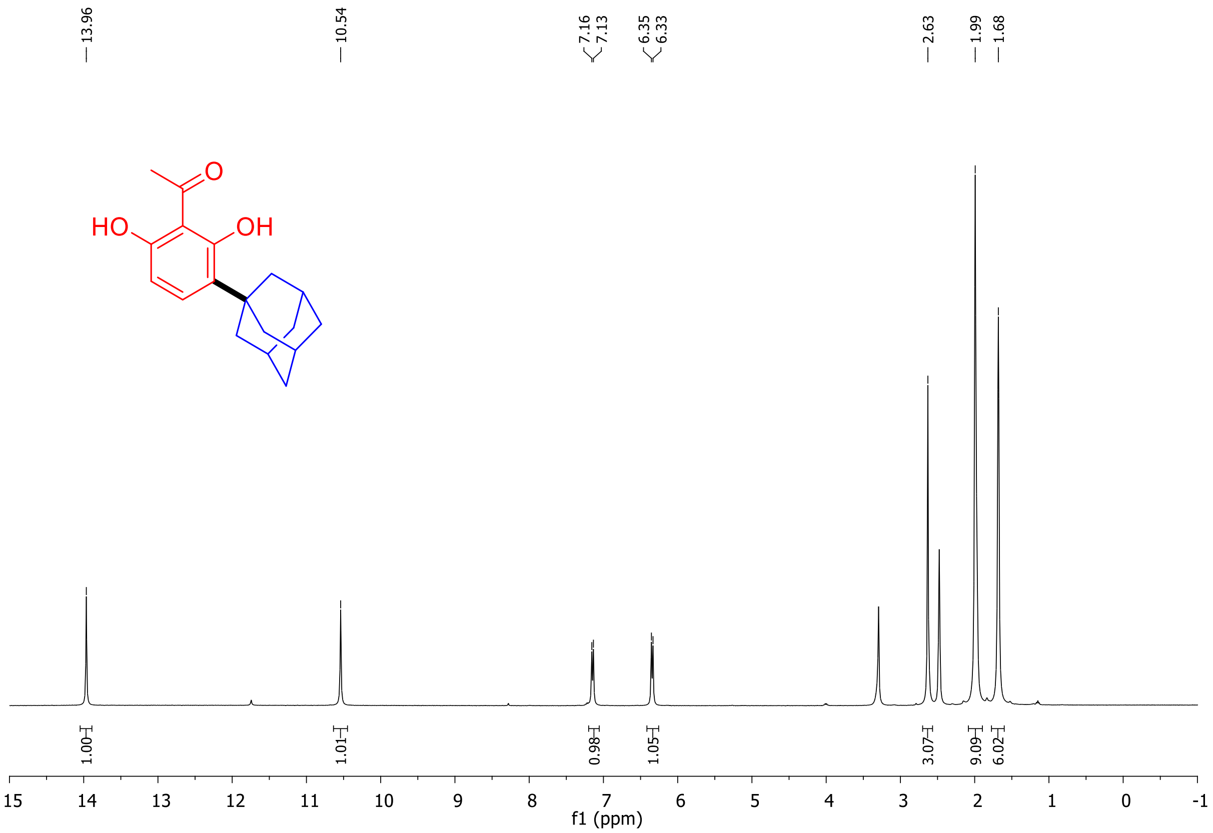

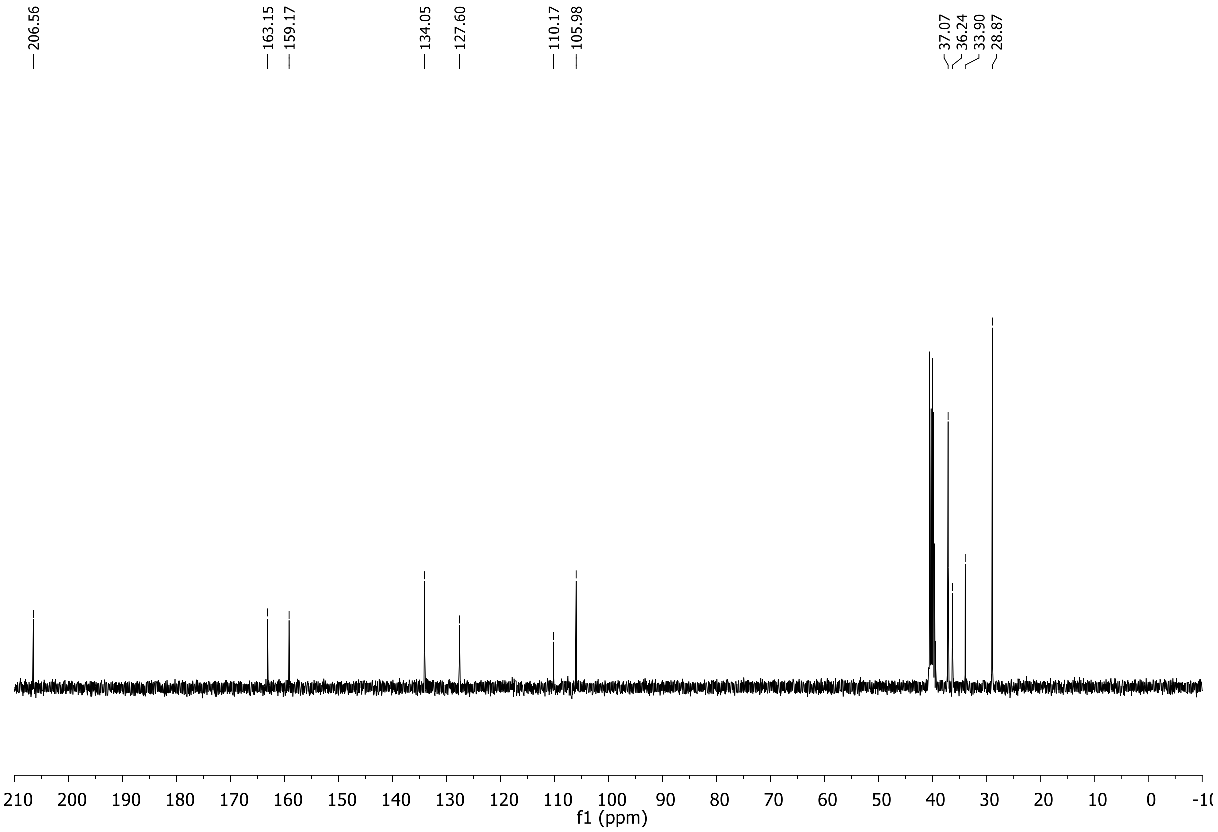

Methyl 3-(adamantan-1-yl)-2,6-dihydroxybenzoate (**59**)

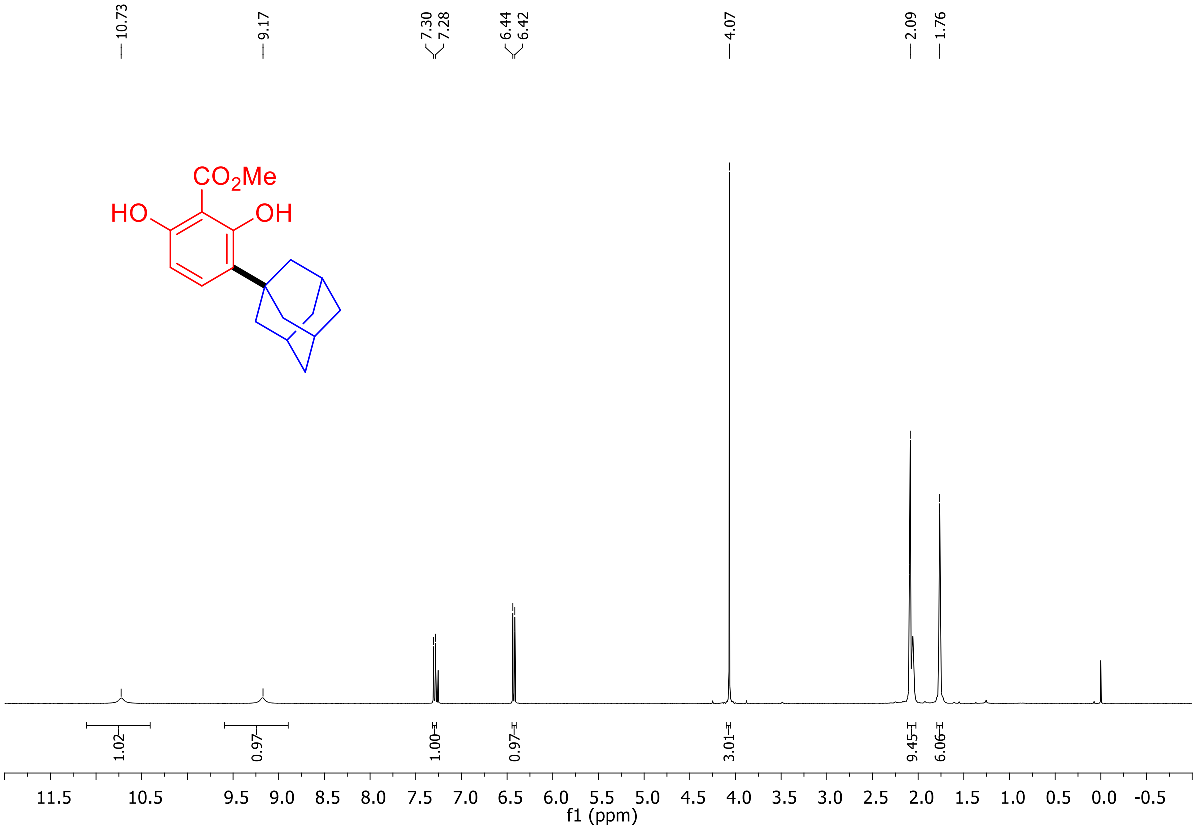

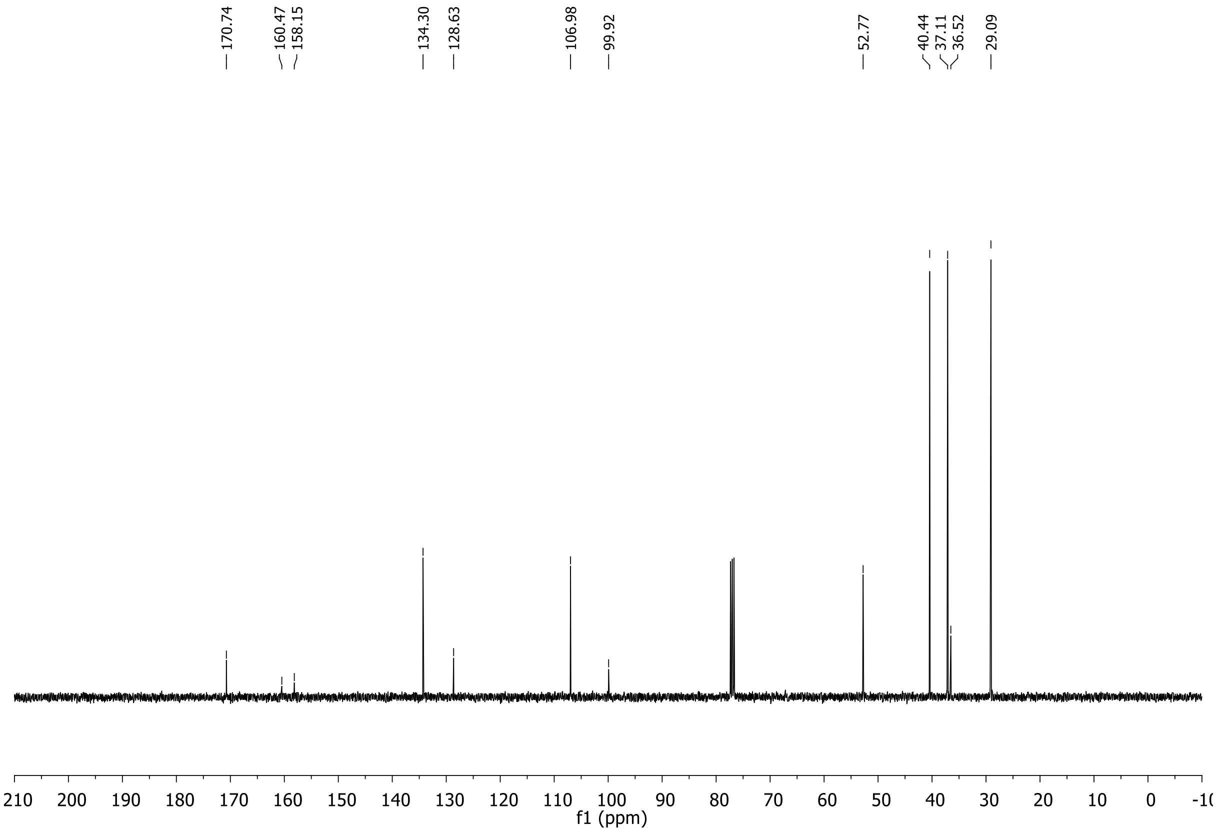

4-(Adamantan-1-yl)-2-nitroresorcinol (**60**)

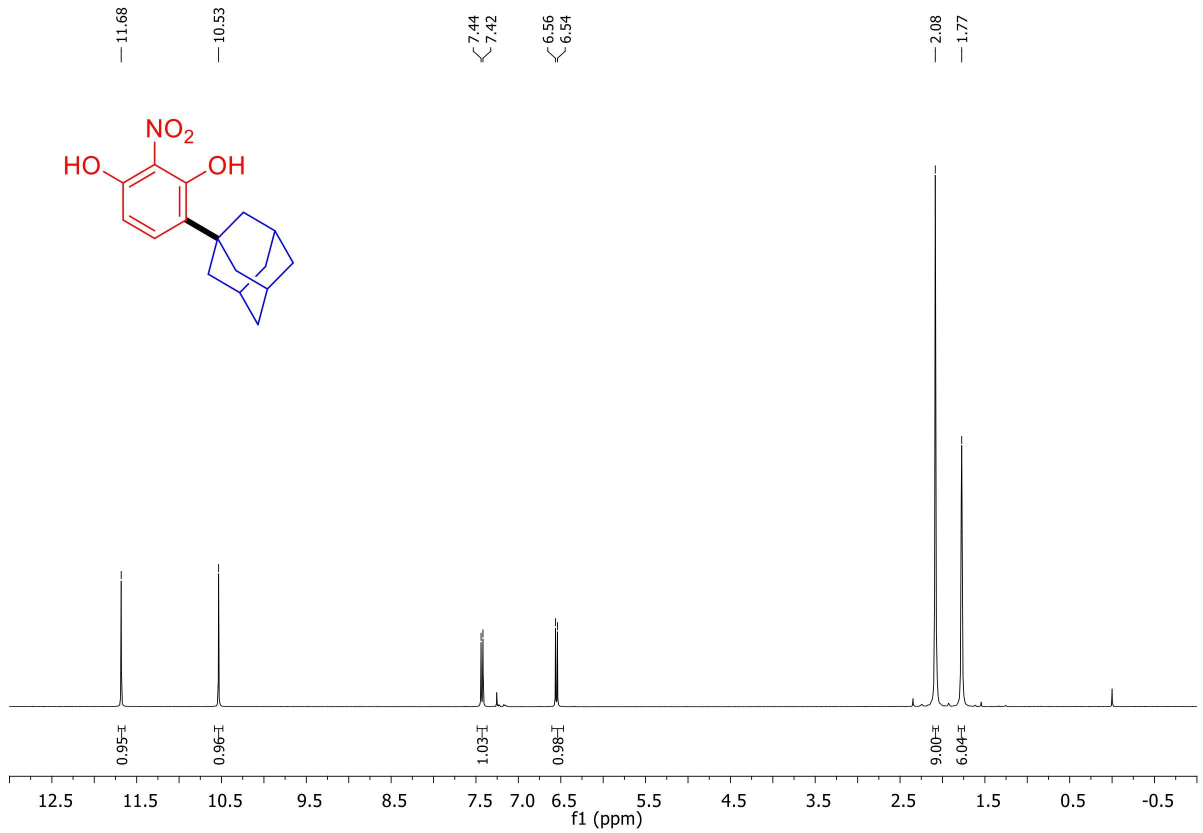

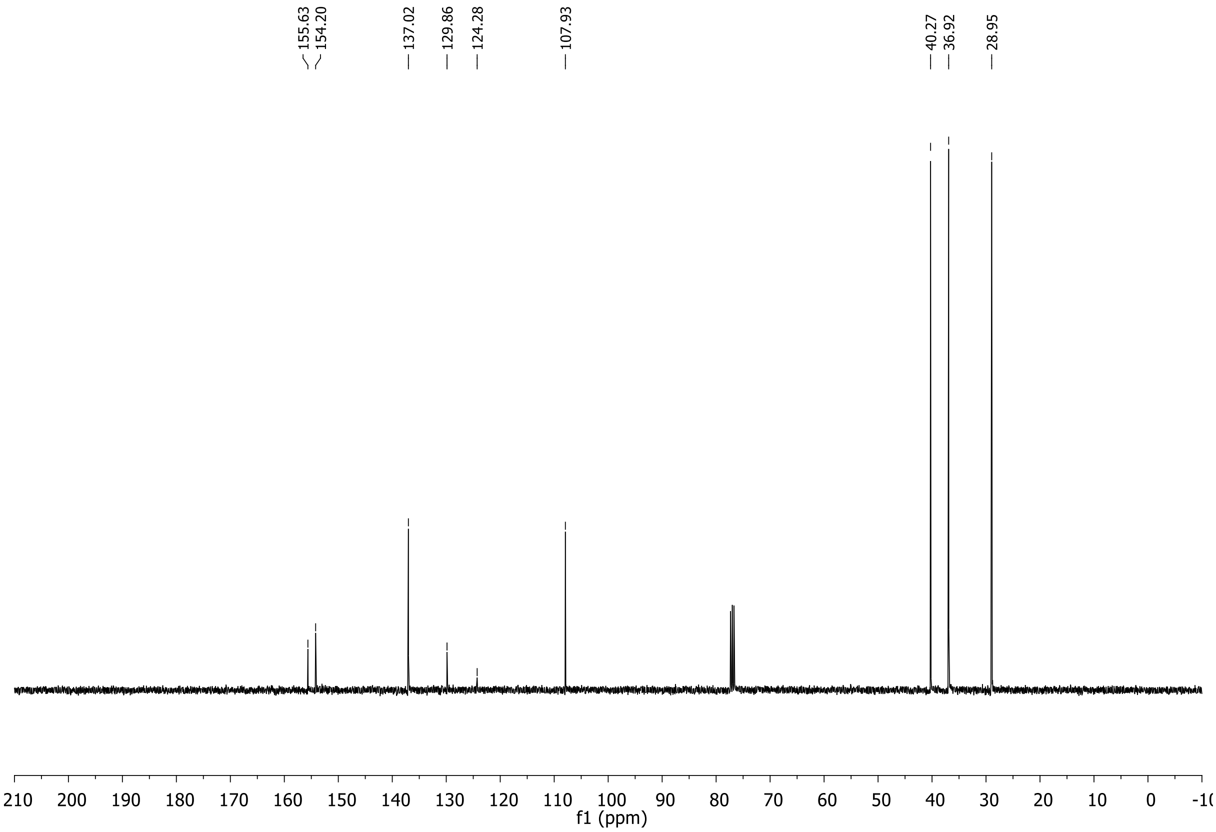

(8R,9S,13S,14S)-2-Benzhydryl-3-hydroxy-13-methyl-6,7,8,9,11,12,13,14,15,16-decahydro-17H-cyclopenta[a]phenanthren-17-one (**61**)

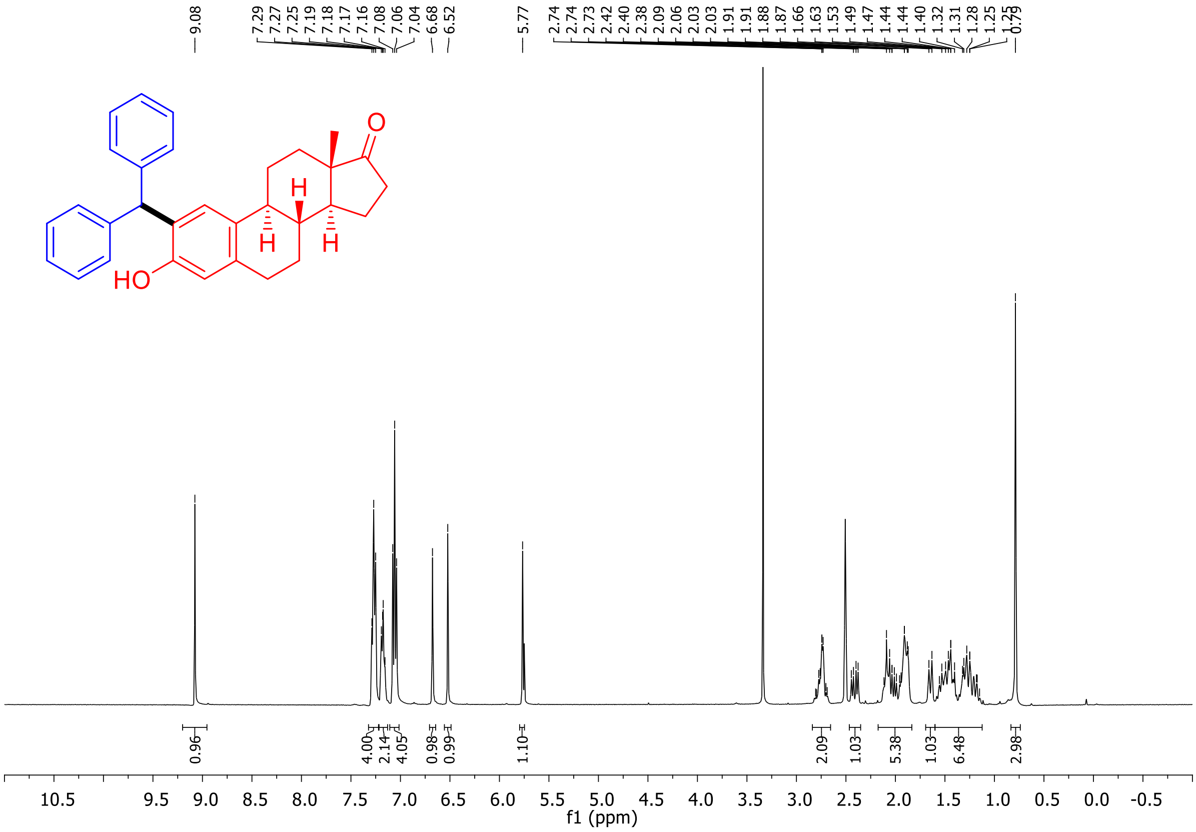

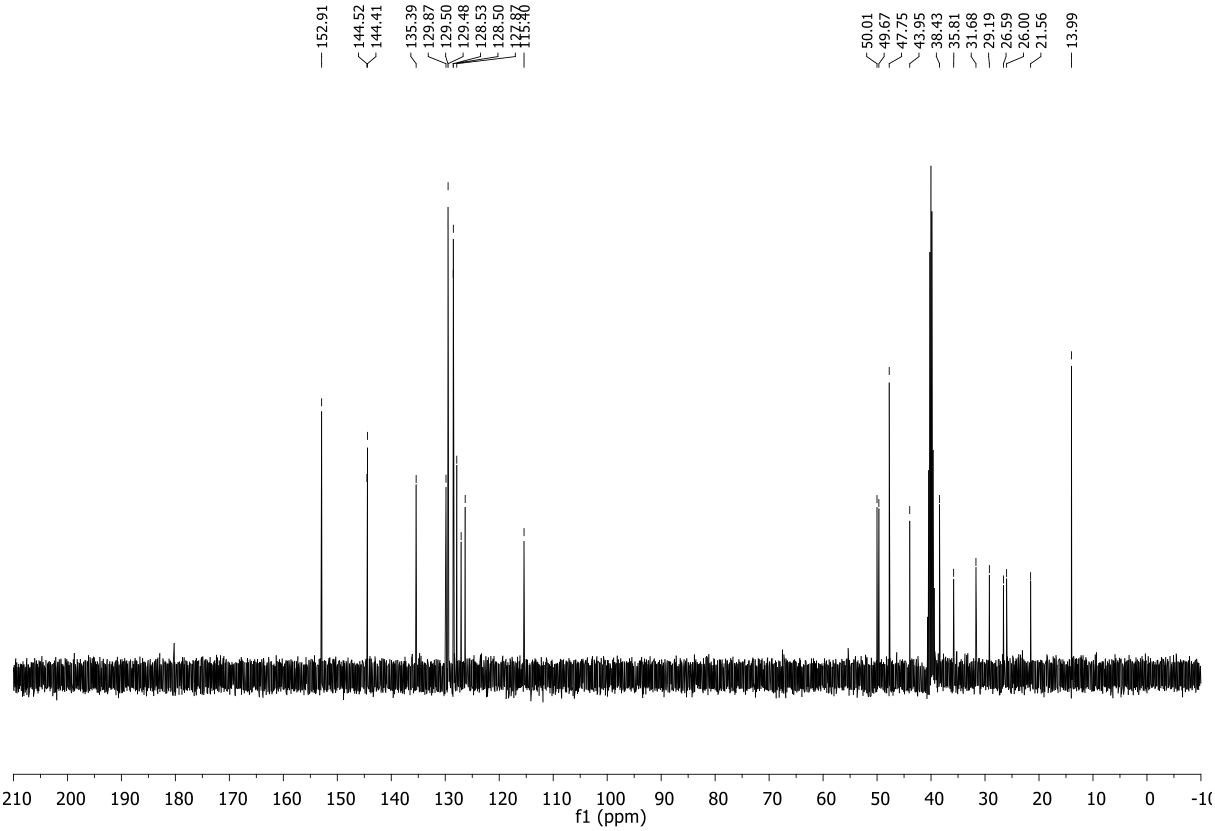

2-(4-Methoxybenzyl)-4-methylaniline (**62**)

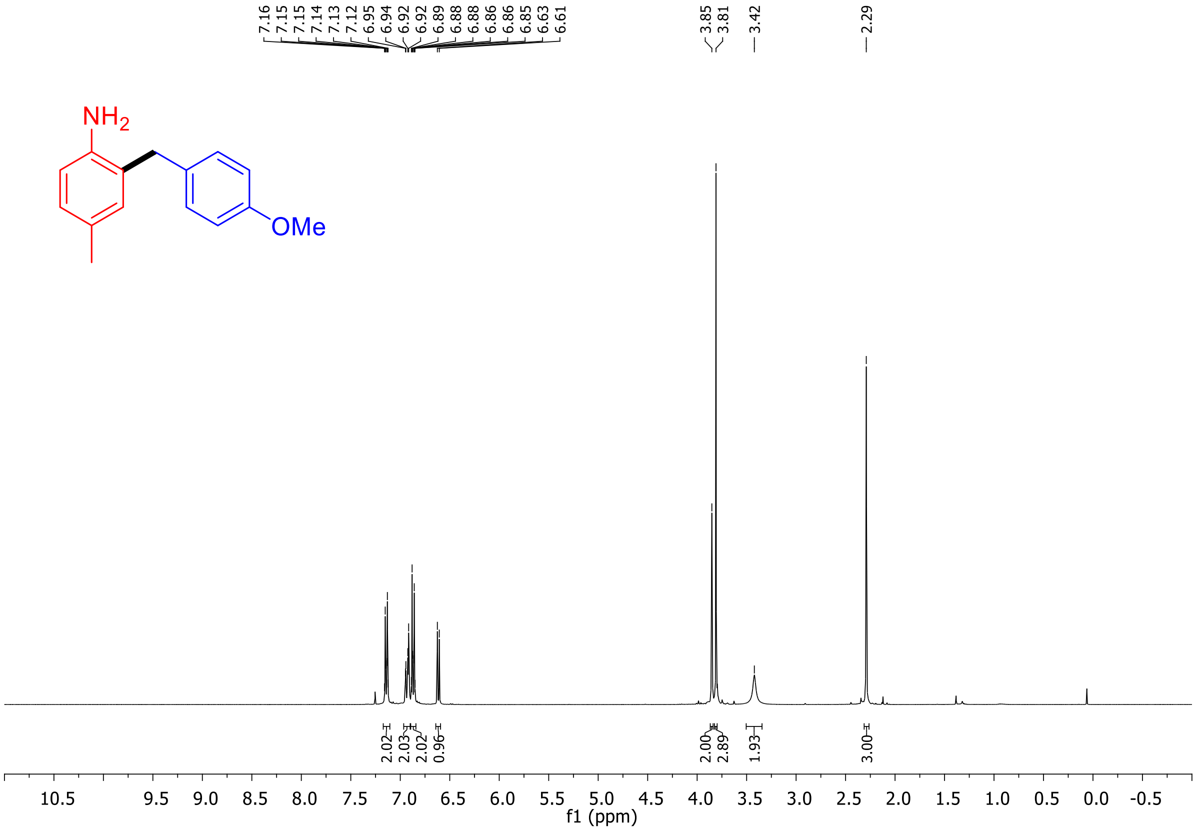

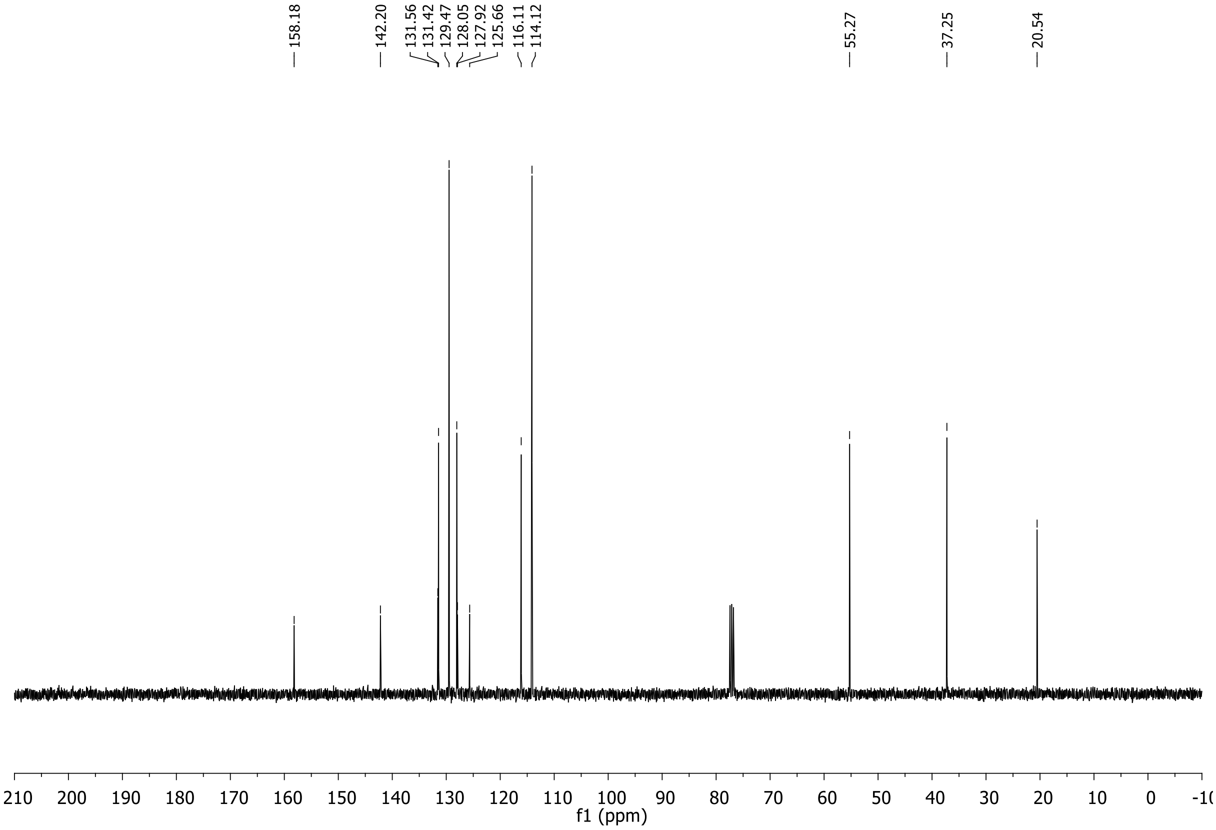

2-(4-Methoxybenzyl)-4,*N*,*N*-trimethylaniline (**63**)

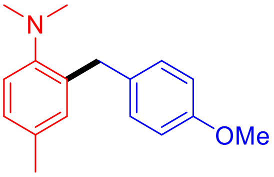

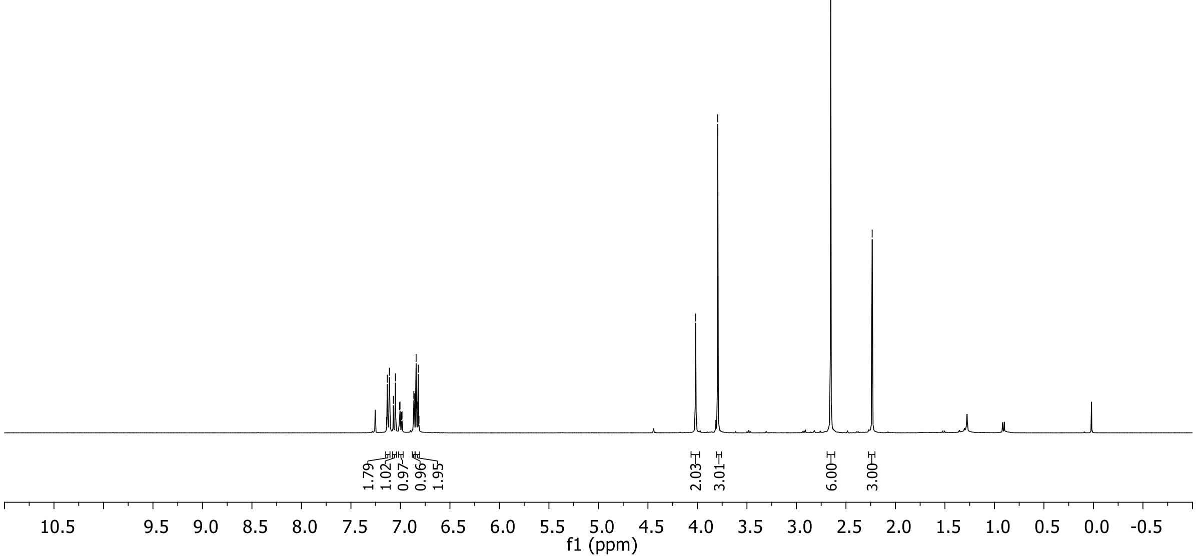

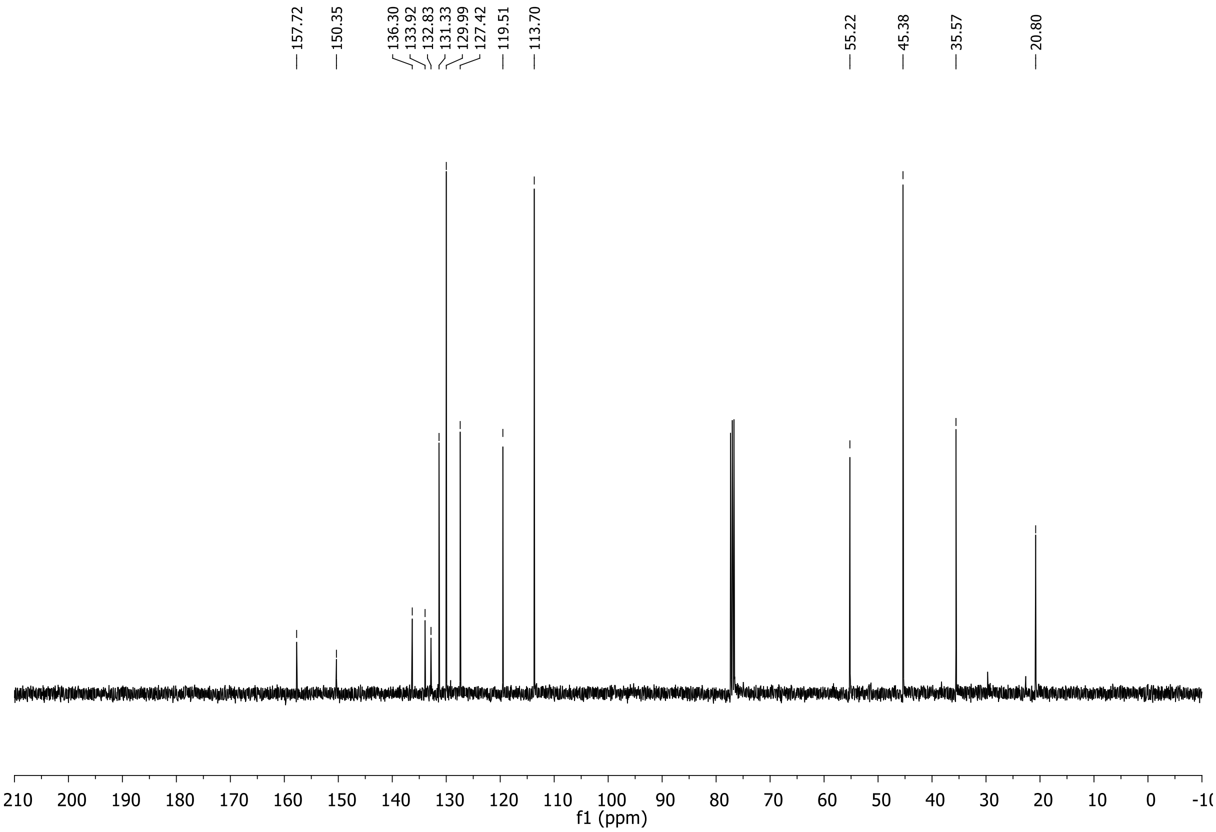

2-Benzhydryl-4-methoxyaniline (**64**)

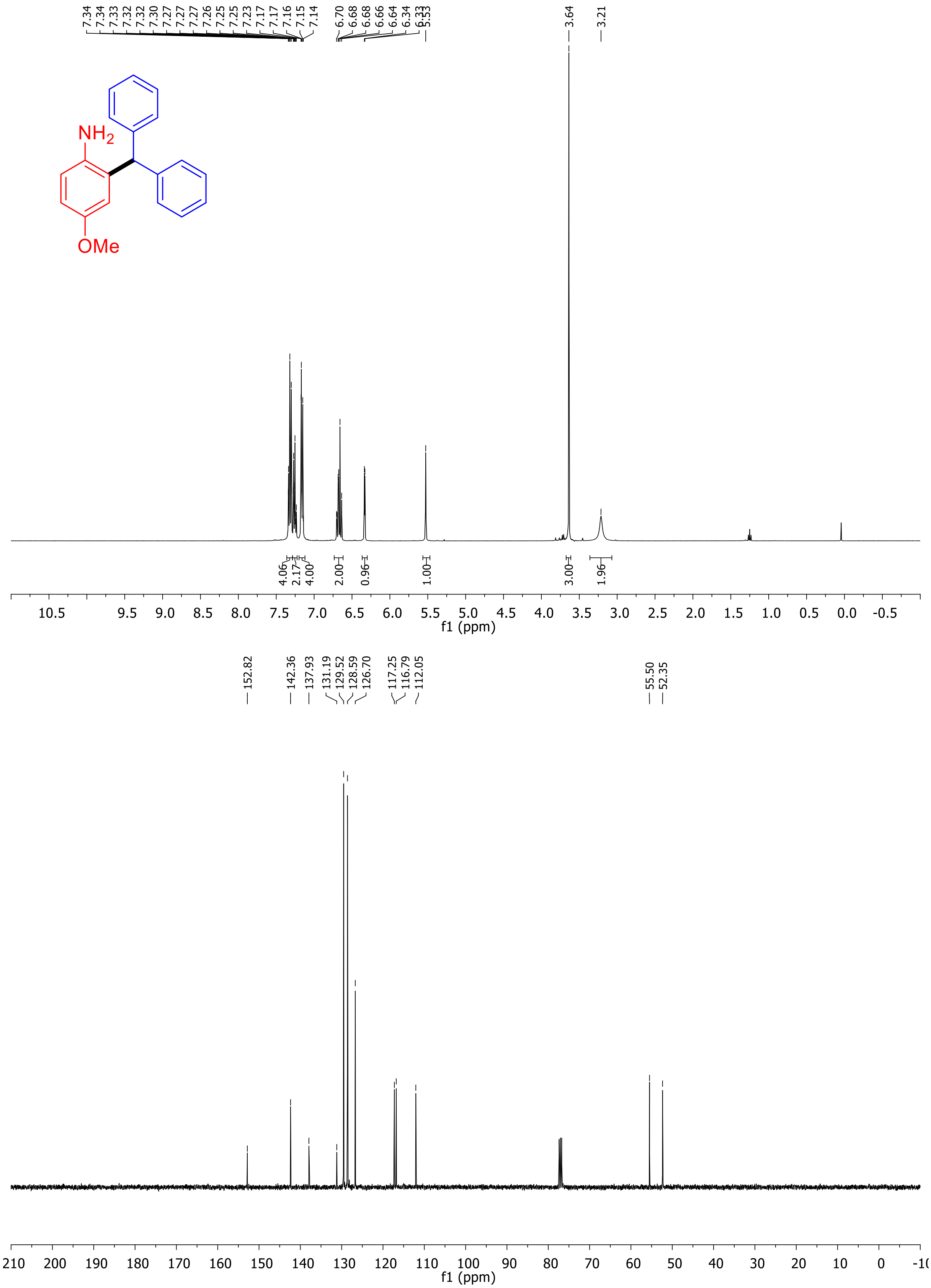

2-Benzhydryl-4-bromoaniline (**65**)

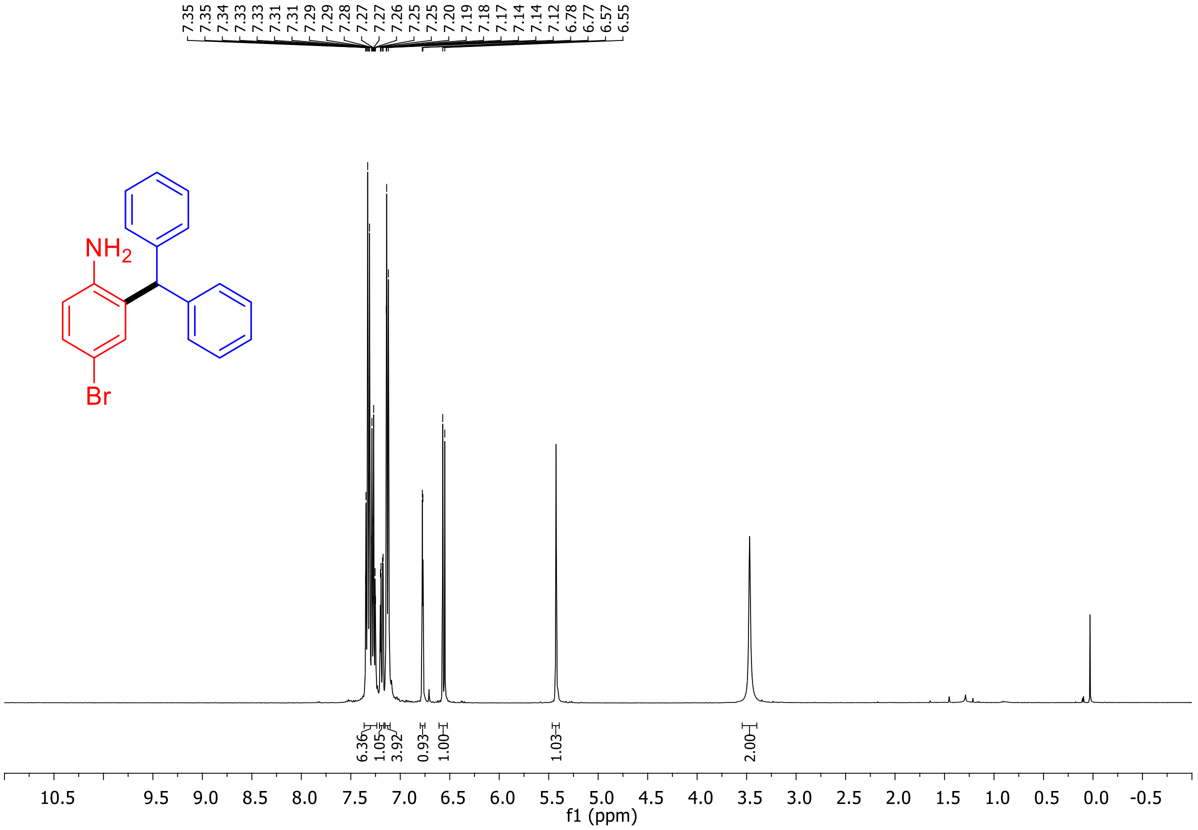

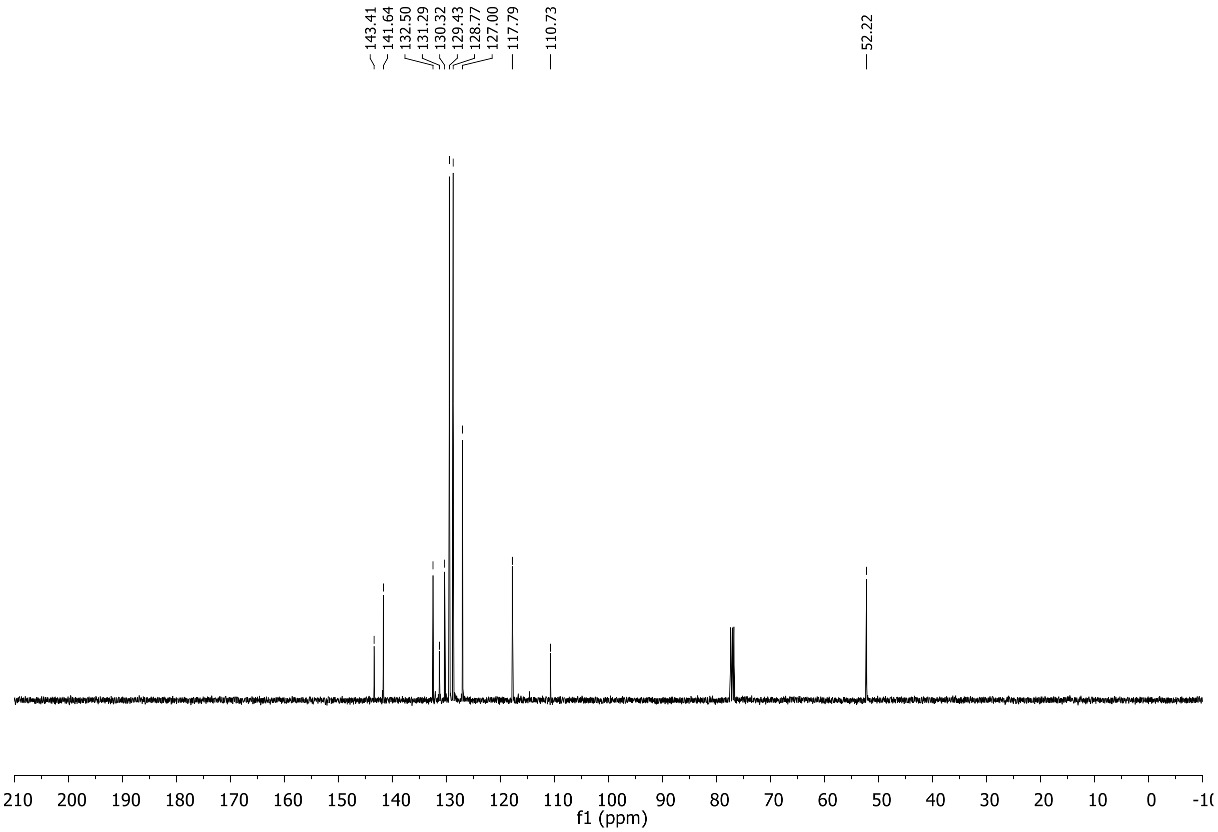

2-Benzhydryl-4-nitroaniline (**66**)

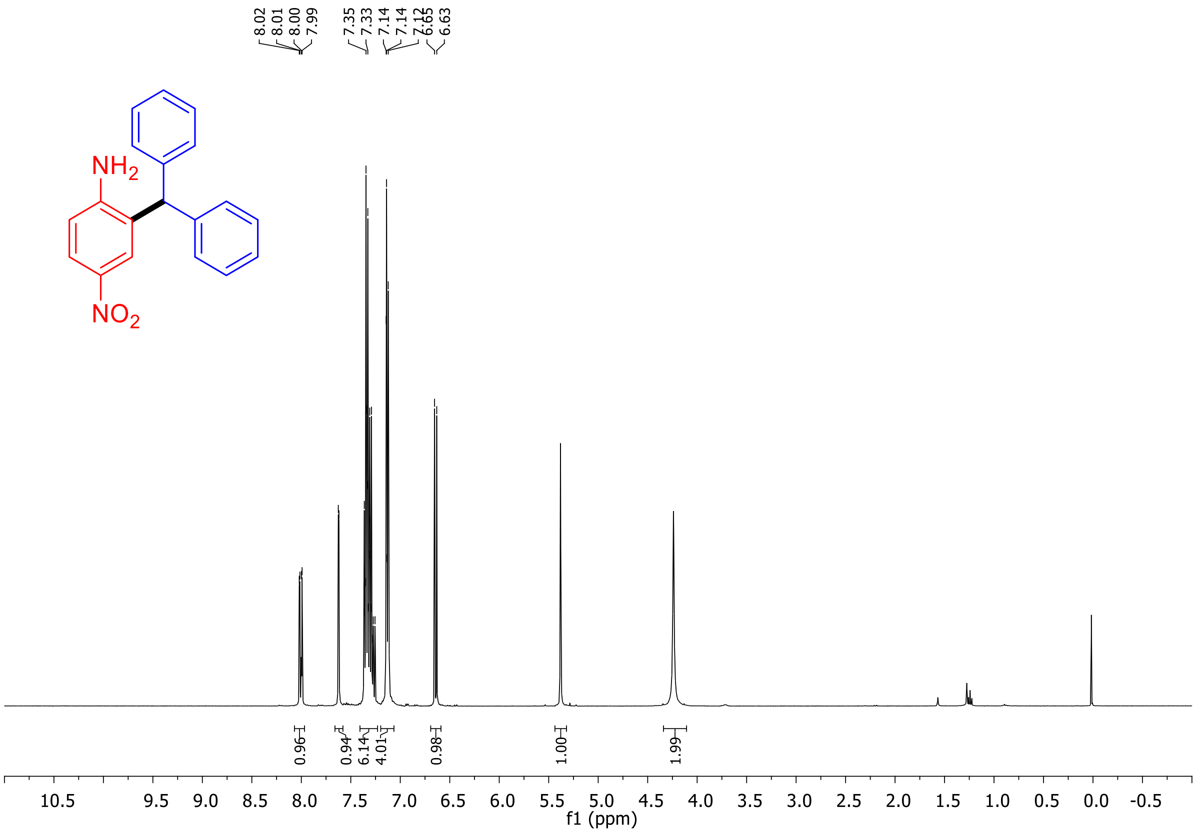

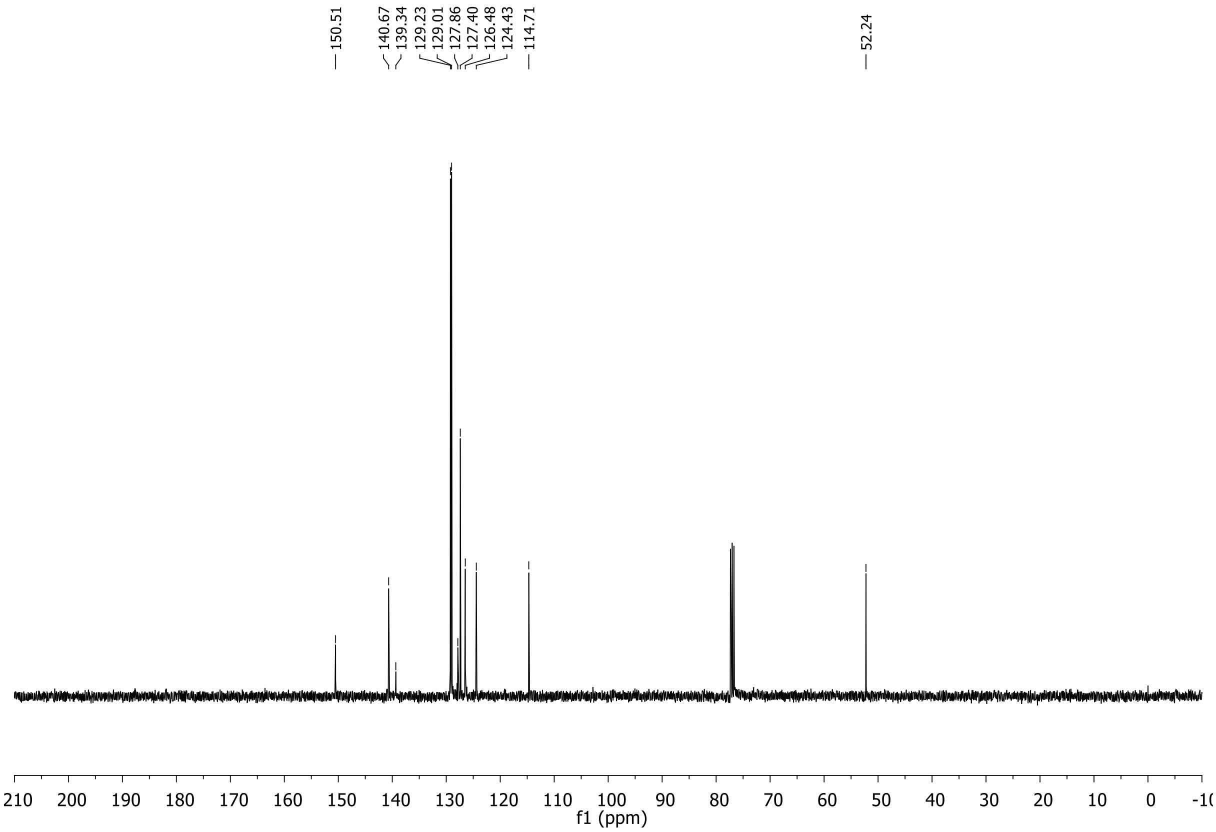

3-(4-Methoxybenzyl)indole (**67**)

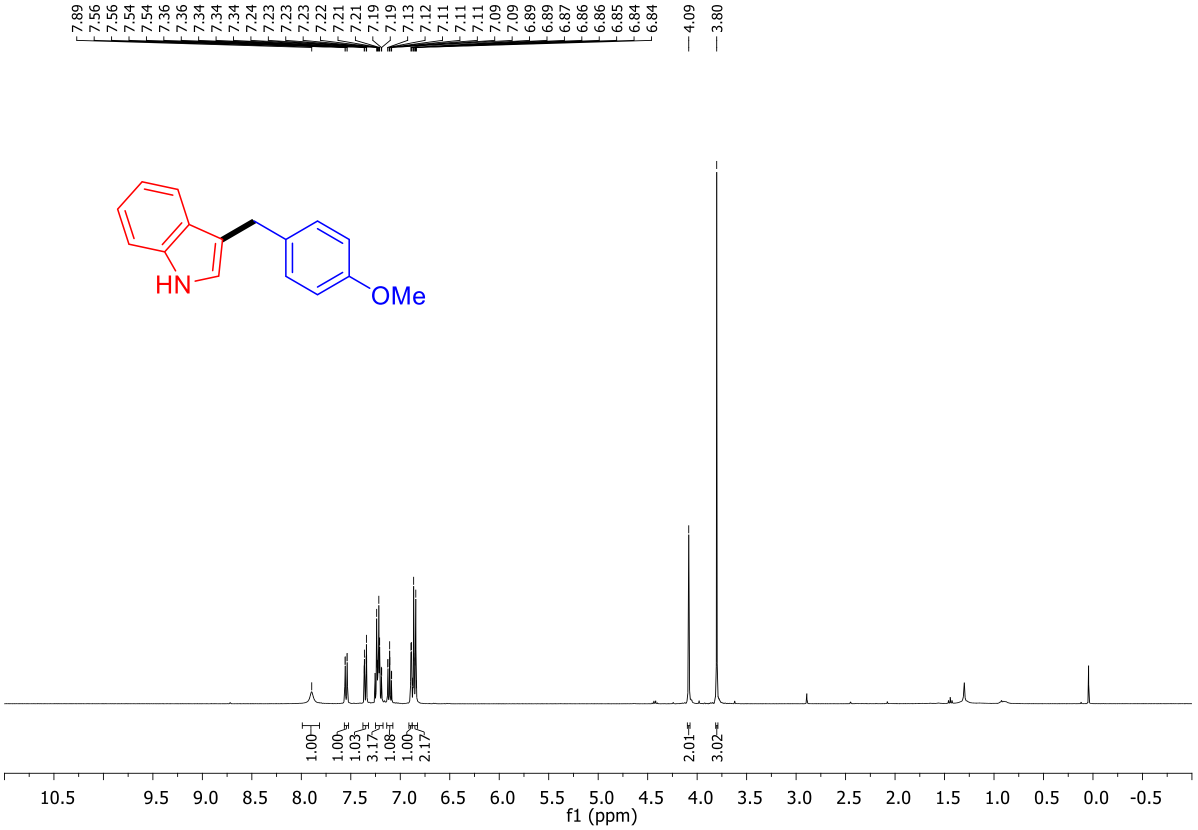

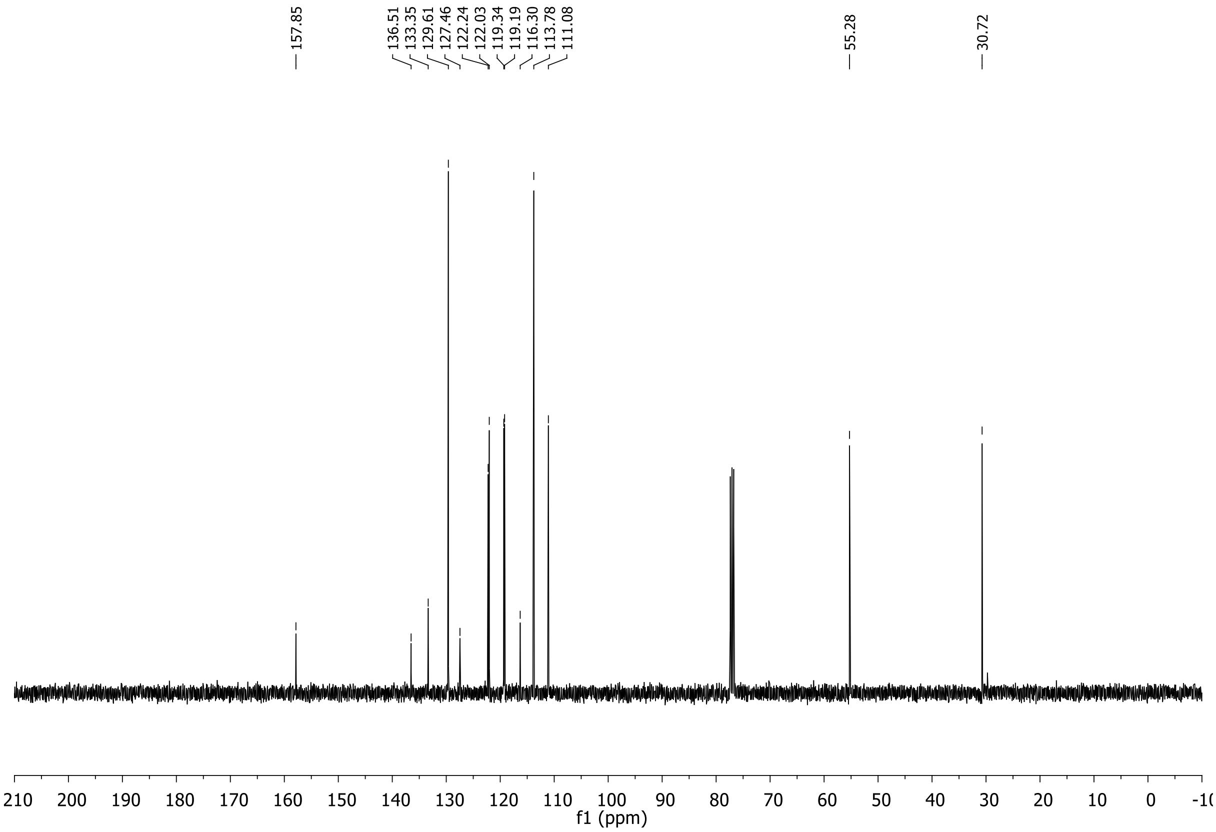

3-(4- Methoxybenzyl)-1-methylindole (**68**)

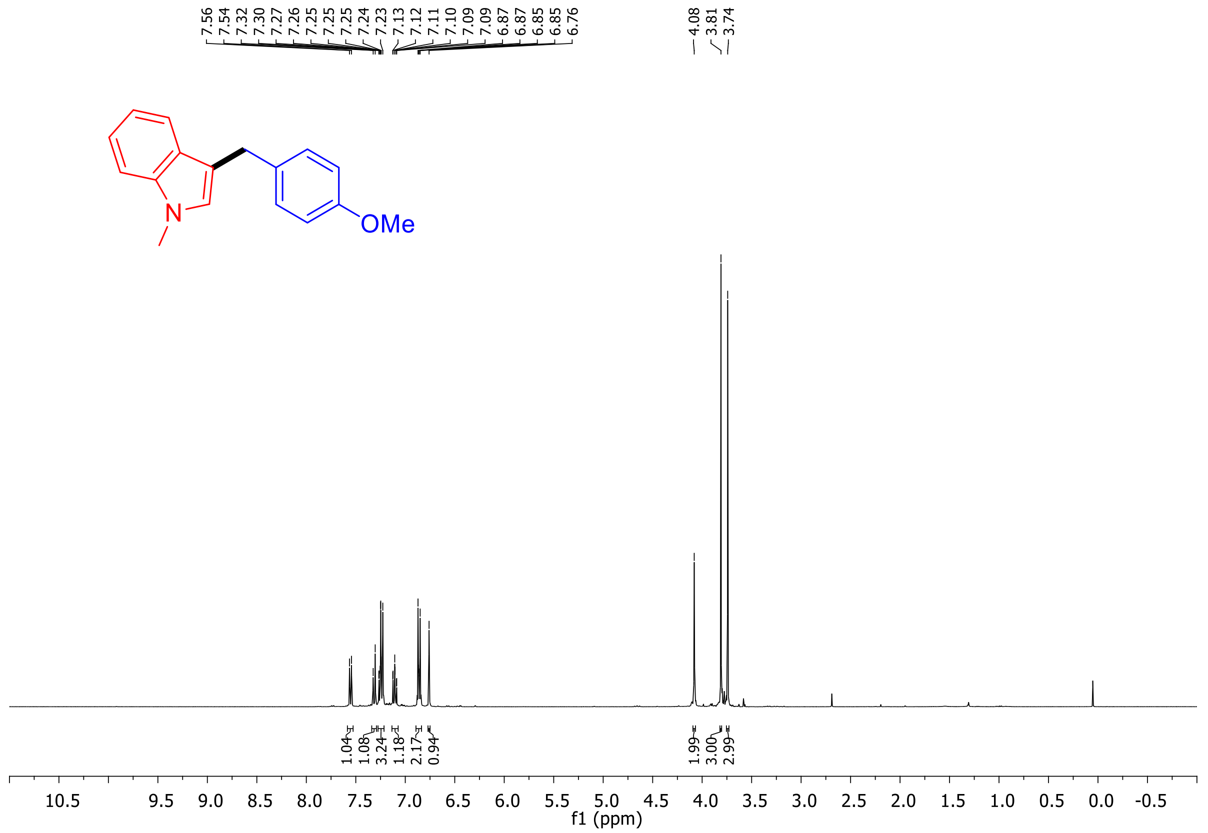

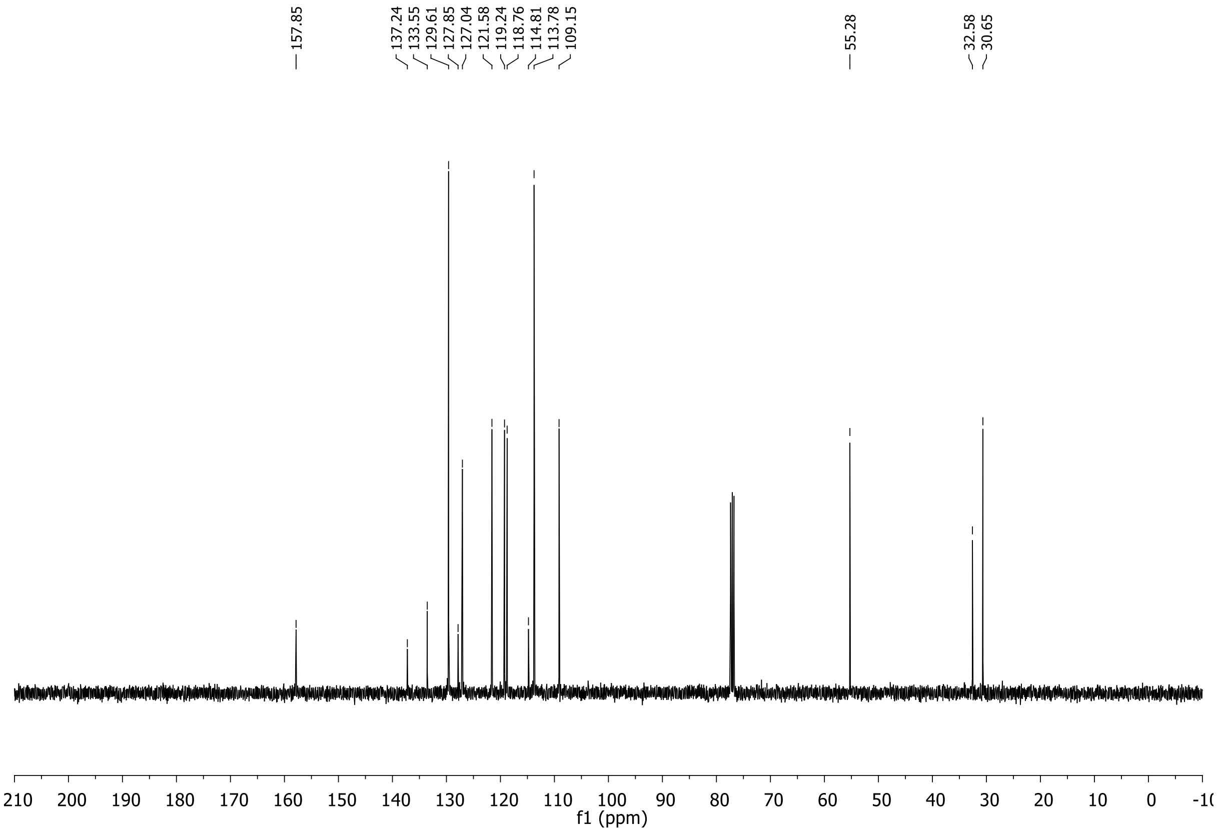

3-(4- Methoxybenzyl)-1-tosylindole (**69**)

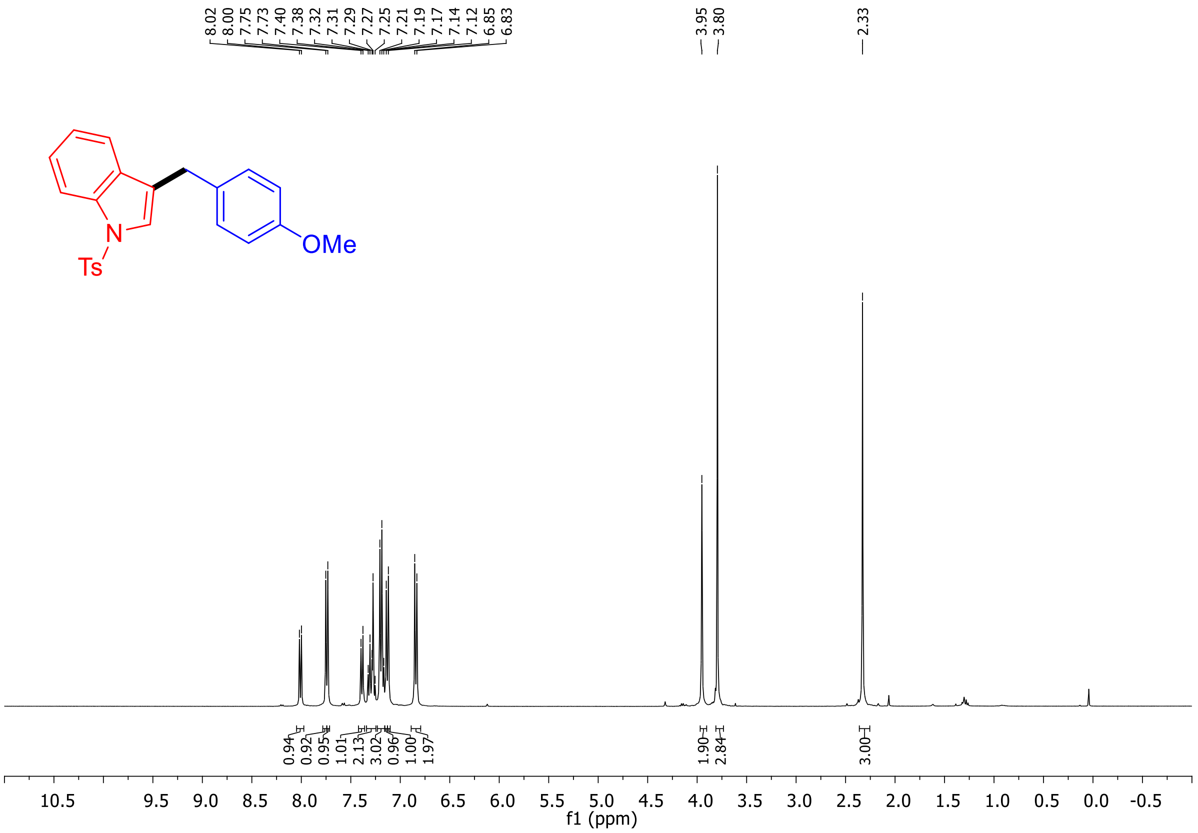

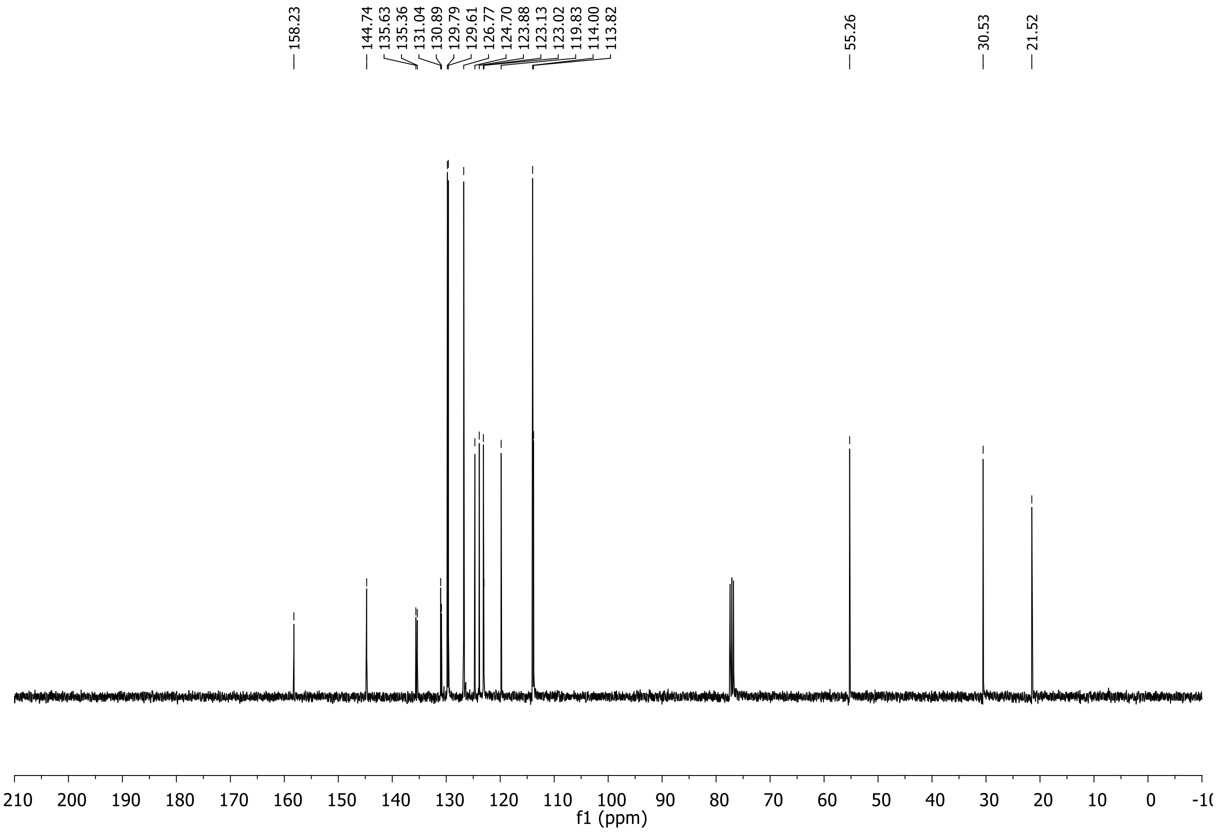

2,5-Dimethyl-3-(naphthalen-1-ylmethyl)thiophene (**70**)

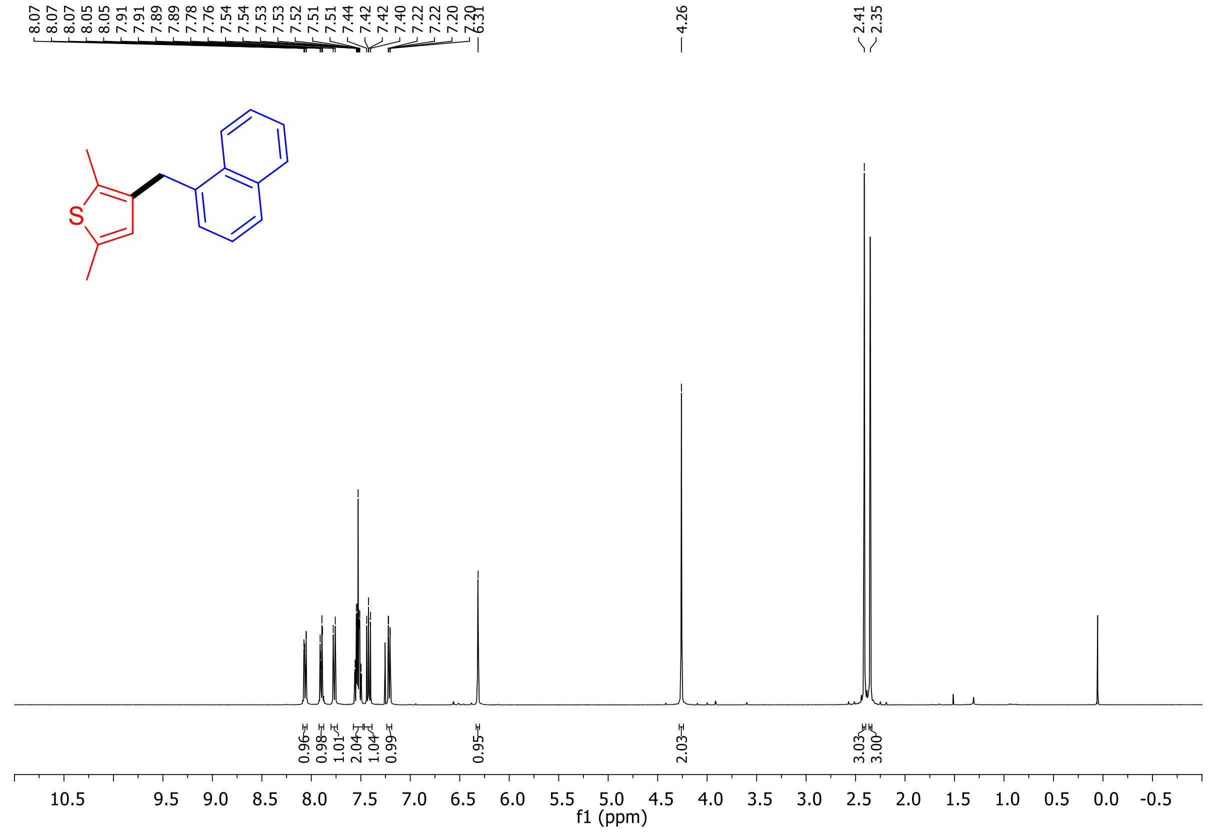

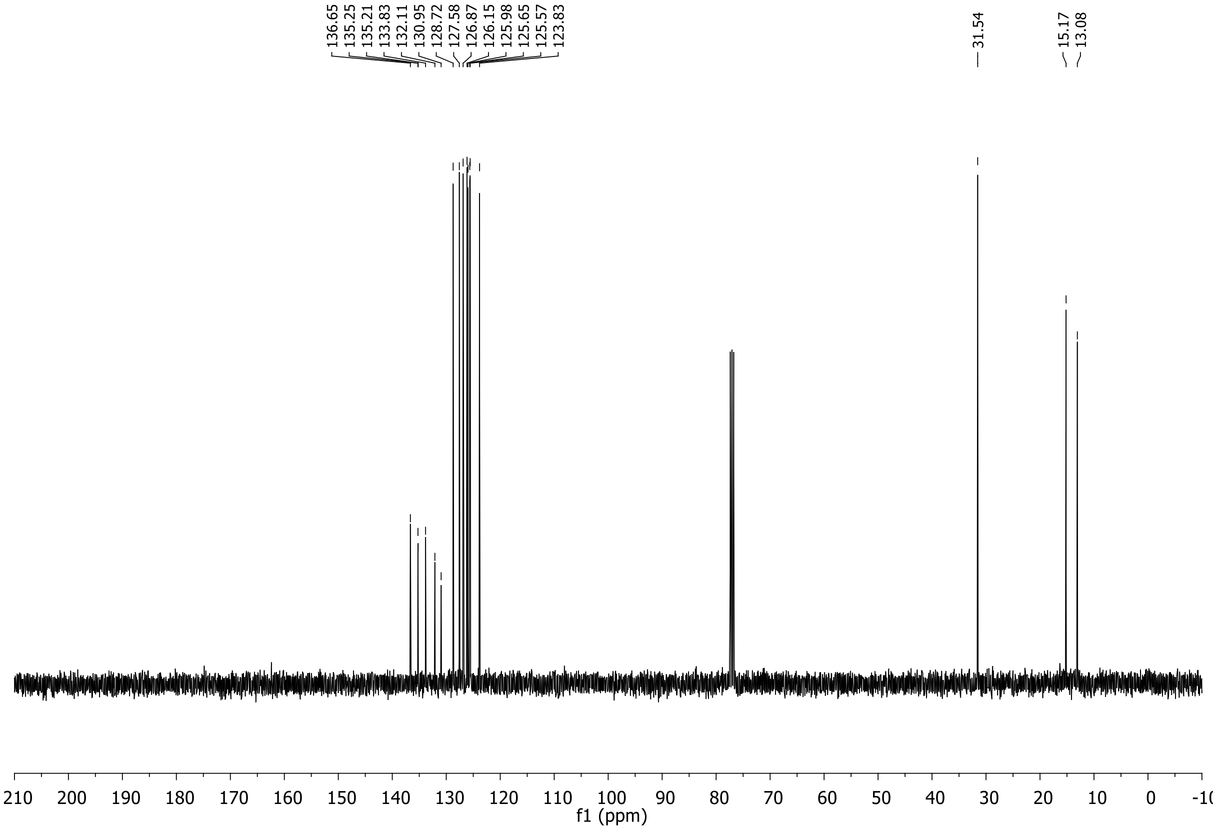

3-(Adamantan-1-yl)benzothiophene (**71**)

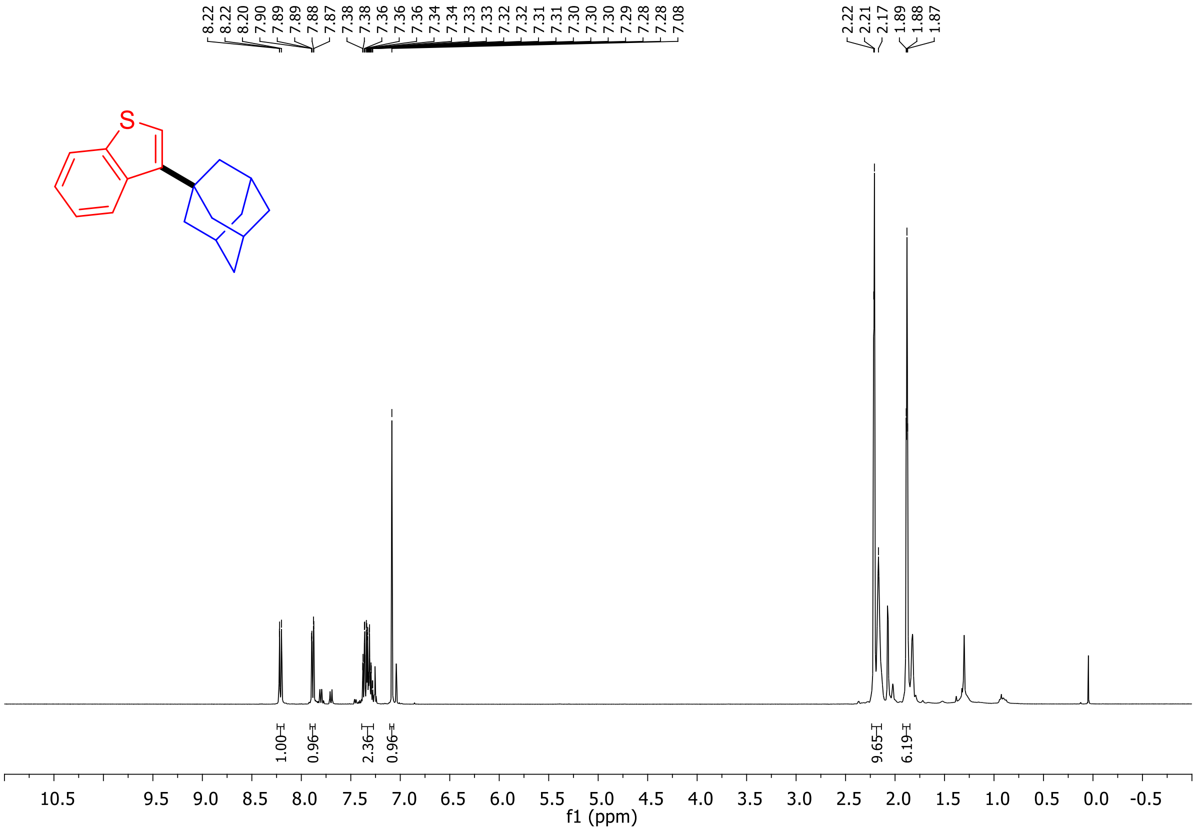

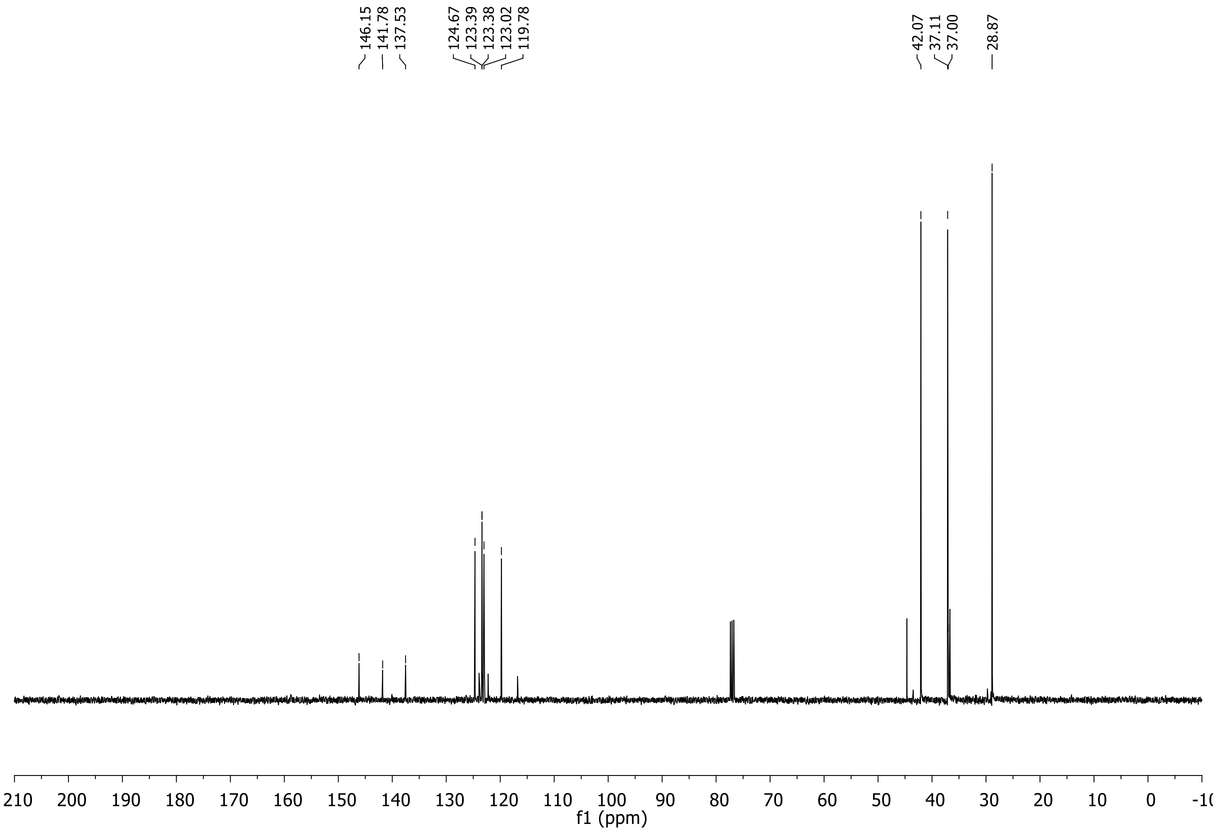

4-Bromobenzyl chloride (**74**)

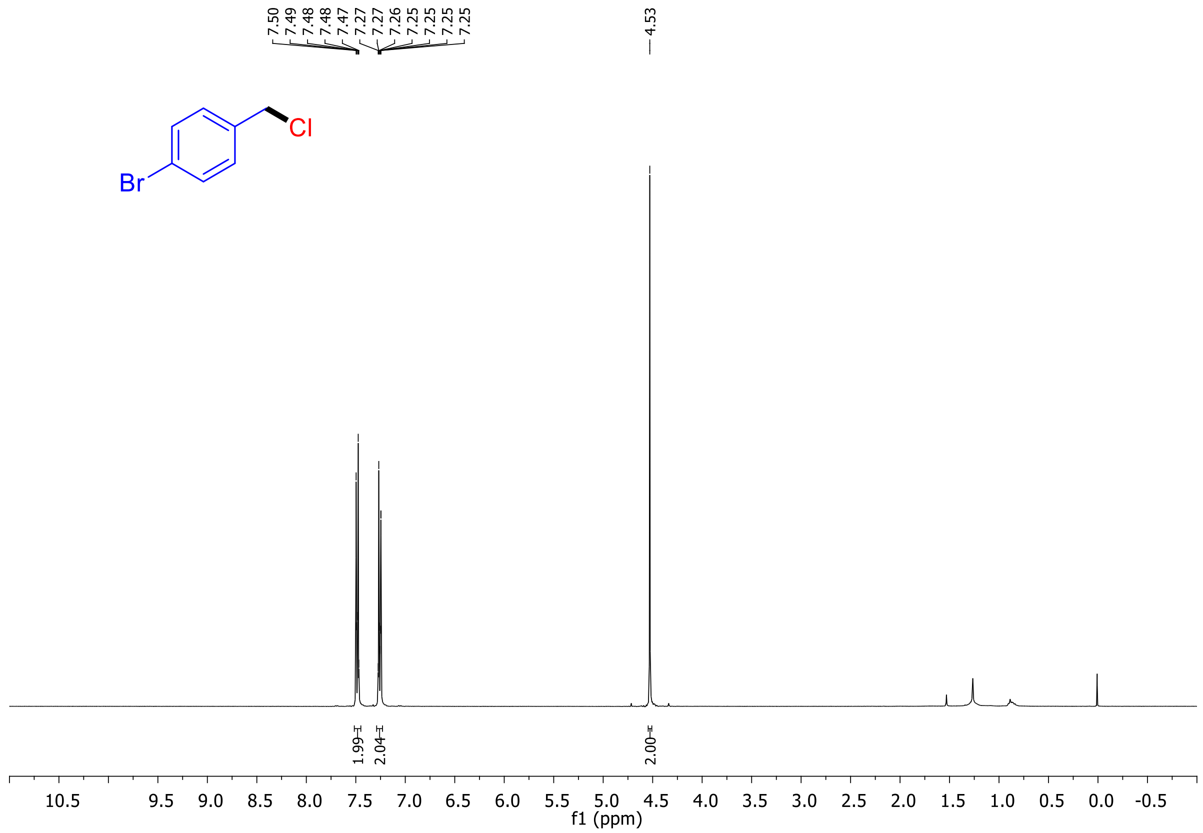

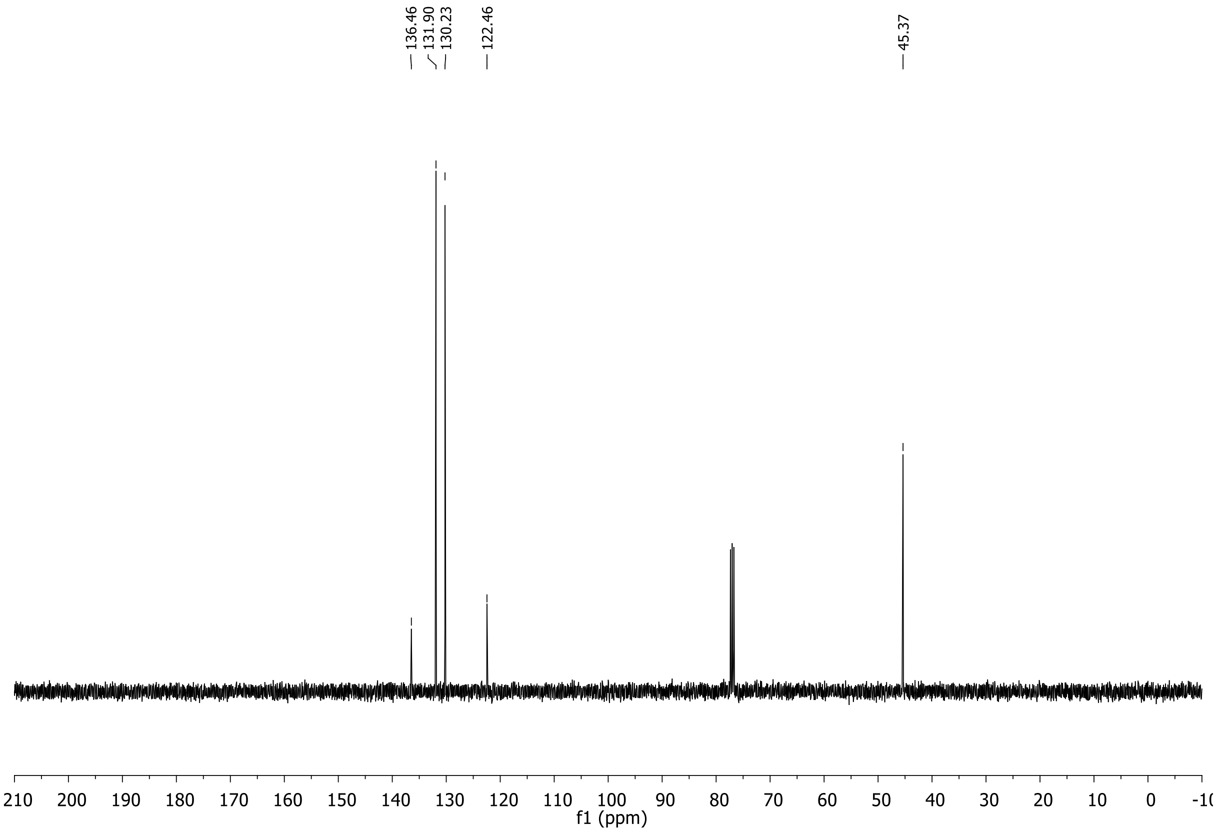

4-Bromobenzyl bromide (**75**)

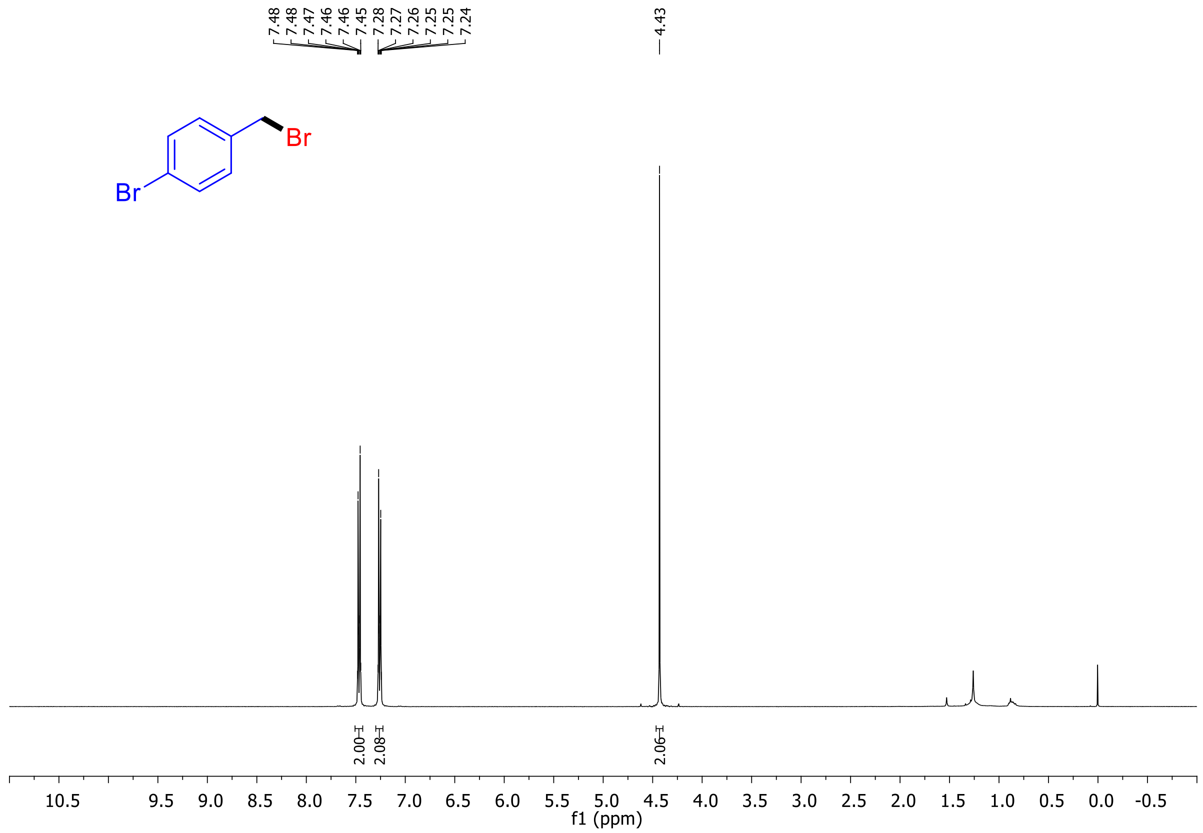

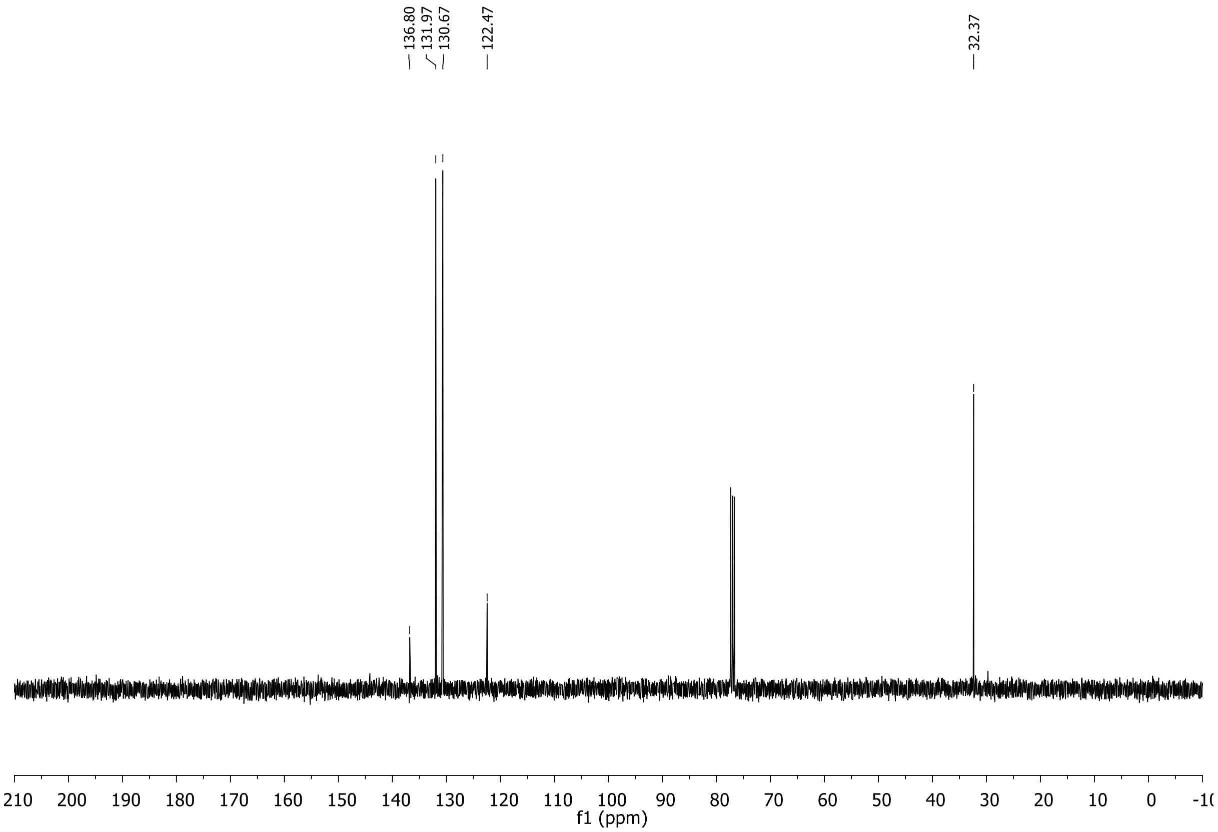

2-(4-Chlorophenyl)ethyl chloride (**76**)

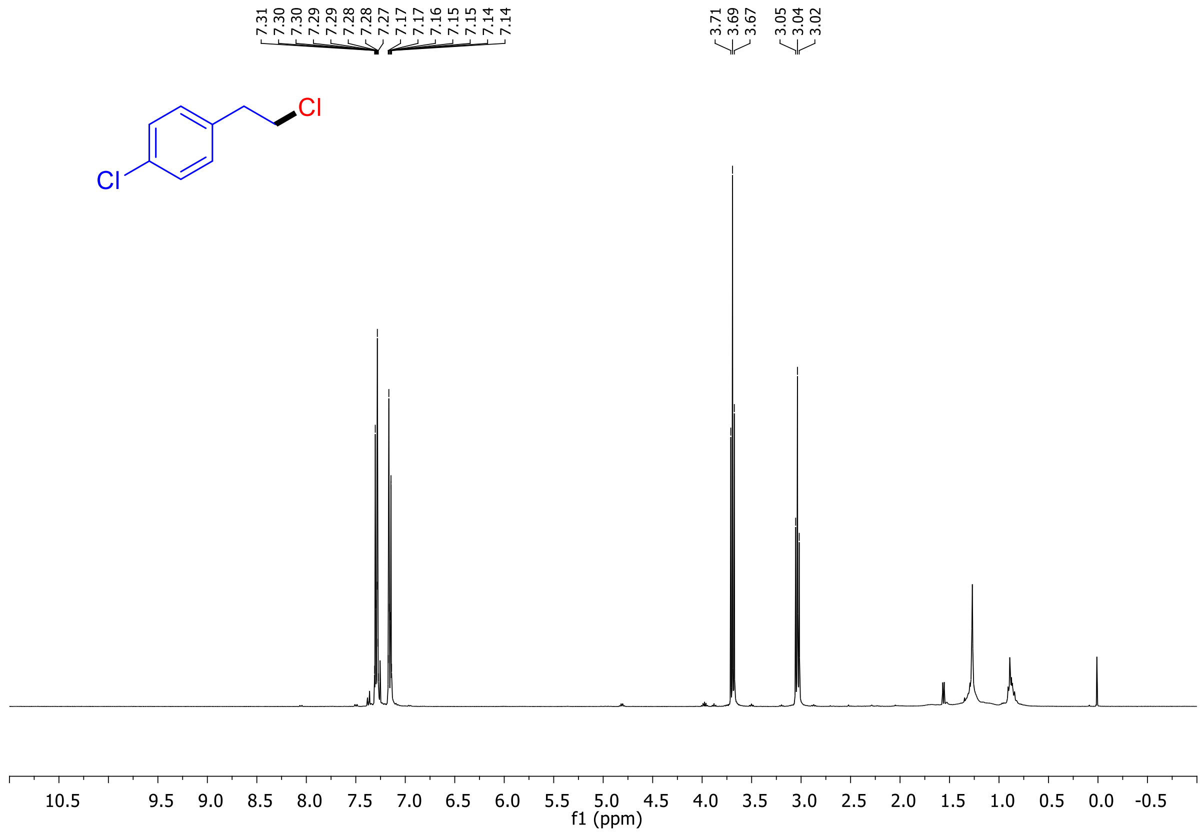

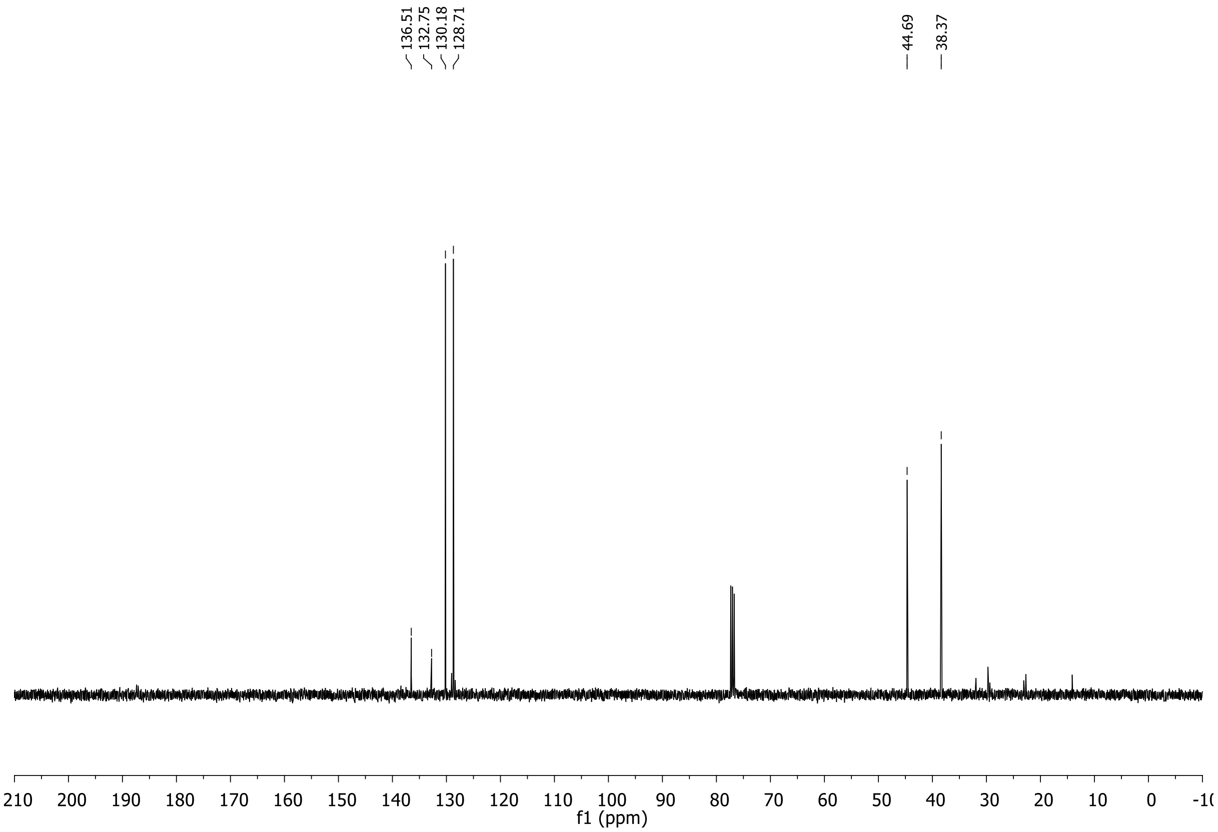

2-(4-Chlorophenyl)ethyl bromide (**77**)

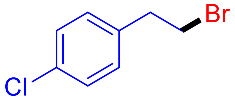

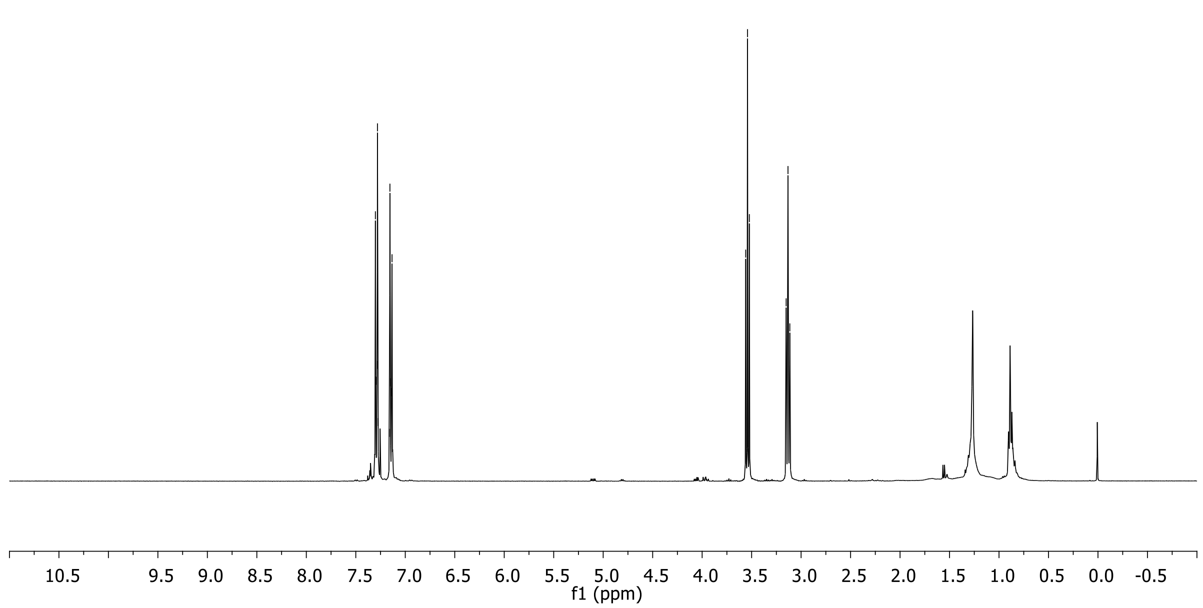

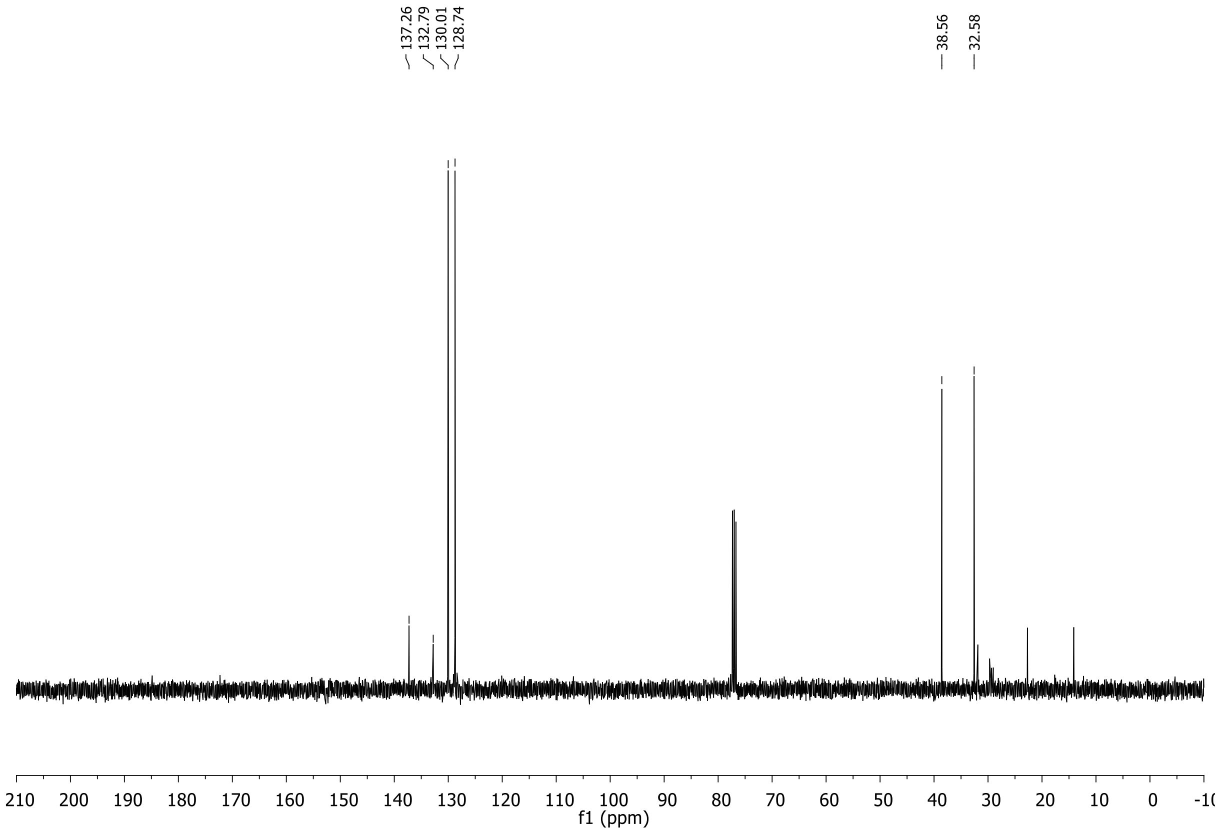

3-(1-Adamantyl)pentane-2,4-dione (**78**)

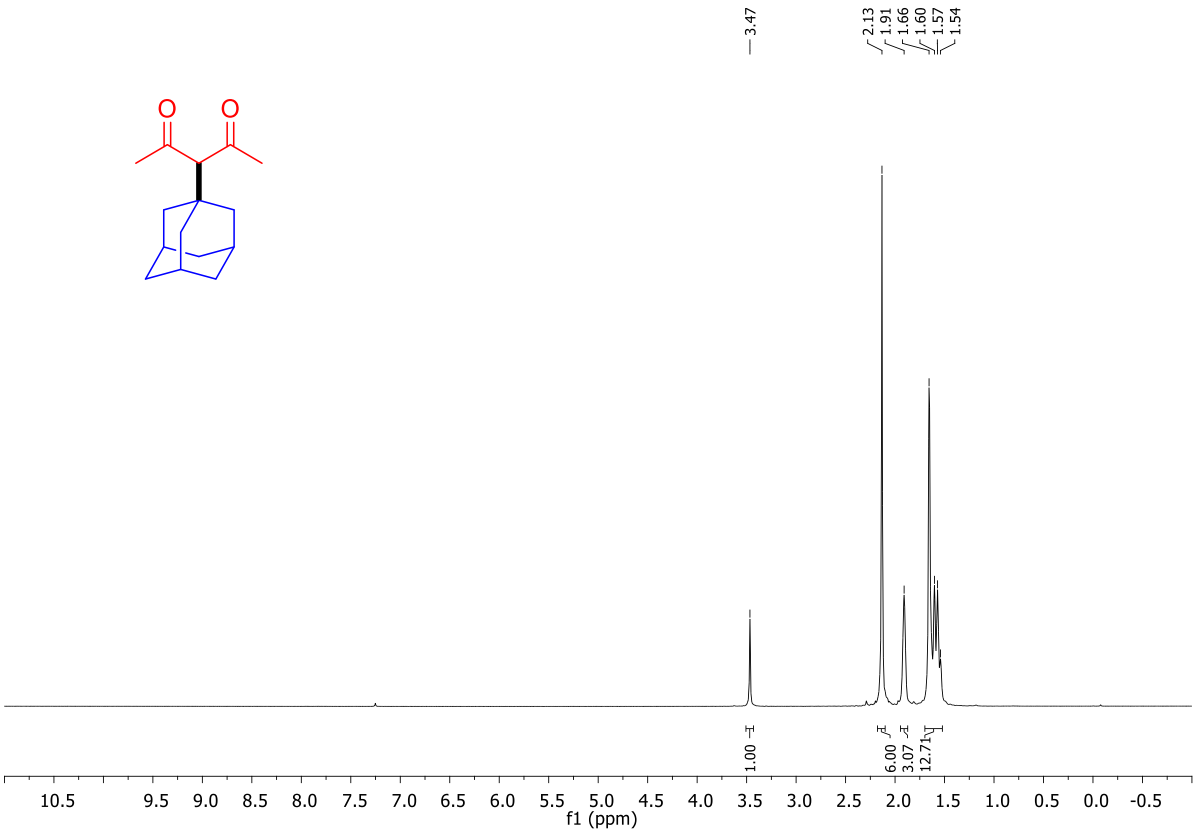

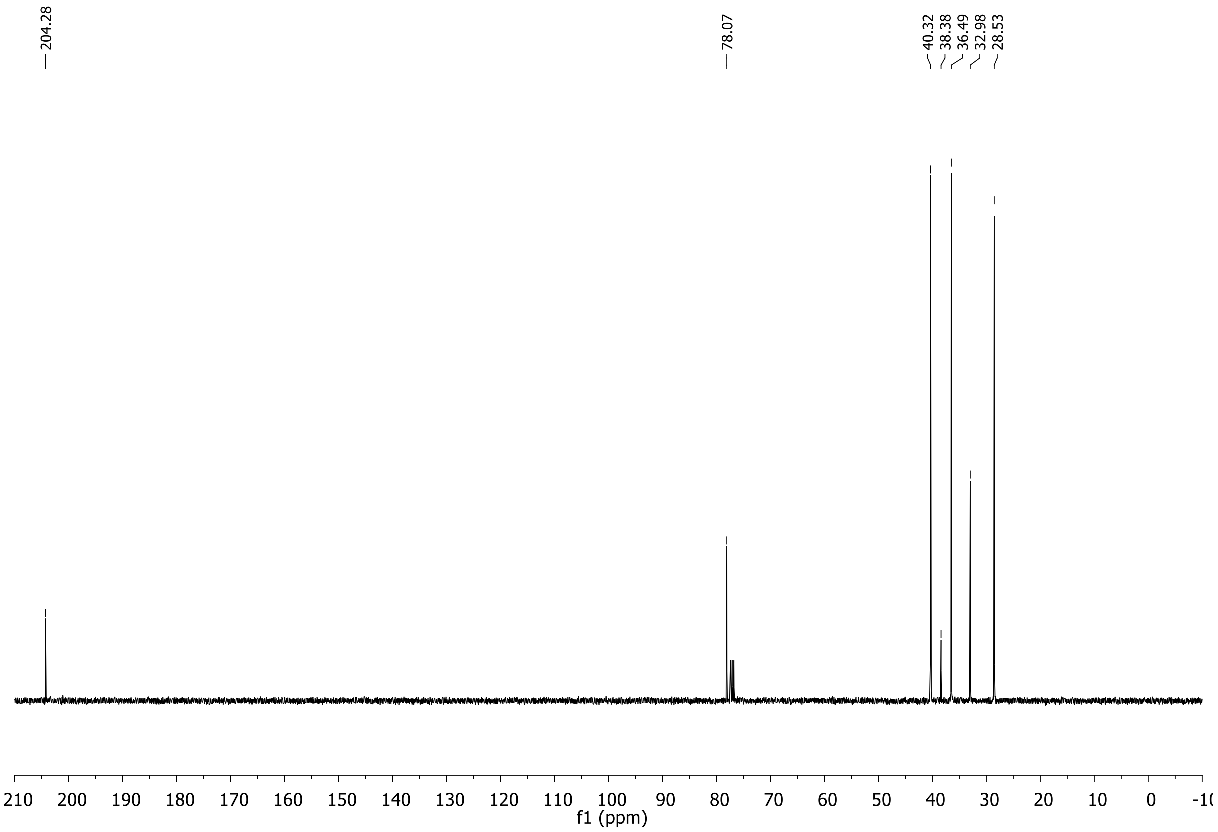

Ethyl 2-(adamantan-1-yl)-3-oxobutanoate (**79**)

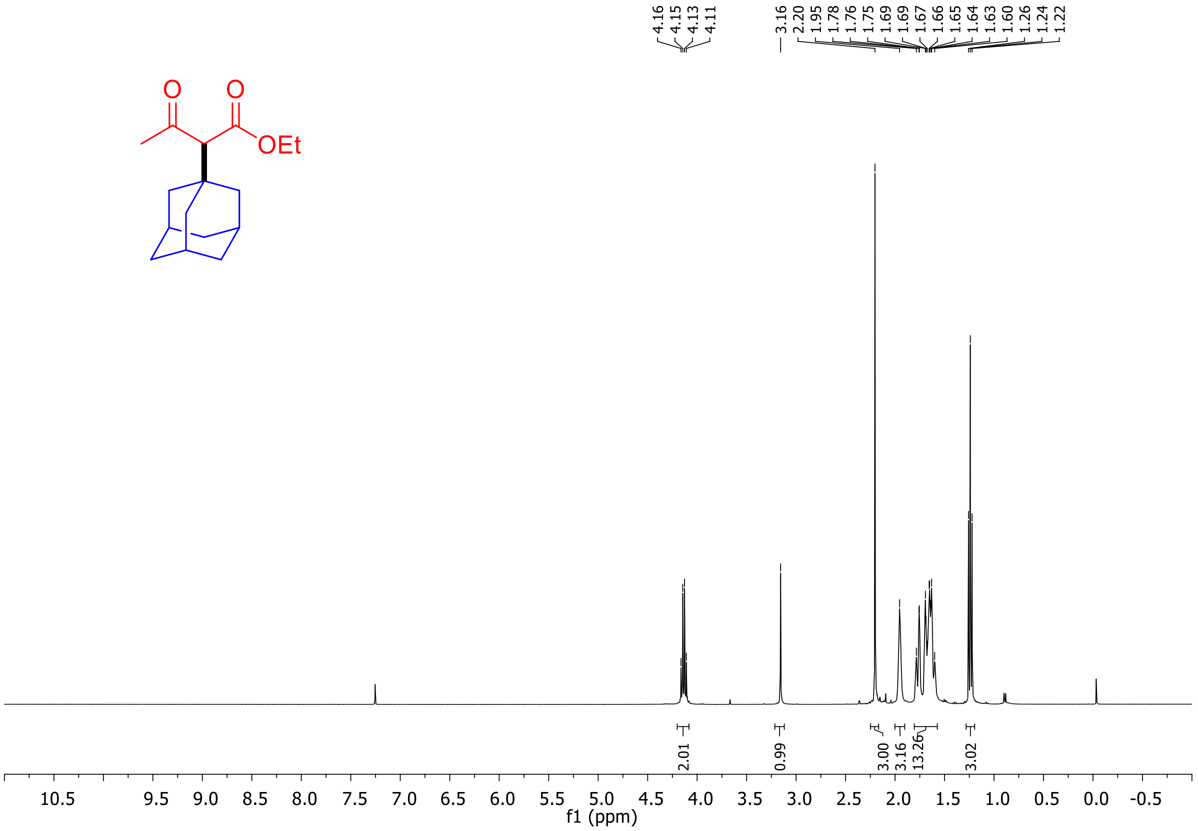

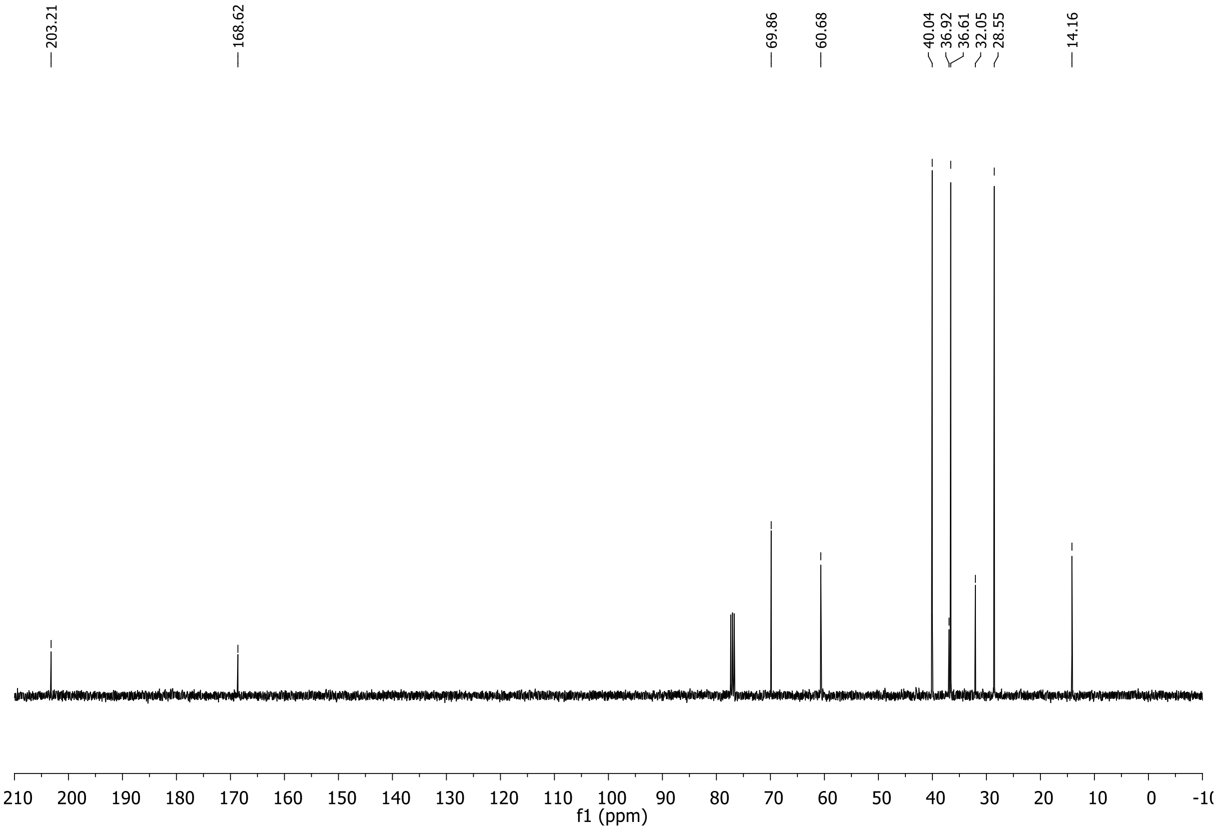

1-Adamantanecarboxylic acid (**80**)

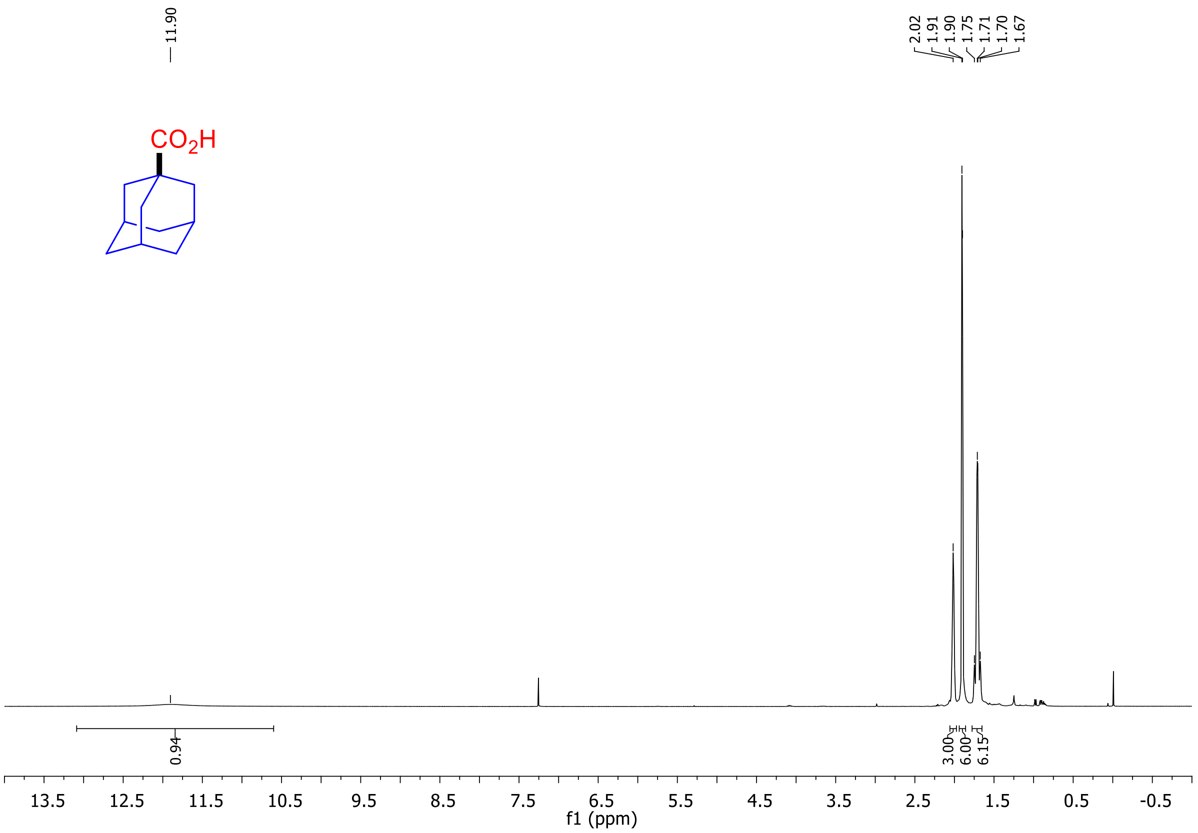

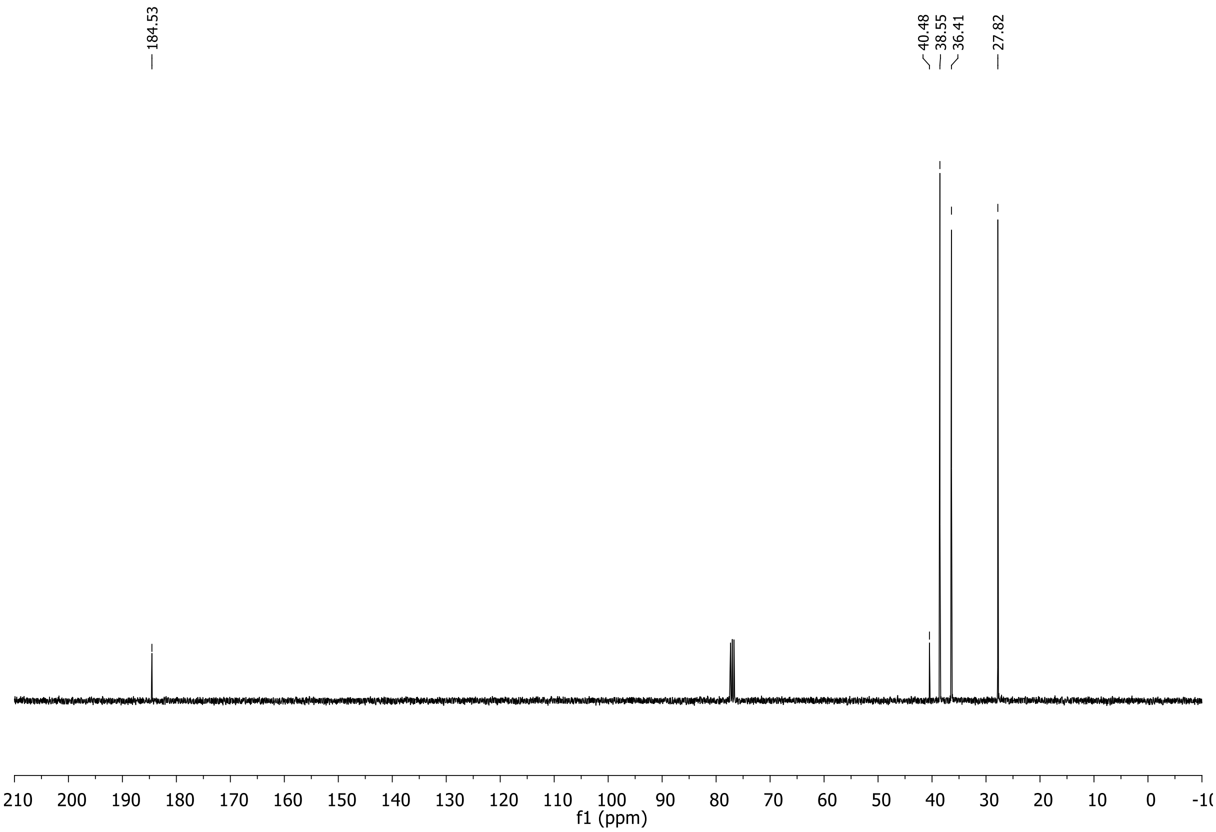

7. References

(1) Lv, F.; Xiao, J.; Xiang, J.; Guo, F.; Tang, Z.-L.; Han, L.-B. *J. Org. Chem.* **2021**, *86* (3), 3081–3088.

(2) Bering, L.; Jeyakumar, K.; Antonchick, A. P. *Org. Lett.* **2018**, *20* (13), 3911–3914.

(3) Khodaei, M. M.; Nazari, E. *Tetrahedron Lett.* **2012**, *53* (38), 5131–5135.

(4) Sun, H.-B.; Li, B.; Chen, S.; Li, J.; Hua, R. *Tetrahedron* **2007**, *63* (41), 10185–10188.

(5) Mo, X.; Yakiwchuk, J.; Dansereau, J.; McCubbin, J. A.; Hall, D. G. *J. Am. Chem. Soc.* **2015**, *137* (30), 9694–9703.

(6) Qin, Q.; Xie, Y.; Floreancig, P. E. *Chem. Sci.* **2018**, *9* (45), 8528–8534.

(7) Zhang, S.; Vayer, M.; Noël, F.; Vuković, V. D.; Golushko, A.; Rezajooei, N.; Rowley, C. N.; Lebœuf, D.; Moran, J. *Chem* **2021**, *7* (12), 3425–3441.

(8) Yang, X.-W.; Li, D.-H.; Song, A.-X.; Liu, F.-S. *J. Org. Chem.* **2020**, *85* (18), 11750–11765.

(9) Mannschreck, A.; Ernst, L. *Chem. Ber.* **1971**, *104* (1), 228–247.

(10) Pallikonda, G.; Chakravarty, M. *J. Org. Chem.* **2016**, *81* (5), 2135–2142.

(11) Stepakov, A. V.; Molchanov, A. P.; Kostikov, R. R. *Russ. J. Org. Chem.* **2007**, *43* (4), 538–543.

(12) Khusnutdinov, R. I.; Shchadneva, N. A.; Khisamova, L. F. *Russ. J. Org. Chem.* **2015**, *51* (11), 1545–1550.

(13) Akram, M. O.; Tidwell, J. R.; Dutton, J. L.; Martin, C. D. *Angew. Chemie Int. Ed.* **2022**, *61* (46).

(14) He, Y.; Liu, C.; Yu, L.; Zhu, S. *Angew. Chemie Int. Ed.* **2020**, *59* (23), 9186–9191.

(15) Liu, W.; Li, J.; Querard, P.; Li, C.-J. *J. Am. Chem. Soc.* **2019**, *141* (16), 6755–6764.

(16) Tang, R.-J.; Milcent, T.; Crousse, B. *J. Org. Chem.* **2018**, *83* (22), 14001–14009.

(17) Fujita, H.; Kakuyama, S.; Fukuyoshi, S.; Hayakawa, N.; Oda, A.; Kunishima, M. *J. Org. Chem.* **2018**, *83* (8), 4568–4580.

(18) Sha, S.-C.; Tcyrulnikov, S.; Li, M.; Hu, B.; Fu, Y.; Kozlowski, M. C.; Walsh, P. J. *J. Am. Chem. Soc.* **2018**, *140* (39), 12415–12423.

(19) Primer, D. N.; Molander, G. A. *J. Am. Chem. Soc.* **2017**, *139* (29), 9847–9850.

(20) Zurabishvili, D. S.; Lomidze, M. O.; Samsoniya, S. A.; Wesquet, A.; Kazmaier, U. *Chem. Heterocycl. Compd.* **2008**, *44* (8), 941–949.

(21) Huang, H.-M.; Bellotti, P.; Pflüger, P. M.; Schwarz, J. L.; Heidrich, B.; Glorius, F. *J.*

Am. Chem. Soc. **2020**, *142* (22), 10173–10183.
(22) Meng, S.-S.; Tang, X.; Luo, X.; Wu, R.; Zhao, J.-L.; Chan, A. S. C. *ACS Catal.* **2019**, *9* (9), 8397–8403.
(23) Su, J.; Li, C.; Hu, X.; Guo, Y.; Song, Q. *Angew. Chemie Int. Ed.* **2022**, *61* (52).
(24) Wang, Z.-H.; Wei, L.; Jiao, K.-J.; Ma, C.; Mei, T.-S. *Chem. Commun.* **2022**, *58* (59), 8202–8205.
(25) Yang, L.; Chen, X.; Ni, K.; Li, Y.; Wu, J.; Chen, W.; Ji, Y.; Feng, L.; Li, F.; Chen, D. *Tetrahedron Lett.* **2020**, *61* (29), 152123.
(26) Sämann, C.; Dhayalan, V.; Schreiner, P. R.; Knochel, P. *Org. Lett.* **2014**, *16* (9), 2418–2421.
(27) Wang, N.; Wang, R.; Shi, X.; Zou, G. *Beilstein J. Org. Chem.* **2012**, *8*, 227–233.
(28) Bao, Z.; Zou, J.; Mou, C.; Jin, Z.; Ren, S.-C.; Chi, Y. R. *Org. Lett.* **2022**, *24* (48), 8907–8913.
(29) Tang, Y.; Zhang, Y.; Chen, X.; Xie, X.; Zhou, N.; Dai, Z.; Xiong, Y. *Angew. Chemie Int. Ed.* **2023**, *62* (4).
(30) Basu Baul, T. S.; Dutta, D.; Duthie, A.; Guedes da Silva, M. F. C. *Inorganica Chim. Acta* **2017**, *455*, 627–637.
(31) Reed, J. H.; Cramer, N. *ChemCatChem* **2020**, *12* (17), 4262–4266.
(32) Ramachandran, P. V.; Alawaed, A. A.; Hamann, H. J. *Org. Lett.* **2023**, *25* (25), 4650–4655.
(33) Nair, A.; Tiwari, V.; Rath, S.; Saini, P.; Verma, A.; Elias, A. J. *Chem. Commun.* **2023**, *59* (74), 11117–11120.
(34) Miele, M.; Citarella, A.; Langer, T.; Urban, E.; Zehl, M.; Holzer, W.; Ielo, L.; Pace, V. *Org. Lett.* **2020**, *22* (19), 7629–7634.
(35) Ai, H.; Leidecker, B. N.; Dam, P.; Kubis, C.; Rabeah, J.; Wu, X. *Angew. Chemie Int. Ed.* **2022**, *61* (43).
(36) Khusnutdinov, R. I.; Kislitsina, K. S.; Shchadneva, N. A. *Russ. J. Org. Chem.* **2014**, *50* (10), 1409–1411.
(37) Vil', V. A.; dos Passos Gomes, G.; Bityukov, O. V.; Lyssenko, K. A.; Nikishin, G. I.; Alabugin, I. V.; Terent'ev, A. O. *Angew. Chemie Int. Ed.* **2018**, *57* (13), 3372–3376.
(38) Yu, Y.; Zhai, D.; Zhou, Z.; Jiang, S.; Qian, H.; Ma, S. *Chem. Commun.* **2023**, *59* (35), 5281–5284.
